# Synthesis, Biological Activities and Docking Studies of Novel 4-(Arylaminomethyl)benzamide Derivatives as Potential Tyrosine Kinase Inhibitors

**DOI:** 10.3390/molecules24193543

**Published:** 2019-09-30

**Authors:** Elena Kalinichenko, Aliaksandr Faryna, Viktoria Kondrateva, Alena Vlasova, Valentina Shevchenko, Alla Melnik, Olga Avdoshko, Alla Belko

**Affiliations:** Institute of Bioorganic Chemistry, National Academy of Sciences of Belarus, BY-220141 Minsk, Belarus; farynaa@yandex.ru (A.F.); kondrateva@iboch.by (V.K.); alenavlasova@iboch.by (A.V.); vas_57iboch@mail.ru (V.S.); al_melk50@mail.ru (A.M.); olga.avdoshko@iboch.by (O.A.); alyabelko@iboch.by (A.B.)

**Keywords:** 4-(aminomethyl)benzamide, Bcr-Abl, protein kinase inhibitors, receptor tyrosine kinases, molecular docking, molecular dynamics, anticancer activity

## Abstract

A number of new compounds containing the 4-(aminomethyl)benzamide fragment as a linker were designed and synthesized, and their biological activities were evaluated as potential anticancer agents. The cytotoxicity activity of the designed compounds was studied in two hematological and five solid cell lines in comparison with the reference drugs. Targeted structures against eight receptor tyrosine kinases including EGFR, HER-2, HER-4, IGF1R, InsR, KDR, PDGFRa, and PDGFRb were investigated. The majority of the compounds showed a potent inhibitory activity against the tested kinases. The analogues **11** and **13** with the (trifluoromethyl)benzene ring in the amide or amine moiety of the molecule were proven to be highly potent against EGFR, with 91% and 92% inhibition at 10 nM, respectively. The docking of synthesized target compounds for nine protein kinases contained in the Protein Data Bank (PDB) database was carried out. The molecular modeling results for analogue **10** showed that the use of the 4-(aminomethyl)benzamide as a flexible linker leads to a favorable overall geometry of the molecule, which allows one to bypass the bulk isoleucine residue and provides the necessary binding to the active center of the T315I-mutant Abl (PDB: 3QRJ).

## 1. Introduction

Protein kinases serve as therapeutic targets for a range of clinical indications and represent the largest category of drug targets in current clinical trials [1]. The first-generation Bcr-Abl inhibitor imatinib achieved great clinical success and became the first-line drug for the treatment of chronic myeloid leukemia (CML) [2]. Despite advances in treatment with imatinib, some patients are initially insensitive to the drug, or, amid ongoing therapy with imatinib, they lose the achieved response [3,4,5]. After the introduction of imatinib, the therapeutic armamentarium in solid malignancies was expanded by the registration of several other tyrosine kinase inhibitors (TKIs). These include the epidermal growth factor receptor (EGFR) inhibitors erlotinib and gefitinib, the dual EGFR and human epidermal growth factor receptor (HER)-2 inhibitor lapatinib, and the VEGFR (vascular endothelial growth factor receptor) inhibitors sunitinib, sorafenib, and, recently, pazopanib. Importantly, none of the TKIs are entirely specific for one target. In particular, the VEGFR inhibitors target a wide spectrum of kinases, including the platelet-derived growth factor receptor (PDGFR) and fibroblast growth factor receptor (FGFR).

The progress in kinase structural biology offers a conceptual framework for understanding many aspects of kinase biology and accelerating drug discovery programs targeting protein kinases [6,7]. The structure of human Abl in complex with imatinib shows that the molecule binds in the cleft between the N- and C-terminal lobes [8]. A number of approved drugs which are type I inhibitors, such as gefitinib, target the ATP binding pocket and are termed as ATP competitive inhibitors. Type II inhibitors, which include imatinib, nilotinib, and ponatinib, bind to the hydrophobic pocket adjacent to the ATP binding pocket, which is accessible only in a DFG (Asp-Phe-Gly)-out conformation of the enzyme. Although the occupancy at the allosteric site is characteristic of type II inhibitors, they also extend past the “gatekeeper” into the adenine pocket and form hydrogen bonds with the “hinge” residues [9,10,11].

**Figure 1:**
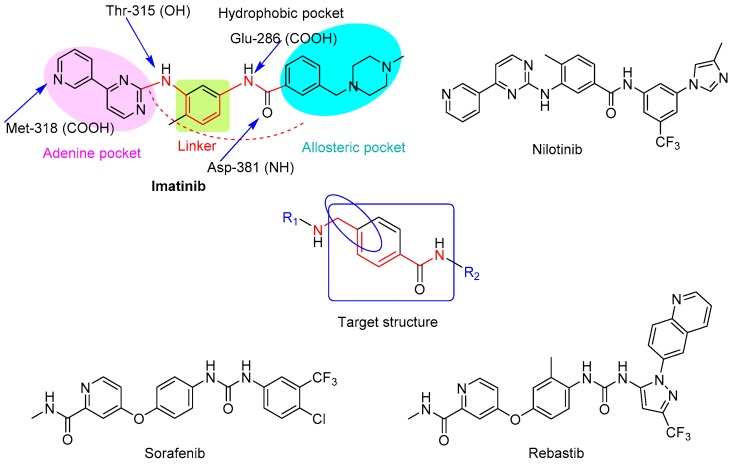
Inhibitors of the tyrosine kinase type II and target structure. Interactions between imatinib and Brc-Abl tyrosine kinase.

Imatinib joins in the ATP pocket of the Bcr-Abl protein mainly with its pyridine–pyrimidine fragment, next to which there is a methylphenyl group located opposite the “gatekeeper” residue, Thr-315, while the methyl piperazinyl group of the molecule is located superficially mostly in the pocket of specificity (Figure 1). A hydrogen-bond interaction forms between the hydroxyl group of Thr-315 and the secondary amine linking the methylphenyl group to the pyrimidine moiety. The benzamide moiety packs against the residue Leu-293 in this pocket, and the carbonyl group in the benzamide moiety forms a hydrogen bond with the amide group of the residue Asp-381. In addition, an amine in the piperazine group forms a hydrogen bond with the carbonyl group of Ile-360 [12].

Despite high response rates, CML patients treated with imatinib often develop resistance to the drug from point mutations in the kinase domain of Bcr-Abl that disrupt imatinib binding directly [13]. An additional structure–activity relationship study around the core phenylaminopyrimidine scaffold of imatinib led to a more potent Bcr-Abl inhibitor, nilotinib. The higher binding affinity of nilotinib for Abl derives from van der Waals interactions with the trifluoromethyl and imidazole substituents on the phenyl ring, which are located in the pocket of specificity [12].

One key mutation against which nilotinib is inactive is T315I, the gatekeeper mutation. Ponatinib was designed to overcome this type of mutation [14,15]. The key feature of the drug is a carbon–carbon triple bond between the methylphenyl and purine groups that can accommodate the isoleucine side chain without steric interference [11].

However, resistance to ponatinib was observed in some patients, making the development of new T315I-targeted inhibitors a necessity. The new drug rebastinib demonstrates the potential for treating patients with relapsed chronic myeloid leukemia and acute myeloid leukemia [16].

Being a drug of new generation, rebastib is a “switch control” inhibitor of several tyrosine kinases, including ABL1 (Abelson tyrosine kinase 1), FLT3 (FMs-related tyrosine kinase 3), and TIE2 (tunica interna endothelial cell kinase 2). Rebastinib binds as a type II inhibitor that additionally penetrates into and binds the switch control pocket of the ABL1 kinase domain, key amino-acid residues that mediate the change from inactive to active conformation. Through occupancy of this switch control pocket, rebastinib blocks the conformational process required for kinase activation. This unique binding mode enables rebastinib to maintain the ABL1 kinase domain in the inactive conformation, independent of the state of phosphorylation of the regulatory Tyr-393. [17].

Sorafenib is a clinically approved drug that is a type II as well. While this drug inhibits several kinases, including many receptor tyrosine kinases, VEGFR was implicated as the primary target, in the context of the approved drug indications for renal cell carcinoma and colorectal cancer.

In a crystal structure of sorafenib-bound VEGFR kinase domain, the kinase domain adopts a “DGF-out”, activation loop-in conformation, resembling the inactive conformation of Abl. Sorafenib forms extensive interactions with VEGFR in this conformation. Notably, the trifluoromethylphenyl ring is tightly packed into the specificity pocket. This binding mode of the trifluoromethylphenyl ring also resembles the one in the nilotinib-bound Abl kinase domain [18].

Compounds shown in Figure 1 are ATP-competitive inhibitors, classified as type II inhibitors, by reference to the conformation of the highly conserved aspartate–phenylalanine–glycine (DFG) motif in the beginning of the activation loop in the C-lobe of the kinase domain [19].

Based on the structural analysis of imatinib, nilotinib, and their analogues (Figure 1), we started the development of new Bcr-Abl inhibitors by applying a combination strategy of bio-isosteric replacement and conformational restriction. The structural modification focused on two parts: the heterocyclic fragment binding to the adenine pocket and forming a hydrogen bond with the amino acids Met-318 and Thr-315 and the modification of the “tail area”, introducing various aromatic and acyclic substituents. As a “linker” between adenine and allosteric pockets the methylphenyl group is used as a rule (Figure 1). We proposed to use a flexible “linker” 4-(aminomethyl)benzamide to avoid a steric collision with the remainder of Ile-315 Bcr-Abl. In general, the structure suggested the possibility of developing specific inhibitors of protein kinase type II binding, in which the allosteric pocket of the protein active site is involved in ligand binding. Considering these assumptions, we designed and synthesized 22 novel target compounds as potential TKIs.

## 2. Results and Discussion

### 2.1. Chemistry

**Scheme 1:**
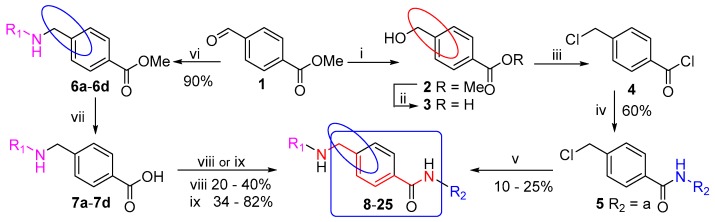

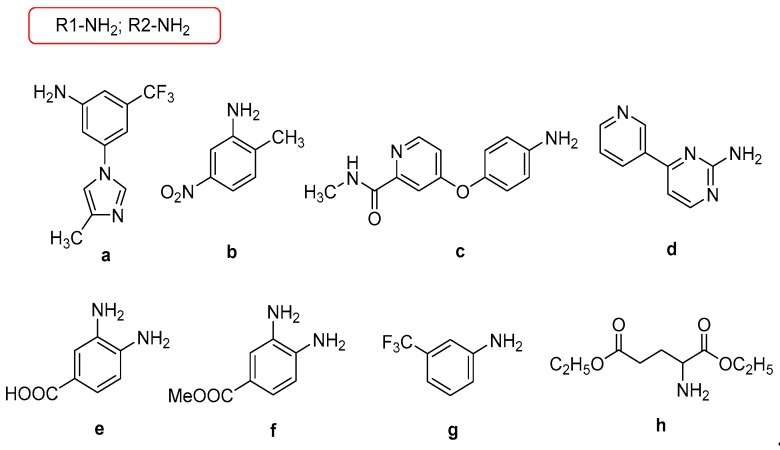
Reagents and conditions: (i) NaBH_4_, MeOH; (ii) KOH, MeOH/H_2_O; (iii) SOCl_2_, CHCl_3_, reflux; (iv) R_2_NH_2_ (**a**), Et_3_N, CHCl_3_; (v) R_1_NH_2_ (**a/b/c/e/g)**, DMF, reflux; (vi) R_1_NH_2_ (**a**–**d)**, NaBH(OAc)_3_, AcOH, CHCl_3_; (vii) HCl (18–27%), 80 °C; (viii) (1) SOCl_2_, CHCl_3_, (2) R_2_NH_2_ **(a/b/g/h)**, NEt_3_, CHCl_3_; (ix) R_2_NH_2_ **(a/c/f/g/h)**, DCC (CDI), BtOH, NEt_3_, DMF.

Two general strategies were developed to obtain target compounds starting from initial methyl 4-formylbenzoate (**1**) by introducing other functional moieties (Scheme 1).

In the first approach, the initial methyl 4-formylbenzoate (**1**) was converted to benzoyl chloride **4** with a quantitative yield by known methods [20,21]. A key intermediate product **5** was obtained by the interaction of **4** with amine **a** in chloroform with 60% yield. Compounds **8**–**12** were synthesized by the reaction of primary amines **a/b/c/e/g** with the 4-(chloromethyl)benzamide (**5**) with moderate yields (10–25%).

A previously described synthetic method of the reductive amination [22] was used in the second approach. The reaction between aldehyde **1** and primary amines **a**–**d** in the presence of triacetoxyborohydride sodium and acetic acid proceeded at room temperature with the formation of the amines **6a**–**6d** with high yield (80–95%). Treatment of **6a**–**6d** with an aqueous solution of HCl (18–27%) at 80–90 °C allowed obtaining derivatives **7a**–**7d** with high yield (80–85%).

Two ways were studied to obtain the targeted compounds **13**–**25** with the aim of the yield optimizing. The first way was to obtain the intermediate acyl chlorides by the action of thionyl chloride on carboxyl derivatives **7a**–**7d**, which were then directly introduced into the reaction with the corresponding amine **a/b/g/h** in the presence of triethylamine. Yields of target amides **10, 13**–**15,** and **22**–**25** were 20–50% after isolation and purification by column chromatography. The second way was to activate the carboxyl group of compounds **7a**–**7d** with the use of condensing agents *N*,*N’*-dicyclohexylcarbodiimide (DCC) or 1,1’-carbonyldiimidazole (CDI) in the presence of NEt_3_/BtOH and the subsequent addition of primary amines **a/c/f/g/h**. This approach made it possible to obtain target amides **16**–**21** with a fairly good yield (34–82%) (Table 1).

**Scheme 2:**
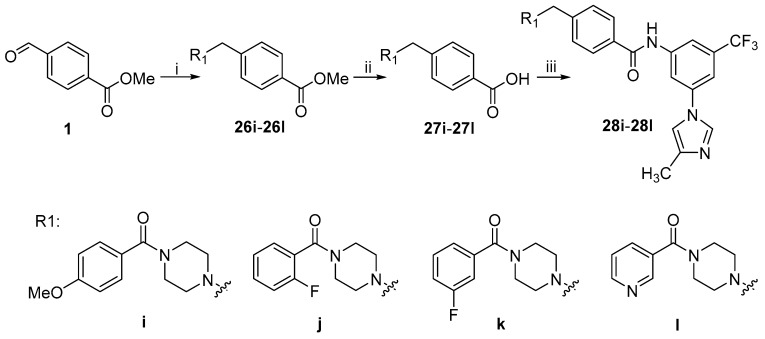
Reagents and conditions: (i) R_1_H (**i/j/k/l**), NaBH(OAc)_3_, AcOH, CHCl_3_; (ii) HCl (18–27%), 80 °C; (iii) (1) SOCl_2_, CHCl_3_, (2) RNH_2_ (**a)**, NEt_3_, CHCl_3_.

The synthesis of compounds **28i**–**28l** is summarized in Scheme 2. Derivatives **26i**–**26l** were prepared by the method of reductive amination. Amidation of the corresponding intermediates **27i**–**27l** with amine **a** gave targeted compounds **28i**–**28l**. The selected amines were chosen on the basis of their lipophilicity and limited number of possible conformations, providing more lipophilic and rigid final compounds **28i**–**28l**.

All target compounds were isolated as free bases. In most cases, crystallization or column chromatography was required for the purification. The structures of most synthetic intermediates and products were established by spectroscopy techniques (^1^H-, ^13^C-NMR) and high-resolution mass spectrometry (HRMS). The purities of compounds were 95–99.00% (by HPLC).

### 2.2. Biological Studies

#### 2.2.1. Anti-Proliferation Activity

To determine the selectivity of the synthesized compounds **8**–**25** and **28i**–**28l** toward various cancer cells and normal human cells, the anti-proliferative activities against K-562 (chronic myelogenous leukemia), HL-60 (promyelocytic leukemia), MCF-7 (breast adenocarcinoma), HeLa (cervix carcinoma), A549 (lung carcinoma), OCD-GS (carcinoma of the kidney), and normal cells RPMI 1788 (B lymphocyte, the human cell line), were compared with imatinib, nilotinib, and sorafenib as the positive control. All new compounds showed moderate to significant activity against two or more hematologic and/or solid tumor cell lines in 100 µM concentration. Compounds **5, 8**–**11, 13**–**15, 19, 23** and **28i**–**28l**, which showed activity against more than two cell lines, were further studied in vitro for the anti-proliferative activity at a concentration of 0.1–100 μM (Table 2).

It was found that most compounds displayed a moderate suppression on the growth of the positive human chronic myelogenous leukemia K562 cells expressing a high level of Bcr-Abl protein and Bcr-Abl-negative leukemia cells (line HL60). Generally, they moderately inhibited solid tumor cell cultures. As shown in Table 2, compounds **10** and **15** turned out to be more active against HL60 (IC_50_ values 8.2 μM and 5.6 μM, respectively) compared to K562 cells (40 μM and 31 μM). Analog **13** showed activity against K562 with the value IC_50_ = 5.6 μM. Among the series of tertiary heterocyclic amines, compounds **28j**–**28l** exhibited the highest potency against K562 cells with IC_50_ values 6.9 μM, 3,6 μM, and 4.5 μM, respectively. Promyelocytic leukemia cell line (HL60) survived at much higher concentrations of **28j**–**28l** indicating the specificity of these compounds. We supposed that the introduction of the [4-(benzoyl)piperazin-1-yl]methyl group including tertiary heterocyclic amine could improve the lipophilicity which led to the high permeability [23]. Log P of imatinib was 3.89, while amides **28i**–**28l** had log P values between 2.65 and 4.02 (Table 2).

#### 2.2.2. Kinase Inhibitory Assays

We investigated the kinase selectivity of targeted compounds against eight receptor tyrosine kinases including EGFR (epidermal growth factor receptor), HER-2 and HER4 (human epidermal growth factor receptor), IGF1R (insulin like growth factor 1 receptor), InsR (gene insulin receptor), KDR (kinase insert domain receptor), and PDGFRa and PDGFRb (platelet-derived growth factor receptors). The assay was performed using the Kinase-Glo Plus luminescence kinase assay kit (TK-1+ADP-Glo™, Promega, San Diego, CA, USA). It measures kinase activity by quantitating the amount of ATP remaining in solution following a kinase reaction. The inhibition percentage of the enzymatic activities caused by our compounds was evaluated at the single concentration of 10 nM against imatinib. The obtained results are given in Table 3.

As follows from the obtained data, analogues **11** and **13** with *N*-(trifluoromethyl)benzene moiety in the amide or amine moiety revealed their high potency against EGFR, 91% and 92%, respectively. Compounds **10**, **20**, **22**, and **24** also showed significant inhibitory activity EGFR, which was between 21% and 65%, as illustrated in Table 3. Target products **10**, **11**, **13**, **18**, **20**, **22**, **24**, **28k**, and **28l** exhibited inhibitory activity comparable to imatinib in relation to HER-4, a receptor tyrosine kinase of the EGFR family. 

Vascular endothelial growth factor receptor 2 (VEGFR2), also called kinase insert domain receptor (KDR), is a tyrosine kinase receptor for VEGFs that plays a central role in tumor angiogenesis; therefore, the inhibition of VEGFR2 is a promising therapeutic strategy for inhibiting angiogenesis and tumor growth. Compounds **10**, **11**, **13**, **18**, **20**, **22**, **28k**, and **28l** showed inhibitory activity of VEGFR2 in the range of 16–48% at a concentration of 10 nM.

Compounds **18** and **20** containing the *N*-[3-(trifluoromethyl)phenyl]benzamide fragment exhibited a high inhibitory activity against PDGFRa, 67% and 77%, respectively, despite various substituents in the amine fragment. The influence of the difference in ATP binding sites between various tyrosine kinase receptors is obvious, yet not very clear. Perhaps these features can explain why compounds **5**, **14**, **15**,**17**, **28i**, and **28j** showed high selectivity for the gene insulin receptor, whereas synthetic products **10**, **11**, **13**, **18**, **20**, **22**, **24**, **28k**, and **28l** had significant influence on the biochemical activities of EGFR, HER-4, VEGFR-2, and PDGFRa.

### 2.3. Docking and Molecular Dynamics

An in-depth in silico study of the inhibitory activity of synthesized compounds **8**–**25** and **28i**–**28l** to various protein kinases was implemented in comparison with some known inhibitors aiming the investigation of a possible binding mechanism.

Initially, docking was performed to obtain protein–ligand complexes of the studied structures **8**–**25** and **28i**–**28l** with a variety of tyrosine kinases. AutoDock Vina was used as a docking tool [24]. Structures of receptors were taken from Protein Data Bank (PDB) complexes of known protein kinase inhibitors: human ABL kinase (PDB record 3CS9), human ABL kinase (2HYY), VEGF-receptor (3WZE), and T315I-mutant Abl kinase (3QRJ). Imatinib (the original ligand in the complex 2HYY), nilotinib (3CS9), rebastinib (3QRJ), sorafenib (3WZE), and ponatinib were used as reference structures. Docking results showed that AutoDock Vina correctly reproduced the experimental binding models of imatinib, nilotinib, sorafenib, and rebastinib with RMSDs (root mean squared deviation of heavy atoms) of less than 2 Å. Nilotinib (−13.2) showed the best docking score and sorafenib (−11.5) was the worst (Table 4). The obtained protein–ligand complexes having docking scores better than −11.0 were taken to the 2-ns molecular dynamics (MD) simulations using GROMACS [25].

Based on the molecular dynamics trajectories, the binding energy for each complex was estimated by the MM-PBSA method with decomposition to polar interactions, apolar van der Waals (VdW) interactions, and polar solvation energies. Energy calculations were carried out using g_mmpbsa [26]. In terms of binding energy, the studied structures showed generally worse results but in some cases comparable ones with known inhibitors. For reference, the structure with rebastinib showed the best binding energy of −178.0 ± 14.2 kJ/mol in complex with T315I-mutant tyrosine kinase. The lowest values of binding energies were obtained for imatinib (−154.7 ± 12.3 kJ/mol, 2HYY) and ponatinib (−149.3 ± 20.2 kJ/mol, 2HYY). The results of binding energy estimations are summarized in Table 5.

**Figure 2:**
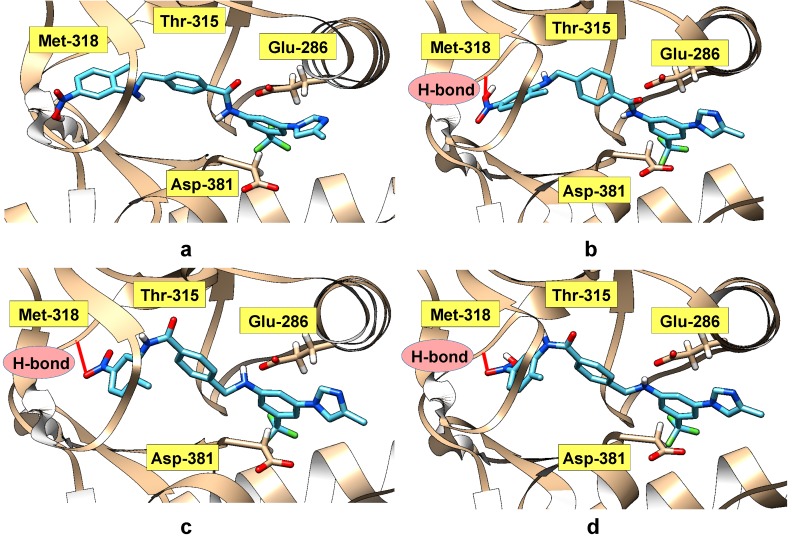
Binding models of inhibitor **9** and **15** to Abl-wt kinase (wild-type Abl structure). Hydrogen bonds (H-bonds) are indicated by red lines to key amino acids. (**a**) Binding model of **9** with Abl (Protein Data Bank (PDB): 3CS9; H-bond—non-detected). (**b**) Binding model of **9** with Abl (PDB: 2HYY; H-bond—Met-318). (**c**) Binding model of **15** with Abl (PDB: 3CS9; H-bond—Met-318). (**d**) Binding model of **15** with Abl (PDB: 2HYY; H-bond—Met-318).

**Figure 3:**
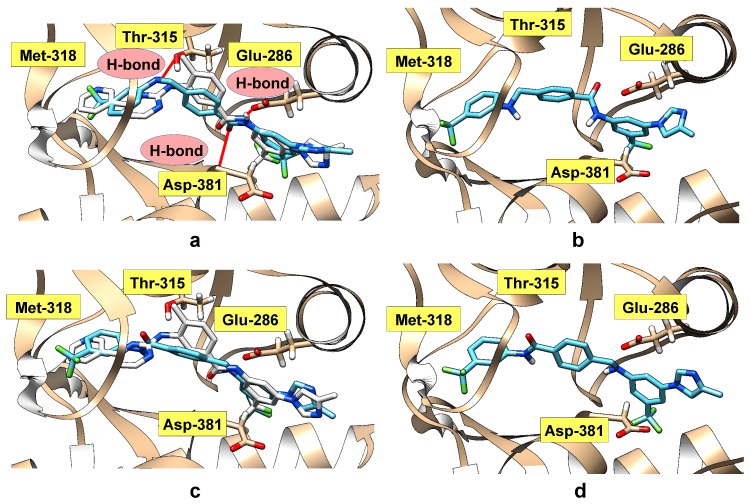
Binding models of inhibitor **11** and **13** to Abl-wt kinase. Hydrogen bonds are indicated by red lines to key amino acids. Experimental binding model of nilotinib is shown in white (**a, c**). (**a**) Binding model of **11** with Abl (PDB: 3CS9; H-bond—Thr-315, Glu-286, Asp-381). (**b**) Binding model of **11** with Abl (PDB: 2HYY; H-bond—non-detected). (**c**) Binding model of **13** with Abl (PDB: 3CS9; H-bond—non-detected). (**d**) Binding model of **13** with Abl (PDB: 2HYY; H-bond—non-detected).

**Figure 4:**
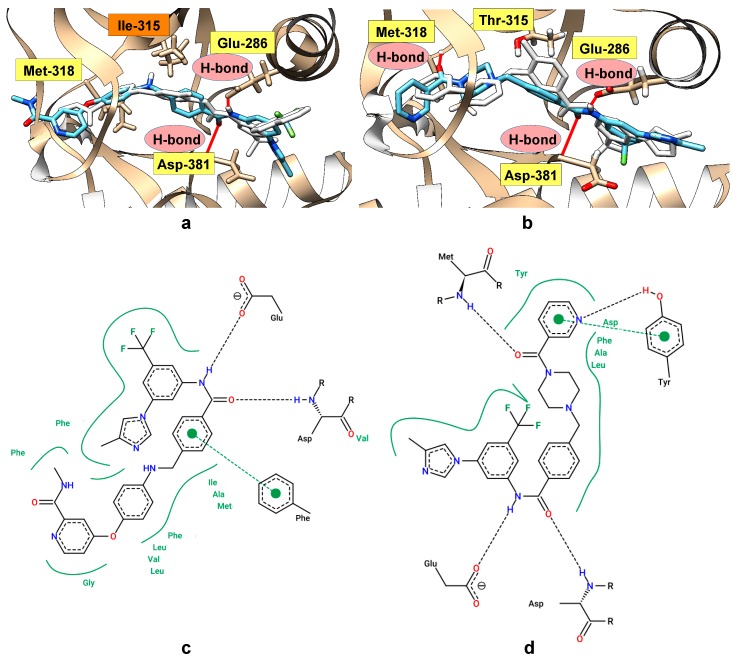
Binding models of inhibitor **10** and **28l** to Abl kinase. Hydrogen bonds are indicated by red lines to key amino acids. (**a**) Binding model of **10** with T315I-mutant Abl (PDB: 3QRJ; H-bond—Asp-381, Glu-286). Experimental binding model of rebastinib is shown in white. (**b**) Binding model of **28l** with Abl-wt (PDB: 3CS9; H-bond—Met-318, Asp-381, Glu-286). Experimental binding model of nilotinib is shown in white. (**c**) Two-dimensional interactions map of **10** with T315L-mutant Abl (PDB: 3QRJ; H-bond—Asp-381, Glu-286). (**d**) Two-dimensional interactions map of **28l** with Abl-wt (PDB: 3CS9; H-bond—Met-318, Asp-381, Glu-286, Tyr-253).

To further analyze the interactions between the inhibitors and Abl-wt kinase, more specific docking details are given in Figure 2, Figure 3 and Figure 4.

The comparable binding modes for the structures having the same substituents but attached to the benzyl group via amine or amide bonds, as in the case with pairs of compounds **9** and **15** or **11** and **13** to Abl-wt kinases, are presented in the Figure 2. As follows from the results of molecular docking, the analogue **9** containing the 2-methyl-5-nitroaniline fragment exhibited a higher binding affinity (−11.4) to Abl (PDB 2HYY) compared to the *N*-(2-methyl-5-nitrophenyl)benzamide derivative **15** (−10.4). In both cases, AutoDock Vina considered a binding advantage to place the trifluoromethyl and imidazole substituents of the phenyl ring in the allosteric pocket, regardless of amide and amine bonds placement (Figure 2). In addition, the nitro group in the 2-methyl-5-nitrophenyl ring formed a hydrogen bond with the amino group of Met-318 (Figure 2b,d).

The docking studies of compounds **11** and **13** and nilotinib were carried out. As in the case of nilotinib, the *N*-arylimidazole group of the abovementioned analogues was located in the allosteric pocket, regardless of the connection type (Figure 3). Unlike the rigid methylphenyl group, the flexible linker avoids the clash with the gatekeeper residue which often occurs in kinase resistance mutations. The geometry of molecules **11** and **13** is more flattened due to the conformational mobility of the 4-(aminomethyl)benzamide, which apparently leads to a closer interaction with the ABL kinase active site (Figure 3a,c).

The experimental binding model shows that analog **11** can form three hydrogen bonds: between the secondary amine of the 3-(trifluoromethyl)aniline fragment and Thr-315 residue, and between the residues Glu-286 and Asp-381 and the amide fragment of linker (Figure 3a). Compound **11** (Table 4) is characterized by a higher estimated binding energy (−151.5 ± 11.8 kJ/mol) in relation to Abl-wt kinase (PDB: 3CS9) compared to the isomeric molecule **13** (−117.9 ± 17.6 kJ/mol) and completely comparable to ponatinib (−149.3 ± 20.2 kJ/mol, PDB 3CS9).

Thr-315 residue is a critical point for the binding of most Bcr-Abl inhibitors, regardless of whether they interact with the active or inactive conformation of the kinase [27,28,29,30]. Compound **10** demonstrated a high estimated binding energy against T315I-mutant Abl (−160.0 ± 13.7 kJ/mol), although slightly inferior to rebastinib (−178.0 ± 14.2) (Table 5). Docking of designed analog **10** with T315I Bcr-Abl (PDB code 3QRJ) was performed with rebastinib as the control (Figure 4a,c).

Our computational research shows that the use of the 4-(aminomethyl)benzamide as a flexible linker leads to a favorable overall geometry of the molecule comparable to rebastinib, which allows one to bypass the bulk isoleucine residue and provides the necessary binding to the active center of the T315I-mutant Abl (PDB 3QRJ) (Figure 4a). The docking-simulated conformation of the compound **10** in the active center of the mutant protein suggests the possibility of forming hydrogen bonds with amino acids Glu-286 and Asp-381. MD simulations showed high level of van der Waals interactions of trifluoromethyl and imidazole groups in the allosteric fragment. In dynamics, there is also possibility of formation of several hydrogen bonds in the ATP site due to the presence of a large number of heteroatoms (Figure 4c, Table 5).

Compounds **28i**–**28l** are characterized by the presence of a tertiary amino group of piperazine as a part of the linker. In the molecular dynamics experiment, structure **28l** showed a decrease in total binding energy (−148.6 ± 19.5) compared to nilotinib and comparable values of polar (−290.5 ± 14.6) and non-polar components (−69.0 ± 20.3) in the complex with the human ABL kinase (PDB 3CS9). As seen in Figure 4b, analog **28l** binds to Abl in an imatinib/nilotinib-like (DFG-out) mode and makes similar interactions, including hydrogen bonds with the main chain of Met-318, side chain of Glu-286, and main chain amide of Asp-381. The two-dimensional interaction map of **28l** with Abl-wt (PDB 3CS9) reveals the presence of four hydrogen bonds (Met-318, Asp-381, Glu-286, and Tyr-253) and the non-covalent interaction between the pyridine fragment and the aromatic ring Tyr-253 (Figure 4d).

### 2.4. Structure–Activity Relationship Analysis

Molecular modeling showed that for compounds **11** and **13** or **9** and **15**, the placement of the trifluoromethyl and imidazole substitutes on the phenyl ring in the allosteric pocket is the most favorable. The aniline fragment of molecules containing strong electronegative groups, CF_3_ or NO_2_, is located in the adenine pocket. 

According to Table 2 and Table 3, the EGFR protein inhibitory activity of compounds **11** and **13** is positively correlated with the anti-tumor cell proliferation activity in contrast to compounds **9** and **15** containing the NO_2_ group. Consequently, we believe that the inhibitory activity against tumor cells of target compounds **11** and **13** containing (trifluoromethyl)aniline fragments was closely related to the affinity of these compounds to EGFR, thus inhibiting EGFR protein binding competitively. Its phosphorylation process is probably the main reason of the anti-tumor effect of these compounds.

The results also indicated that there was a moderate correlation between inhibition of receptor tyrosine kinases and the inhibition of cancer cellular proliferation. Comparing compounds **28i**–**28l**, the priority order of R_1_ substituents was established as follows: (3-fluorobenzoyl)piperazin > (4-nicotinoylpiperazin) > (2-fluorobenzoyl)piperazin >> (4-methoxybenzoyl)piperazin (Table 2). In addition, the results indicated that the chemical space position of the fluor atom could have slight influences on the inhibitory activity of compounds; the *meta*-position was better than the *ortho*-position.

The docking results revealed that introduction of the 4-(aminomethyl)benzamide as a flexible linker leads to a flattening of the structure, which allows the target molecules to be located in the allosteric pocket and penetrate into the adenine pocket, providing a more favorable location in the active center of the protein kinases. Perhaps this can explain the appearance of the high inhibitory activity of target compounds against some of protein kinase receptors. Taking into account the results obtained, further structural modifications of the studied compounds may be promising for discovering new inhibitors of protein kinases.

## 3. Experimental Section

### 3.1. General Information 

Organic solvents were purified and dried by standard methods before usage in the synthesis. The reactions were monitored and the purities of the compounds were checked by ascending thin-layer chromatography (TLC) on silica gel-coated aluminum plates (60 F254, 0.25 mm, Merck, Darmstadt, Germany). The following solvent systems were used: mixture of chloroform and methanol (10:1 or 4:1) or *n*-hexane–EtOAc (3:2 or 1:1); the spots were visualized under ultraviolet light at 254 nm. Preparative column chromatography was performed on silica gel Merck 60 (70–230 mesh). NMR spectra were registered on Bruker Avance 500 MHz spectrometer at 500 MHz (^1^H) and 125 MHz (^13^C). Chemical shifts (δ) are given in ppm related to internal SiMe_4_ and coupling constants (*J*) in Hz. The signals were designated as follows: s, singlet; d, doublet; t, triplet; q, quartet; m, multiplet. High-resolution mass spectra (HRMS) were recorded on an Agilent 1290 Accurate-Mass 6500 Series Q-TOF using ESI (electrospray ionization). The purity of the synthesized compounds was determined by high-performance liquid chromatography (HPLC) (Waters, 996, USA) with an EC 250/4,6 NUCLEODUR 100-5 C18ec column using KH_2_PO_4_(0.02 M, pH = 6.8) with formic acid/acetonitrile 45:55 mobile phase (1.0 mL/min). Melting points were determined on an electrically heated melting point (m.p.) apparatus and were uncorrected.

### 3.2. Chemistry

The synthesis of the starting compounds **2**–**4** according to the previously reported method is given below [20,21].

*4-(Chloromethyl)-N-(3-(4-methyl-1H-imidazol-1-yl)-5-(trifluoromethyl)phenyl)benzamide***(5).** To a solution of amine 3-(4-methyl-1H-imidazol-1-yl)-5-(trifluoromethyl)aniline **(a)** (one equivalent) and Et_3_N (1.5 equivalents) in dry CHCl_3_ cooled to 0 °C was added dropwise to a solution of 4-(chloromethyl)benzoyl chloride **(4)** (one equivalent) in dry CHCl_3_. The reaction mixture was stirred at room temperature. The progress was monitored by TLC. Cold water was added to the reaction mixture. The organic layer was separated, and the water layer was extracted by CHCl_3_ three times. The combined organic layers were dried over Na_2_SO_4_, filtered, and concentrated under vacuum. The product was purified by column chromatography on silica gel. The white solid **5** was given with 60% yield; m.p. 80–82 °C; ^1^H-NMR (DMSO-*d*_6_, 500 MHz) δ: 10.74 (s, 1H), 8.32 (s, 1H), 8.26 (d, *J* = 1.0 Hz, 1H), 8.17 (s, 1H), 8.03 (d, *J* = 8.3 Hz, 2H), 7.77 (s, 1H), 7.66 (d, *J* = 8.0 Hz, 2H), 7.52 (s, 1H), 4.89 (s, 2H), 2.21 (s, 3H); ^13^C-NMR (DMSO-*d*_6_, 125 MHz) δ: 166.1, 142.2, 141.7, 139.4, 138.4, 135.5, 134.4, 129.4, 128.6, 115.4, 114.7, 112.2, 45.8, 14.0; HRMS (ESI^+^) *m*/*z* calculated for C_19_H_16_ClF_3_N_3_O [M + H]^+^ 394.0929, found 394.0927; purity: 97.93% (by HPLC).

#### 3.2.1. General Methods for the Synthesis of **8**–**12**

A mixture of **5** (one equivalent), amine RNH_2_ (**a/b/c/e/g**) (1.1 equivalents) in *N,N*-Dimethylformamide (DMF) was stirred at reflux. The progress of reaction was monitored by TLC. DMF was removed under reduced pressure. The crude was diluted with water, and NH_3_∙H_2_O was added to obtain pH = 10–11. The resulting solid was filtered and washed with water. The product was purified by column chromatography on silica gel.

*N-(3-(4-methyl-1H-imidazol-1-yl)-5-(trifluoromethyl)phenyl)-4-(((3-(4-methyl-1H-imidazol-1-yl)-5-(trifluoromethyl)phenyl)amino)methyl)benzamide***(8)** was synthesized from compound **5** and 3-(4-methyl-1H-imidazol-1-yl)-5-(trifluoromethyl)aniline **(a)** as a white solid. M.p. 200–202 °C; ^1^H-NMR (DMSO-*d_6_*, 500 MHz) *δ*: 11.06 (s, 1H), 10.05 (d, *J* = 1.6 Hz, 1H), 8.47 (s, 1H), 8.28 (s, 1H), 8.23 (d, *J* = 1.3 Hz, 1H), 8.18 (d, *J* = 8.3 Hz, 2H), 8.15 (s, 1H), 7.76 (s, 1H), 7.64 (d, *J* = 8.3 Hz, 2H), 7.51 (s, 1H), 7.24 (s, 1H), 7.11 (s, 1H), 7.07 (s, 1H), 6.28 (s, 1H), 5.67 (s, 2H), 2.24 (d, *J* = 0.6 Hz, 2H), 2.20 (s, 3H); ^13^C-NMR (DMSO-*d_6_*, 125 MHz) *δ*: 165.9, 151.7, 141.8, 139.4, 138.3, 136.9, 136.6, 135.4, 134.5, 132.1, 129.1, 128.4, 119.3, 115.5, 114.6, 109.8, 105.1, 50.1, 14.0, 9.4; HRMS (ESI^+^) *m*/*z* calculated for C_30_H_25_F_6_N_6_O [M + H]^+^ 599.1989, found 599.1993.

*N-(3-(4-methyl-1H-imidazol-1-yl)-5-(trifluoromethyl)phenyl)-4-(((2-methyl-5-nitrophenyl)amino)methyl)benzamide***(9)** was synthesized from compound **5** and 2-methyl-5-nitroaniline **(b)** using the general method in Section 3.2.1 or from compound **7b** and 3-(4-methyl-1*H*-imidazol-1-yl)-5-(trifluoromethyl)aniline **(a)** using the general method in Section 3.2.4.2 as a yellow solid. M.p. 113–115 °C; ^1^H-NMR (DMSO-*d_6_*, 500 MHz) *δ*: 10.69 (s, 1H), 8.31 (s, 1H), 8.29 (d, *J* = 1.0 Hz, 1H), 8.16 (s, 1H), 7.98 (d, *J* = 8.0 Hz, 2H), 7.75 (s, 1H), 7.59 (d, *J* = 8.3 Hz, 2H), 7.52 (s, 1H), 7.38 (dd, *J* = 8.0, 2.2 Hz, 1H), 7.27 (d, *J* = 8.3 Hz, 1H), 7.09 (d, *J* = 2.2 Hz, 1H), 6.54 (t, *J* = 6.1 Hz, 1H), 4.58 (d, *J* = 6.1 Hz, 2H), 2.33 (s, 3H), 2.21 (s, 3H); ^13^C-NMR (DMSO-*d*_6_, 125 MHz) δ: 166.4, 147.4, 147.3, 144.5, 141.9, 139.0, 138.3, 135.4, 133.2, 131.0, 130.8, 128.5, 127.4, 115.4, 114.8, 111.1, 103.2, 46.3, 18.5, 13.8; HRMS (ESI^+^) *m*/*z* calculated for C_26_H_23_F_3_N_5_O_3_ [M + H]^+^ 510.1748, found 510.1747; purity: 96.63% (by HPLC).

*N-methyl-4-(4-((4-((3-(4-methyl-1H-imidazol-1-yl)-5-(trifluoromethyl)phenyl)carbamoyl)benzyl)amino)phenoxy)picolinamide***(10)** was synthesized from compound **5** and 4-(4-aminophenoxy)-*N*-methylpicolinamide **(c)** using the general method in Section 3.2.1 or from compound **7c** and 3-(4-methyl-1H-imidazol-1-yl)-5-(trifluoromethyl)aniline **(a)** using the general method in Section 3.2.4.1 as a white solid. M.p. 153–156 °C; ^1^H-NMR (DMSO-*d_6_*, 500 MHz) *δ*: 10.68 (s, 1H), 8.76 (q, *J* = 4.7 Hz, 1H), 8.47 (d, *J* = 5.4 Hz, 1H), 8.31 (s, 1H), 8.23 (d, *J* = 1.3 Hz, 1H), 8.17 (s, 1H), 8.00 (d, *J* = 8.3 Hz, 1H), 7.74 (s, 1H), 7.60 (d, *J* = 8.0 Hz, 1H), 7.50 (s, 1H), 7.33 (d, *J* = 2.6 Hz, 1H), 7.09 (dd, *J* = 5.8, 2.6 Hz, 1H), 6.95 (d, *J* = 8.7 Hz, 1H), 6.69 (d, *J* = 9.0 Hz, 1H), 6.59 (t, *J* = 5.9 Hz, 1H), 4.43 (d, *J* = 5.8 Hz, 1H), 2.79 (d, *J* = 4.8 Hz, 3H), 2.20(s, 2H); ^13^C-NMR (DMSO-*d*_6_, 125 MHz) δ: 167.2, 166.4, 164.4, 152.8, 150.7, 147.1, 145.3, 143.6, 141.9, 139.4, 138.4, 135.4, 133.1, 128.4, 127.7, 122.2, 115.3, 114.7, 114.3, 113.8, 112.0, 108.7, 46.9, 26.5, 14.0; HRMS (ESI^+^) *m*/*z* calculated for C_32_H_28_F_3_N_6_O_3_ [M + H]^+^ 601.2170, found 601.2167; purity: 99.27% (by HPLC).

*N-(3-(4-methyl-1H-imidazol-1-yl)-5-(trifluoromethyl)phenyl)-4-(((3-(trifluoromethyl)phenyl)amino)methyl)benzamide***(11)** was synthesized from compound **5** and 3-(trifluoromethyl)aniline **(g)** as a white solid. M.p. 83–85 °C; ^1^H-NMR (DMSO-*d_6_*, 500 MHz) δ: 10.67 (s, 1H), 8.31 (s, 1H), 8.16 (s, 1H), 7.98 (d, *J* = 8.3 Hz, 1H), 7.75 (s, 1H), 7.57 (d, *J* = 8.3 Hz, 1H), 7.27 (t, *J* = 7.9 Hz, 1H), 6.91 (t, *J* = 6.1 Hz, 1H), 6.88 (s, 1H), 6.84 (s, 1H), 6.83 (s, 1H), 4.46 (d, *J* = 6.1 Hz, 1H), 2.21 (s, 3H); ^13^C-NMR (DMSO-*d*_6_, 125 MHz) δ: 166.4, 149.4, 144.8, 141.8, 138.4, 133.1, 130.4, 128.4, 127.7, 116.1, 115.4, 114.8, 112.4, 108.7, 46.3, 13.9; HRMS (ESI*^+^) m/z* calculated for C_26_H_21_F_6_N_4_O [M + H]^+^ 519.1614, found 519.1613; purity: 97.02% (by HPLC).

*Methyl-4-amino-3-((4-((3-(4-methyl-1H-imidazol-1-yl)-5-(trifluoromethyl)phenyl)carbamoyl)benzyl)amino)benzoate***(12)** was synthesized from compound **5** and 3,4-diaminobenzoic acid **(e)** as a brown solid. M.p. 130–132 °C; ^1^H-NMR (DMSO-*d*_6_, 500 MHz) δ: 11.88 (brs, 1H), 10.68 (s, 1H), 8.34 (s, 1H), 8.31 (s, 1H), 8.25 (d, *J* = 1.3 Hz, 1H), 8.16 (s, 1H), 7.98 (d, *J* = 8.3 Hz, 2H), 7.75 (s, 1H), 7.57 (d, *J* = 8.3 Hz, 2H), 7.51 (s, 1H), 7.14 (dd, *J* = 8.2, 1.8 Hz, 1H), 6.90 (d, *J* = 1.9 Hz, 1H), 6.59 (d, *J* = 8.0 Hz, 1H), 5.47 (brs., 1H), 4.47 (d, *J* = 1.0 Hz, 2H), 2.20 (s, 3H), 2.11 (s, 1H); ^13^C-NMR (DMSO-*d*_6_, 125 MHz) δ: 168.4, 166.5, 145.3, 141.9, 141.2, 139.2, 138.4, 135.4, 134.3, 133.1, 128.3, 127.5, 121.1, 118.8, 115.4, 114.7, 112.8, 112.1, 111.5, 79.7, 47.0, 14.0; HRMS (ESI^+^) *m*/*z* calculated for C_26_H_23_F_3_N_5_O_3_ [M + H]^+^ 510.1748, found 510.1752.

#### 3.2.2. General Procedure for the Synthesis of **6a**–**d**

A mixture of methyl 4-formylbenzoate (**1**) (1.1 equivalents) and amine R_1_NH_2_ **(a**–**d)** (1.0 equivalent) was dissolved in dry CHCl_3_. Sodium triacetoxyborohydride (2.8 equivalents) and AcOH (six equivalents) were added to this solution, and the resulting solution was stirred at room temperature for 2–3 h. The reaction mixture was quenched with an ice-cold saturated solution of NaHCO_3_ (to pH 7), and extracted with CHCl_3_. The combined organic layers were dried over Na_2_SO_4_, filtered, and concentrated under vacuum. The residue was dissolved in ethyl acetate, and the product was precipitated with hexane. The solid was filtered and washed with ethyl acetate/hexane.

*Methyl 4-({[3-(4-methyl-1H-imidazol-1-yl)-5-(trifluoromethyl)phenyl]amino}methyl)benzoate***(6a)** was synthesized from methyl 4-formylbenzoate (**1**) and compound **a** as a white solid. The yield was 81%. ^1^H-NMR (DMSO-*d_6_*, 500 MHz) *δ*: 8.13 (d, *J* = 1.0 Hz, 1H), 7.96 (d, *J* = 8.3 Hz, 2H), 7.54 (d, *J* = 8.3 Hz, 2H), 7.42 (s, 1H), 7.14 (s, 1H), 7.04 (s, 1H), 6.96 (s, 1H), 6.86 (s, 1H), 4.54 (d, *J* = 6.1 Hz, 2H), 4.51–4.56 (m, 2H), 3.85 (s, 3H), 2.15 (s, 3H); ^13^C-NMR (DMSO-*d*_6_, 125 MHz) δ: 166.6, 150.7, 145.6, 139.1, 138.9, 135.3, 129.9, 127.9, 114.6, 107.6, 106.1, 103.8, 52.5, 46.2, 14.0; HRMS (ESI^+^) *m*/*z* calculated for C_20_H_19_F_3_N_3_O_2_ [M + H]^+^ 390,1424, found 390.1425.

*Methyl-4-{[(2-methyl-5-nitrophenyl) amino) methyl} benzoate***(6b)** was synthesized from **1** and 2-methyl-5-nitroaniline (**b**) as a yellow solid. The yield was 93%. ^1^H-NMR (DMSO-*d_6_*, 500 MHz) δ: 7.95 (d, *J* = 8.3 Hz, 2H), 7.53 (d, *J* = 8.3 Hz, 2H), 7.37 (dd, *J* = 8.0, 2.2 Hz, 1H), 7.26 (d, *J* = 8.0 Hz, 1H), 7.04 (d, *J* = 2.2 Hz, 1H), 6.50 (t, *J* = 6.1 Hz, 1H), 4.56 (d, *J* = 6.1 Hz, 2H), 3.84 (s, 3H), 2.31 (s, 3H); ^13^C NMR (DMSO-*d_6_*, 125 MHz) *δ*: 166.6, 147.4, 147.3, 145.8, 131.0, 130.8, 129.9, 128.8, 127.5, 111.1, 103.1, 67.5, 52.5, 46.4, 25.6, 18.5; HRMS (ESI^+^) *m*/*z* calculated for C_16_H_17_N_2_O_4_ [M + H]^+^ 301.1183, found 301.1185.

*Methyl-4-{[(4-{[2-(methylcarbamoyl)pyridin-4-yl]oxy}phenyl)amino]methyl}benzoate***(6c)** was synthesized from **1** and 4-(4-aminophenoxy)-*N*-methylpicolinamide **(c)** as a white solid. The yield was 88%. ^1^H-NMR (DMSO-*d*_6_, 500 MHz) *δ*: 8.73–8.79 (m, 1H), 8.44–8.49 (m, 1H), 7.93–7.98 (m, 2H), 7.53–7.58 (m, 2H), 7.31–7.35 (m, 1H), 7.06–7.10 (m, 1H), 6.90–6.96 (m, 2H), 6.64–6.68 (m, 2H), 6.52–6.57 (m, 1H), 4.38–4.43 (m, 2H), 3.86 (s, 3H), 2.79 (d, *J* = 4.8 Hz, 3H); ^13^C-NMR (DMSO-*d*_6_, 500 MHz) δ: 221.7, 221.5, 221.0, 218.0, 217.5, 216.6, 216.5, 215.8, 212.3, 212.0, 211.8, 210.4, 208.4, 208.2, 207.0, 192.8, 191.5, 186.3; HRMS (ESI^+^) *m*/*z* calculated for C_22_H_22_N_3_O_4_ [M + H]^+^ 392,1605, found 392.1602.

*Methyl 4-({[4-(pyridin-3-yl)pyrimidin-2-yl]amino}methyl)benzoate***(6d)** was synthesized from **1** and 4-(pyridin-3-yl)pyrimidin-2-amine (**d**) as a white solid. The yield was 81%. ^1^H-NMR (DMSO-*d*_6_, 500 MHz) δ: 9.23 (br.s., 1H), 8.70 (br. s., 1H), 8.43 (d, *J* = 5.1 Hz, 2H), 8.03 (t, *J* = 6.1 Hz, 1H), 7.93 (d, *J* = 8.3 Hz, 2H), 7.53 (br.s., 3H), 7.28 (d, *J* = 5.1 Hz, 1H), 4.67 (d, *J* = 5.8 Hz, 2H), 3.84 (s, 3H); ^13^C-NMR (DMSO-*d_6_*, 125 MHz) δ: 166.6, 162.9, 151.7, 148.5, 146.9, 134.7, 132.8, 129.7, 128.4, 127.7, 124.3, 52.5, 44.5; HRMS (ESI^+^) *m*/*z* calculated for C_18_H_17_N_4_O_2_ [M + H]^+^ 321.1346, found 321.1347.

#### 3.2.3. General Procedure for the Synthesis of **7a**–**d**

Aqueous 18–27% HCl (6 ml) was added to 2.5 mmol of ester **6a**–**d**, and the reaction mixture heated for 4–6 h at 80–90 °C. The reaction mixture was cooled to room temperature. The solid was filtered and washed with water. The product **7** was purified by precipitation from organic solvents.

*4-({[3-(4-Methyl-1H-imidazol-1-yl)-5-(trifluoromethyl)phenyl]amino}methyl)benzoic acid***(7a)** was synthesized from compound **6a** as a white solid. The yield was 82%. ^1^H-NMR (DMSO-*d*_6_, 500 MHz) δ: 8.90 (s, 1H), 7.94 (d, *J* = 8.3 Hz, 1H), 7.73 (s, 1H), 7.51 (d, *J* = 8.0 Hz, 1H), 7.29 (t, *J* = 5.9 Hz, 1H), 7.14 (s, 1H), 7.06 (s, 1H), 6.98 (s, 1H), 4.54 (d, *J* = 5.4 Hz, 1H), 2.26 (s, 3H); ^13^C-NMR (DMSO-*d*_6_, 125 MHz) *δ*: 167.6, 150.8, 144.8, 138.1, 134.8, 130.0, 127.8, 116.3, 108.9, 106.9, 104.6, 46.1, 12.0; HRMS (ESI^+^) *m*/*z* calculated for C_19_H_17_F_3_N_3_O_2_ [M + H]^+^ 376,1267, found 376.1271.

*4-{[(2-Methyl-5-nitrophenyl) amino)methyl}benzoic acid***(7b)** was synthesized from compound **6b** as a yellow solid. The yield was 83%. ^1^H-NMR (DMSO-*d_6_*, 500 MHz) *δ*: 7.92 (d, *J* = 8.3 Hz, 2H), 7.50 (d, *J* = 8.3 Hz, 2H), 7.37 (dd, *J* = 8.0, 2.2 Hz, 1H), 7.26 (d, *J* = 8.3 Hz, 1H), 7.05 (d, *J* = 2.6 Hz, 1H), 6.48 (t, *J* = 6.1 Hz, 1H), 4.55 (d, *J* = 6.1 Hz, 2H), 2.31 (s, 3H); ^13^C-NMR (DMSO-*d*_6_, 125 MHz) δ: 167.7, 147.4, 147.3, 145.2, 131.0, 130.8, 130.0, 129.9, 127.3, 111.1, 103.1, 46.4, 18.5; HRMS (ESI^+^) *m*/*z* calculated for C_15_H_15_N_2_O_4_ [M + H]^+^ 287.1026, found 287.1030.

*4-{[(4-{[2-(Methylcarbamoyl)pyridin-4-yl]oxy}phenyl)amino]methyl}benzoic acid***(7c)** was synthesized from compound **6c** as a white solid. The yield was 79%. ^1^H-NMR (DMSO-*d_6_*, 500 MHz) *δ*: 8.76 (q, *J* = 4.5 Hz, 1H), 8.46 (d, *J* = 5.8 Hz, 1H), 7.93 (d, *J* = 8.3 Hz, 2H), 7.51 (d, *J* = 8.3 Hz, 2H), 7.33 (d, *J* = 2.6 Hz, 1H), 7.07 (dd, *J* = 5.6, 2.7 Hz, 1H), 6.93 (d, *J* = 9.0 Hz, 2H), 6.66 (d, *J* = 8.7 Hz, 2H), 6.55 (brs., 1H), 4.38 (d, *J* = 4.8 Hz, 2H), 2.79 (d, *J* = 4.8 Hz, 3H); ^13^C-NMR (DMSO-*d*_6_, 125 MHz) δ: 221.8, 221.7, 221.0, 218.0, 217.5, 216.6, 216.3, 215.7, 212.3, 211.7, 210.4, 208.4, 208.2, 207.0, 191.5, 186.3; HRMS (ESI^+^) *m*/*z* calculated for C_21_H_20_N_3_O_4_ [M + H]^+^ 378,1448, found 378.1454.

*4-({[4-(Pyridin-3-yl)pyrimidin-2-yl]amino}methyl)benzoic acid***(7d)** was synthesized from compound **6d** as a white solid. The yield was 68%. ^1^H-NMR (DMSO-*d*_6_, 500 MHz) δ: 11.72–13.85 (m, 1H), 9.34 (brs., 1H), 8.83 (brs., 1H), 8.69 (d, *J* = 6.1 Hz, 1H), 8.48 (brs., 1H), 8.21 (brs., 1H), 7.91 (d, *J* = 8.0 Hz, 2H), 7.43–7.56 (m, 4H), 4.69 (brs., 2H); ^13^C-NMR (DMSO-*d*_6_, 125 MHz) δ: 167.7, 159.6, 148.9, 146.0, 145.8, 137.9, 133.9, 129.8, 129.6, 127.6, 125.6, 44.5; HRMS (ESI^+^) *m*/*z* calculated for C_17_H_15_N_4_O_2_ [M + H]^+^ 307.1190, found 307.1190.

#### 3.2.4. General Methods for the Synthesis of **9, 10,** and **13**–**25**

##### 3.2.4.1. Method viii

*Step 1 (synthesis of acyl chloride).* To a solution of acid **7a**–**d** in dry CHCl_3_ was added dropwise SOCl_2_ (four equivalents). The reaction mixture was stirred at 60 °C. The reaction progress was monitored by TLC. The excess thionyl chloride and solvent were removed under reduced pressure. The obtained acyl chlorides were immediately taken to the next step.

*Step 2 (synthesis of benzamide).* To a solution of chloroanhydride in dry CHCl_3_ was added amine R_2_NH_2_ (one equivalent) and Et_3_N (1.5 equivalents). The reaction mixture was stirred at room temperature. The reaction progress was monitored by TLC. Cold water was added to the reaction mixture. The organic layer was separated from the water. The combined organic layers were dried over Na_2_SO_4_, filtered, and concentrated under vacuum. The product was purified by column chromatography on silica gel.

##### 3.2.4.2. Method ix

A mixture of acid **7b** (or **7c**) (1.3 equivalents), 1-hydroxybenzotriazole (1.3 equivalents), and *N,N’*-dicyclohexylcarbodiimide or 1,1’-carbonyldiimidazole (1.3 equivalents) was dissolved in dry DMF. Et_3_N (2.5 equivalents) and amine R_2_NH_2_ (one equivalent) were added. The resulting mixture was stirred at room temperature. The reaction progress was monitored by TLC. The solvent was removed under reduced pressure. The crude was diluted with water, and NH_4_OH was added to obtain pH = 10–11. The resulting solid was filtered and washed with water. The product was purified by column chromatography on silica gel.

*4-({[3-(4-Methyl-1H-imidazol-1-yl)-5-(trifluoromethyl)phenyl]amino}methyl)-N-[3-(trifluoromethyl)phenyl]benzamide***(13)** was synthesized from compound **7a** and 3-(trifluoromethyl)aniline (**g**) using the general method in Section 3.2.4.1 as a white solid. M.p. 129–131 °C; ^1^H-NMR (DMSO-*d_6_*, 500 MHz) δ: 10.59 (s, 1H), 8.84 (brs., 1H), 8.28 (s, 1H), 8.08 (d, *J* = 8.3 Hz, 1H), 8.00 (d, *J* = 8.0 Hz, 2H), 7.72 (s, 1 H), 7.59–7.63 (m, 1H), 7.57 (d, *J* = 8.3 Hz, 2H), 7.46 (d, *J* = 7.7 Hz, 1H), 7.33 (t, *J* = 6.1 Hz, 1H), 7.14 (s, 1H), 7.08 (s, 1H), 7.00 (s, 1H), 4.52–4.60 (m, 2H), 2.25 (s, 3H); ^13^C-NMR (DMSO-*d_6_*, 125 MHz) *δ*: 166.2, 150.8, 143.8, 140.5, 138.2, 134.9, 133.6, 130.3, 128.5, 127.7, 124.2, 120.3, 116.8, 116.8, 116.1, 108.8, 106.9, 104.5, 60.2, 46.1, 14.6, 12.3; HRMS (ESI^+^) *m*/*z* calculated for C_26_H_21_F_6_N_4_O [M + H]^+^ 519.1614, found 519.1611; purity: 97.11% (by HPLC).

*Diethyl(4-(((3-(4-methyl-1H-imidazol-1-yl)-5-(trifluoromethyl)phenyl)amino)methyl)benzoyl)glutamate***(14)** was synthesized from compound **7a** and diethyl glutamate (**h**) using the general method in Section 3.2.4.1 as a white solid. M.p. 125–127 °C; ^1^H-NMR (DMSO-*d_6_*, 500 MHz) δ: 8.70 (d, *J* = 7.4 Hz, 1H), 8.15 (brs., 1H), 7.86 (d, *J* = 8.3 Hz, 2H), 7.51 (d, *J* = 8.0 Hz, 2H), 7.45 (brs., 1H), 7.12 (t, *J* = 5.9 Hz, 1H), 7.04 (s, 1H), 6.97 (s, 1H), 6.87 (s, 1H), 4.51 (d, *J* = 5.8 Hz, 2H), 4.44 (ddd, *J* = 9.4, 7.3, 5.4 Hz, 1H), 4.08–4.17 (m, 2H), 4.05 (q, *J* = 7.1 Hz, 2H), 2.45 (t, *J* = 7.7 Hz, 2H), 2.16 (s, 3H), 1.94–2.14 (m, 2H), 1.12–1.24 (m, 6H); ^13^C-NMR (DMSO-*d_6_*, 125 MHz) *δ*: 172.7, 172.3, 167.0, 150.7, 143.5, 139.0, 132.9, 128.2, 127.6, 107.6, 106.2, 103.8, 61.0, 60.4, 52.5, 46.2, 30.6, 26.2, 14.5, 14.1; HRMS (ESI^+^) *m*/*z* calculated for C_28_H_32_F_3_N_4_O_5_ [M + H]^+^ 561.2319, found 561.2321; purity: 97.13% (by HPLC).

*4-({[3-(4-Methyl-1H-imidazol-1-yl)-5-(trifluoromethyl)phenyl]amino}methyl)-N-{[(2-methyl-5-nitrophenyl) benzoate***(15)** was synthesized from compound **7a** and 2-methyl-5-nitroaniline (**b**) using the general method in Section 3.2.4.1 as a white solid. M.p. 205–208 °C; ^1^H-NMR (DMSO-*d*_6_, 500 MHz) δ: 10.11 (s, 1H), 8.37 (d, *J* = 2.2 Hz, 1H), 8.23 (s, 1H), 8.04 (dd, *J* = 8.5, 2.4 Hz, 1H), 7.99 (d, *J* = 8.3 Hz, 2H), 7.56–7.60 (m, 3H), 7.48 (s, 1H), 7.19 (t, *J* = 6.3 Hz, 1H), 7.06 (s, 1H), 6.99 (s, 1H), 6.91 (s, 1H), 4.56 (d, *J* = 6.1 Hz, 2H), 2.40 (s, 3H), 2.17 (s, 3H); ^13^C-NMR (DMSO-*d_6_*, 125 MHz) δ: 166.0, 150.7, 146.2, 144.0, 142.0, 138.9, 138.5, 137.9, 135.3, 133.2, 132.0, 128.6, 127.7, 120.8, 120.7, 114.8, 107.8, 106.2, 103.8, 46.1, 18.7, 13.9; HRMS (ESI^+^) *m*/*z* calculated for C_26_H_23_F_3_N_5_O_3_ [M + H]^+^ 510.1748, found 510.1745; purity: 99.06% (by HPLC).

*Diethyl (4-(((2-methyl-5-nitrophenyl)amino)methyl)benzoyl)glutamate***(16)** was synthesized from compound **7b** and diethyl glutamate (**h**) using the general method in Section 3.2.4.2 as a yellow solid. M.p. 127–129 °C; ^1^H-NMR (DMSO-*d*_6_, 500 MHz) δ: 8.70 (d, *J* = 7.7 Hz, 1H), 7.85 (d, *J* = 8.3 Hz, 2H), 7.50 (d, *J* = 8.3 Hz, 2H), 7.37 (dd, *J* = 8.0, 2.2 Hz, 1H), 7.26 (d, *J* = 8.3 Hz, 1H), 7.07 (d, *J* = 2.2 Hz, 1H), 6.49 (t, *J* = 6.1 Hz, 1H), 4.54 (d, *J* = 6.1 Hz, 2H), 4.44 (ddd, *J* = 9.5, 7.5, 5.4 Hz, 1H), 4.12 (qd, *J* = 7.1, 2.2 Hz, 2H), 4.05 (q, *J* = 7.1 Hz, 2H), 2.43–2.48 (m, 2H), 2.31 (s, 3H), 2.07–2.16 (m, 1H), 2.01 (dd, *J* = 9.6, 7.4 Hz, 1H), 1.18 (dt, *J* = 15.4, 7.2 Hz, 6H); ^13^C-NMR (DMSO-*d_6_*, 125 MHz) *δ*: 172.7, 172.3, 167.1, 147.4, 147.3, 143.6, 132.9, 130.9, 130.7, 128.2, 127.1, 111.0, 103.2, 79.7, 61.0, 60.4, 52.5, 46.3, 30.6, 26.2, 18.5, 14.5; HRMS (ESI^+^) *m/z* calculated for C_24_H_30_N_3_O_7_ [M + H]^+^ 472.2078, found 472.2080; purity: 97.32% (by HPLC).

*4-{[(2-Methyl-5-nitrophenyl)amino]methyl}-N-(4-{[2-(methylcarbamoyl)pyridin-4-yl]oxy}phenyl) benzoate***(17)** was synthesized from compound **7b** and 4-(4-aminophenoxy)-*N*-methylpicolinamide **(c)** using the general method in Section 3.2.4.2 as a yellow solid. M.p. 231–233 °C; ^1^H-NMR (DMSO-*d_6_*, 500 MHz) *δ*: 10.38 (brs, 1H), 8.80 (brs, 1H), 8.53 (brs, 1H), 7.93 (brs, 4H), 7.56 (brs, 2H), 7.41 (brs, 2H), 7.04–7.32 (m, 5H), 6.53 (brs, 1H), 4.58 (brs, 2H), 2.81 (brs, 3H), 2.33 (brs, 3H); ^13^C-NMR (DMSO-*d_6_*, 125 MHz) *δ*: 166.4, 165.9, 164.3, 153.1, 150.9, 149.1, 147.4, 147.3, 144.0, 137.7, 133.9, 131.0, 130.8, 128.4, 127.2, 122.6, 121.7, 114.3, 111.1, 109.2, 103.3, 46.2, 26.5, 18.5; HRMS (ESI^+^) *m/z* calculated for C_28_H_26_N_5_O_5_ [M + H]^+^ 512.1928, found 512.1926; purity: 95.23% (by HPLC).

*4-{[(2-Methyl-5-nitrophenyl)amino]methyl}-N-[3-(trifluoromethyl)phenyl]benzamide***(18)** was synthesized from compound **7b** and 3-(trifluoromethyl)aniline (**g**) using the general method in Section 3.2.4.2 as a yellow solid. M.p. 169–171 °C; ^1^H-NMR (DMSO-*d_6_*, 500 MHz) *δ*: 10.53 (s, 1H), 8.26 (brs, 1H), 8.04 (d, *J* = 8.0 Hz, 1H), 7.95 (d, *J* = 8.0 Hz, 2H), 7.60 (d, *J* = 8.0 Hz, 1H), 7.56 (d, *J* = 8.0 Hz, 2H), 7.46 (d, *J* = 7.4 Hz, 1H), 7.38 (d, *J* = 6.7 Hz, 1H), 7.27 (d, *J* = 8.3 Hz, 1H), 7.09 (s, 1H), 6.52 (brs, 1H), 4.57 (d, *J* = 5.8 Hz, 2H), 2.32 (s, 3H); ^13^C-NMR (DMSO-*d_6_*, 125 MHz): *δ* = 166.3, 147.4, 147.3, 144.2, 140.5, 133.6, 131.0, 130.8, 130.3, 128.5, 127.3, 124.2, 120.3, 116.8, 111.1, 103.2, 46.3, 18.5; HRMS (ESI^+^) *m/z* calculated for C_22_H_19_F_3_N_3_O_3_ [M + H]^+^ 430.1373, found 430.1374; purity: 98.18% (by HPLC).

*Methyl 4-amino-3-[(4-{[(2-methy-5-nitrophenyl)amino]methyl}benzoyl)amino]benzoate***(19)** was synthesized from compound **7b** and methyl 3,4-diaminobenzoate (**f**) using the general method in Section 3.2.4.2 as a yellow solid. M.p. 208–210 °C; ^1^H-NMR (DMSO-*d_6_*, 500 MHz) *δ*: 9.63 (s, 1H), 7.98 (d, *J* = 8.3 Hz, 2H), 7.79 (d, *J* = 1.6 Hz, 1H), 7.60 (dd, *J* = 8.7, 1.9 Hz, 1H), 7.53 (d, *J* = 8.0 Hz, 2H), 7.38 (dd, *J* = 8.0, 2.2 Hz, 1H), 7.27 (d, *J* = 8.0 Hz, 1H), 7.10 (d, *J* = 2.2 Hz, 1H), 6.79 (d, *J* = 8.7 Hz, 1H), 6.50–6.55 (m, 1H), 5.86 (s, 2H), 4.54–4.59 (m, 2H), 3.77 (s, 3H), 2.33 (s, 3H); ^13^C-NMR (DMSO-*d_6_*, 125 MHz) *δ*: 166.6, 166.1, 148.9, 147.4, 147.4, 143.6, 133.6, 131.0, 130.8, 129.4, 128.8, 128.6, 127.0, 122.3, 116.6, 115.0, 111.0, 103.2, 51.8, 46.3, 18.5; HRMS (ESI^+^) *m/z* calculated for C_23_H_23_N_4_O_5_ [M + H]^+^ 435.1663, found 435.1656; purity: 95.87% (by HPLC).

*4-{[(4-{[2-(Methylcarbamoyl)pyridin-4-yl]oxy}phenyl)amino]methyl}-N-[3-(trifluoromethyl)phenyl]benzamide***(20)** was synthesized from compound **7c** and 3-(trifluoromethyl)aniline (**g**) using the general method in Section 3.2.4.2 as a white solid. M.p. 170–171 °C; ^1^H-NMR (DMSO-*d_6_*, 500 MHz) *δ*: 10.54 (s, 1H), 8.77 (d, *J* = 4.8 Hz, 1H), 8.47 (d, *J* = 5.4 Hz, 1H), 8.28 (s, 1H), 8.07 (d, *J* = 8.3 Hz, 1H), 7.97 (d, *J* = 8.3 Hz, 2H), 7.60–7.67 (m, 1H), 7.58 (d, *J* = 8.0 Hz, 2H), 7.47 (d, *J* = 7.7 Hz, 1H), 7.33 (d, *J* = 2.6 Hz, 1H), 7.09 (dd, *J* = 5.8, 2.6 Hz, 1H), 6.95 (d, *J* = 8.7 Hz, 2H), 6.69 (d, *J* = 9.0 Hz, 2H), 6.58 (s, 1H), 4.42 (d, *J* = 5.4 Hz, 2H), 2.79 (d, *J* = 4.8 Hz, 3H); ^13^C-NMR (DMSO-*d_6_*, 125 MHz) *δ*: 167.2, 166.3, 164.4, 152.8, 150.7, 147.1, 145.0, 143.6, 140.5, 133.5, 130.3, 128.4, 127.6, 124.2, 122.2, 120.3, 116.8, 116.7, 114.0, 108.7, 79.7, 46.9, 26.5; HRMS (ESI^+^) *m/z* calculated for C_28_H_24_F_3_N_4_O_3_ [M + H]^+^ 521.1795, found 521.1800; purity: 98.85% (by HPLC).

*Diethyl (4-(((4-((2-(methylcarbamoyl)pyridin-4-yl)oxy)phenyl)amino)methyl)benzoyl)glutamate***(21)** was synthesized from compound **7c** and diethyl glutamate (**h**) using the general method in Section 3.2.4.2 as a white solid. M.p. 162–164 °C; ^1^H-NMR (DMSO-*d_6_*, 500 MHz) *δ*: 8.76 (q, *J* = 4.8 Hz, 1H), 8.70 (d, *J* = 7.4 Hz, 1H), 8.47 (d, *J* = 5.4 Hz, 1H), 7.87 (d, *J* = 8.3 Hz, 2H), 7.51 (d, *J* = 8.3 Hz, 2H), 7.33 (d, *J* = 2.6 Hz, 1H), 7.08 (dd, *J* = 5.4, 2.6 Hz, 1H), 6.91–6.95 (m, 2 H), 6.65–6.68 (m, 2 H), 6.53 (t, *J* = 5.9 Hz, 1 H), 4.45 (ddd, *J* = 9.6, 7.4, 5.1 Hz, 1H), 4.38 (d, *J* = 6.1 Hz, 2H), 4.12 (qd, *J* = 7.1, 2.4 Hz, 2H), 4.06 (q, *J* = 7.1 Hz, 2H), 2.79 (d, *J* = 4.8 Hz, 3H), 2.44–2.48 (m, 2H), 2.08 - 2.17 (m, 1H), 1.97–2.07 (m, 1H), 1.19 (dt, *J* = 13.1, 7.1 Hz, 6H); ^13^C-NMR (DMSO-*d_6_*, 125 MHz): *δ* = 172.7, 172.3, 167.2, 167.1, 164.4, 152.8, 150.7, 147.1, 144.4, 143.6, 132.7, 128.1, 127.4, 122.1, 114.2, 113.8, 108.8, 61.0, 60.4, 52.5, 47.0, 33.8, 30.7, 24.9, 14.6; HRMS (ESI^+^) *m/z* calculated for C_30_H_35_N_4_O_7_ [M + H]^+^ 563.2500, found 563.2502; purity: 98.00% (by HPLC).

*N-(3-(4-methyl-1H-imidazol-1-yl)-5-(trifluoromethyl)phenyl)-4-(((4-(pyridin-2-yl)pyrimidin-2-yl)amino)methyl)benzamide***(22)** was synthesized from compound **7d** and 3-(4-methyl-1*H*-imidazol-1-yl)-5-(trifluoromethyl)aniline (**a**) using the general method in Section 3.2.4.1. as a white solid. M.p. 150–152 °C; ^1^H-NMR (DMSO-*d_6_*, 500 MHz) *δ*: 10.70 (s, 1H), 9.26 (brs., 1H), 8.71 (brs., 1H), 8.44 (d, *J* = 4.8 Hz, 2H), 8.34 (brs., 1H), 8.31 (brs., 1H), 8.18 (s, 1H), 8.05 (t, *J* = 6.1 Hz, 1H), 7.99 (d, *J* = 8.3 Hz, 2H), 7.74 (s, 1H), 7.55 (d, *J* = 13.1 Hz, 4H), 7.29 (d, *J* = 5.1 Hz, 1H), 4.69 (d, *J* = 5.1 Hz, 2H), 2.21 (s, 3H); ^13^C-NMR (DMSO-*d_6_*, 125 MHz): *δ*: 166.4, 162.9, 151.8, 148.5, 141.9, 138.3, 134.7, 132.8, 128.3, 127.6, 124.3, 115.5, 114.8, 112.1, 49.1, 44.5, 13.8; HRMS (ESI^+^) *m/z* calculated for C_28_H_23_F_3_N_7_O [M + H]^+^ 530.1911, found 530.1913; purity: 96.67% (by HPLC).

*Diethyl (4-(((4-(pyridin-3-yl)pyrimidin-2-yl)amino)methyl)benzoyl)glutamate***(23)** was synthesized from compound **7d** and diethyl glutamate (**h**) using the general method in Section 3.2.4.1 as a white solid. M.p. 130–132 °C; ^1^H-NMR (DMSO-*d_6_*, 500 MHz) *δ*: 9.23–9.30 (m, 1H), 8.69–8.75 (m, 1H), 8.65–8.69 (m, 1H), 8.43 (d, *J* = 5.1 Hz, 2H), 7.98–8.06 (m, 1H), 7.84 (d, *J* = 8.3 Hz, 2H), 7.53–7.61 (m, 1H), 7.42–7.52 (m, 2H), 7.28 (d, *J* = 5.1 Hz, 1H), 4.61–4.69 (m, 2H), 4.40–4.46 (m, 1H), 4.01–4.19 (m, 4H), 2.44 (s, 2H), 1.95–2.19 (m, 2H), 1.12–1.25 (m, 6H); ^13^C-NMR (DMSO-*d_6_*, 125 MHz) *δ*: 172.7, 172.3, 167.1, 151.6, 148.3, 134.9, 132.9, 132.5, 127.9, 127.4, 124.4, 61.0, 60.4, 52.4, 44.4, 30.6, 26.2, 14.5; HRMS (ESI^+^) *m/z* calculated for C_26_H_30_N_5_O_5_ [M + H]^+^ 492.2241, found 492.2238; purity: 98.89% (by HPLC).

*4-({[4-(Pyridin-3-yl)pyrimidin-2-yl]amino}methyl)-N-[3-(trifluoromethyl)phenyl]benzamide***(24)** was synthesized from compound **7d** and 3-(trifluoromethyl)aniline (**g**) using the general method in Section 3.2.4.1. as a white solid. M.p. 138–141 °C; ^1^H-NMR (DMSO-*d_6_*, 500 MHz) *δ*: 10.51 (s, 1H), 9.29 (brs., 1H), 8.75 (br.s., 1H), 8.52 (d, *J* = 7.7 Hz, 1H), 8.46 (d, *J* = 4.8 Hz, 1H), 8.26 (s, 1H), 8.09 (brs., 1H), 8.05 (d, *J* = 8.3 Hz, 1H), 7.95 (d, *J* = 8.3 Hz, 2H), 7.52–7.66 (m, 4H), 7.46 (d, *J* = 7.7 Hz, 1H), 7.32 (d, *J* = 5.1 Hz, 1H), 4.69 (br.s., 2H); ^13^C-NMR (DMSO-*d_6_*, 125 MHz) *δ*: 166.3, 150.9, 147.7, 145.2, 140.5, 135.7, 133.2, 130.3, 128.3, 127.5, 124.7, 124.2, 120.3, 116.7; HRMS (ESI^+^) *m/z* calculated for C_24_H_19_F_3_N_5_O [M + H]^+^ 450.1536, found 450.1535; purity: 99.00% (by HPLC).

*4-({[4-(Pyridin-3-yl)pyrimidin-2-yl]amino}methyl)-N-{[(2-methyl-5-nitrophenyl) benzoate***(25)** was synthesized from compound **7d** and 2-methyl-5-nitroaniline (**b**) using the general method in Section 3.2.4.1 as a white solid. M.p. 175–176 °C; ^1^H NMR (DMSO-*d_6_*, 500 MHz) *δ*: 10.08 (s, 1H), 9.27 (brs., 1H), 8.71 (brs., 1H), 8.44 (d, *J* = 4.5 Hz, 2H), 8.36 (d, *J* = 1.9 Hz, 1H), 8.02–8.08 (m, 2H), 7.97 (d, *J* = 8.0 Hz, 2H), 7.58 (d, *J* = 8.3 Hz, 4H), 7.29 (d, *J* = 5.1 Hz, 1H), 4.69 (d, *J* = 4.2 Hz, 2H), 2.39 (s, 3H); ^13^C NMR (DMSO-*d_6_*, 125 MHz): *δ*: 166.1, 151.7, 148.4, 146.2, 141.9, 137.9, 134.8, 132.9, 132.8, 132.0, 128.3, 127.6, 124.4, 120.8, 120.7, 44.5, 18.7; HRMS (ESI^+^) *m/z* calculated for C_24_H_21_N_6_O_3_ [M + H]^+^ 441.1670, found 441.1670; purity: 99.42% (by HPLC).

#### 3.2.5. Compounds **26i**–**l** Prepared According to Section 3.2.2 Described for **6a**–**d**

*Methyl 4-((4-(4-methoxybenzoyl)piperazin-1-yl)methyl)benzoate* (**26i**) was synthesized from (4-methoxyphenyl)(piperazin-1-yl)methanone (**i**) and methyl 4-formylbenzoate (**1**) as a white solid. The yield was 75%. ^1^H-NMR (DMSO-*d_6_*, 500 MHz) *δ*: 7.94 (d, *J* = 8.3 Hz, 2H), 7.49 (d, *J* = 8.3 Hz, 2H), 7.36 (d, *J* = 9.0 Hz, 2H), 6.99 (d, *J* = 8.7 Hz, 2H), 3.84–3.89 (m, 3H), 3.80 (s, 3H), 3.60 (s, 2H), 3.43–3.58 (m, 4H), 2.32–2.46 (m, 4H); ^13^C-NMR (DMSO-*d_6_*, 125 MHz) *δ*: 169.4, 166.6, 160.6, 144.2, 129.7, 129.5, 128.9, 128.2, 114.1, 79.6, 61.8, 55.7, 53.1, 52.6.

*Methyl 4-((4-(2-fluorobenzoyl)piperazin-1-yl)methyl)benzoate* (**26j**) was synthesized from (2-fluorophenyl)(piperazin-1-yl)methanone (**j**) and compound **1** as a white solid. The yield was 22%. ^1^H-NMR (DMSO-*d_6_*, 500 MHz) *δ*: 7.94 (d, *J* = 8.3 Hz, 2H), 7.48 (d, *J* = 8.3 Hz, 3H), 7.37–7.42 (m, 1H), 7.27–7.33 (m, 2H), 3.86 (s, 3H), 3.63–3.73 (m, 2H), 3.60 (s, 2H), 3.19–3.28 (m, 2H), 2.40–2.50 (m, 2H), 2.30–2.39 (m, 2H); ^13^C-NMR (DMSO-*d_6_*, 125 MHz) *δ*: 166.6, 164.4, 159.0, 157.0, 144.1, 131.9, 129.6, 129.5, 129.2, 128.9, 125.4, 116.3, 61.7, 53.2, 52.7, 52.6, 47.0, 41.8.

*Methyl 4-((4-(3-fluorobenzoyl)piperazin-1-yl)methyl)benzoate* (**26k**) was synthesized from (3-fluorophenyl)(piperazin-1-yl)methanone (**k**) and compound **1** as a white solid. The yield was 19%. ^1^H-NMR (DMSO-*d_6_*, 500 MHz) *δ*: 7.94 (d, *J* = 8.3 Hz, 2H), 7.46–7.53 (m, 3H), 7.28–7.33 (m, 1H), 7.21–7.26 (m, 2H), 3.86 (s, 3H), 3.64 (brs, 2H), 3.60 (s, 2H), 3.33 (brs, 2H), 2.45 (brs, 2H), 2.37 (brs, 2H); ^13^C-NMR (DMSO-*d_6_*, 125 MHz) *δ*: 167.9, 166.6, 163.3, 161.3, 144.2, 131.2, 129.7, 129.5, 128.9, 123.4, 116.9, 114.4, 61.7, 53.2, 52.5, 47.5, 42.0.

*Methyl 4-((4-nicotinoylpiperazin-1-yl)methyl)benzoate* (**26l**) was synthesized from piperazin-1-yl(pyridin-3-yl)methanone (**l**) and compound **1** as a white solid. The yield was 83%. ^1^H-NMR (DMSO-*d_6_*, 500 MHz) *δ*: 8.65 (dd, *J* = 4.8, 1.6 Hz, 1H), 8.61 (d, *J* = 1.3 Hz, 1H), 7.94 (d, *J* = 8.3 Hz, 2H), 7.84 (dt, *J* = 7.7, 1.9 Hz, 1H), 7.47–7.51 (m, 3H), 3.86 (s, 3H), 3.67 (brs, 2H), 3.61 (s, 2H), 3.36 (brs, 2H), 2.47 (brs, 2H), 2.39 (brs, 2H); ^13^C-NMR (DMSO-*d_6_*, 125 MHz) *δ*: 167.2, 166.6, 150.9, 148.0, 144.2, 135.3, 132.2, 129.7, 129.5, 128.9, 124.0, 61.7, 53.2, 52.6, 47.7, 42.1.

#### 3.2.6. Compounds **27i**–**l** Prepared According to Section 3.2.3 Described for **7a**–**d**

*4-((4-(4-Methoxybenzoyl)piperazin-1-yl)methyl)benzoic acid* (27i) was synthesized from compound **26i** as a white solid. The yield was 60%. ^1^H-NMR (DMSO-*d_6_*, 500 MHz) *δ*: 7.92 (d, *J* = 8.3 Hz, 2H), 7.46 (d, *J* = 8.3 Hz, 2H), 7.34–7.39 (m, 2H), 6.96 - 7.02 (m, 2H), 3.80 (s, 3H), 3.53–3.70 (m, 6H), 2.41 (brs, 4H); ^13^C-NMR (DMSO-*d_6_*, 125 MHz) *δ*: 169.4, 167.7, 160.6, 143.6, 130.1, 129.8, 129.5, 129.4, 128.2, 114.1, 79.6, 61.9, 55.7, 53.1.

*4-((4-(2-Fluorobenzoyl)piperazin-1-yl)methyl)benzoic acid* (**27j**) was synthesized from compound **26j** as a white solid. The yield was 60%. ^1^H-NMR (DMSO-*d_6_*, 500 MHz) δ: 7.93 (s, 2H), 7.48–7.54 (m, 1H), 7.45 (d, *J* = 8.3 Hz, 2H), 7.40 (td, *J* = 7.3, 1.8 Hz, 1H), 7.27–7.33 (m, 2H), 3.67 (brs, 2H), 3.58–3.61 (m, 2H), 3.22–3.26 (m, 2H), 2.45 (brs, 2H), 2.36 (d, *J* = 9.0 Hz, 2H); ^13^C-NMR (DMSO-*d_6_*, 125 MHz) *δ*: 167.7, 164.4, 159.0, 157.0, 143.5, 131.9, 130.1, 129.8, 129.4, 129.2, 125.4, 116.3, 61.8, 53.2, 52.7, 47.0, 41.8.

*4-((4-(3-Fluorobenzoyl)piperazin-1-yl)methyl)benzoic acid* (**27k**) was synthesized from compound **26k** as a white solid. The yield was 53%. ^1^H-NMR (DMSO-*d_6_*, 500 MHz) *δ*: 7.92 (d, *J* = 8.3 Hz, 2H), 7.50 (td, *J* = 7.9, 5.9 Hz, 1H), 7.45 (d, *J* = 8.0 Hz, 2H), 7.27–7.34 (m, 1H), 7.21–7.26 (m, 2H), 3.64 (brs, 2H), 3.59 (s, 2H), 3.33 (brs, 2H), 2.45 (brs, 2H), 2.38 (brs, 2H); ^13^C-NMR (DMSO-*d_6_*, 125 MHz) *δ*: 167.9, 167.7, 163.3, 161.3, 143.6, 131.2, 130.2, 129.8, 129.4, 123.4, 116.9, 114.4, 61.8, 53.1, 52.6, 47.5, 42.1.

*4-((4-Nicotinoylpiperazin-1-yl)methyl)benzoic acid* (**27l**) was synthesized from methyl 4-((4-nicotinoylpiperazin-1-yl)methyl)benzoate **26l** as a white solid. The yield was 72%. ^1^H-NMR (DMSO-*d_6_*, 500 MHz) *δ*: 8.65–8.67 (m, 1H), 8.61 (d, *J* = 1.6 Hz, 1H), 7.92 (d, *J* = 8.3 Hz, 2H), 7.84 (dt, *J* = 7.7, 1.9 Hz, 1H), 7.47–7.50 (m, 1H), 7.46 (d, *J* = 8.3 Hz, 2H), 3.67 (brs, 2H), 3.60 (s, 2H), 3.36 (brs, 2H), 2.47 (brs, 2H), 2.39 (brs, 2H); ^13^C-NMR (DMSO-*d_6_*, 125 MHz) *δ*: 167.7, 167.2, 150.9, 148.0, 143.6, 135.3, 132.2, 130.1, 129.8, 129.4, 124.0, 61.8, 53.2, 52.6, 47.7, 42.1.

#### 3.2.7. Compounds **28i**–**28l** Prepared According to Method **viii** (Section 3.2.4.1)

*4-{[4-(4-Methoxybenzoyl)piperazin-1-yl]methyl}-N-[3-(4-methyl-1H-imidazol-1-yl)-5-(trifluoromethyl)phenyl]benzamide***(28i)** was synthesized from compound **27i** and amine **a** as a white solid. M.p. 98–102 °C; ^1^H-NMR (DMSO-*d_6_*, 500 MHz) *δ*: 10.69 (s, 1H), 8.30 (s, 1H), 8.22 (d, *J* = 1.0 Hz, 1H), 8.18 (s, 1H), 7.98 (d, *J* = 8.3 Hz, 2H), 7.75 (s, 1H), 7.53 (d, *J* = 8.0 Hz, 2H), 7.50 (s, 1H), 7.34–7.40 (m, *J* = 8.7 Hz, 2H), 6.97–7.02 (m, *J* = 9.0 Hz, 2H), 3.80 (s, 3H), 3.62 (s, 2H), 3.45–3.60 (m, 4H), 2.37 - 2.47 (m, 4H), 2.20 (s, 3H); ^13^C-NMR (DMSO-*d_6_*, 125 MHz) *δ*: 169.4, 166.4, 160.7, 142.9, 141.8, 139.4, 138.4, 135.5, 133.3, 129.5, 129.4, 128.3, 115.4, 114.7, 114.1, 61.8, 55.7, 14.0; HRMS (ESI^+^) *m/z* calculated for C_31_H_31_F_3_N_5_O_3_ [M + H]^+^ 578.2374, found 578.2362; purity: 95.81% (by HPLC).

*4-{[4-(2-Fluorobenzoyl)piperazin-1-yl]methyl}-N-[3-(4-methyl-1H-imidazol-1-yl)-5-(trifluoromethyl)phenyl]benzamide* (**28j**) was synthesized from compound **27j** and amine **a** as a white solid. M.p. 106–109 °C; ^1^H-NMR (DMSO-*d_6_*, 500 MHz) *δ*: 10.68 (s, 1H), 8.31 (s, 1H), 8.22 (d, *J* = 1.0 Hz, 1H), 8.17 (s, 1H), 7.99 (d, J = 8.3 Hz, 2H), 7.75 (s, 1H), 7.49–7.55 (m, 4H), 7.38–7.43 (m, 1H), 7.28–7.34 (m, 2H), 3.69 (brs, 2H), 3.63 (s, 2H), 3.26 (t, *J* = 4.3 Hz, 2H), 2.48 (brs, 2H), 2.39 (d, *J* = 4.5 Hz, 2H), 2.20 (s, 3H); ^13^C-NMR (DMSO-*d_6_*, 125 MHz) *δ*: 166.4, 164.4, 159.1, 157.0, 142.9, 141.8, 139.4, 138.4, 135.5, 133.4, 132.0, 129.4, 128.3, 125.4, 116.4, 116.2, 115.4, 114.7, 112.1, 79.7, 61.7, 53.3, 52.7, 47.1, 41.8, 14.1; HRMS (ESI^+^) *m/z* calculated for C_30_H_28_F_4_N_5_O_2_ [M + H]^+^ 566.2174, found 566.2176; purity: 95.05% (by HPLC).

*4-{[4-(3-Fluorobenzoyl)piperazin-1-yl]methyl}-N-[3-(4-methyl-1H-imidazol-1-yl)-5-(trifluoromethyl)phenyl]benzamide* (**28k**) was synthesized from compound **27k** and amine **a** as a light yellow solid. M.p. 90–93 °C; ^1^H-NMR (DMSO-*d_6_*, 500 MHz) *δ*: 10.69 (s, 1H), 8.31 (s, 1H), 8.22 (d, *J* = 1.3 Hz, 1H), 8.18 (s, 1H), 7.99 (d, *J* = 8.3 Hz, 2H), 7.75 (s, 1H), 7.48–7.55 (m, 4H), 7.29–7.34 (m, 1H), 7.22–7.28 (m, 2H), 3.61–3.71 (m, 4H), 3.33–3.38 (m, 2H), 2.35–2.50 (m, 4H), 2.20 (s, 3H); ^13^C-NMR (DMSO-*d_6_*, 125 MHz) *δ*: 167.9, 166.4, 163.3, 161.3, 142.9, 141.8, 139.4, 138.4, 135.5, 133.4, 131.2, 129.4, 128.3, 123.4, 116.9, 116.8, 115.3, 114.7, 112.1, 79.7, 61.8, 53.2, 52.6, 47.6, 42.0, 14.1; HRMS (ESI^+^) *m/z* calculated for C_30_H_28_F_4_N_5_O_2_ [M + H]^+^ 566,2174, found 566.2176; purity: 94.86% (by HPLC).

*4-{[4-(Pyridin-3-yl-carbonyl)piperazin-1-yl]methyl}}-N-[3-(4-methyl-1H-imidazol-1-yl)-5-(trifluoromethyl)phenyl]benzamide* (**28l**) was synthesized from compound **27l** and amine **a** as a white solid. M.p. 96–100 °C; ^1^H-NMR (DMSO-*d_6_*, 500 MHz) *δ*: 10.68 (s, 1H), 8.67 (dd, *J* = 4.8, 1.6 Hz, 1H), 8.62 (d, *J* = 1.9 Hz, 1H), 8.31 (s, 1H), 8.22 (d, *J* = 1.3 Hz, 1H), 8.17 (s, 1H), 7.99 (d, *J* = 8.3 Hz, 2H), 7.85 (dt, *J* = 7.8, 1.9 Hz, 1H), 7.75 (s, 1H), 7.54 (d, *J* = 8.3 Hz, 2H), 7.49–7.52 (m, 2H), 3.66–3.72 (m, 2H), 3.64 (s, 2H), 3.37–3.42 (m, 2H), 2.50 (brs, 2H), 2.42 (brs, 2H), 2.20 (s, 3H); ^13^C-NMR (DMSO-*d_6_*, 125 MHz) *δ*: 167.2, 166.4, 150.9, 148.1, 142.9, 141.8, 139.4, 138.4, 135.5, 135.3, 133.4, 132.2, 129.4, 128.3, 124.0, 115.4, 114.7, 112.1, 61.7, 53.2, 52.6, 49.1, 42.1, 14.0; HRMS (ESI^+^) *m/z* calculated for C_29_H_28_F_3_N_6_O_2_ [M + H]^+^ 549.2221, found 549.2242; purity: 98.12% (by HPLC).

### 3.3. Cytotoxicity Assay

Each in vitro experiment was performed at least in triplicate, and the standard deviation of absorbance was less than 10% of the mean. For the in vitro assays, a stock solution (1% DMSO in the appropriate buffer with the tested compound diluted under sonication) was prepared from which several dilutions were made with the appropriate buffer.

Human cell lines were obtained from the Institute of Cytology, Russian Academy of Sciences. The cell lines studied were maintained in RPMI 1640 medium (AppliChem, Darmstadt, Germany) supplemented with 20% (HL-60, RPMI 1788) fetal calf serum (HyClone, Cramlington, UK), in DMEM (A 549) and Eagle’s MEM (AppliChem) (MCF7, HeLa) with 10% fetal calf serum (HyClone). Cells were cultivated at 37 °C in a humidified atmosphere of 5% CO_2_. Cellular sensitivities to clofarabine and its lipid derivatives were measured with the MTT assay. Cells were plated in triplicate in 96-well plates (10^5^ cells/well for suspended cell cultures and 5 × 10^3^ cells/well for monolayer cultures); on the same day (suspended cell cultures) or on the following day (monolayer cultures), compounds were added at the appropriate dilutions. Plates were incubated under standard conditions for 48 h. Thereafter, 10 μL of MTT (Sigma) in phosphate-buffered saline (5 mg/mL) was added. The plates were incubated for an additional 4 h, and 150 μL of dimethyl sulfoxide (DMSO) was added to dissolve the formazan crystals. The optical density was read on a HALO MPR-95 Microplate Reader (Dynamica Scientific Ltd., Australia) at 570 nm. The antiproliferative effects were determined as degrees of inhibition of tumor cells (%).

### 3.4. Kinase Inhibitory Assays

The assay was performed using a Kinase Selectivity Profiling System (TK-1+ADP-Glo™ Assay and TK-2+ADP-Glo™ Assay, Promega), where the kinase activity was measured by quantifying the amount of ADP produced during a kinase reaction. The assay is performed in two steps; firstly, after the kinase reaction, an equal volume of ADP-Glo™ Reagent is added to terminate the kinase reaction and deplete the remaining ATP. Secondly, the Kinase Detection Reagent is added to simultaneously convert ADP to ATP and allow the newly synthesized ATP to be measured using a luciferase/luciferin reaction. The luminescent signal generated is proportional to the ADP concentration produced and is correlated with kinase activity. Our compounds were diluted with kinase buffer (5% DMSO), and 1 μL of the dilution was added to a 5-μL reaction so that the final concentration of DMSO was 1% in all of reactions. All of the enzymatic reactions were conducted at 23 °C for 60 min. The 5-μL reaction mixture contained 1µL of the compound solution, 2µL of the enzyme solution, and 2 µL of kinase substrate. After the enzymatic reaction, 5 µL of ADP-Glo™ Reagent (Promega, USA) was added to each reaction, and the plate was incubated for 40 min at 23 °C. Then, 10 µL of Kinase Detection Reagent (Promega, USA) was added to each reaction, and the plate was incubated for 30 min at 23 °C. Luminescence signal was measured using an Infinite M 200 plate reader (Tecan, Switzerland).

Kinase activity assays were performed in duplicate at each concentration. The luminescence data were analyzed using the computer software, Magellan™. The difference between luminescence intensities in the absence of Kinase (Lut) and in the presence of Kinase (Luc) was defined as 100% activity (Lut − Luc). Using luminescence signal (Lu) in the presence of the compound, percentage inhibition of kinase was calculated as follows:

% inhibition = [1 − (Lut − Lu)(Lut − Luc)] × 100%

where Lu is the luminescence intensity in the presence of the compound.

### 3.5. Docking

AutoDock Vina was used for docking studies [24]. Ligands from PDB complexes were extracted by copying the HEATATM section of the PDB file. Two-dimensional structures were created using Marvin Sketch [31]. Three-dimensional (3D) structures were generated using molconvert [32]. Conformation generation and structure minimization were performed with Open Babel [33]. Missing protein residues were restored with MODELLER [34]. GROMACS with the AMBER FF99SB-ILDN force field was used for obtaining minimized receptors. Binding site coordinates of receptors were determined by the size of the ligand in the PDB file and were scaled up by 20% in each dimension. Docking results were limited by only one docking pose having the best docking score. Preparation of ligands and receptors for docking was carried out in MGL Tools [35]. Visualization of docking results and the H-bond search were done in Chimera [36]. Two-dimensional interaction maps were generated by PoseView [37].

### 3.6. Molecular Dynamics

Molecular dynamics was carried out using the GROMACS package with the AMBER FF99SB-ILDN force field. All ligands were parametrized with ACPYPE [38]. The simulation workflow was as follows: solvation, neutralizing by adding NaCl ions up to a concentration of 0.15 mol, energy minimization, NPT and NVT equilibration steps (200 ps each), and a final 2-ns production run at 300 K. A dodecahedron box of 1.2 nm and periodic bounding conditions were used. The Berendsen thermostat was used for equilibration. Long electrostatic coupling was treated according to the PME method. The g_mmpbsa tool was used for MM-PBSA binding energy calculations [26]. To perform energy calculations, every 20th frame of the final molecular dynamics trajectory was extracted, skipping the first 100 frames.

Experimental data of compounds, biological assays and computational methods are available in Supplementary Materials.

## 4. Conclusions

In summary, a series of novel compounds, containing as a linker the 4-(aminomethyl)benzamide, were successfully prepared and examined for their anti-tumor activities. The tertiary heterocyclic amines **28j**, **28k**, **28l**, and analog **13** demonstrated significant activity against K562 cells (IC_50_ of 6.9 µM, 3.6 µM, 4.5 µM, and 5.6 µM, respectively). The biological evaluation indicated that most of compounds displayed multitarget profile and high inhibitory activity toward EGFR, KDR, PDGFRa, HER-4, and InsR kinases.

The docking results showed that the studied compounds exhibited a similar binding mode to type II inhibitors of tyrosine kinase. We established for analog **10** that the use of the 4-(aminomethyl)benzamide as a flexible linker leads to a favorable overall geometry of the molecule, allowing one to bypass the bulk isoleucine residue, and providing the necessary binding to the active center of the T315I-mutant Abl. The results gleaned from this investigation have important potential applications in the development of new anticancer drugs overcoming the clinical acquired resistance.

## Figures and Tables

**Table 1:** Synthesis methods and structures of obtained compounds **8**–**25**.

№	Structure	Method Yield (%)	№	Structure	Method Yield (%)
**8**	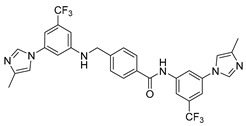	v 15	**17**	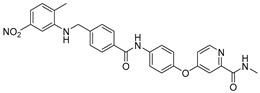	ix 58
**9**	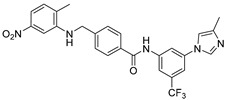	v (ix) 10 (21)	**18**	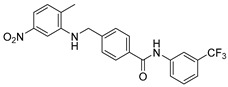	ix 38
**10**	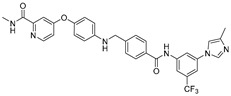	v (viii) 19 (20)	**19**	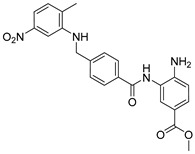	ix 34
**11**	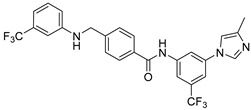	v 17	**20**	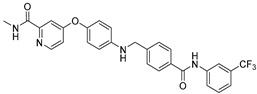	ix 80
**12**	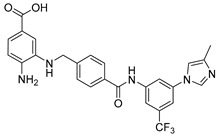	v 25	**21**	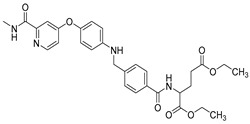	ix 82
**13**	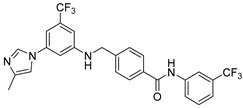	viii 25	**22**	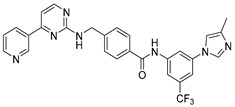	viii 50
**14**	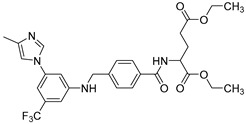	viii 40	**23**	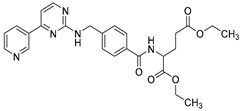	viii 31
**15**	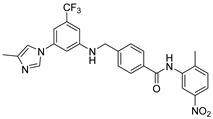	viii 30	**24**	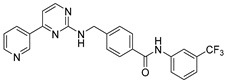	viii 37
**16**	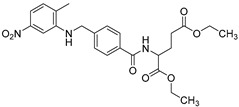	ix 76	**25**	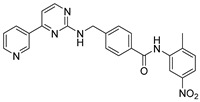	viii 27

**Table 2:** *In vitro* anti-proliferative activities of targets compounds against cancer cells (K-562, HL-60, MCF-7, Hela, A-549, OKP-GS) and human normal cells (RPMI 1788).

Comp.	Log P ^b^ (TPSA)		Anti-Proliferative Activities in Different Cancer Cell Lines (IC_50_ (μM)^a^						Inhibition (%) 100 μM
			K-562	HL-60	MCF-7	Hela	A-549	OKP-GS	RPMI 1788
**5**	4.20 (46.92)		69	45	54	51.8	53.5	70	<0.1
**8**	5.68 (76.78)		n/a	n/a	9.5	87	85	>100	19.32
**9**	4.30 (97.63)		51	57	>100	n/a	>100	>100	n/a
**10**	5.00 (110.17)		40	8.2	>100	>100	>100	81	10.42
**11**	5.75 (58.95)		53	51	57	54	61	54.3	15.53
**13**	5.75 (58.95)		5.6	46	69	57.5	55	52	9.36
**14**	0.75 (133.55)		50	45	n/a	n/a	n/a	n/a	10.45
**15**	5.22 (104.78)		31	5.8	>100	78.5	73	>100	15.93
**19**	3.74 (150.27)		64	>100	n/a	n/a	n/a	n/a	10
**23**	−0.63 (154.40)		66	49	n/a	n/a	n/a	n/a	11.52
**28i**	3.94 (79.70)		30.7	38	63	11.5	>100	78	6.32
**28j**	4.00 (70.47)		6.9	30.6	71	n/a	>100	n/a	6.44
**28k**	4.02 (70.47)		3.6	38.5	n/a	n/a	n/a	n/a	3.14
**28l**	2.65 (83.36)		4.5	53.5	77	>100	>100	>100	n/a
**Reference Compounds**									
**Imatinib**		3.89 (86.28)	0.41	44.5	49	45.8	54	48	13.37
**Nilotinib**		4.99 (97.63)	<0.1	55	n/a	n/a	n/a	55	<0.1
**Sorafenib**		3.76 (92.35)	n/a	n/a	55	n/a	58	51	n/a

^a^ IC_50_, the concentration that causes 50% growth inhibition; ^b^ logP_o/w_ values were calculated based on Molinspiration program predictions (http://www.molinspiration.com/cdi-bin/properties); TPSA—topological polar surface area; n/a—not applicable.

**Table 3:** Inhibition of receptor tyrosine kinases by compounds **5**, **10**, **11**, **13**–**15**, **17**, **18**, **20**, **22**–**24**, **28i**–**28l**, and imatinib.

Compound	% Inhibition at 10 nM							
	EGFR	HER-2	HER-4	IGF1R	InsR	KDR	PDGFRa	PDGFRb
**5**	0	0	0	0	57	0	0	0
**10**	65	0	51	0	46	27	66	0
**11**	91	0	49	0	0	16	55	0
**13**	92	34	54	0	0	20	59	8
14	0	0	0	0	23	0	0	0
**15**	0	0	0	0	43	0	0	0
**17**	0	0	0	0	49	0	0	0
**18**	0	14	56	0	0	24	67	13
**20**	56	0	57	18	0	48	77	28
**22**	21	0	49	0	0	28	0	0
**23**	0	0	12	1	49	0	0	0
**24**	24	0	44	0	0	2	0	0
**28i**	0	0	0	3	52	0	0	0
**28j**	0	0	0	0	50	0	8	2
**28k**	0	0	43	0	33	23	0	2
**28l**	0	0	51	0	0	17	0	7
**Imatinib**	0	0	42	0	36	0	56	75

**Table 4:** AutoDock Vina’s docking scores for targets (**8**–**25**, **28i**–**28l)** and reference compounds.

Ligand	Protein Data Bank (PDB) Code Protein Kinases								
	2HYY	2PL0	3GCS	3WZE	3CS9	3QRJ *	4AG8	4ASD	5HI2
**8**	−10.7	−7.9	−8.4	−4.2	−10.8	−9.1	−3.0	−2.4	−5.9
**9**	−11.4	−7.8	−9.8	−8.8	−10.6	−11.0	−7.2	−8.7	−7.8
**10**	−8.9	−8.8	−7.8	−6.7	−8.5	−12.3	−6.3	−6.7	−3.9
**11**	−11.6	−7.0	−10.5	−8.1	−11.9	−12.2	−8.6	−8.6	−8.3
**12**	−7.7	−7.2	−8.9	−4.4	−7.9	−9.7	−6.4	−5.2	−8.5
**13**	−11.4	−9.5	−10.2	−9.4	−11.7	−11.5	−9.9	−8.6	−10.9
**14**	−10.1	−7.3	−7.8	−8.3	−10.5	−9.4	−8.1	−7.9	−7.8
**15**	−10.4	−8.4	−8.9	−8.9	−10.7	−10.9	−9.0	−9.2	−9.6
**16**	−9.2	−8.1	−8.1	−8.8	−9.6	−7.5	−8.7	−8.6	−8.4
**17**	−10.9	−10.3	−8.1	−10.5	−10.1	−10.2	−9.1	−11.4	−9.4
**18**	−11.1	−9.4	−9.0	−11.1	−11.7	−10.1	−10.3	−10.8	−10.8
**19**	−8.8	−8.0	−8.4	−8.6	−8.8	−8.8	−8.5	−8.8	−8.7
**20**	−11.6	−11.0	−9.5	−11.5	−10.9	−11.2	−9.4	−11.3	−10.4
**21**	−9.8	−8.6	−8.3	−9.2	−9.4	−9.9	−9.1	−9.3	−8.5
**22**	−11.0	−8.0	−9.5	−8.6	−11.0	−11.7	−4.2	−7.0	−9.3
**23**	−9.4	−8.8	−8.5	−9.4	−9.8	−9.2	−9.3	−9.3	−8.5
**24**	−11.5	−10.4	−9.7	−11.5	−11.3	−10.9	−10.4	−11.3	−10.7
**25**	−10.5	−9.0	−8.7	−9.2	−10.3	−9.1	−8.3	−9.2	−8.9
**28i**	−7.3	−7.8	−4.4	−4.5	−3.2	−10.8	−6.2	−9.3	6.3
**28j**	−9.8	−8.2	−6.6	−8.3	−10.9	−9.1	1.2	−8.7	15.5
**28k**	−8.7	−9.1	−7.1	−10.1	−10.2	−11.0	−4.9	−10.1	9.8
**28l**	−9.7	−8.9	−6.3	−9.8	−11.2	−10.5	−6.6	−9.9	3.8
Imatinib	−11.9								
Nilotinib					−13.2				
Ponatinib	−11.6				−11.6				
Rebastinib						−12.6			
Sorafenib				−11.5					

* Abl tyrosine kinase with T315I mutation.

**Table 5:** Calculated binding affinities for complexes of target structures with protein kinases and reference compounds.

Comp.	AutoDock Vina Score	PDB Code for Protein Kinases	VdW	Electrostatic	Polar Solvation	Binding Energy
**9**	−11.4	2HYY	−270.9 ± 10.7	−68.5 ± 11.1	222.2 ± 14.4	−144.1 ± 12.5
**10**	−12.3	3QRJ *	−289.1 ± 11.8	−18.2 ± 14.1	175.7 ± 21.0	−160.0 ± 13.7
**11**	−11.6	2HYY	−261.1 ± 11.6	−38.4 ± 10.2	187.6 ± 14.8	−137.6 ± 13.9
**11**	−12.2	3QRJ *	−261.2 ± 14.4	−20.7 ± 7.6	168.1 ± 17.1	−139.2 ± 15.6
**11**	−11.9	3CS9	−259.0 ± 10.3	−39.4 ± 15.6	173.2 ± 17.4	−151.5 ± 11.8
**13**	−11.7	3CS9	−257.5 ± 14.9	−34.7 ± 8.2	200.3 ± 17.8	−117.9 ± 17.6
**15**	−10.7	3CS9	−269.2 ± 11.1	−65.9 ± 8.5	239.0 ± 16.0	−122.3 ± 17.1
**18**	−11.2	2HYY	−215.1 ± 12.6	−69.8 ± 14.3	208.2 ± 22.0	−99.1 ± 18.0
**20**	−11.5	3WZE	−271.5 ± 13.0	−41.9 ± 14.1	203.3 ± 19.8	−136.2 ± 14.7
**22**	−11.5	2HYY	−272.3 ± 17.3	−51.8 ± 12.9	202.8 ± 17.2	−148.2 ± 15.7
**24**	−11.2	2HYY	−250.5 ± 12.3	−23.0 ± 8.6	161.9 ± 18.0	−135.3 ± 17.9
**24**	−11.5	3WZE	−238.3 ± 14.7	−26.9 ± 8.0	172.0 ± 8.3	−115.9 ± 14.5
**28l**	−12.0	3CS9	−290.5 ± 14.6	−69.0 ± 20.3	238.9 ± 41.2	−148.6 ± 19.5
**Reference**						
imatinib	−11.9	2HYY	−282.1 ± 10.0	−42.4 ± 7.4	195.6 ± 10.4	−154.7 ± 12.3
ponatinib	−11.6	2HYY	−290.9 ± 10.9	−32.9 ± 8.7	191.0 ± 20.0	−159.4 ± 18.4
ponatinib	−11.6	3CS9	−273.2 ± 13.1	−47.7 ± 15.2	198.4 ± 24.1	−149.3 ± 20.2
nilotinib	−13.2	3CS9	−304.1 ± 12.4	−63.2 ± 10.6	216.4 ± 13.2	−177.7 ± 11.6
rebastinib	−12.6	3QRJ *	−322.1 ± 10.7	−77.0 ± 13.0	250.3 ± 13.8	−178.0 ± 14.2
sorafenib	−11.5	3WZE	−254.3 ± 12.4	−73.9 ± 8.4	181.0 ± 9.8	−170.0 ± 12.0

* Abl tyrosine kinase with T315I mutation.

## Supplementary Materials

The following are available online.
