# Supplementary material for: Synthesis, Biological Activities and Docking Studies of Novel 4-(Arylaminomethyl)benzamide Derivatives as Potential Tyrosine Kinase Inhibitors
Source: Molecules. 2019 Sep 30;24(19):3543. doi: 10.3390/molecules24193543 (PMC6804006; doi:10.3390/molecules24193543)
Supplement: Supplementary file 1 [file molecules-24-03543-s001.pdf]

*Supplementary Materials*

# Synthesis, Biological Activities and Docking Studies of Novel 4-(Arylaminomethyl)benzamide Derivatives as Potential Tyrosine Kinase Inhibitors

**Elena Kalinichenko*, Aliaksandr Faryna, Viktoria Kondrateva, Alena Vlasova, Valentina Shevchenko, Alla Melnik, Olga Avdoshko, Alla Belko**

Institute of Bioorganic Chemistry, National Academy of Sciences of Belarus, BY-220141 Minsk, Belarus; kalinichenko@iboch.by

* Correspondence: kalinichenko@iboch.by Tel.: +375-17-265-06-11; Fax: +375-17-265-06-11

## Table of contents

Experimental data of compounds 5-25, 28i-28l ........ S2

Biological assays ........ S67

Computational methods ........ S67

***4-(chloromethyl)-N-(3-(4-methyl-1H-imidazol-1-yl)-5-(trifluoromethyl)phenyl)benzamide* (5):**
$^{1}$H-NMR (DMSO-$d_6$, 500 MHz) δ: 10.74 (s, 1H), 8.32 (s, 1H), 8.26 (d, J = 1.0 Hz, 1H), 8.17 (s, 1H), 8.03 (d, J = 8.3 Hz, 2H), 7.77 (s, 1H), 7.66 (d, J = 8.0 Hz, 2H), 7.52 (s, 1H), 4.89 (s, 2H), 2.21 (s, 3H); $^{13}$C-NMR (DMSO-$d_6$, 126 MHz) δ: 166.1, 142.2, 141.7, 139.4, 138.4, 135.5, 134.4, 129.4, 128.6, 115.4, 114.7, 112.2, 45.8, 14.0. HRMS(ESI+) m/z calcd for $C_{19}H_{15}ClF_3N_3O$ [M + H]+ 394.0856, found 394.0927. Purity: 97.93% (by HPLC).

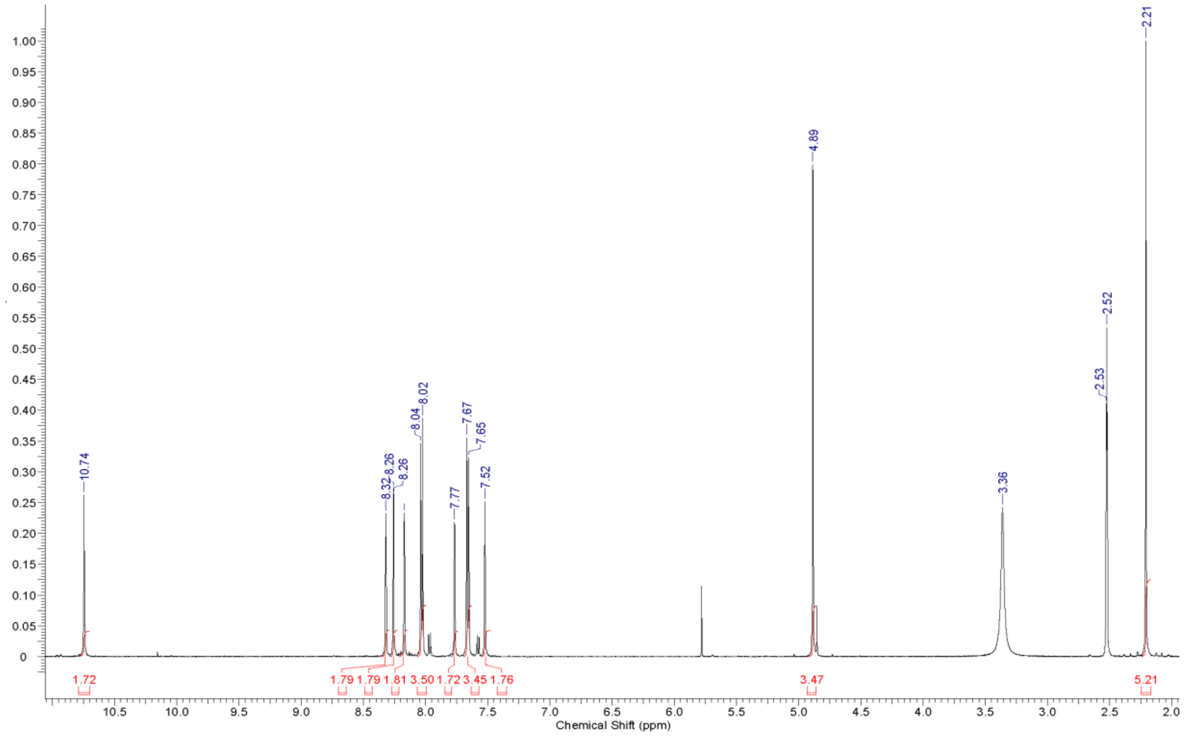


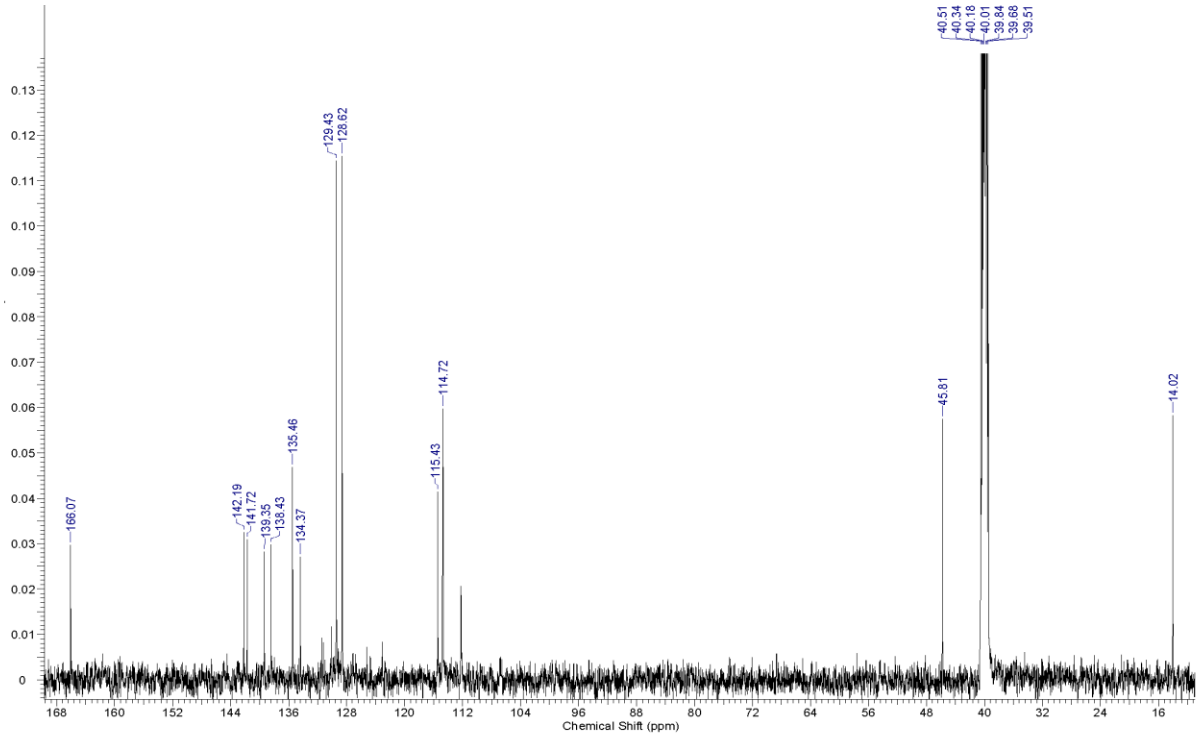

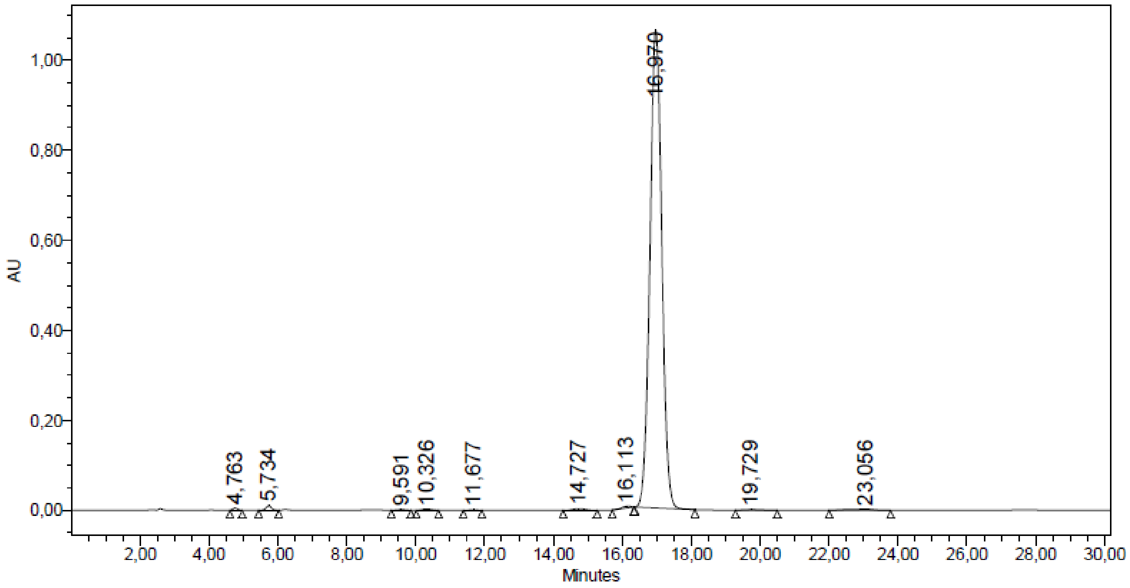


| | RT | Area | % Area | Height |
|---|---|---|---|---|
| 1 | 4,763 | 41880 | 0,16 | 4918 |
| 2 | 5,734 | 107148 | 0,41 | 10424 |
| 3 | 9,591 | 18116 | 0,07 | 1190 |
| 4 | 10,326 | 40586 | 0,16 | 2237 |
| 5 | 11,677 | 14428 | 0,06 | 948 |
| 6 | 14,727 | 85526 | 0,33 | 3538 |
| 7 | 16,113 | 71276 | 0,27 | 4164 |
| 8 | 16,970 | 25417988 | 97,93 | 1061852 |
| 9 | 19,729 | 38034 | 0,15 | 1186 |
| 10 | 23,056 | 118991 | 0,46 | 2575 |

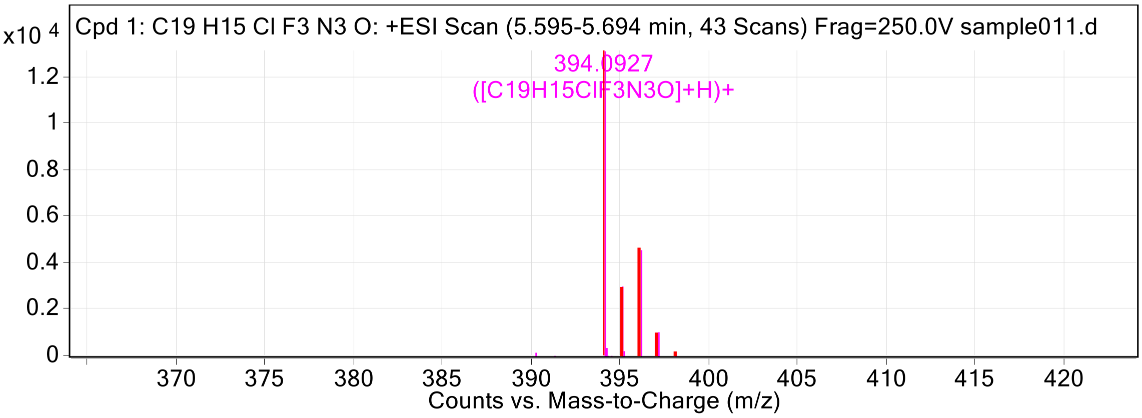

***Methyl 4-({[3-(4-methyl-1H-imidazol-1-yl)-5-(trifluoromethyl)phenyl]amino}methyl)benzoate*** **(6a)** was synthesized from methyl-4-formylbenzoate **(1)** and 3-(4-methyl-1H-imidazol-1-yl)-5-(trifluoromethyl)aniline **(a)** as a white solid. The yield was 81%. $^{1}$H-NMR (DMSO-$d_6$, 500 MHz) δ: 8.13 (d, *J* = 1.0 Hz, 1H), 7.96 (d, *J* = 8.3 Hz, 2H), 7.54 (d, *J* = 8.3 Hz, 2H), 7.42 (s, 1H), 7.14 (s, 1H), 7.04 (s, 1H), 6.96 (s, 1H), 6.86 (s, 1H), 4.54 (d, *J* = 6.1 Hz, 2H), 4.51 - 4.56 (m, 2H), 3.85 (s, 3H), 2.15 (s, 3H); $^{13}$C-NMR (DMSO-$d_6$, 126 MHz) δ: 166.6, 150.7, 145.6, 139.1, 138.9, 135.3, 129.9, 127.9, 114.6, 107.6, 106.1, 103.8, 52.5, 46.2, 14.0. HRMS(ESI+) m/z calcd for $C_{20}H_{18}F_3N_3O_2$ [M + H]+ 390,1351, found 390.1425.

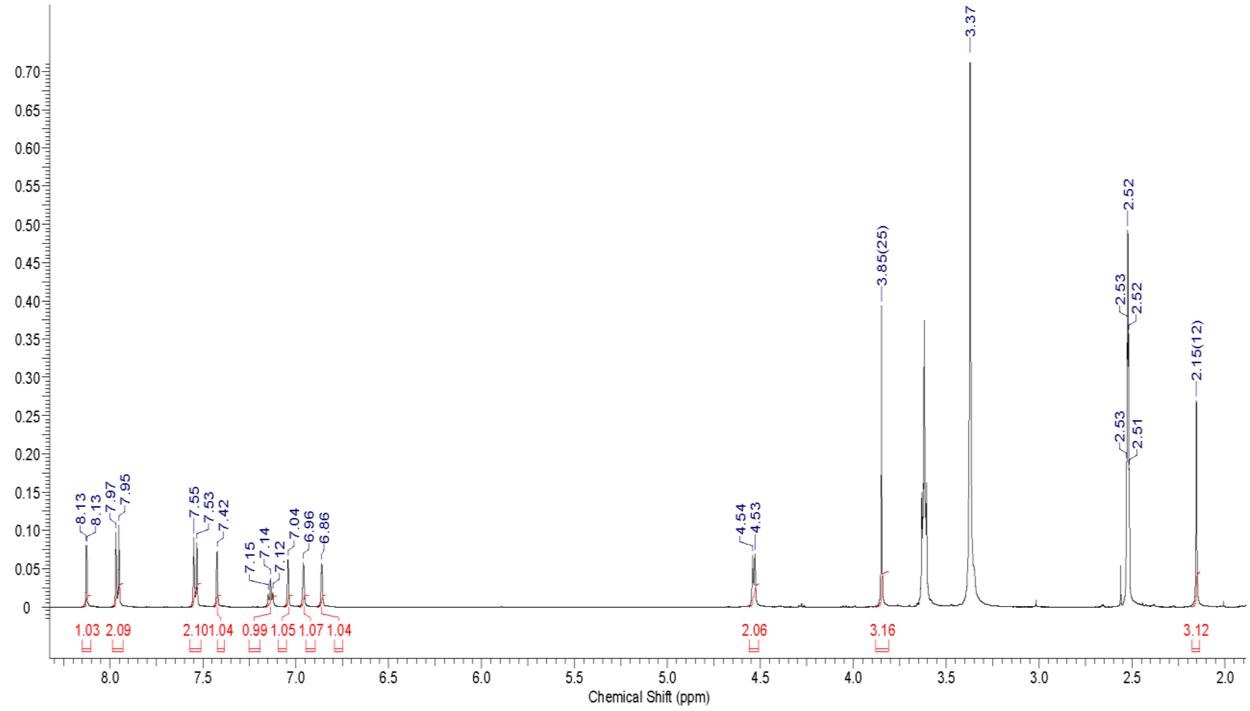


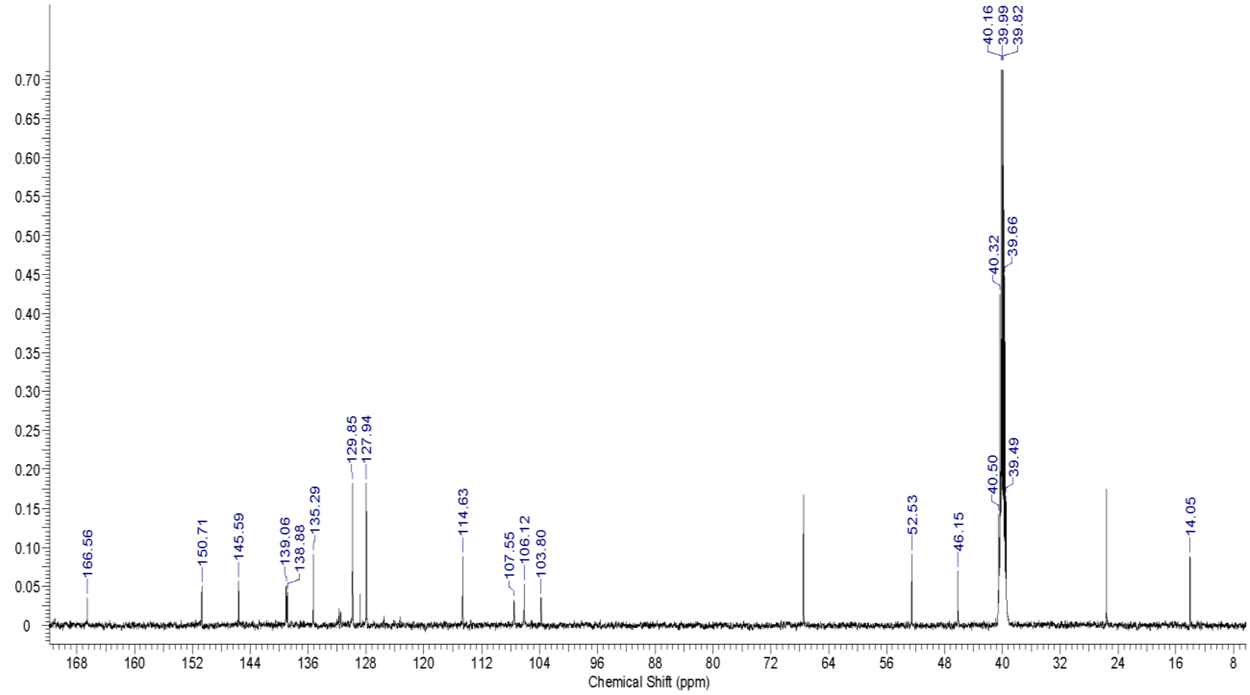

***Methyl-4-{[(2-methyl-5-nitrophenyl) amino) methyl} benzoate* (6b)** was synthesized from methyl-4-formylbenzoate **(1)** and 2-methyl-5-nitroaniline **(b)** as a yellow solid. The yield was 93%. $^{1}$H-NMR (DMSO-$d_6$, 500 MHz) δ: 7.95 (d, *J* = 8.3 Hz, 2H), 7.53 (d, *J* = 8.3 Hz, 2H), 7.37 (dd, *J* = 8.0, 2.2 Hz, 1H), 7.26 (d, *J* = 8.0 Hz, 1H), 7.04 (d, *J* = 2.2 Hz, 1H), 6.50 (t, *J* = 6.1 Hz, 1H), 4.56 (d, *J* = 6.1 Hz, 2H), 3.84 (s, 3H), 2.31 (s, 3H); $^{13}$C NMR (DMSO-$d_6$, 126 MHz) δ: 166.6, 147.4, 147.3, 145.8, 131.0, 130.8, 129.9, 128.8, 127.5, 111.1, 103.1, 67.5, 52.5, 46.4, 25.6, 18.5. HRMS(ESI+) m/z calcd for $C_{16}H_{16}N_2O_4$ [M + H]+ 301.1110, found 301.1185.

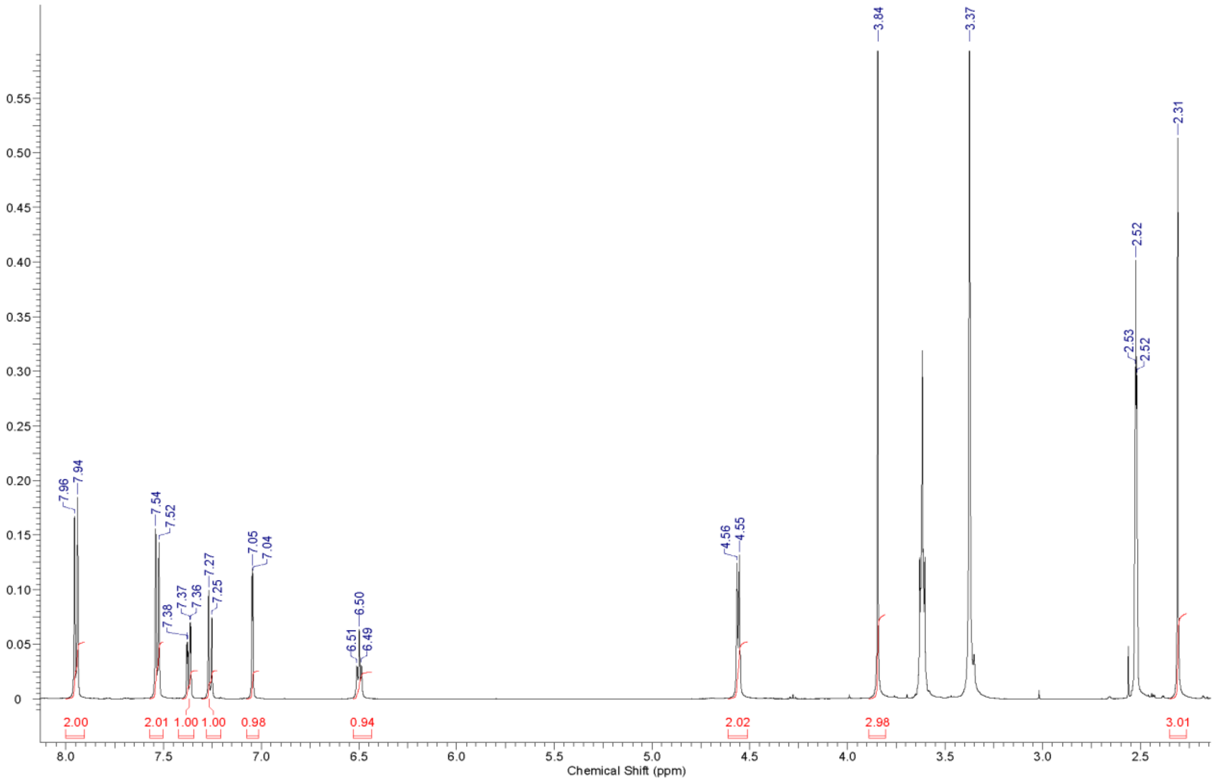


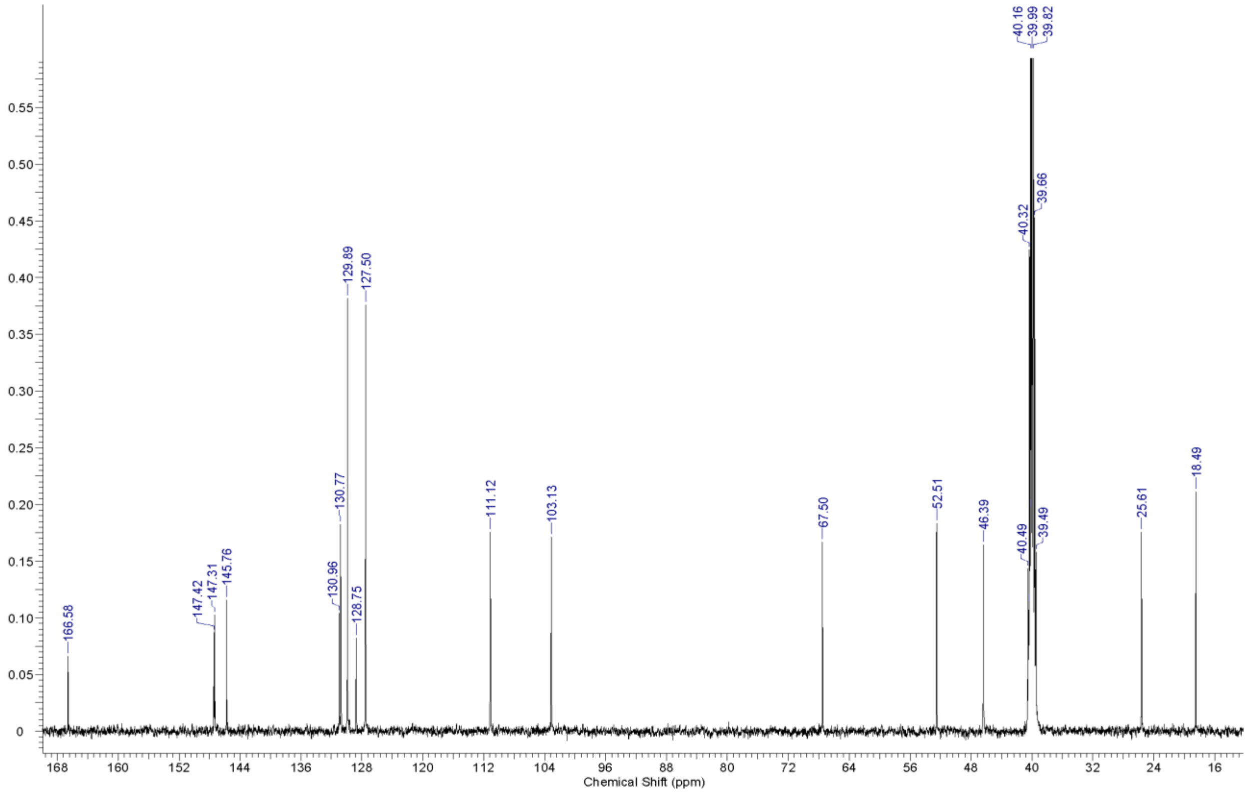

***Methyl 4-{[(4-{[2-(methylcarbamoyl)pyridin-4-yl]oxy}phenyl)amino]methyl}benzoate*** **(6c)** was synthesized from methyl-4-formylbenzoate **(1)** and 4-(4-aminophenoxy)-N-methylpicolinamide **(c)** as a white solid. The yield was 88%. $^{1}$H-NMR (DMSO-$d_6$, 500 MHz) $\delta$: 8.73 - 8.79 (m, 1H), 8.44 - 8.49 (m, 1H), 7.93 - 7.98 (m, 2H), 7.53 - 7.58 (m, 2H), 7.31 - 7.35 (m, 1H), 7.06 - 7.10 (m, 1H), 6.90 - 6.96 (m, 2H), 6.64 - 6.68 (m, 2H), 6.52 - 6.57 (m, 1H), 4.38 - 4.43 (m, 2H), 3.86 (s, 3H), 2.79 (d, $J$ = 4.8 Hz, 3H); $^{13}$C-NMR (DMSO-$d_6$, 500 MHz) $\delta$: 221.7, 221.5, 221.0, 218.0, 217.5, 216.6, 216.5, 215.8, 212.3, 212.0, 211.8, 210.4, 208.4, 208.2, 207.0, 192.8, 191.5, 186.3. HRMS(ESI+) m/z calcd for $C_{22}H_{21}N_3O_4$ [M + H]+ 392,1532, found 392.1602.

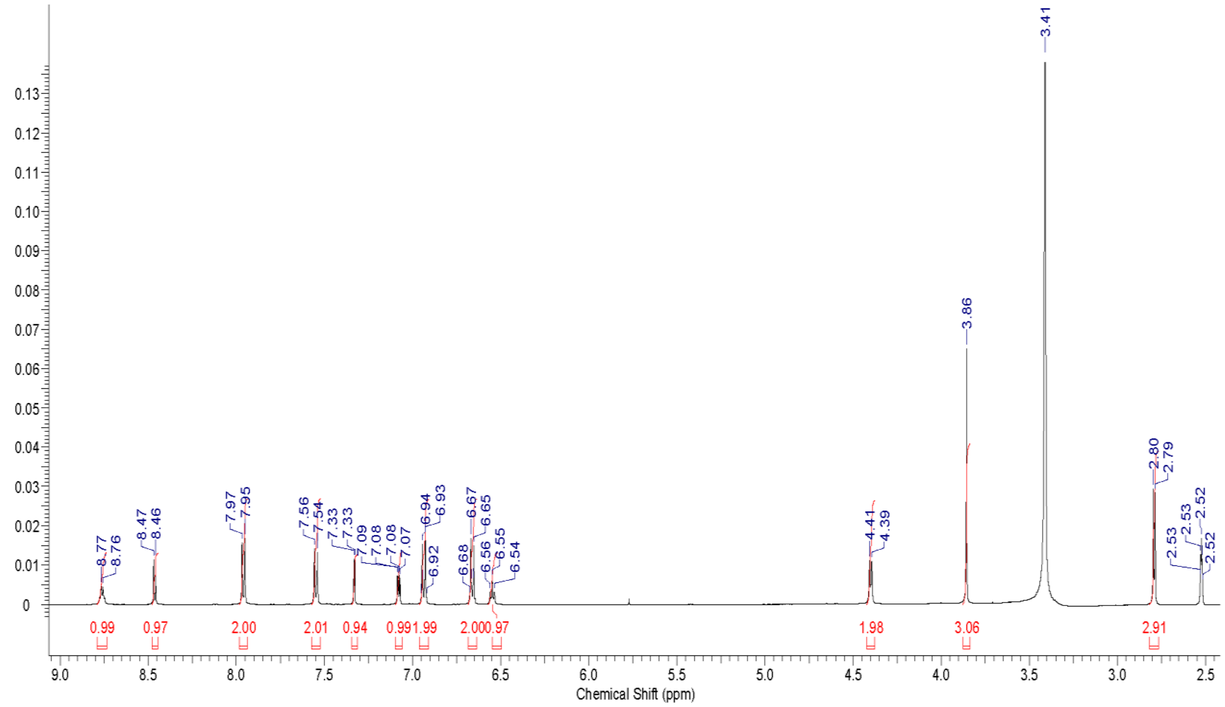


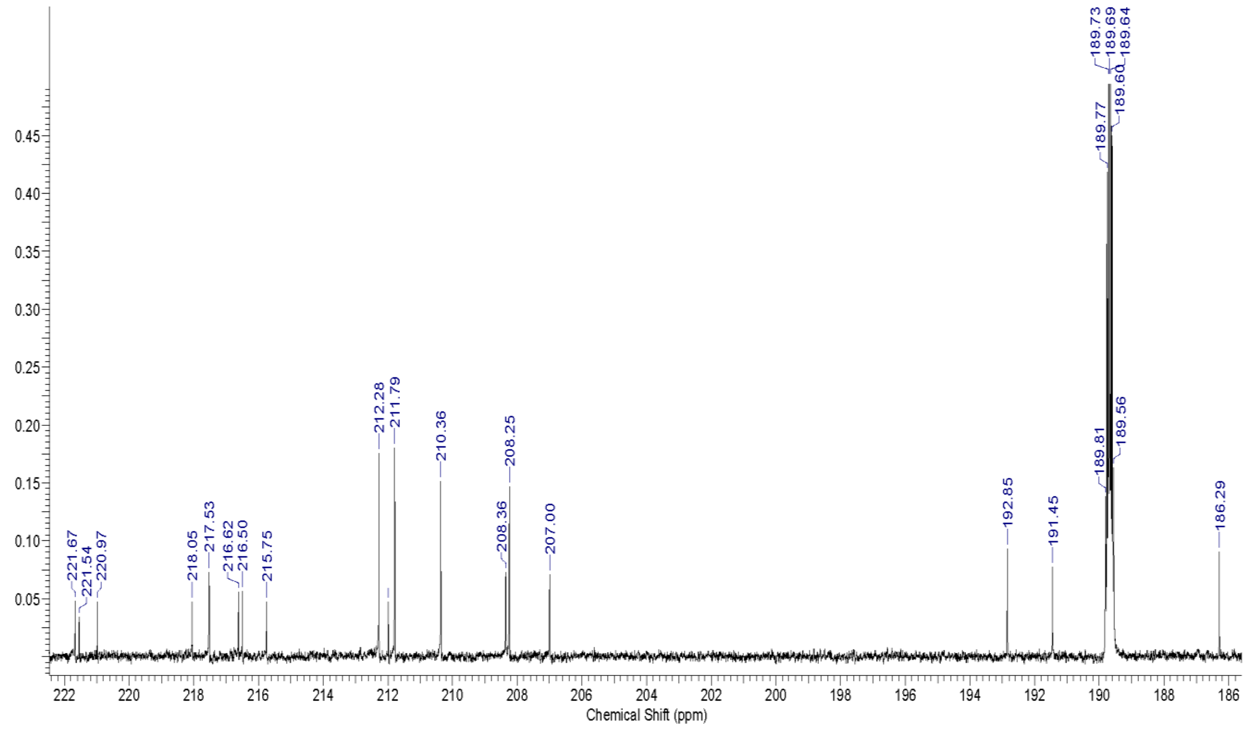

***Methyl 4-({[4-(pyridin-3-yl)pyrimidin-2-yl]amino}methyl)benzoate*** **(6d)** was synthesized from methyl-4-formylbenzoate **(1)** and 4-(pyridin-3-yl)pyrimidin-2-amine (**d**) as a white solid. The yield was 81%. $^1$H-NMR (DMSO-*d*$_6$, 500 MHz) $\delta$: 9.23 (br.s., 1H), 8.70 (br. s., 1H), 8.43 (d, *J* = 5.1 Hz, 2H), 8.03 (t, *J* = 6.1 Hz, 1H), 7.93 (d, *J* = 8.3 Hz, 2H), 7.53 (br.s., 3H), 7.28 (d, *J* = 5.1 Hz, 1H), 4.67 (d, *J* = 5.8 Hz, 2H), 3.84 (s, 3H); $^{13}$C-NMR (DMSO-*d*$_6$, 126 MHz) $\delta$: 166.6, 162.9, 151.7, 148.5, 146.9, 134.7, 132.8, 129.7, 128.4, 127.7, 124.3, 52.5, 44.5. HRMS(ESI+) m/z calcd for $C_{18}H_{16}N_4O_2$ [M + H]+ 321.1273, found 321.1347.

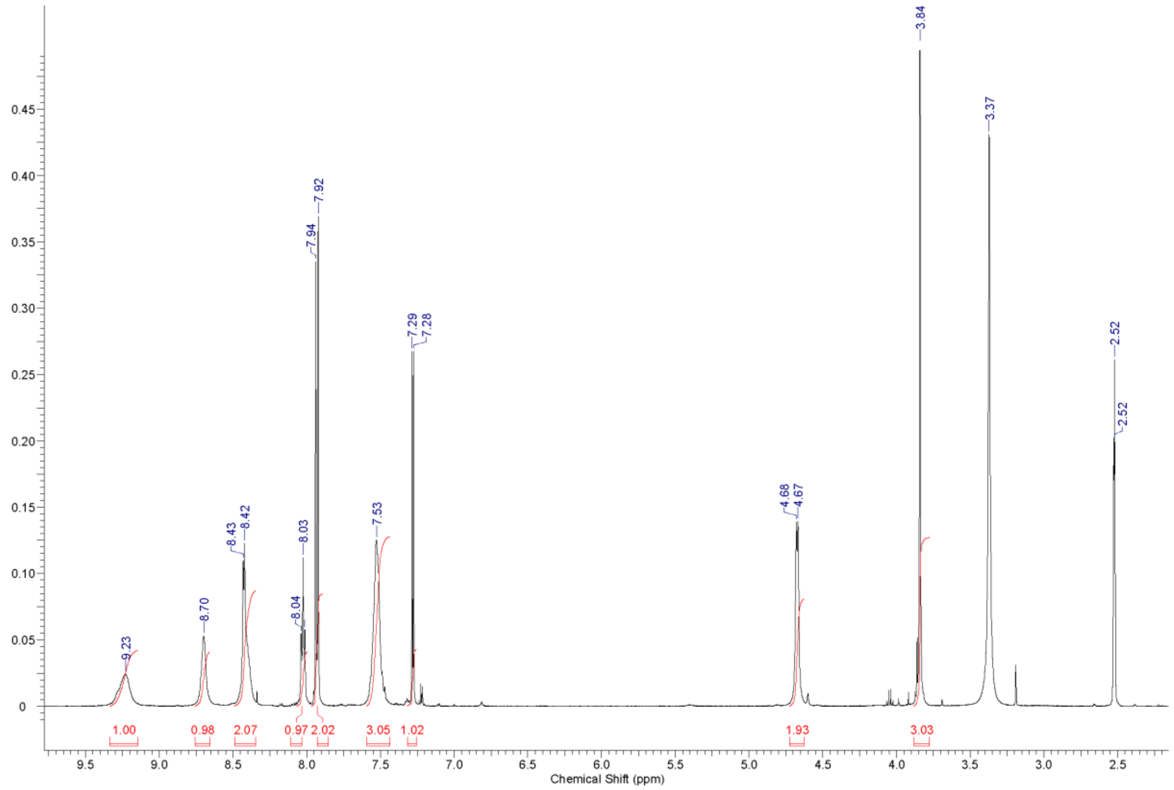


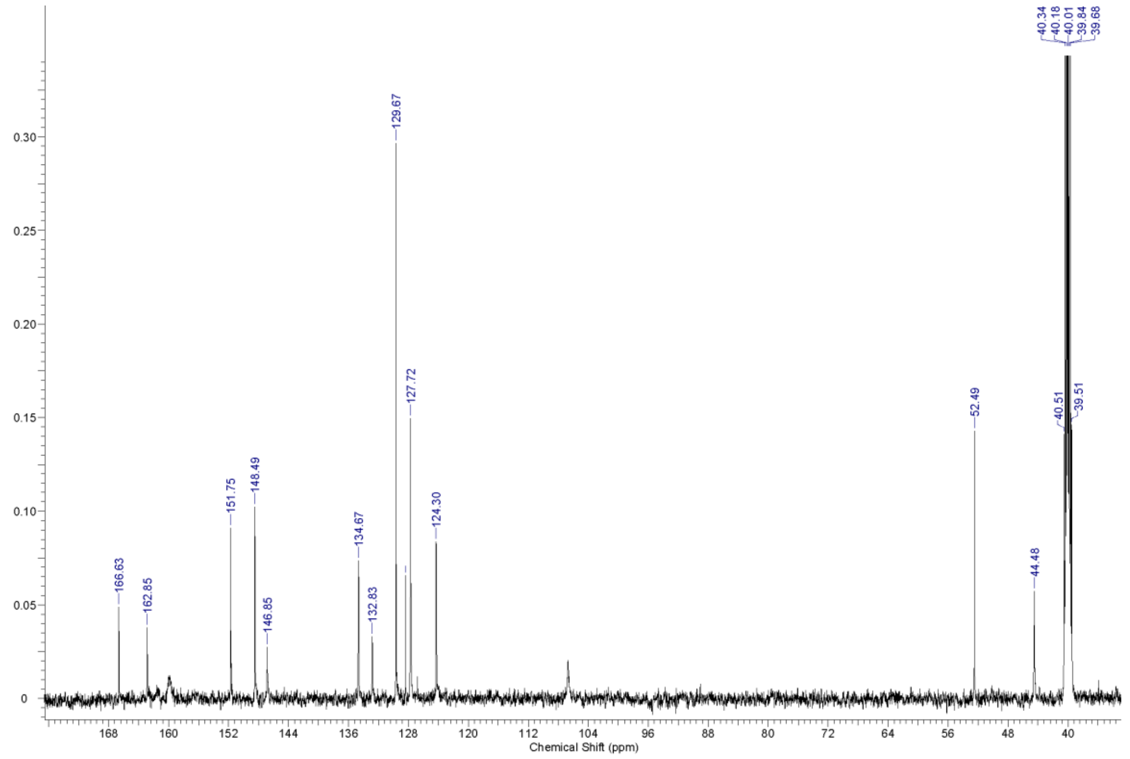

***4-({[3-(4-methyl-1H-imidazol-1-yl)-5-(trifluoromethyl)phenyl]amino}methyl)benzoic acid*** **(7a)** was synthesized from compound **6a** as a white solid. The yield was 82%. $^{1}$H-NMR (DMSO-$d_6$, 500 MHz) $\delta$: 8.90 (s, 1H), 7.94 (d, $J$ = 8.3 Hz, 1H), 7.73 (s, 1H), 7.51 (d, $J$ = 8.0 Hz, 1H), 7.29 (t, $J$ = 5.9 Hz, 1H), 7.14 (s, 1H), 7.06 (s, 1H), 6.98 (s, 1H), 4.54 (d, $J$ = 5.4 Hz, 1H), 2.26 (s, 3H); $^{13}$C-NMR (DMSO-$d_6$, 126 MHz) $\delta$: 167.6, 150.8, 144.8, 138.1, 134.8, 130.0, 127.8, 116.3, 108.9, 106.9, 104.6, 46.1, 12.0. HRMS(ESI+) m/z calcd for $C_{19}H_{16}F_3N_3O_2$ [M + H]+ 376,1195, found 376.1271.

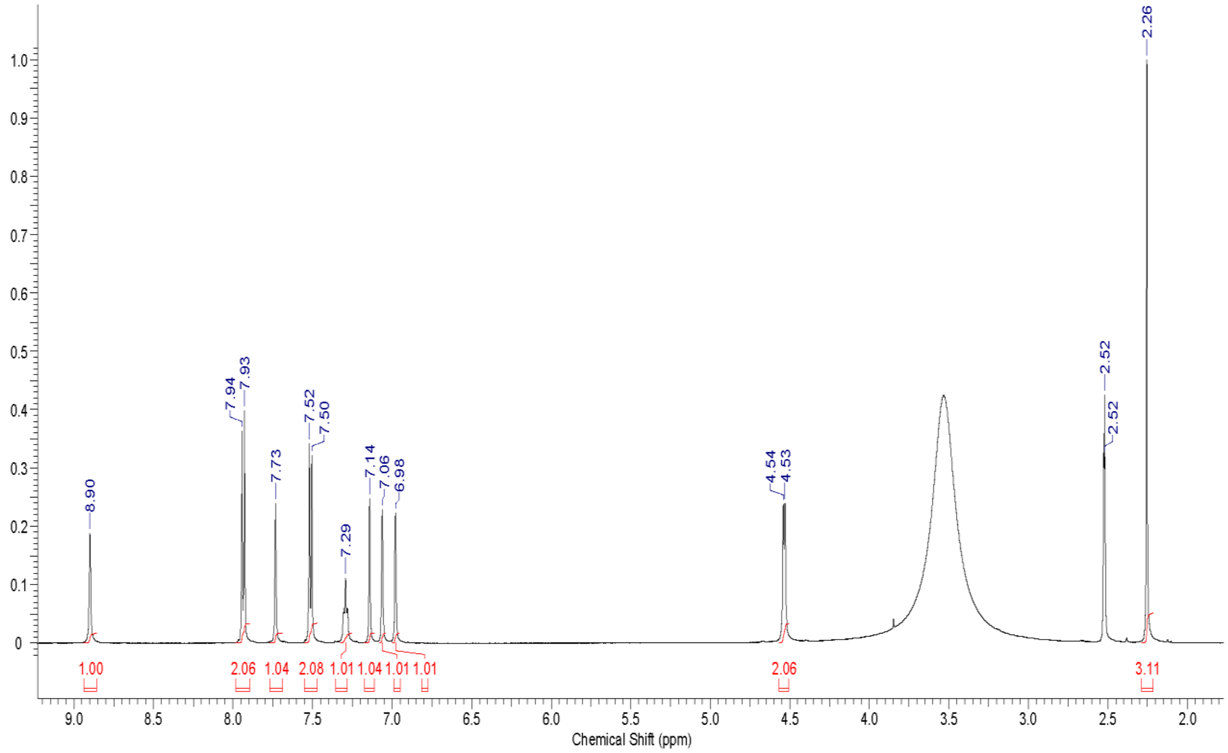


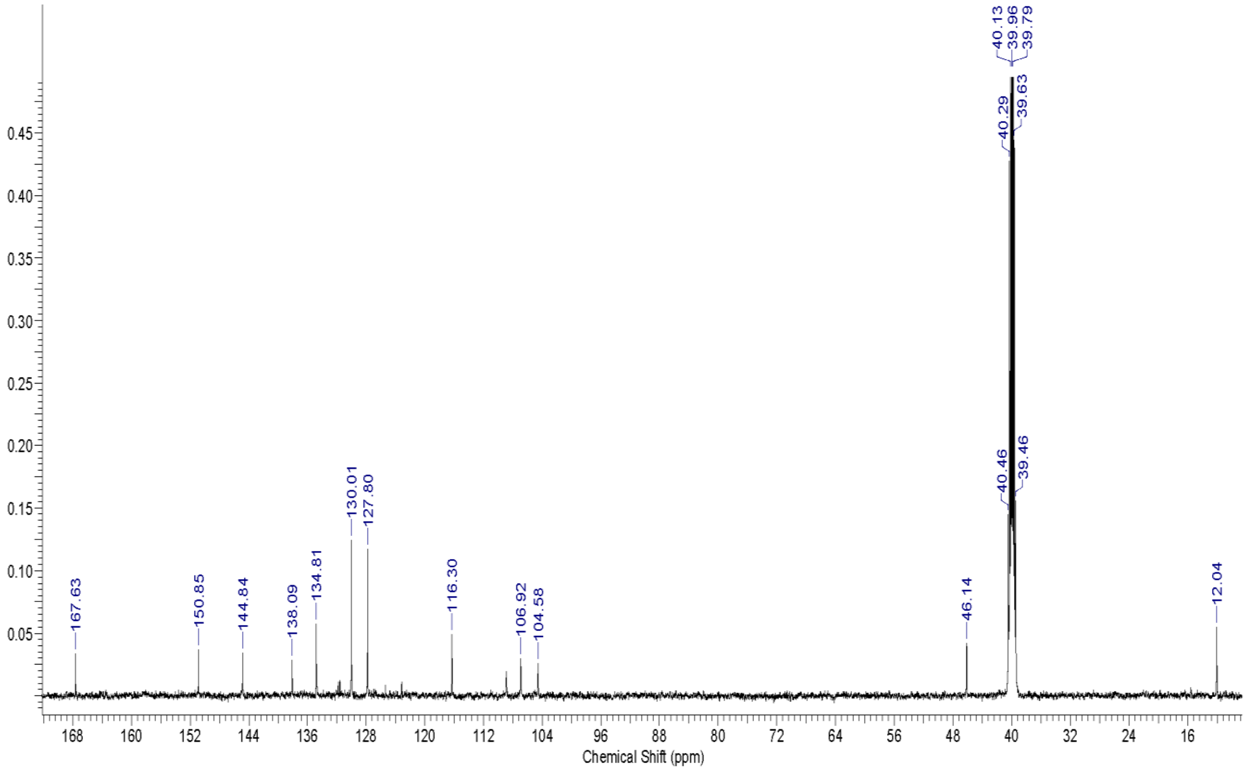

***4 - {[(2-methyl-5-nitrophenyl) amino) methyl} benzoic acid*** **(7b)** was synthesized from compound **6b** as a yellow solid. The yield was 83%. $^{1}$H-NMR (DMSO-*d*$_6$, 500 MHz) δ: 7.92 (d, *J* = 8.3 Hz, 2H), 7.50 (d, *J* = 8.3 Hz, 2H), 7.37 (dd, *J* = 8.0, 2.2 Hz, 1H), 7.26 (d, *J* = 8.3 Hz, 1H), 7.05 (d, *J* = 2.6 Hz, 1H), 6.48 (t, *J* = 6.1 Hz, 1H), 4.55 (d, *J* = 6.1 Hz, 2H), 2.31 (s, 3H); $^{13}$C-NMR (DMSO-*d*$_6$, 126 MHz) δ: 167.7, 147.4, 147.3, 145.2, 131.0, 130.8, 130.0, 129.9, 127.3, 111.1, 103.1, 46.4, 18.5. HRMS(ESI+) m/z calcd for $C_{15}H_{14}N_2O_4$ [M + H]+ 287.0954, found 287.1030.

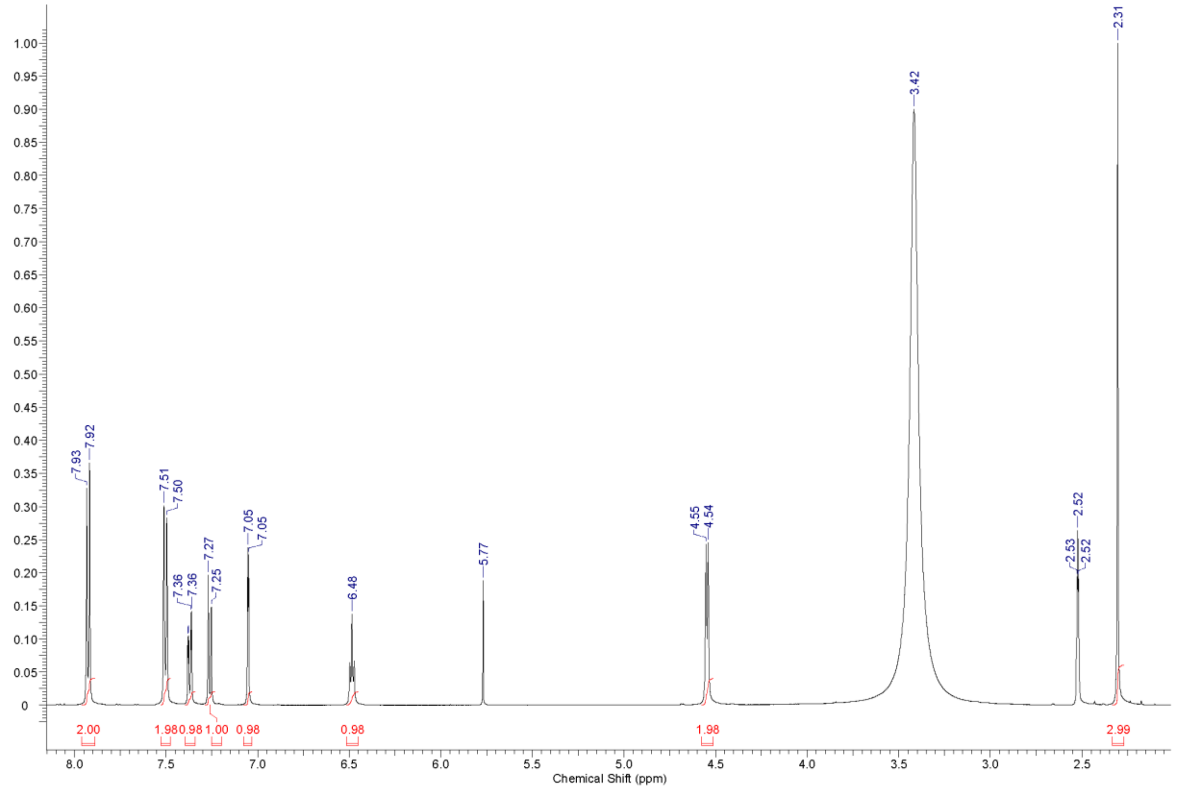


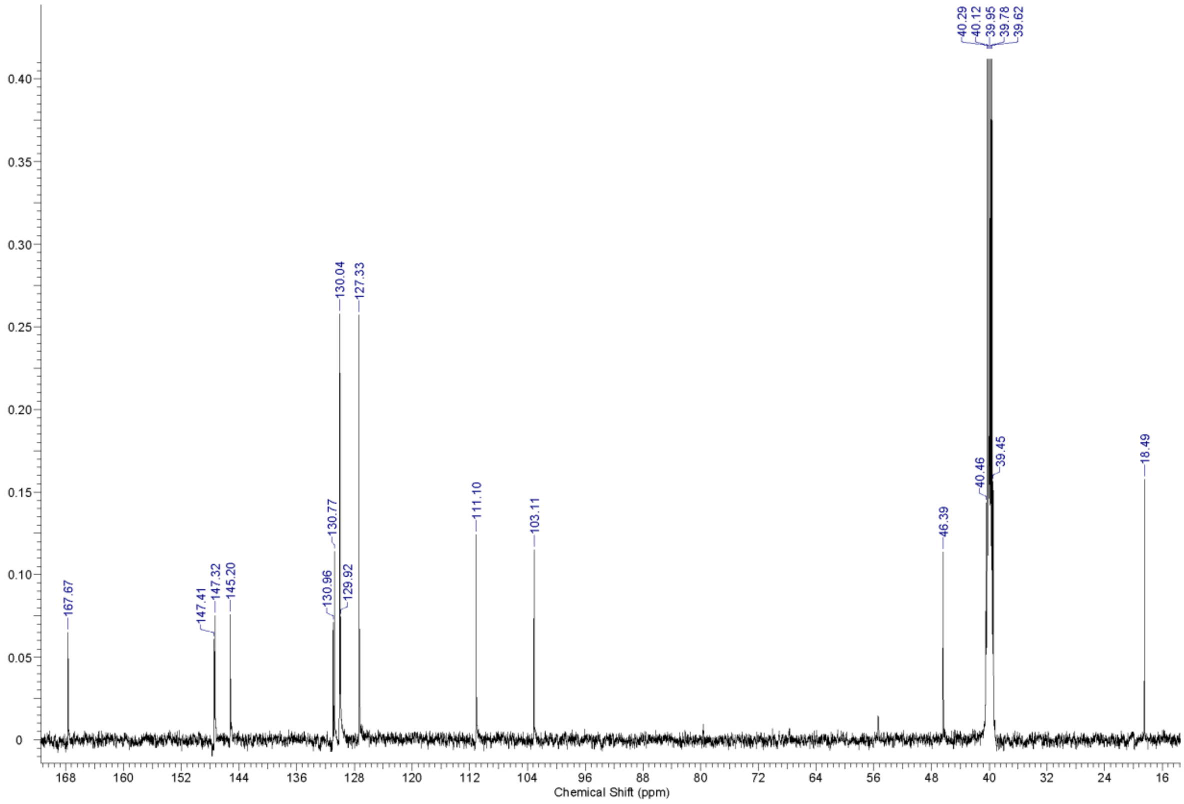

***4-{[(4-{[2-(methylcarbamoyl)pyridin-4-yl]oxy}phenyl)amino]methyl}benzoic acid*** **(7c)** was synthesized from compound **6c** as a white solid. The yield was 79%. $^{1}$H-NMR (DMSO-$d_6$, 500 MHz) $\delta$: 8.76 (q, $J$ = 4.5 Hz, 1H), 8.46 (d, $J$ = 5.8 Hz, 1H), 7.93 (d, $J$ = 8.3 Hz, 2H), 7.51 (d, $J$ = 8.3 Hz, 2H), 7.33 (d, $J$ = 2.6 Hz, 1H), 7.07 (dd, $J$ = 5.6, 2.7 Hz, 1H), 6.93 (d, $J$ = 9.0 Hz, 2H), 6.66 (d, $J$ = 8.7 Hz, 2H), 6.55 (br.s., 1H), 4.38 (d, $J$ = 4.8 Hz, 2H), 2.79 (d, $J$ = 4.8 Hz, 3H); $^{13}$C-NMR (DMSO-$d_6$, 126 MHz) $\delta$: 221.8, 221.7, 221.0, 218.0, 217.5, 216.6, 216.3, 215.7, 212.3, 211.7, 210.4, 208.4, 208.2, 207.0, 191.5, 186.3. HRMS(ESI+) m/z calcd for $C_{21}H_{19}N_3O_4$ [M + H]+ 378,1376, found 378.1454.

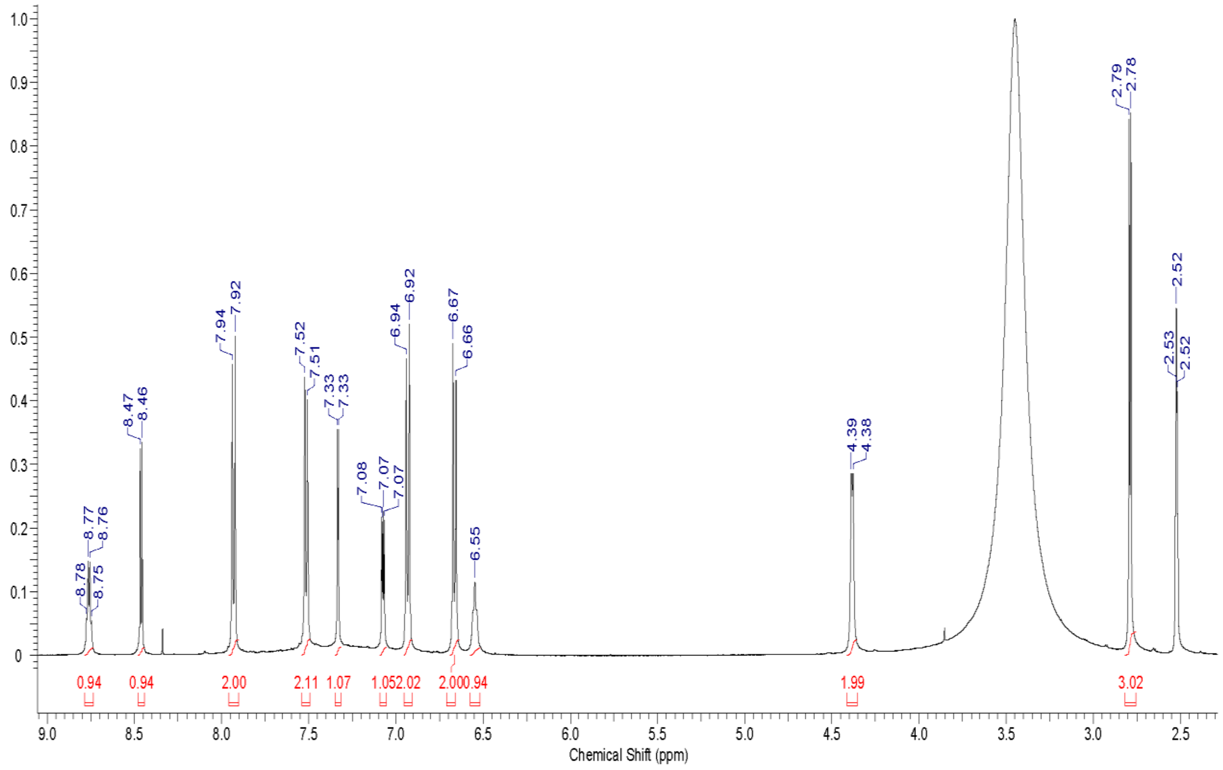


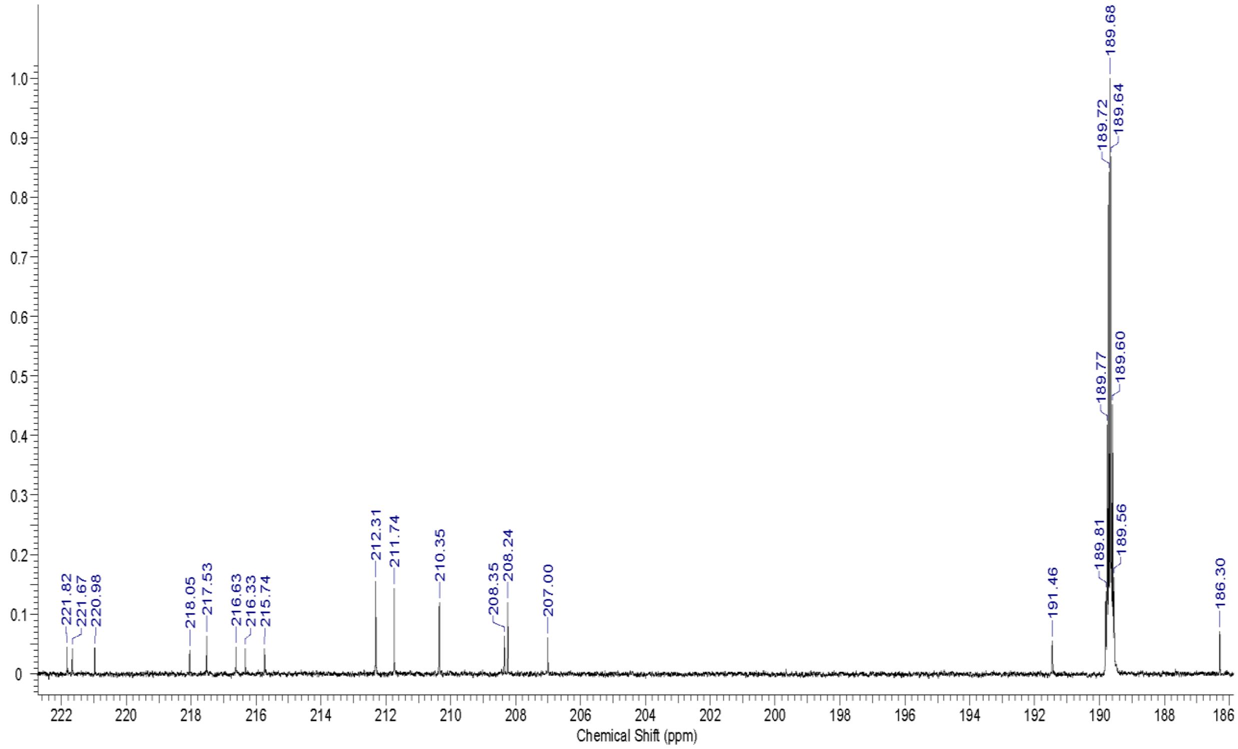

***4-({[4-(pyridin-3-yl)pyrimidin-2-yl]amino}methyl)benzoic acid* (7d)** was synthesized from compound **6d** as a white solid. The yield was 68%. $^{1}$H-NMR (DMSO-*d*6, 500 MHz) δ: 11.72 - 13.85 (m, 1H), 9.34 (br.s., 1H), 8.83 (br.s., 1H), 8.69 (d, *J* = 6.1 Hz, 1H), 8.48 (br.s., 1H), 8.21 (br.s., 1H), 7.91 (d, *J* = 8.0 Hz, 2H), 7.43 - 7.56 (m, 4H), 4.69 (br.s., 2H); $^{13}$C-NMR (DMSO-*d*6, 126 MHz) δ: 167.7, 159.6, 148.9, 146.0, 145.8, 137.9, 133.9, 129.8, 129.6, 127.6, 125.6, 44.5. HRMS(ESI+) m/z calcd for $C_{17}H_{14}N_4O_2$ [M + H]+ 307.1117, found 307.1190.

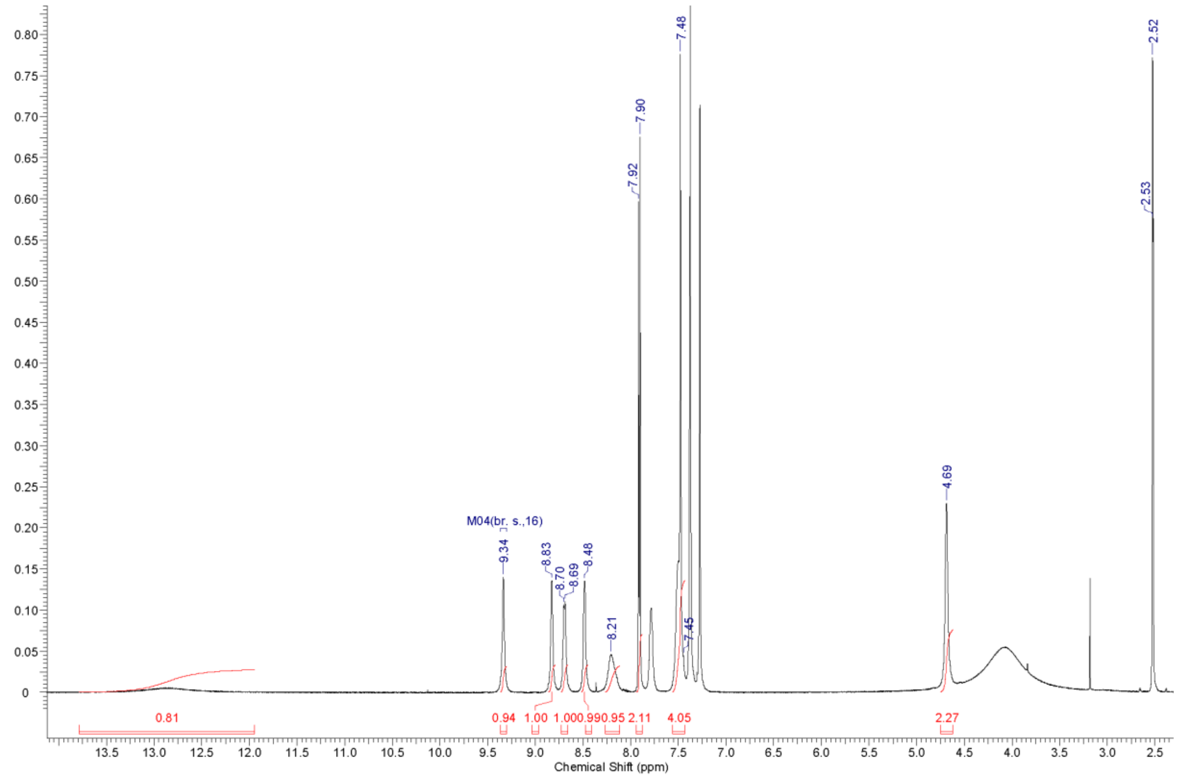


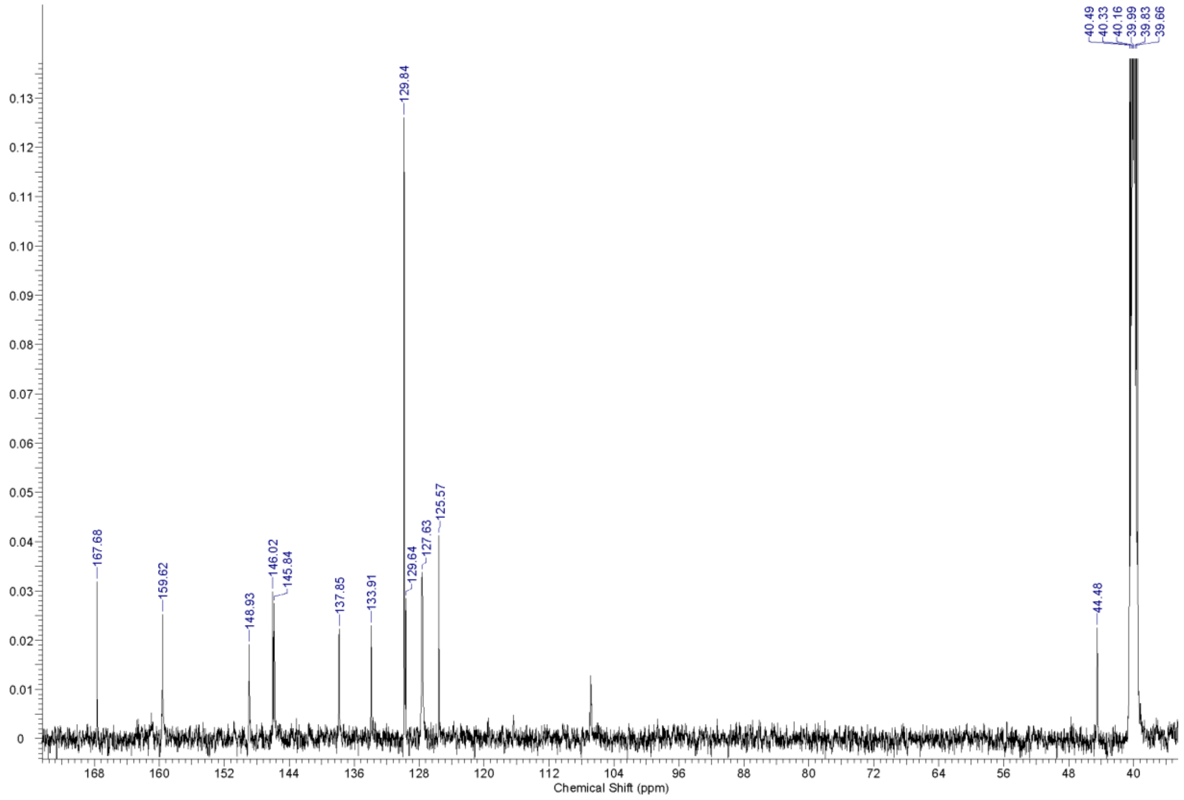

***N-(3-(4-methyl-1H-imidazol-1-yl)-5-(trifluoromethyl)phenyl)-4-(((3-(4-methyl-1H-imidazol-1-yl)-5-(trifluoromethyl)phenyl)amino)methyl)benzamide*** **(8)** was synthesized from compound **5** and 3-(4-methyl-1H-imidazol-1-yl)-5-(trifluoromethyl)aniline **(a)** as a white solid. M.p. 200-202 °C. $^{1}$H-NMR (DMSO-$d_6$, 500 MHz) $\delta$: 11.06 (s, 1H), 10.05 (d, $J$ = 1.6 Hz, 1H), 8.47 (s, 1H), 8.28 (s, 1H), 8.23 (d, $J$ = 1.3 Hz, 1H), 8.18 (d, $J$ = 8.3 Hz, 2H), 8.15 (s, 1H), 7.76 (s, 1H), 7.64 (d, $J$ = 8.3 Hz, 2H), 7.51 (s, 1H), 7.24 (s, 1H), 7.11 (s, 1H), 7.07 (s, 1H), 6.28 (s, 1H), 5.67 (s, 2H), 2.24 (d, $J$ = 0.6 Hz, 2H), 2.20 (s, 3H); $^{13}$C-NMR (DMSO-$d_6$, 126 MHz) $\delta$: 165.9, 151.7, 141.8, 139.4, 138.3, 136.9, 136.6, 135.4, 134.5, 132.1, 129.1, 128.4, 119.3, 115.5, 114.6, 109.8, 105.1, 50.1, 14.0, 9.4. HRMS(ESI+) m/z calcd for $C_{30}H_{24}F_6N_6O$ [M + H]+ 599.1916, found 599.1993.

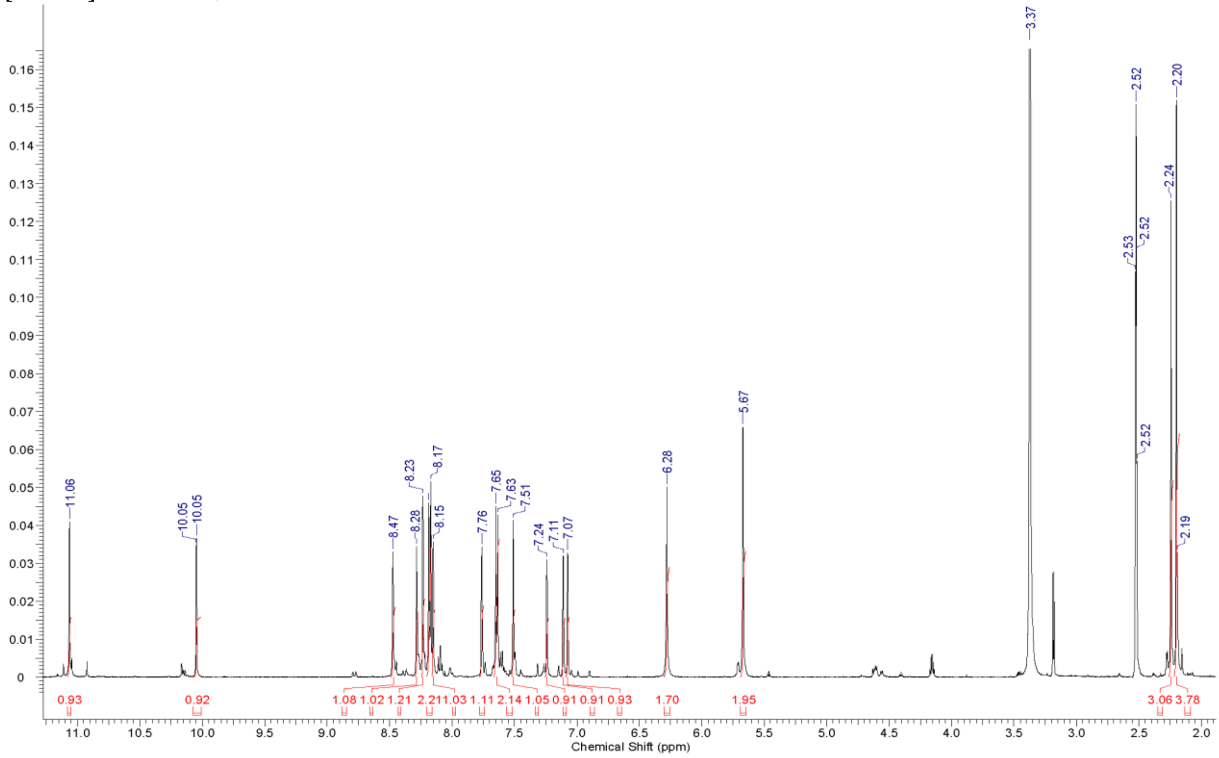


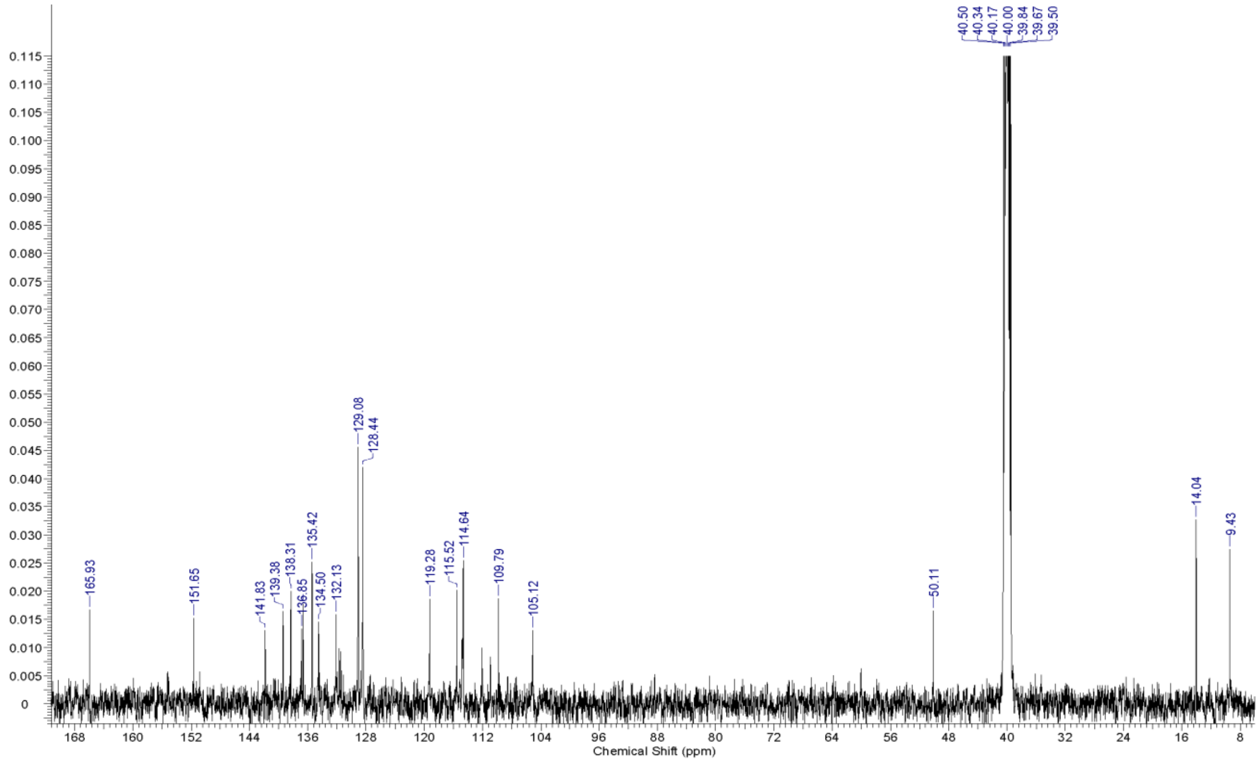

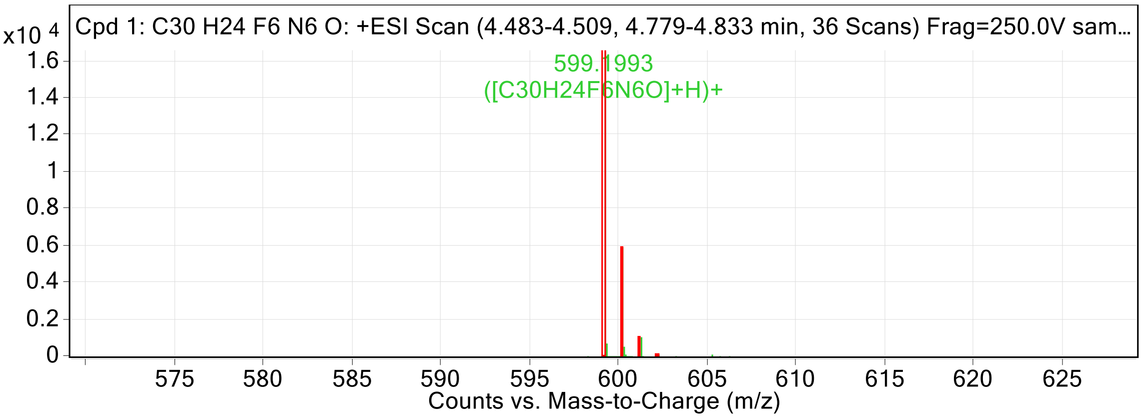

Cpd 1: C30 H24 F6 N6 O: +ESI Scan (4.483-4.509, 4.779-4.833 min, 36 Scans) Frag=250.0V sam...
x10 4
1.6
1.4
1.2
1
0.8
0.6
0.4
0.2
0
599.1993
([C30H24F6N6O]+H)+
575
580
585
590
595
600
605
610
615
620
625
Counts vs. Mass-to-Charge (m/z)

***N*-(3-(4-methyl-1H-imidazol-1-yl)-5-(trifluoromethyl)phenyl)-4-(((2-methyl-5-nitrophenyl)amino)methyl)benzamide** **(9)** was synthesized from compound **5** and 2-methyl-5-nitroaniline **(b)** using general method 3.2.1 or from compound **7b** and 3-(4-methyl-1H-imidazol-1-yl)-5-(trifluoromethyl)aniline **(a)** using general method 3.2.4.2 as a yellow solid. M.p. 113-115 °C. $^{1}$H-NMR (DMSO-*d*$_6$, 500 MHz) *δ*: 10.69 (s, 1H), 8.31 (s, 1H), 8.29 (d, *J* = 1.0 Hz, 1H), 8.16 (s, 1H), 7.98 (d, *J* = 8.0 Hz, 2H), 7.75 (s, 1H), 7.59 (d, *J* = 8.3 Hz, 2H), 7.52 (s, 1H), 7.38 (dd, *J* = 8.0, 2.2 Hz, 1H), 7.27 (d, *J* = 8.3 Hz, 1H), 7.09 (d, *J* = 2.2 Hz, 1H), 6.54 (t, *J* = 6.1 Hz, 1H), 4.58 (d, *J* = 6.1 Hz, 2H), 2.33 (s, 3H), 2.21 (s, 3H); $^{13}$C-NMR (DMSO-*d*$_6$, 126 MHz) *δ*: 166.4, 147.4, 147.3, 144.5, 141.9, 139.0, 138.3, 135.4, 133.2, 131.0, 130.8, 128.5, 127.4, 115.4, 114.8, 111.1, 103.2, 46.3, 18.5, 13.8. HRMS(ESI+) m/z calcd for $C_{26}H_{22}F_3N_5O_3$ [M + H]+ 510.1675, found 510.1747. Purity: 96.63% (by HPLC).

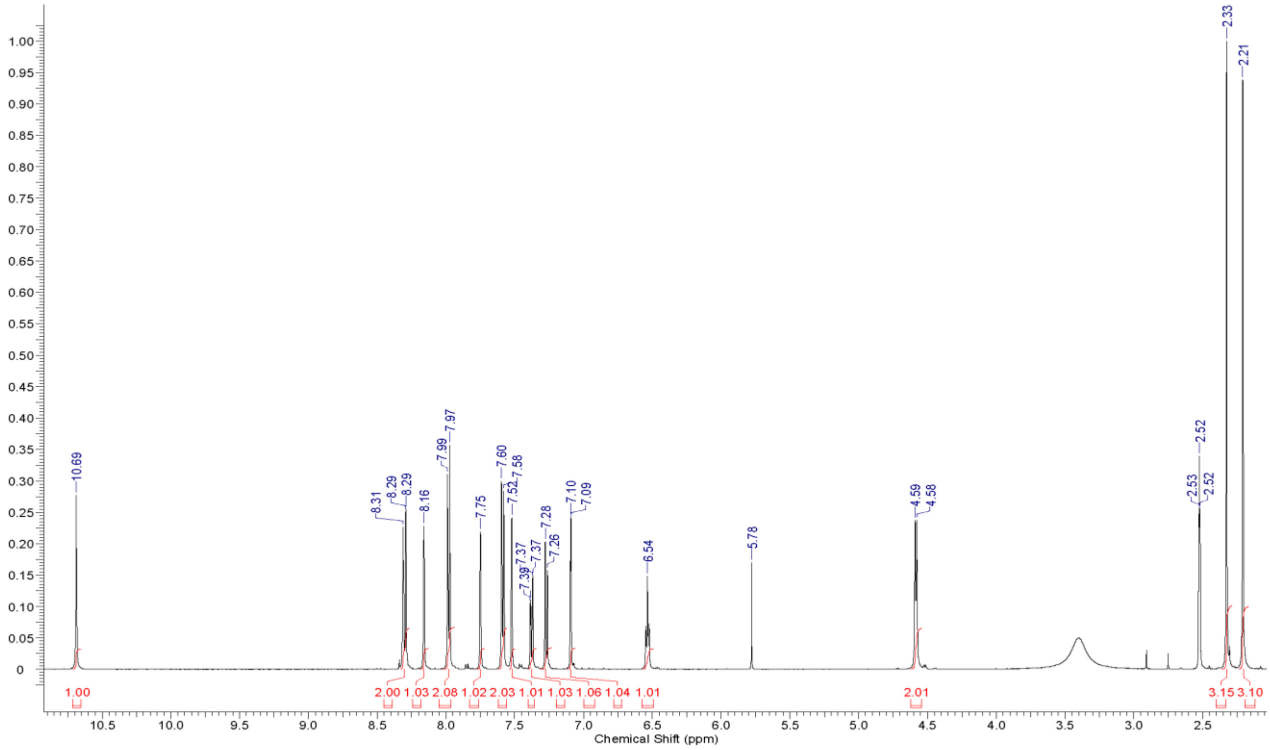

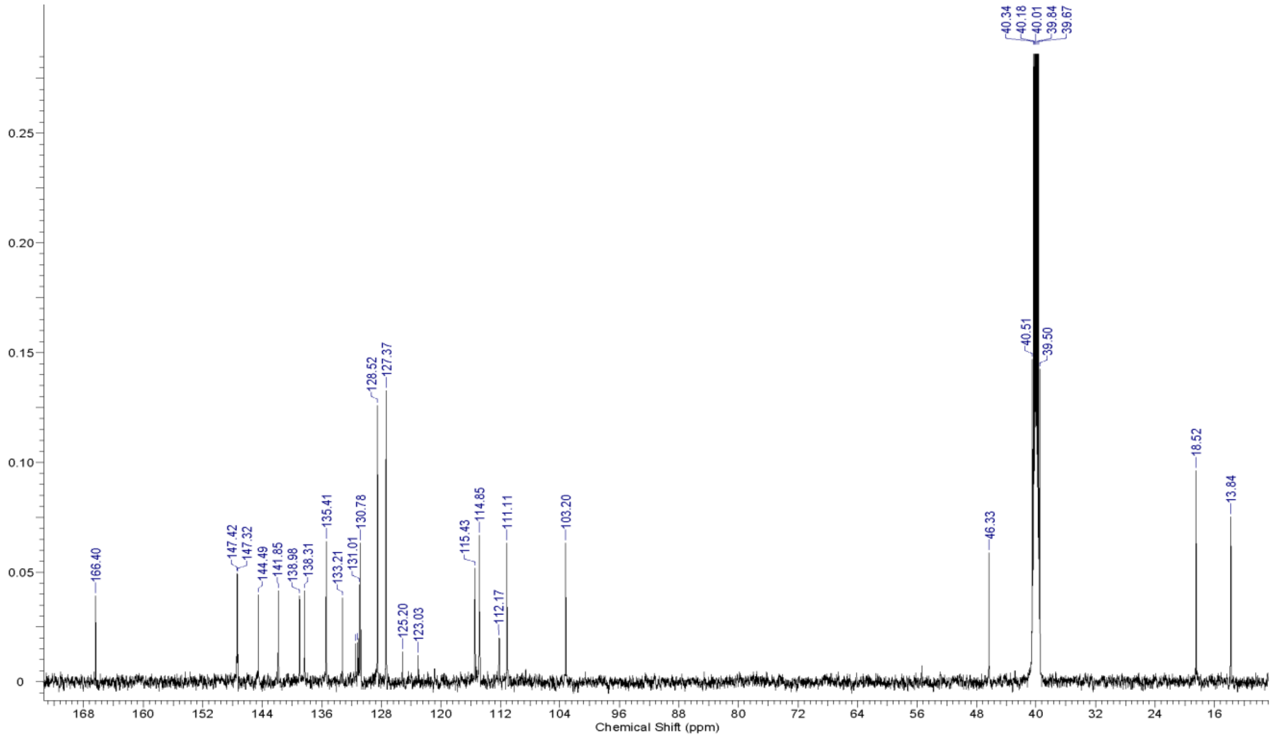

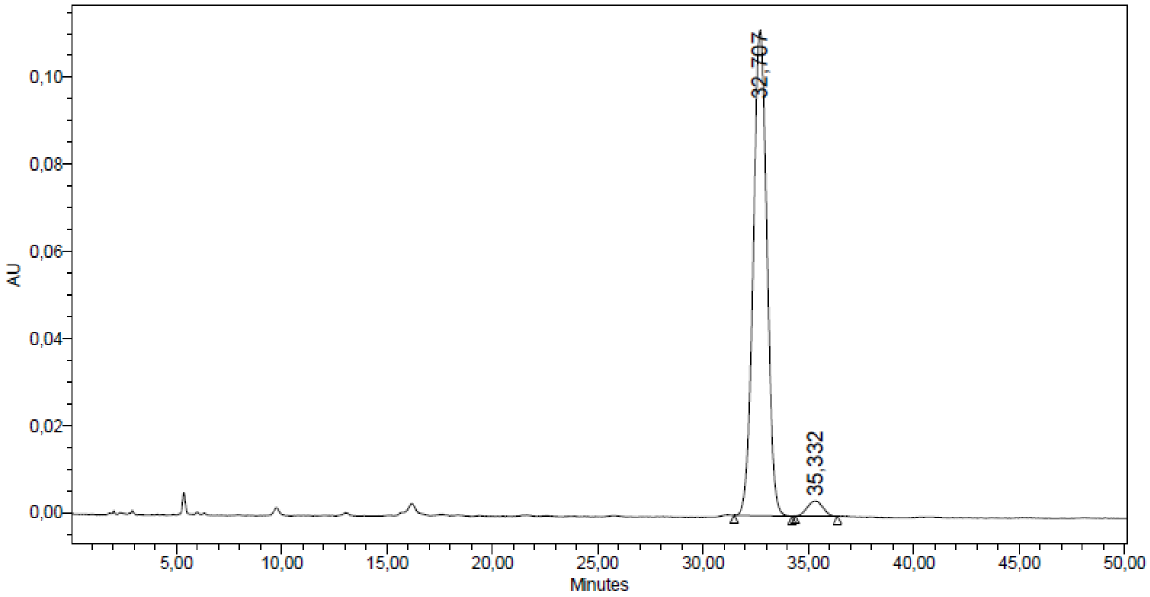

| | RT | Area | % Area | Height |
|---|---|---|---|---|
| 1 | 32,707 | 5037232 | 96,63 | 111462 |
| 2 | 35,332 | 175921 | 3,37 | 3498 |

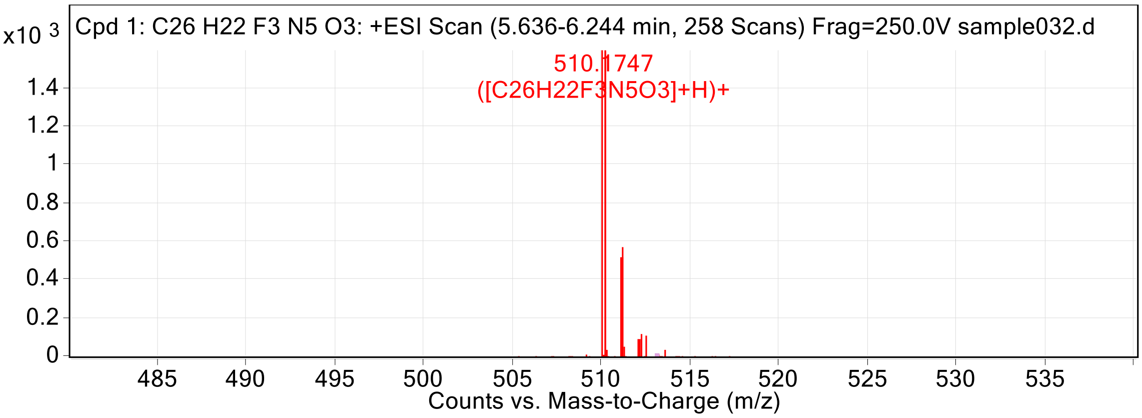
x10 3
Cpd 1: C26 H22 F3 N5 O3: +ESI Scan (5.636-6.244 min, 258 Scans) Frag=250.0V sample032.d
510.1747
([C26H22F3N5O3]+H)+
1.4
1.2
1
0.8
0.6
0.4
0.2
0
485
490
495
500
505
510
515
520
525
530
535
Counts vs. Mass-to-Charge (m/z)

***N-methyl-4-(4-((4-((3-(4-methyl-1H-imidazol-1-yl)-5-(trifluoromethyl)phenyl)carbamoyl)benzyl)amino)phenoxy)picolinamide* (10)** was synthesized from compound **5** and 4-(4-aminophenoxy)-N-methylpicolinamide **(c)** using general method 3.2.1 or from compound **7c** and 3-(4-methyl-1H-imidazol-1-yl)-5-(trifluoromethyl)aniline **(a)** using method 3.2.4.1 as a white solid. M.p. 153-156 °C. $^{1}$H-NMR (DMSO-$d_6$, 500 MHz) $\delta$: 10.68 (s, 1H), 8.76 (q, $J$ = 4.7 Hz, 1H), 8.47 (d, $J$ = 5.4 Hz, 1H), 8.31 (s, 1H), 8.23 (d, $J$ = 1.3 Hz, 1H), 8.17 (s, 1H), 8.00 (d, $J$ = 8.3 Hz, 1H), 7.74 (s, 1H), 7.60 (d, $J$ = 8.0 Hz, 1H), 7.50 (s, 1H), 7.33 (d, $J$ = 2.6 Hz, 1H), 7.09 (dd, $J$ = 5.8, 2.6 Hz, 1H), 6.95 (d, $J$ = 8.7 Hz, 1H), 6.69 (d, $J$ = 9.0 Hz, 1H), 6.59 (t, $J$ = 5.9 Hz, 1H), 4.43 (d, $J$ = 5.8 Hz, 1H), 2.79 (d, $J$ = 4.8 Hz, 3H), 2.20(s, 2H); $^{13}$C-NMR (DMSO-$d_6$, 126 MHz) $\delta$: 167.2, 166.4, 164.4, 152.8, 150.7, 147.1, 145.3, 143.6, 141.9, 139.4, 138.4, 135.4, 133.1, 128.4, 127.7, 122.2, 115.3, 114.7, 114.3, 113.8, 112.0, 108.7, 46.9, 26.5, 14.0. HRMS(ESI+) m/z calcd for $C_{32}H_{27}F_3N_6O_3$ [M + H]+ 601.2097, found 601.2167. Purity: 99.27% (by HPLC).

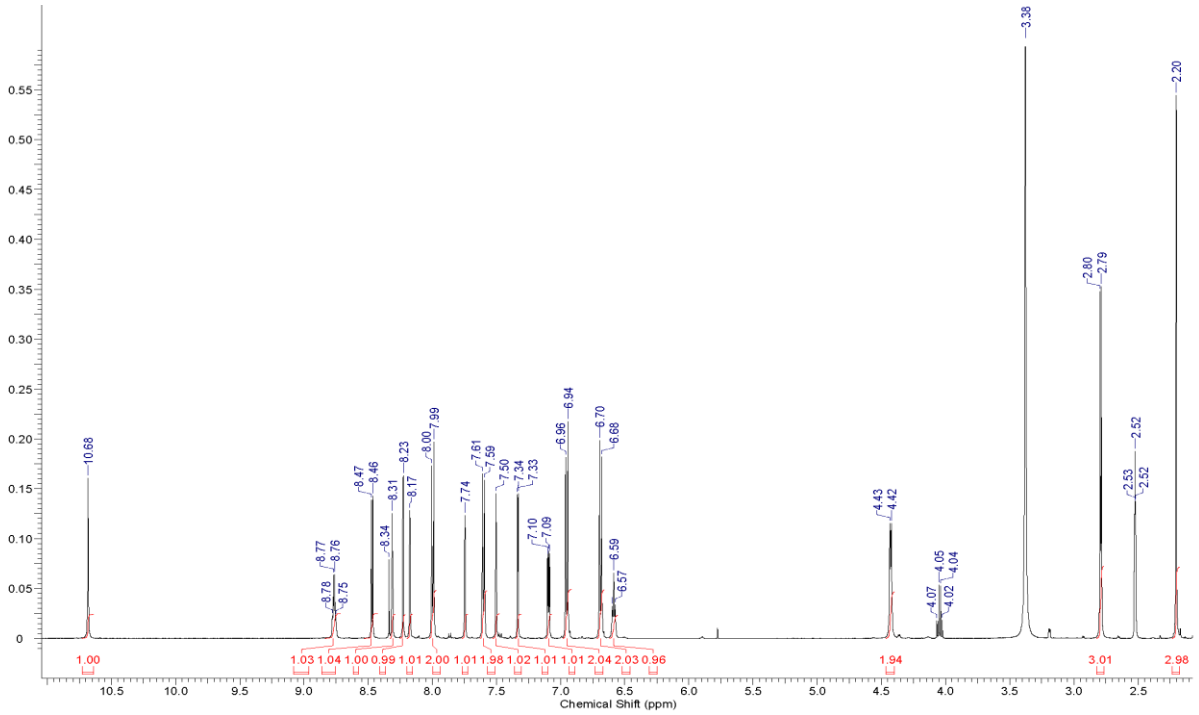

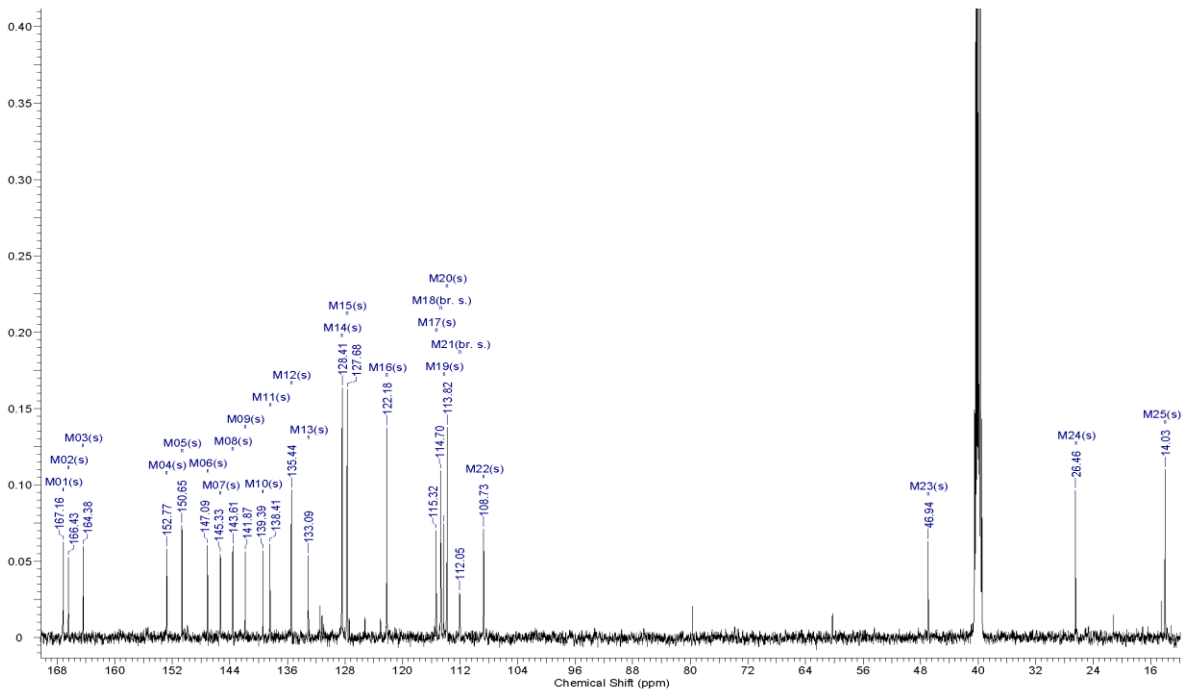


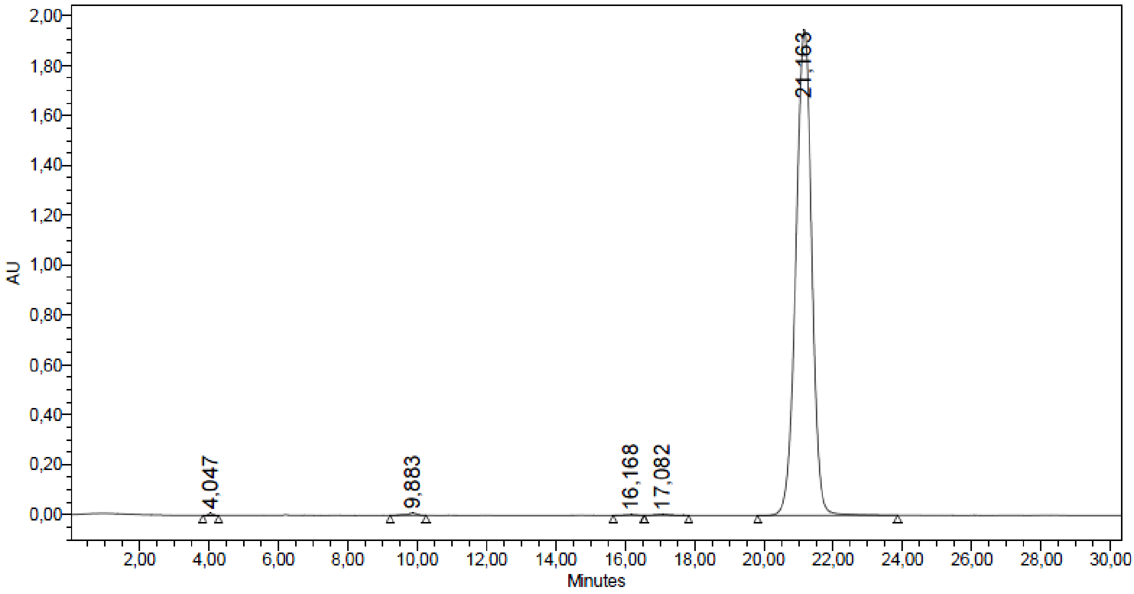


| | RT | Area | % Area | Height |
|---|---|---|---|---|
| 1 | 4,047 | 62665 | 0,10 | 7328 |
| 2 | 9,883 | 185512 | 0,30 | 8669 |
| 3 | 16,168 | 64841 | 0,10 | 2662 |
| 4 | 17,082 | 138640 | 0,22 | 5028 |
| 5 | 21,163 | 61823772 | 99,27 | 1945549 |

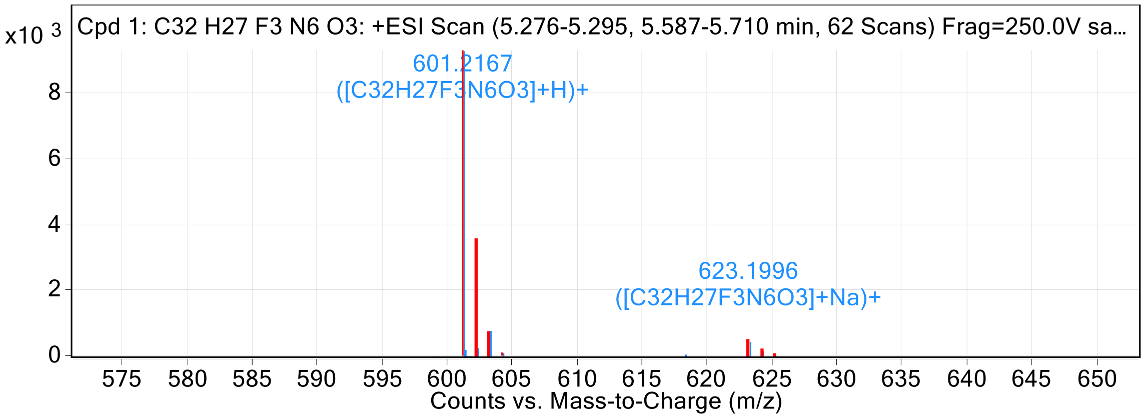
x10 3
Cpd 1: C32 H27 F3 N6 O3: +ESI Scan (5.276-5.295, 5.587-5.710 min, 62 Scans) Frag=250.0V sa...
601.2167
([C32H27F3N6O3]+H)+
623.1996
([C32H27F3N6O3]+Na)+
0
2
4
6
8
575 580 585 590 595 600 605 610 615 620 625 630 635 640 645 650
Counts vs. Mass-to-Charge (m/z)

***N*-(3-(4-methyl-1*H*-imidazol-1-yl)-5-(trifluoromethyl)phenyl)-4-(((3-(trifluoromethyl)phenyl)amino)methyl)benzamide** **(11)** was synthesized from compound **5** and 3-(trifluoromethyl)aniline **(g)** as a white solid. M.p. 83-85 °C. $^1$H-NMR (DMSO-$d_6$, 500 MHz) $\delta$: 10.67 (s, 1H), 8.31 (s, 1H), 8.16 (s, 1H), 7.98 (d, $J$ = 8.3 Hz, 1H), 7.75 (s, 1H), 7.57 (d, $J$ = 8.3 Hz, 1H), 7.27 (t, $J$ = 7.9 Hz, 1H), 6.91 (t, $J$ = 6.1 Hz, 1H), 6.88 (s, 1H), 6.84 (s, 1H), 6.83 (s, 1H), 4.46 (d, $J$ = 6.1 Hz, 1H), 2.21 (s, 3H); $^{13}$C-NMR (DMSO-$d_6$, 126 MHz) $\delta$: 166.4, 149.4, 144.8, 141.8, 138.4, 133.1, 130.4, 128.4, 127.7, 116.1, 115.4, 114.8, 112.4, 108.7, 46.3, 13.9. HRMS(ESI+) m/z calcd for $C_{26}H_{20}F_6N_4O$ [M + H]+ 519.1541, found 519.1613. Purity: 97.02% (by HPLC).

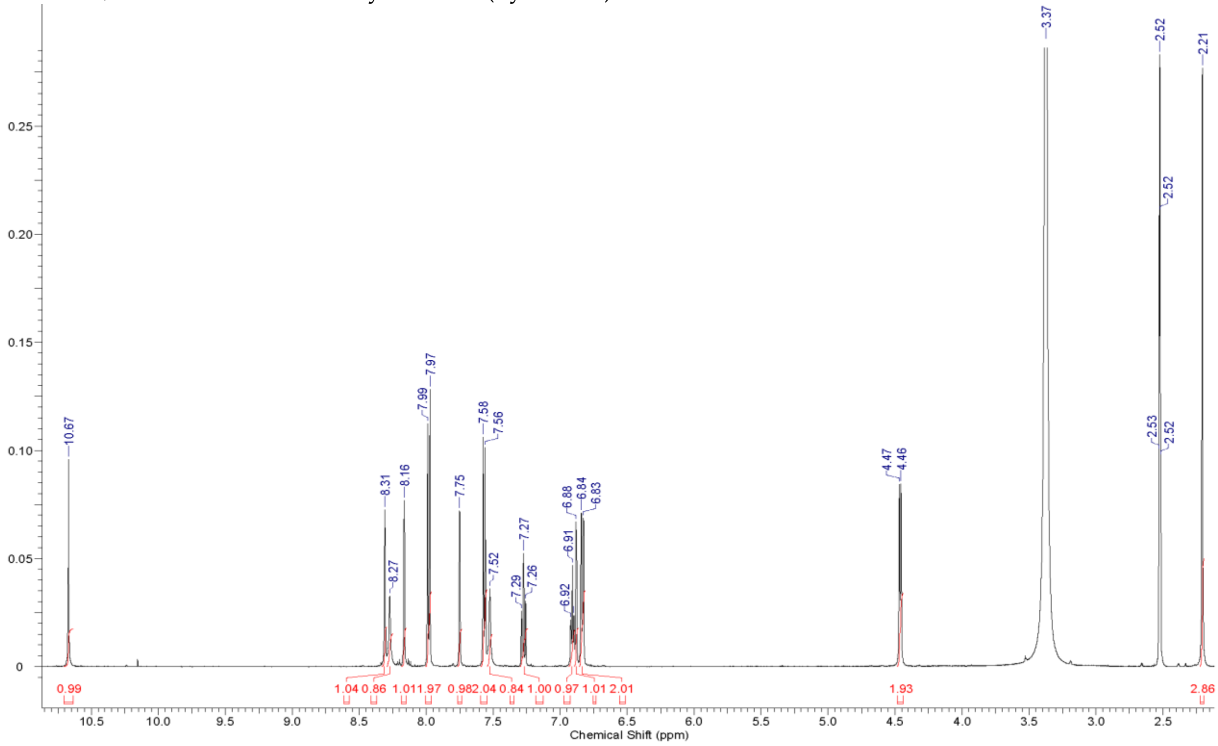


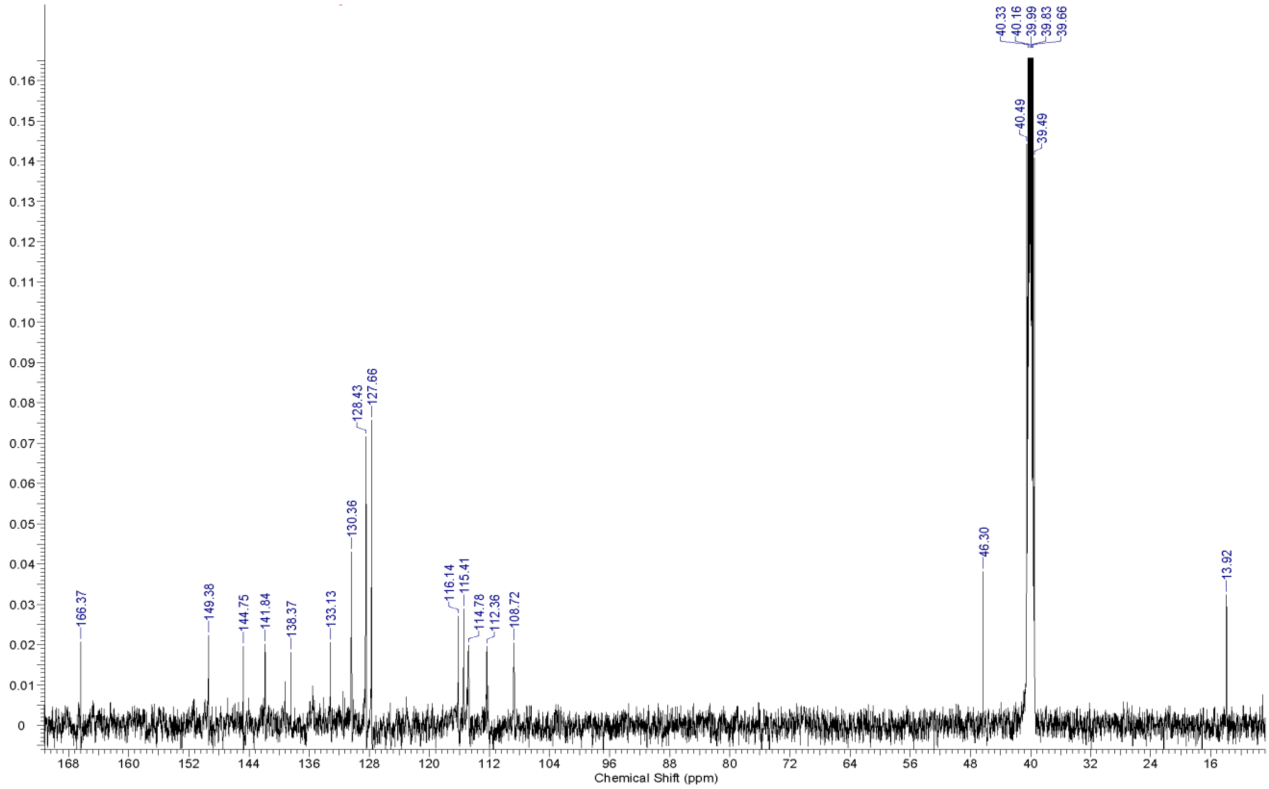

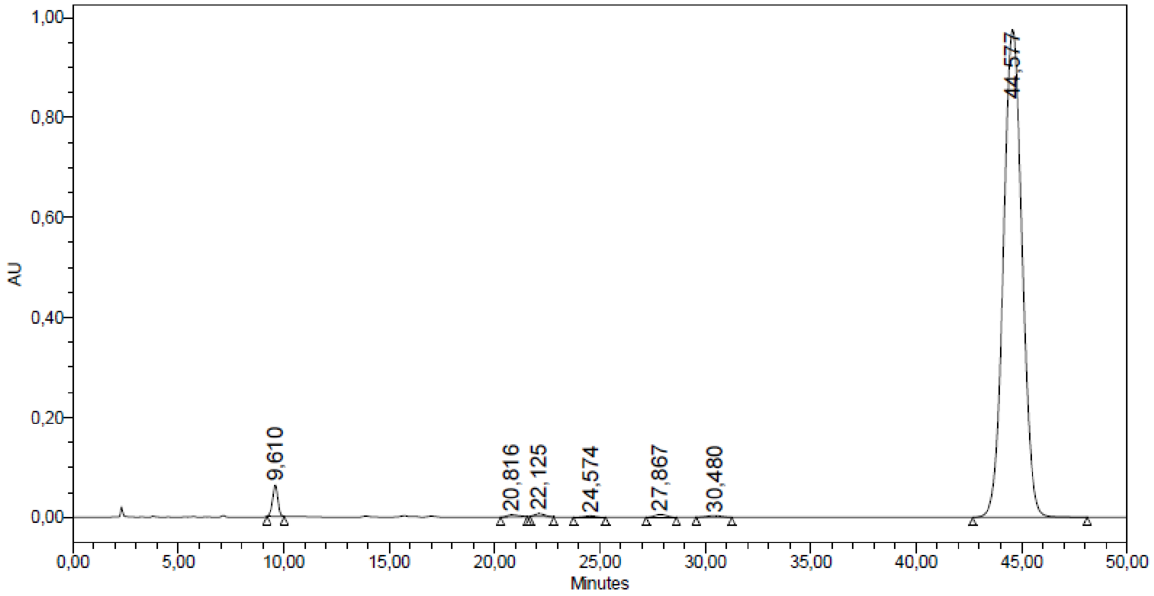


| | RT | Area | % Area | Height |
|---|---|---|---|---|
| 1 | 9,610 | 1078934 | 1,74 | 62444 |
| 2 | 20,816 | 149447 | 0,24 | 4165 |
| 3 | 22,125 | 183407 | 0,30 | 5566 |
| 4 | 24,574 | 87497 | 0,14 | 2361 |
| 5 | 27,867 | 207145 | 0,33 | 5444 |
| 6 | 30,480 | 139500 | 0,23 | 2901 |
| 7 | 44,577 | 60125805 | 97,02 | 975129 |

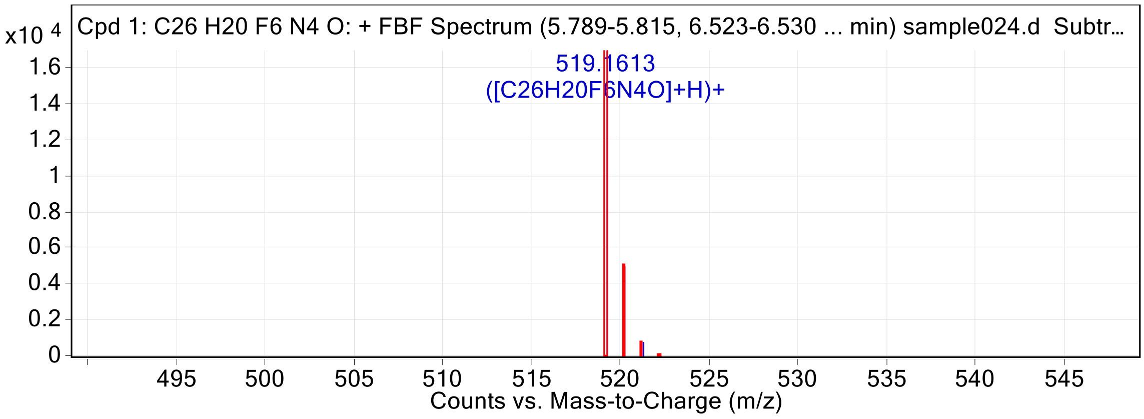

***Methyl-4-amino-3-((4-((3-(4-methyl-1H-imidazol-1-yl)-5-(trifluoromethyl)phenyl)carbamoyl)benzyl)amino)benzoate* (12)** was synthesized from compound **5** and 3,4-diaminobenzoic acid **(e)** as a brown solid. M.p. 130-132 °C. $^{1}$H-NMR (DMSO-*d*$_6$, 500 MHz) δ: 11.88 (br.s, 1H), 10.68 (s, 1H), 8.34 (s, 1H), 8.31 (s, 1H), 8.25 (d, *J* = 1.3 Hz, 1H), 8.16 (s, 1H), 7.98 (d, *J* = 8.3 Hz, 2H), 7.75 (s, 1H), 7.57 (d, *J* = 8.3 Hz, 2H), 7.51 (s, 1H), 7.14 (dd, *J* = 8.2, 1.8 Hz, 1H), 6.90 (d, *J* = 1.9 Hz, 1H), 6.59 (d, *J* = 8.0 Hz, 1H), 5.47 (br.s., 1H), 4.47 (d, *J* = 1.0 Hz, 2H), 2.20 (s, 3H), 2.11 (s, 1H); $^{13}$C-NMR (DMSO-*d*$_6$, 126 MHz) δ: 168.4, 166.5, 145.3, 141.9, 141.2, 139.2, 138.4, 135.4, 134.3, 133.1, 128.3, 127.5, 121.1, 118.8, 115.4, 114.7, 112.8, 112.1, 111.5, 79.7, 47.0, 14.0. HRMS(ESI+) m/z calcd for $C_{26}H_{22}F_3N_5O_3$ $[M + H]^+$ 510.1675, found 510.1752.

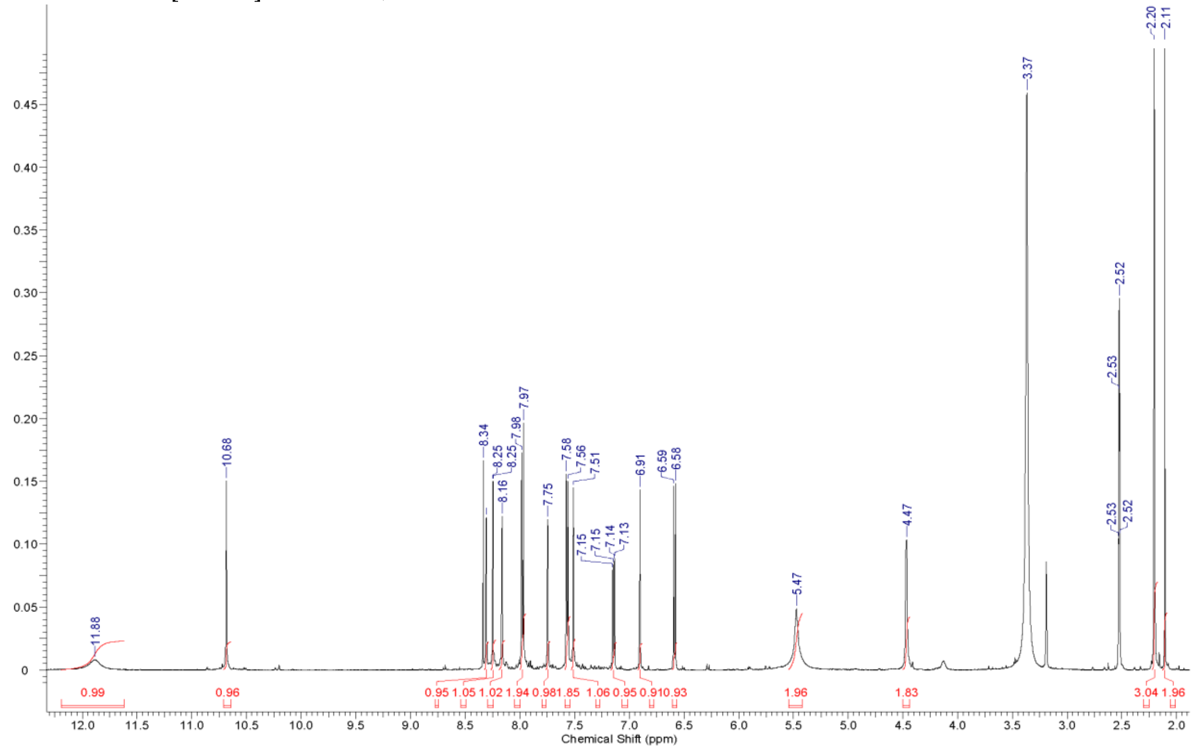


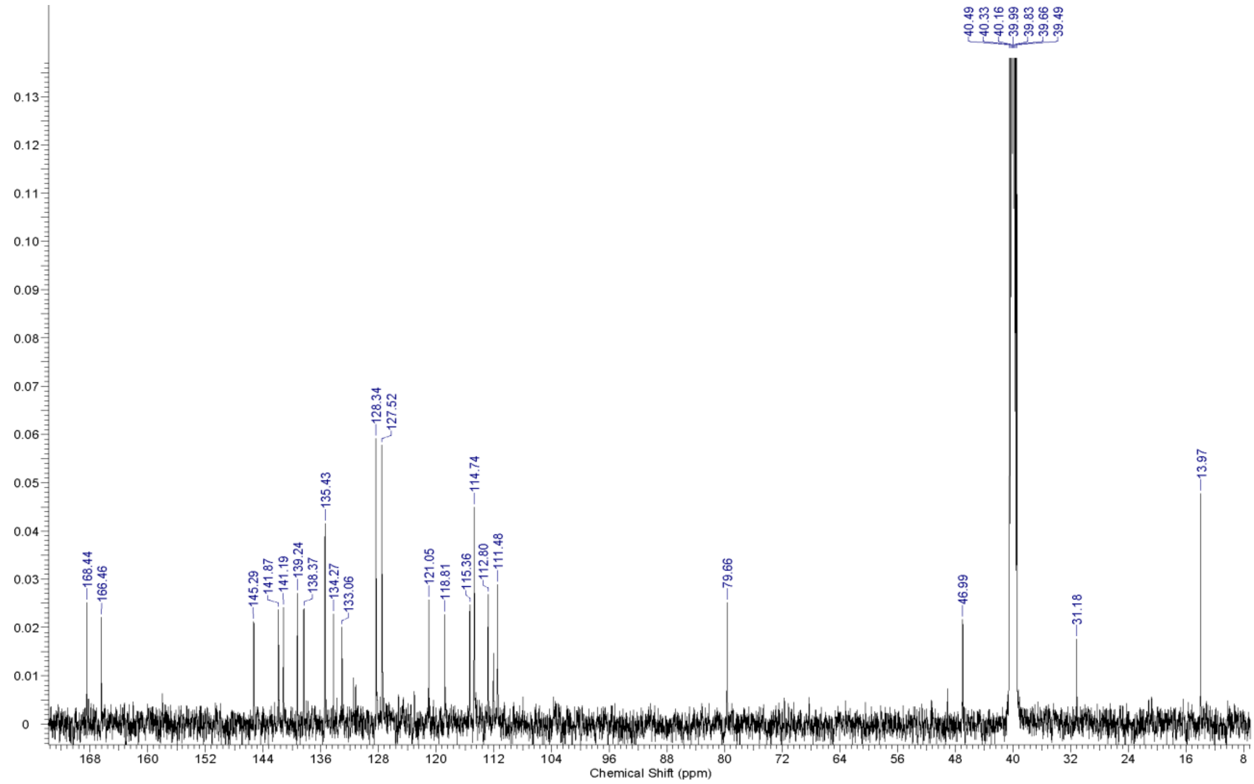

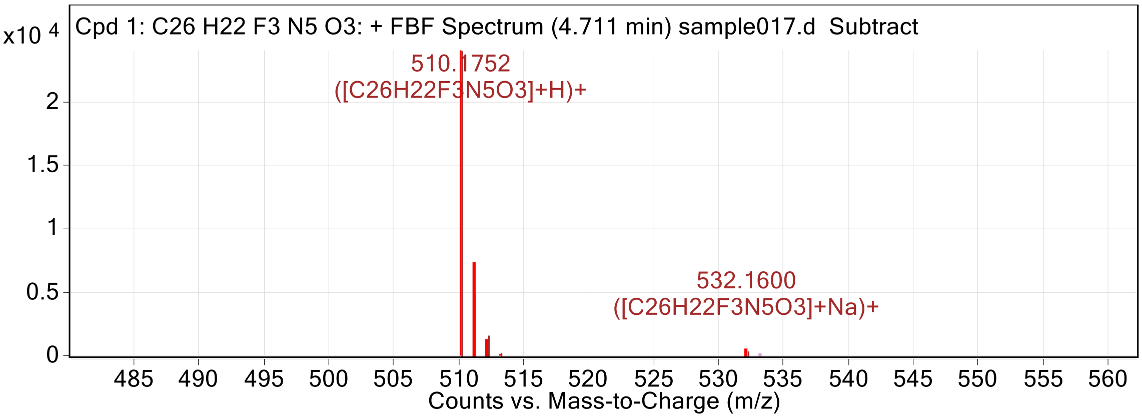
Cpd 1: C26 H22 F3 N5 O3: + FBF Spectrum (4.711 min) sample017.d Subtract
x10 4
510.1752
([C26H22F3N5O3]+H)+
532.1600
([C26H22F3N5O3]+Na)+
Counts vs. Mass-to-Charge (m/z)

***4-({[3-(4-methyl-1H-imidazol-1-yl)-5-(trifluoromethyl)phenyl]amino}methyl)-N-[3-(trifluoromethyl)phenyl]benzamide*** **(13)** was synthesized from compound **7a** and 3-(trifluoromethyl)aniline **(g)** as a white solid. M.p. 129-131 °C. $^{1}$H-NMR (DMSO-*d*$_6$, 500 MHz) $\delta$: 10.59 (s, 1H), 8.84 (br.s., 1H), 8.28 (s, 1H), 8.08 (d, *J* = 8.3 Hz, 1H), 8.00 (d, *J* = 8.0 Hz, 2H), 7.72 (s, 1 H), 7.59 - 7.63 (m, 1H), 7.57 (d, *J* = 8.3 Hz, 2H), 7.46 (d, *J* = 7.7 Hz, 1H), 7.33 (t, *J* = 6.1 Hz, 1H), 7.14 (s, 1H), 7.08 (s, 1H), 7.00 (s, 1H), 4.52 - 4.60 (m, 2H), 2.25 (s, 3H); $^{13}$C-NMR (DMSO-*d*$_6$, 126 MHz) $\delta$: 166.2, 150.8, 143.8, 140.5, 138.2, 134.9, 133.6, 130.3, 128.5, 127.7, 124.2, 120.3, 116.8, 116.8, 116.1, 108.8, 106.9, 104.5, 60.2, 46.1, 14.6, 12.3. HRMS(ESI+) m/z calcd for $C_{26}H_{20}F_6N_4O$ [M + H]+ 519.1541, found 519.1611. Purity: 97.11% (by HPLC).

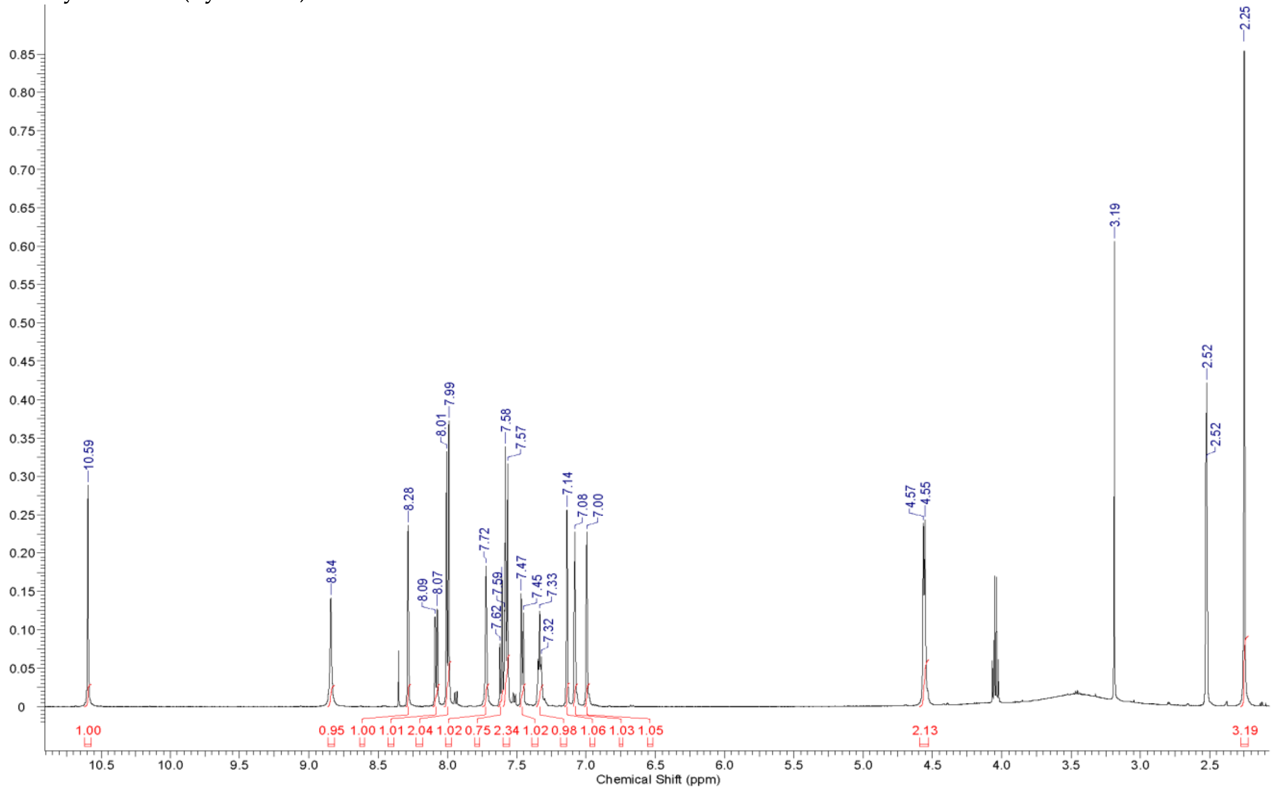

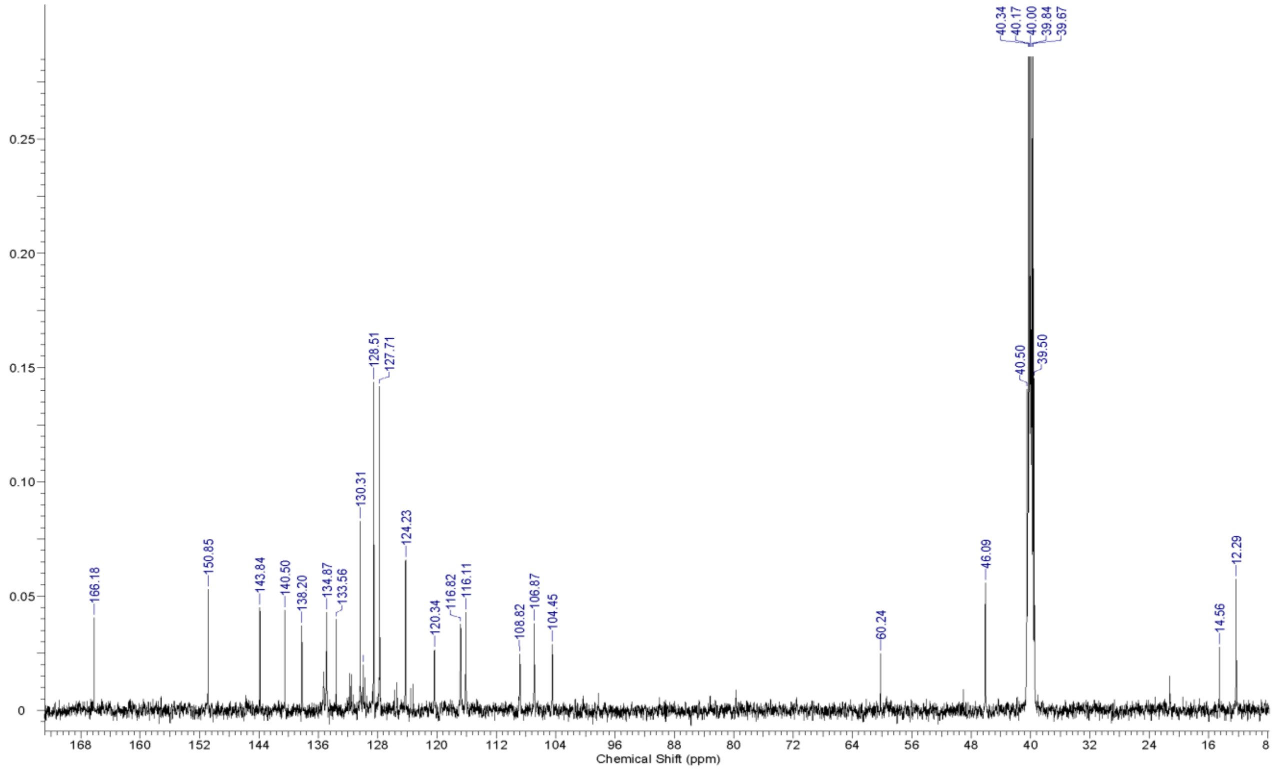


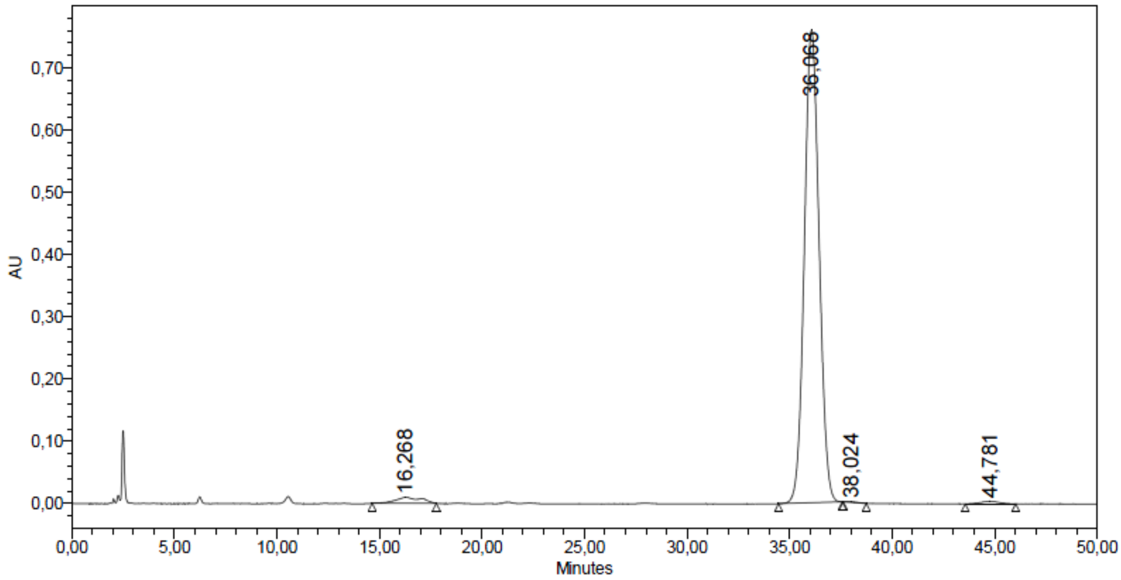


| | RT | Area | % Area | Height |
|---|---|---|---|---|
| 1 | 16,268 | 843545 | 2,16 | 9688 |
| 2 | 36,068 | 37919342 | 97,11 | 759499 |
| 3 | 38,024 | 33759 | 0,09 | 1053 |
| 4 | 44,781 | 250772 | 0,64 | 4152 |

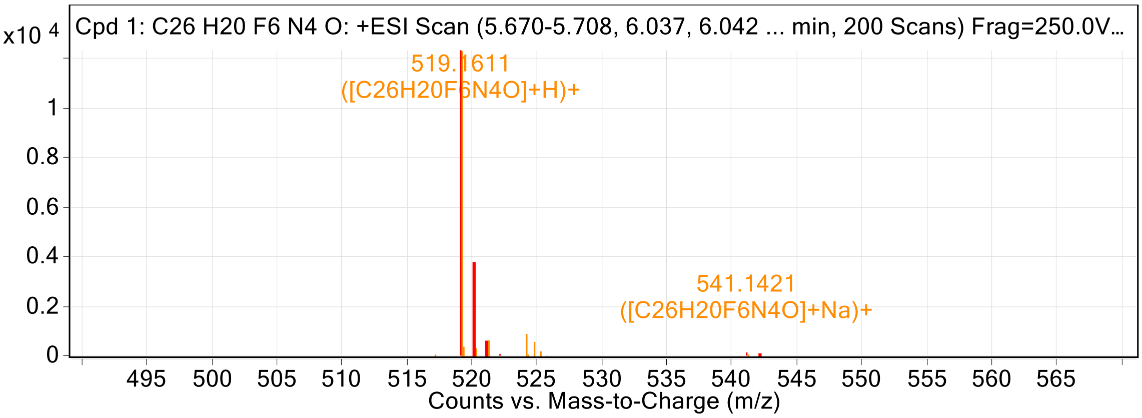

Cpd 1: C26 H20 F6 N4 O: +ESI Scan (5.670-5.708, 6.037, 6.042 ... min, 200 Scans) Frag=250.0V...
x10 4
519.1611
([C26H20F6N4O]+H)+
541.1421
([C26H20F6N4O]+Na)+
Counts vs. Mass-to-Charge (m/z)

***Diethyl(4-(((3-(4-methyl-1H-imidazol-1-yl)-5-(trifluoromethyl)phenyl)amino)methyl)benzoyl)glutamate* (14)** was synthesized from compound **7a** and diethyl glutamate **(h)** as a white solid. M.p. 125-127 °C. $^1$H-NMR (DMSO-$d_6$, 500 MHz) δ: 8.70 (d, J = 7.4 Hz, 1H), 8.15 (br.s., 1H), 7.86 (d, *J* = 8.3 Hz, 2H), 7.51 (d, *J* = 8.0 Hz, 2H), 7.45 (br.s., 1H), 7.12 (t, *J* = 5.9 Hz, 1H), 7.04 (s, 1H), 6.97 (s, 1H), 6.87 (s, 1H), 4.51 (d, *J* = 5.8 Hz, 2H), 4.44 (ddd, *J* = 9.4, 7.3, 5.4 Hz, 1H), 4.08 - 4.17 (m, 2H), 4.05 (q, *J* = 7.1 Hz, 2H), 2.45 (t, *J* = 7.7 Hz, 2H), 2.16 (s, 3H), 1.94 - 2.14 (m, 2H), 1.12 - 1.24 (m, 6H); $^{13}$C-NMR (DMSO-$d_6$ , 126 MHz) δ: 172.7, 172.3, 167.0, 150.7, 143.5, 139.0, 132.9, 128.2, 127.6, 107.6, 106.2, 103.8, 61.0, 60.4, 52.5, 46.2, 30.6, 26.2, 14.5, 14.1. HRMS(ESI+) m/z calcd for $C_{28}H_{31}F_3N_4O_5$ [M + H]+ 561.2247, found 561.2321. Purity: 97.13% (by HPLC).

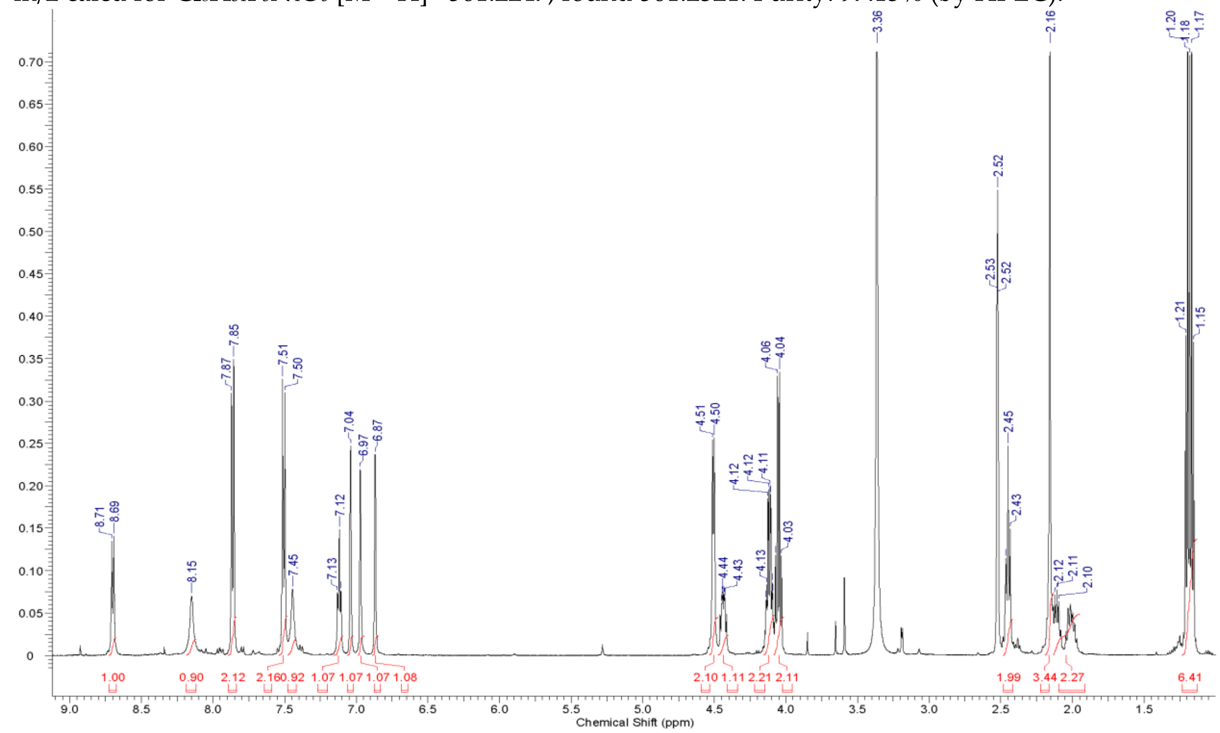


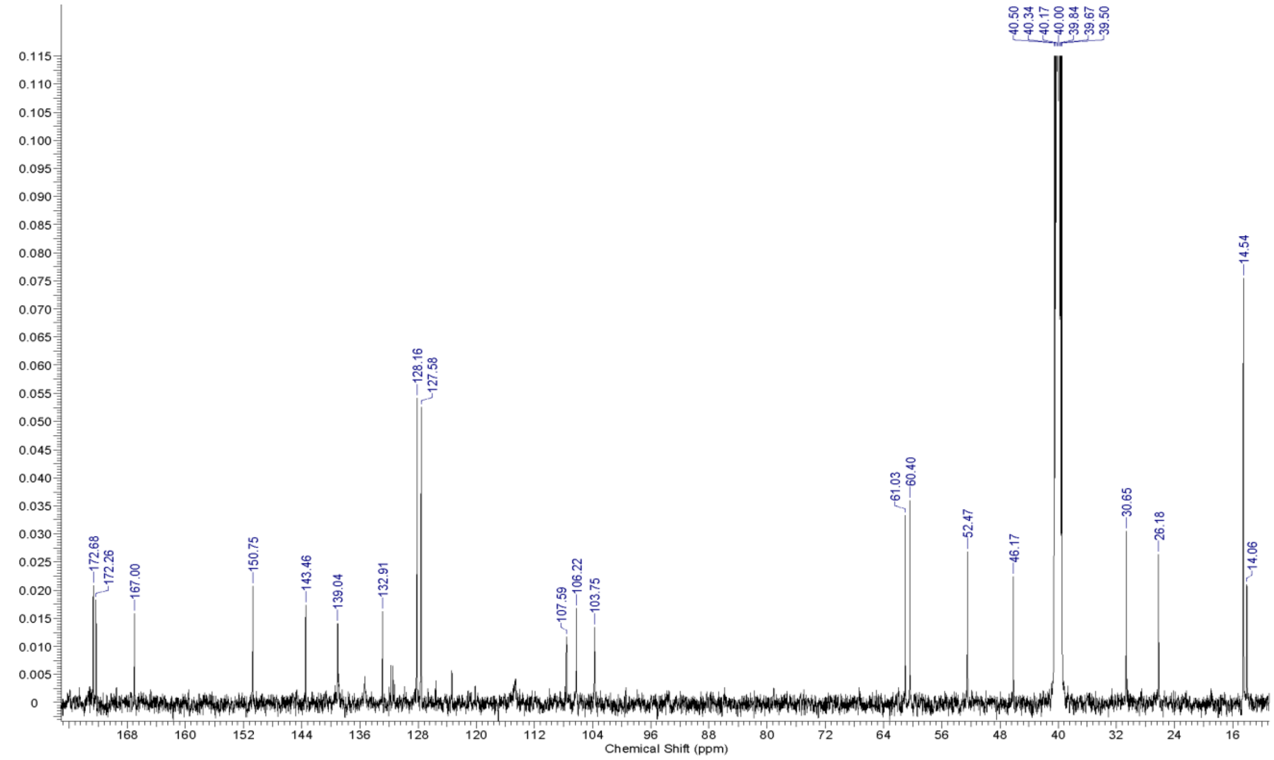

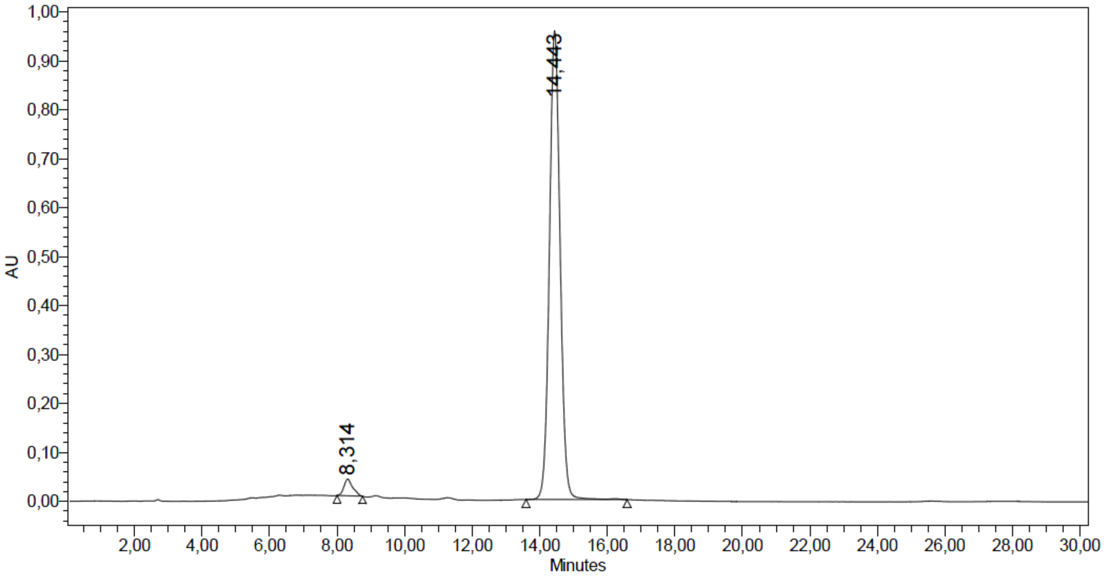


| | RT | Area | % Area | Height |
|---|---|---|---|---|
| 1 | 8,314 | 623818 | 2,87 | 34228 |
| 2 | 14,443 | 21084994 | 97,13 | 957457 |

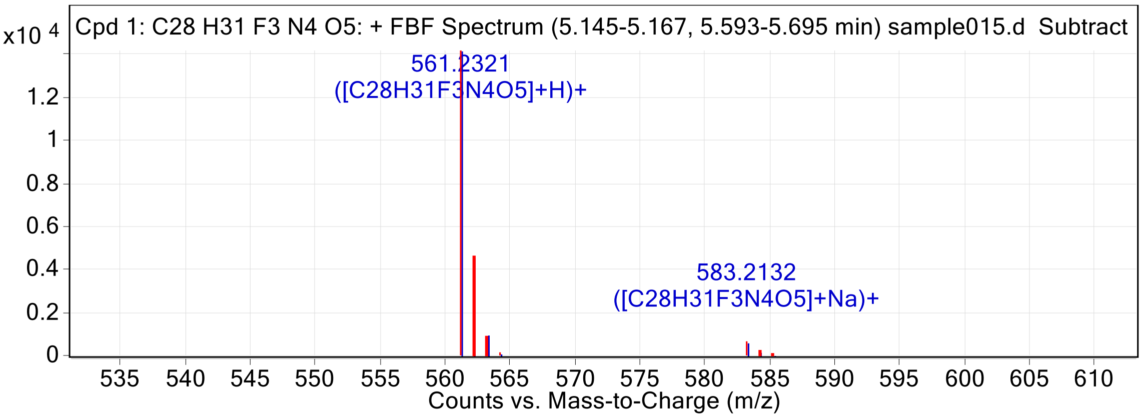

***4-({[3-(4-methyl-1H-imidazol-1-yl)-5-(trifluoromethyl)phenyl]amino}methyl)-N-{[(2-methyl-5-nitrophenyl) benzoate*** **(15)** was synthesized from compound **7a** and 2-methyl-5-nitroaniline **(b)** as a white solid. M.p. 205-208 °C. $^1$H-NMR (DMSO-$d_6$, 500 MHz) $\delta$: 10.11 (s, 1H), 8.37 (d, $J$ = 2.2 Hz, 1H), 8.23 (s, 1H), 8.04 (dd, $J$ = 8.5, 2.4 Hz, 1H), 7.99 (d, $J$ = 8.3 Hz, 2H), 7.56 - 7.60 (m, 3H), 7.48 (s, 1H), 7.19 (t, $J$ = 6.3 Hz, 1H), 7.06 (s, 1H), 6.99 (s, 1H), 6.91 (s, 1H), 4.56 (d, $J$ = 6.1 Hz, 2H), 2.40 (s, 3H), 2.17 (s, 3H); $^{13}$C-NMR (DMSO-$d_6$, 126 MHz) $\delta$: 166.0, 150.7, 146.2, 144.0, 142.0, 138.9, 138.5, 137.9, 135.3, 133.2, 132.0, 128.6, 127.7, 120.8, 120.7, 114.8, 107.8, 106.2, 103.8, 46.1, 18.7, 13.9. HRMS(ESI+) m/z calcd for $C_{26}H_{22}F_3N_5O_3$ [M + H]+ 510.1675, found 510.1745. Purity: 99.06% (by HPLC).

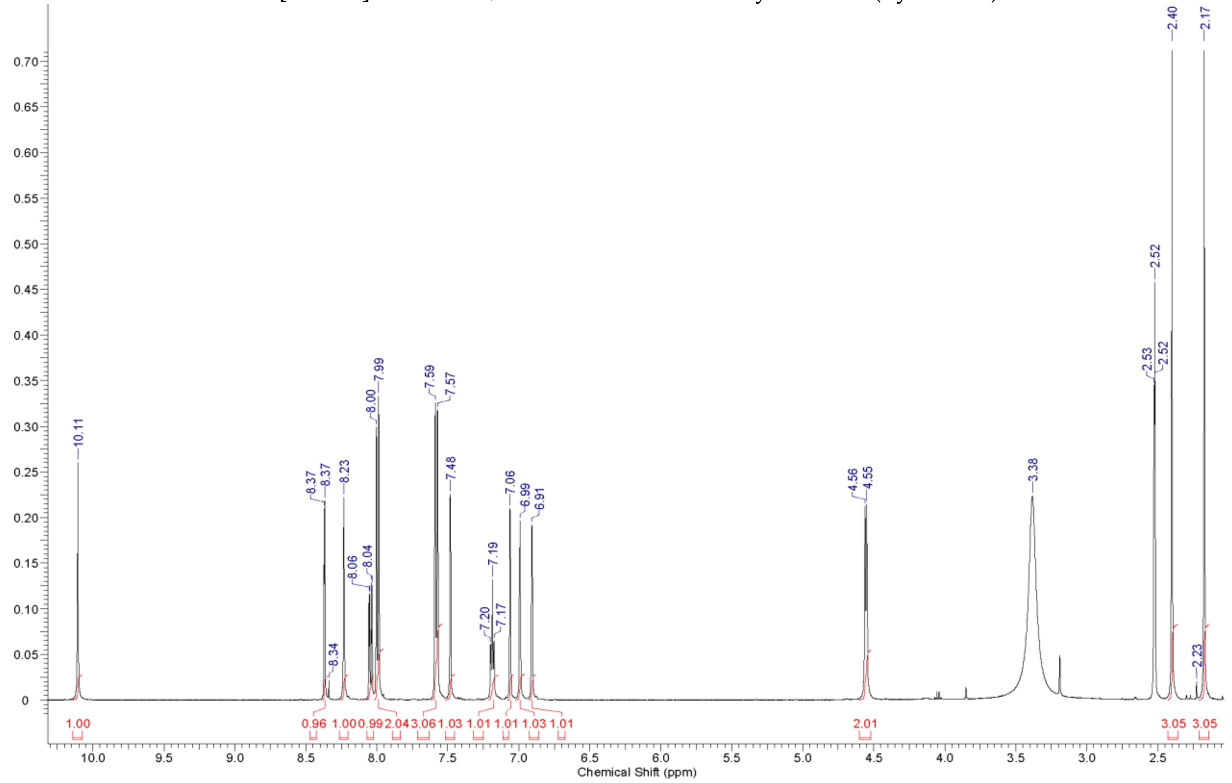


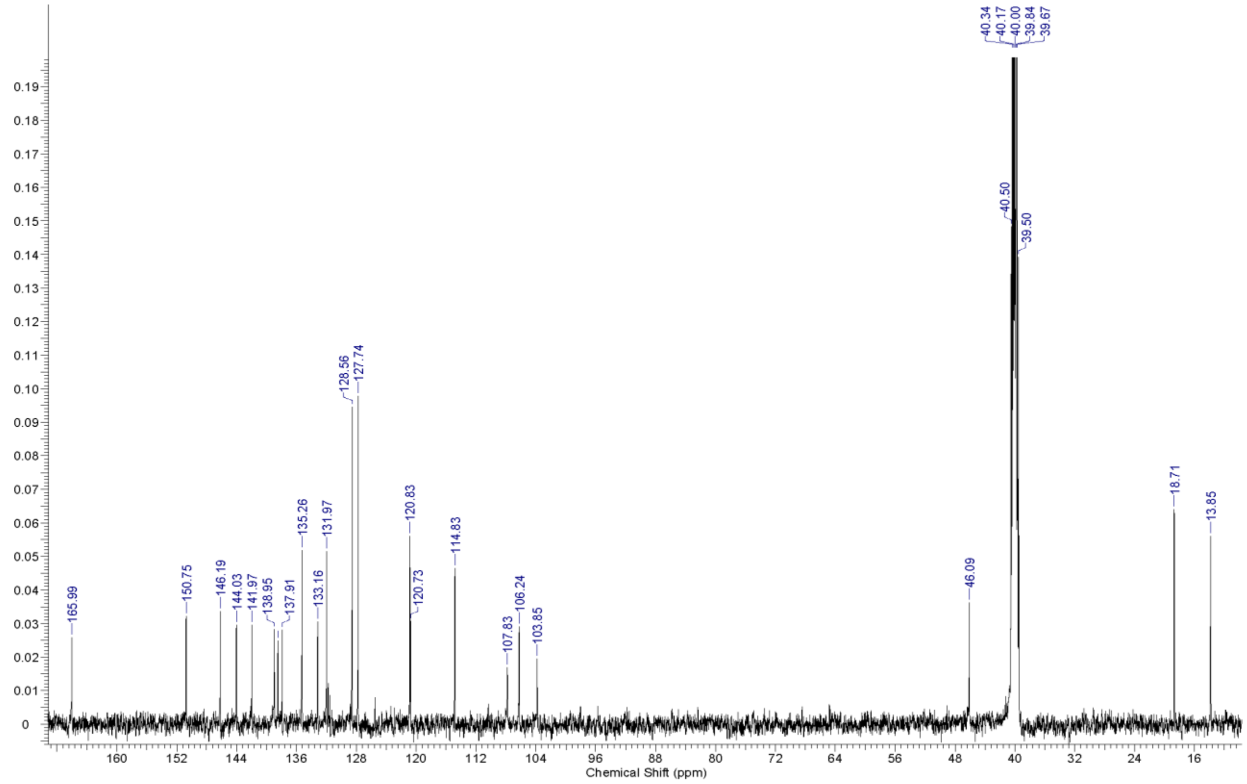

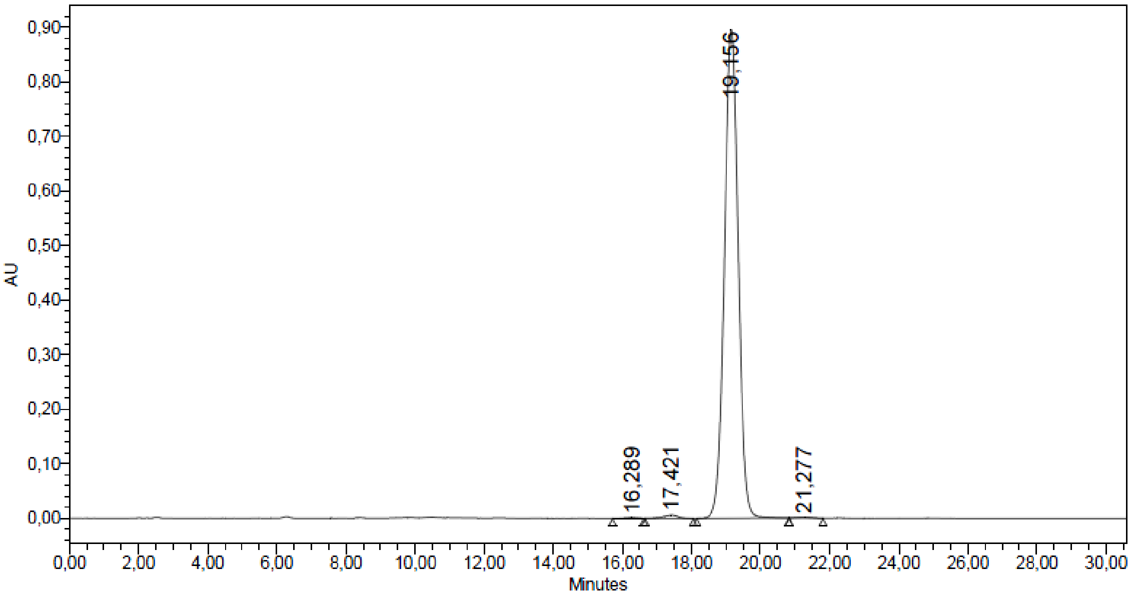


| | RT | Area | % Area | Height |
|---|---|---|---|---|
| 1 | 16,289 | 30395 | 0,12 | 1275 |
| 2 | 17,421 | 164130 | 0,66 | 5863 |
| 3 | 19,156 | 24789567 | 99,06 | 894547 |
| 4 | 21,277 | 41293 | 0,17 | 1500 |

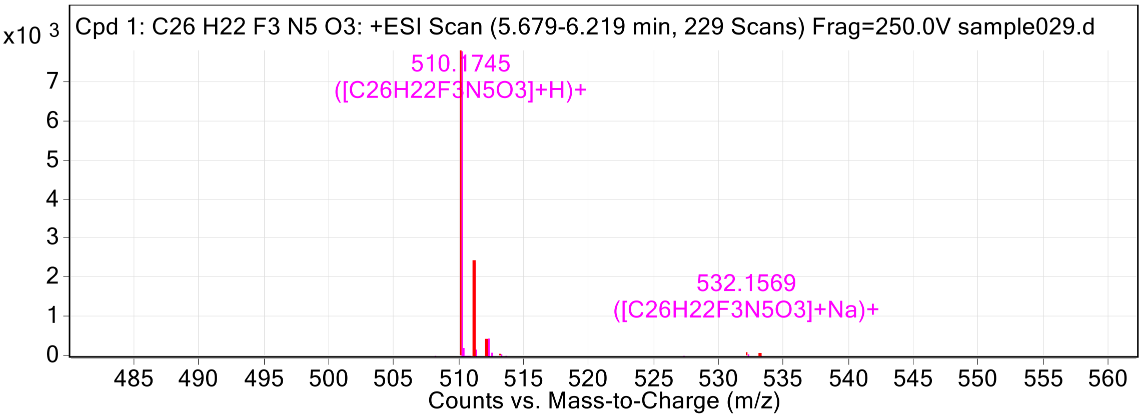

***Diethyl (4-(((2-methyl-5-nitrophenyl)amino)methyl)benzoyl)glutamate*** **(16)** was synthesized from compound **7b** and diethyl glutamate **(h)** as a yellow solid. M.p. 127-129 °C. $^{1}$H-NMR (DMSO-*d*$_6$ , 500 MHz) $\delta$: 8.70 (d, *J* = 7.7 Hz, 1H), 7.85 (d, *J* = 8.3 Hz, 2H), 7.50 (d, *J* = 8.3 Hz, 2H), 7.37 (dd, *J* = 8.0, 2.2 Hz, 1H), 7.26 (d, *J* = 8.3 Hz, 1H), 7.07 (d, *J* = 2.2 Hz, 1H), 6.49 (t, *J* = 6.1 Hz, 1H), 4.54 (d, *J* = 6.1 Hz, 2H), 4.44 (ddd, *J* = 9.5, 7.5, 5.4 Hz, 1H), 4.12 (qd, *J* = 7.1, 2.2 Hz, 2H), 4.05 (q, *J* = 7.1 Hz, 2H), 2.43 - 2.48 (m, 2H), 2.31 (s, 3H), 2.07 - 2.16 (m, 1H), 2.01 (dd, *J* = 9.6, 7.4 Hz, 1H), 1.18 (dt, *J* = 15.4, 7.2 Hz, 6H); $^{13}$C-NMR (DMSO-*d*$_6$, 126 MHz) $\delta$: 172.7, 172.3, 167.1, 147.4, 147.3, 143.6, 132.9, 130.9, 130.7, 128.2, 127.1, 111.0, 103.2, 79.7, 61.0, 60.4, 52.5, 46.3, 30.6, 26.2, 18.5, 14.5. HRMS(ESI+) m/z calcd for $C_{24}H_{29}N_3O_7$ [M + H]+ 472.2006, found 472.2080. Purity: 97.32% (by HPLC).

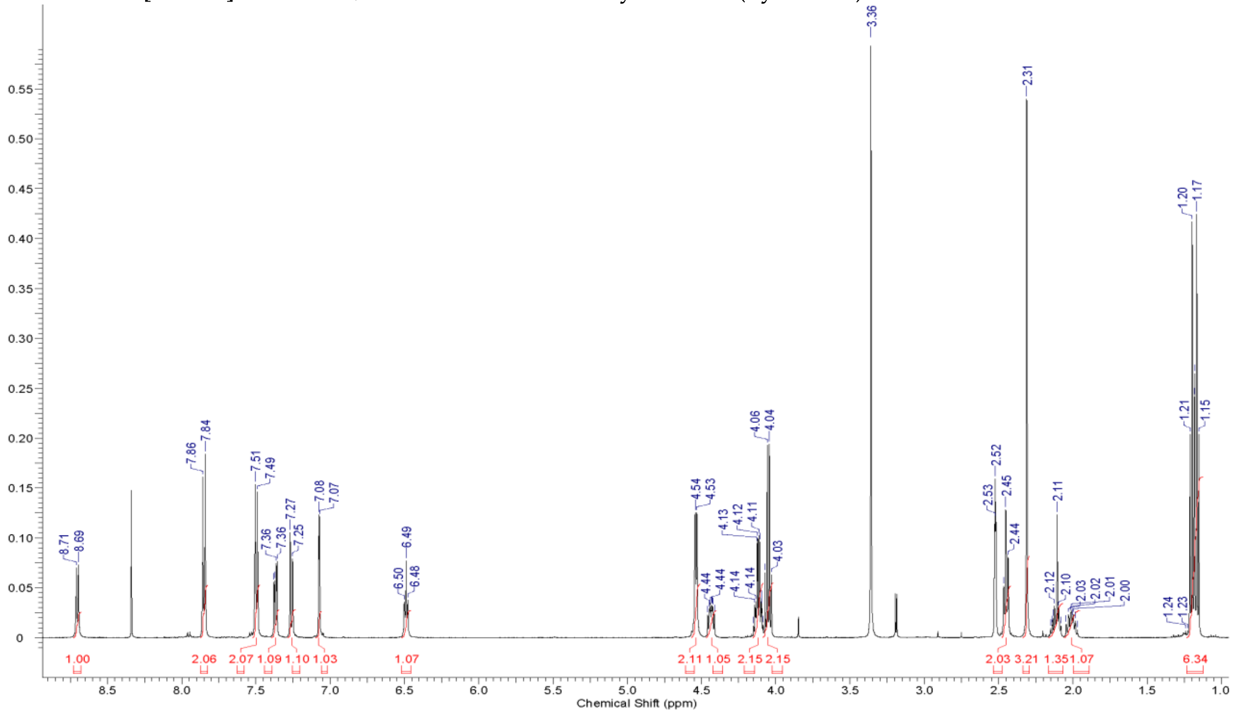


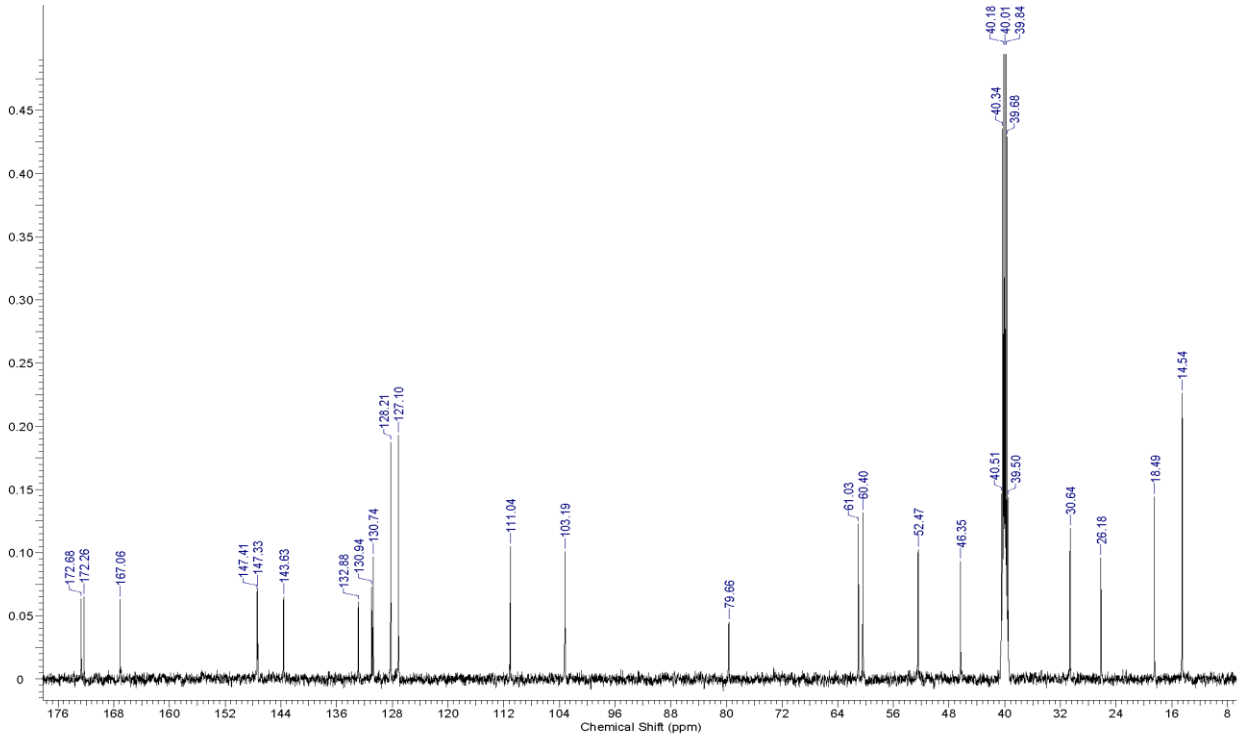

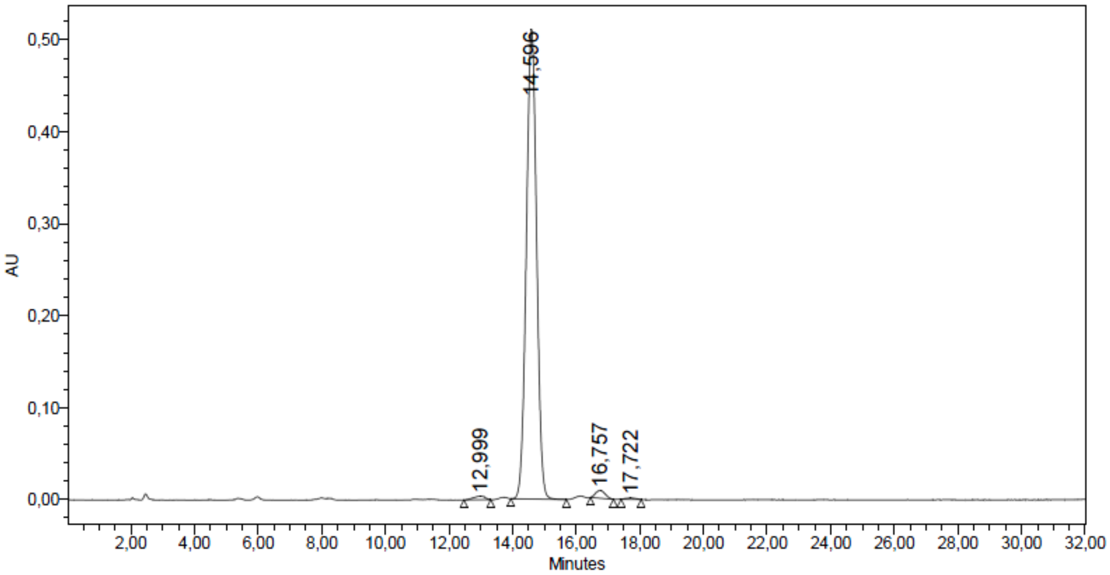


| | RT | Area | % Area | Height |
|---|---|---|---|---|
| 1 | 12,999 | 102051 | 0,87 | 4109 |
| 2 | 14,596 | 11423382 | 97,32 | 510404 |
| 3 | 16,757 | 178011 | 1,52 | 8494 |
| 4 | 17,722 | 34177 | 0,29 | 1636 |

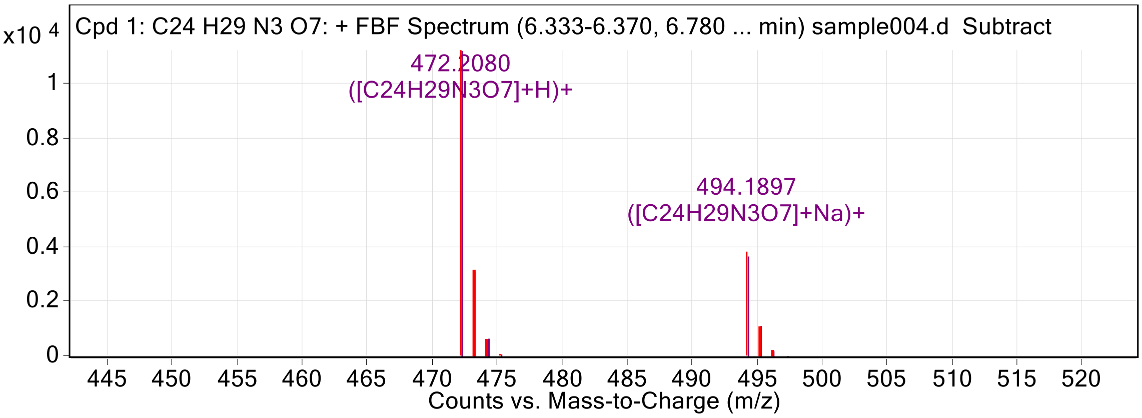

***4-{[(2-methyl-5-nitrophenyl)amino]methyl}-N-(4-{[2-(methylcarbamoyl)pyridin-4-yl]oxy}phenyl) benzoate* (17)** was synthesized from compound **7b** and 4-(4-aminophenoxy)-N-methylpicolinamide **(c)** as a yellow solid. M.p. 231-233 °C. $^1$H-NMR (DMSO-*d*$_6$ ,500 MHz) δ: 10.38 (br.s, 1H), 8.80 (br.s, 1H), 8.53 (br.s, 1H), 7.93 (br.s, 4H), 7.56 (br.s, 2H), 7.41 (br.s, 2H), 7.04 - 7.32 (m, 5H), 6.53 (br.s, 1H), 4.58 (br.s, 2H), 2.81 (br.s, 3H), 2.33 (br.s, 3H); $^{13}$C-NMR (DMSO-*d*$_6$ ,126 MHz) δ: 166.4, 165.9, 164.3, 153.1, 150.9, 149.1, 147.4, 147.3, 144.0, 137.7, 133.9, 131.0, 130.8, 128.4, 127.2, 122.6, 121.7, 114.3, 111.1, 109.2, 103.3, 46.2, 26.5, 18.5. HRMS(ESI+) m/z calcd for $C_{28}H_{25}N_5O_5$ [M + H]+ 512.1856, found 512.1926. Purity: 95.23% (by HPLC).

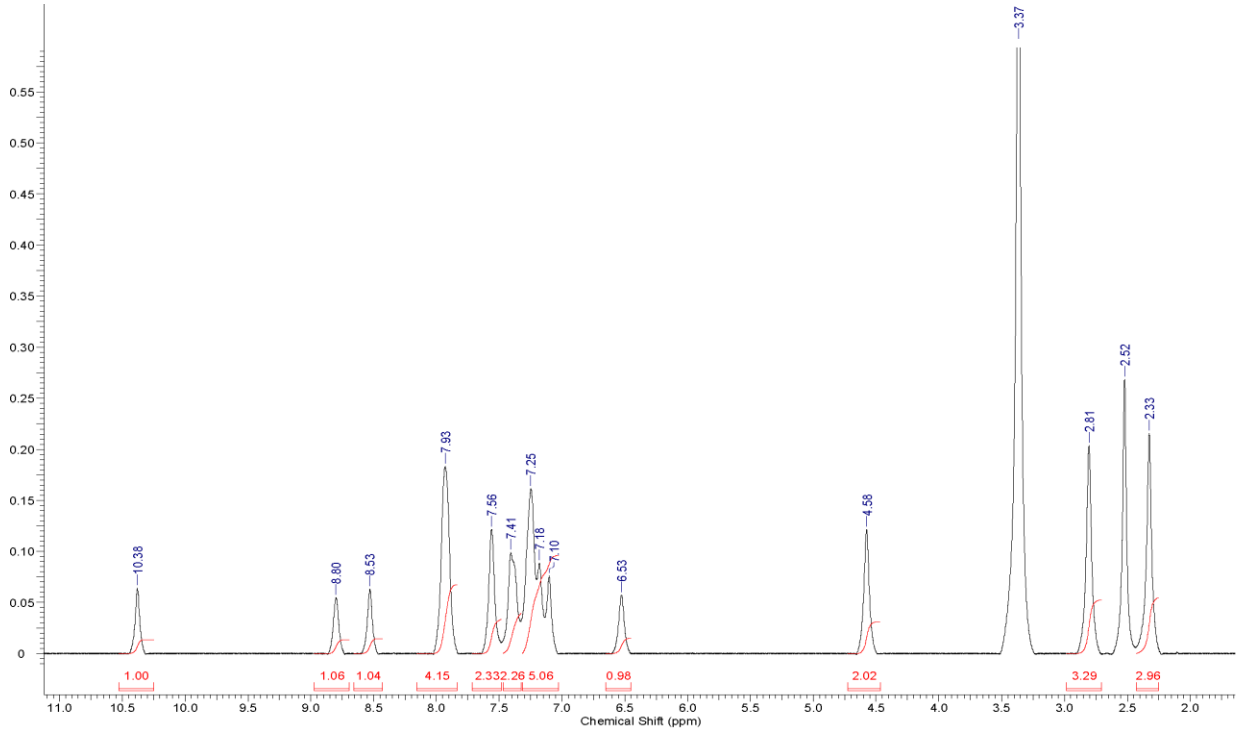


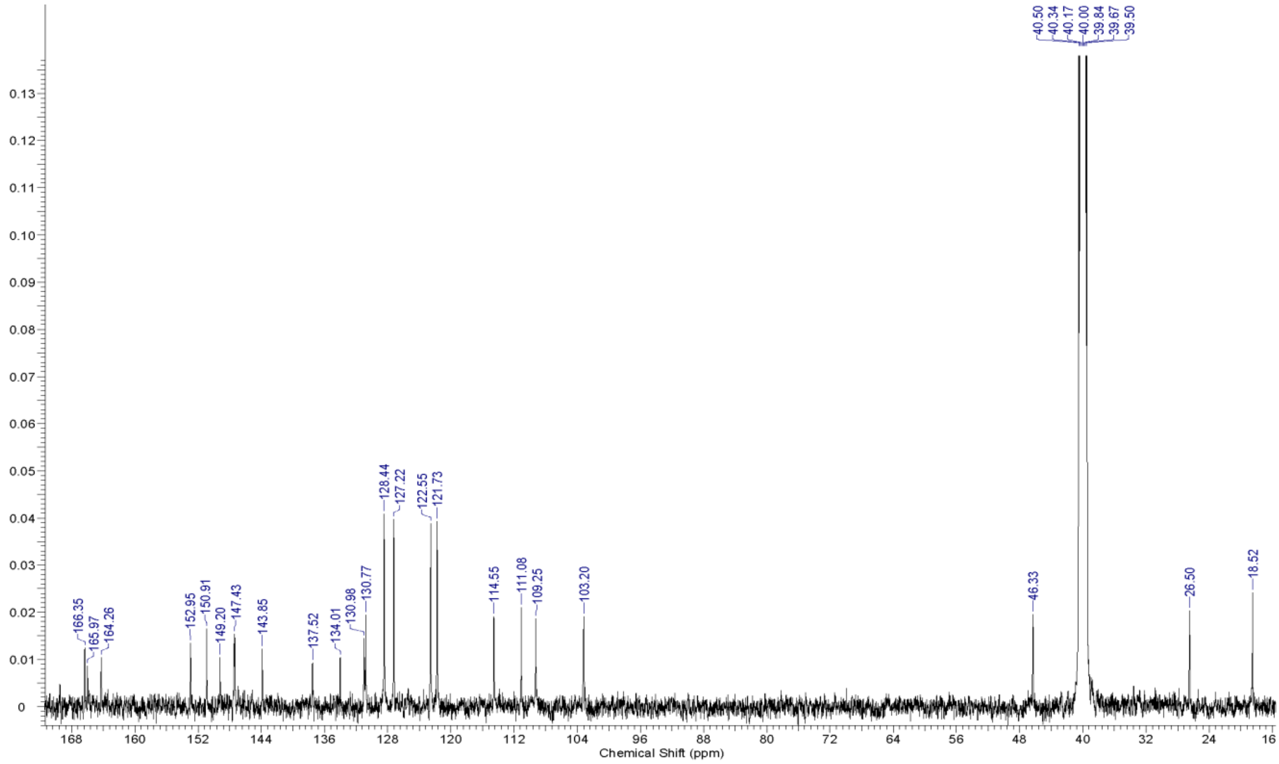

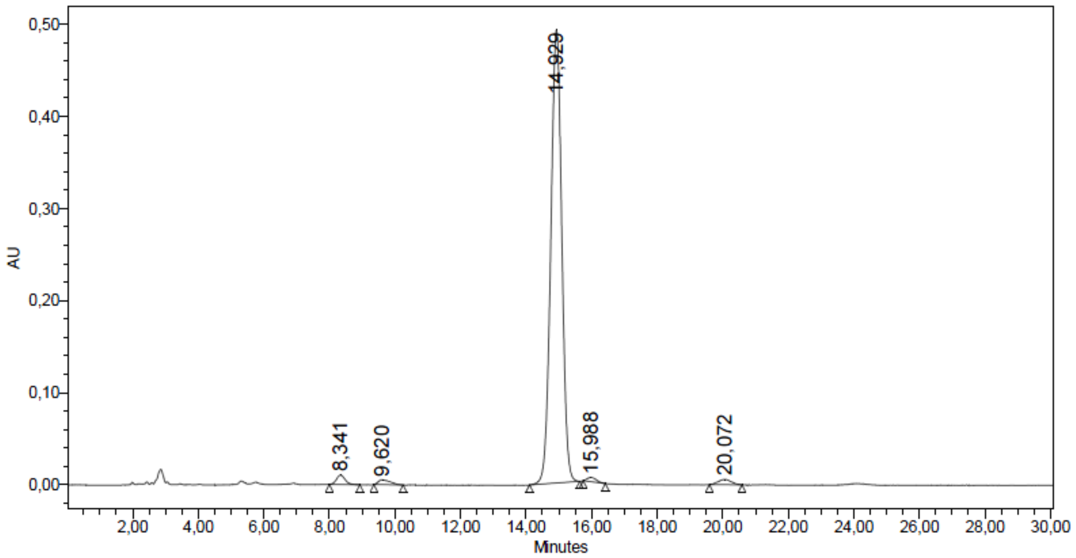


| | RT | Area | % Area | Height |
|---|---|---|---|---|
| 1 | 8,341 | 186730 | 1,56 | 10220 |
| 2 | 9,620 | 132509 | 1,11 | 5334 |
| 3 | 14,929 | 11410780 | 95,23 | 492157 |
| 4 | 15,988 | 90402 | 0,75 | 4627 |
| 5 | 20,072 | 161732 | 1,35 | 5706 |

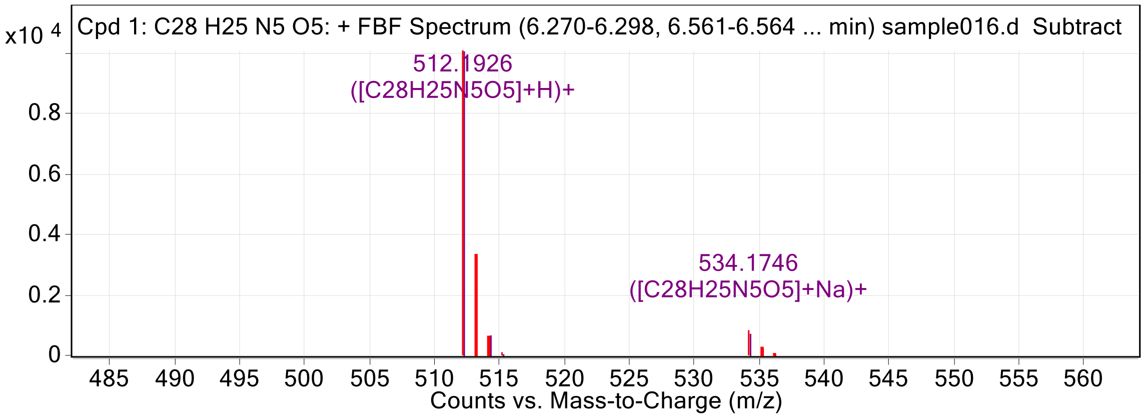

*4-{[(2-methyl-5-nitrophenyl)amino]methyl}-N-[3-(trifluoromethyl)phenyl]benzamide* **(18)** was synthesized from compound **7b** and 3-(trifluoromethyl)aniline **(g)** as a yellow solid. M.p. 169-171 °C. $^{1}$H-NMR (DMSO-*d*6, 500 MHz) $\delta$: 10.53 (s, 1H), 8.26 (br.s, 1H), 8.04 (d, *J* = 8.0 Hz, 1H), 7.95 (d, *J* = 8.0 Hz, 2H), 7.60 (d, *J* = 8.0 Hz, 1H), 7.56 (d, *J* = 8.0 Hz, 2H), 7.46 (d, *J* = 7.4 Hz, 1H), 7.38 (d, J = 6.7 Hz, 1H), 7.27 (d, *J* = 8.3 Hz, 1H), 7.09 (s, 1H), 6.52 (br.s, 1H), 4.57 (d, *J* = 5.8 Hz, 2H), 2.32 (s, 3H); $^{13}$C-NMR (DMSO-*d*6, 126 MHz): $\delta$ = 166.3, 147.4, 147.3, 144.2, 140.5, 133.6, 131.0, 130.8, 130.3, 128.5, 127.3, 124.2, 120.3, 116.8, 111.1, 103.2, 46.3, 18.5. HRMS(ESI+) m/z calcd for $C_{22}H_{18}F_3N_3O_3$ [M + H]+ 430.1300, found 430.1374. Purity: 98.18% (by HPLC).

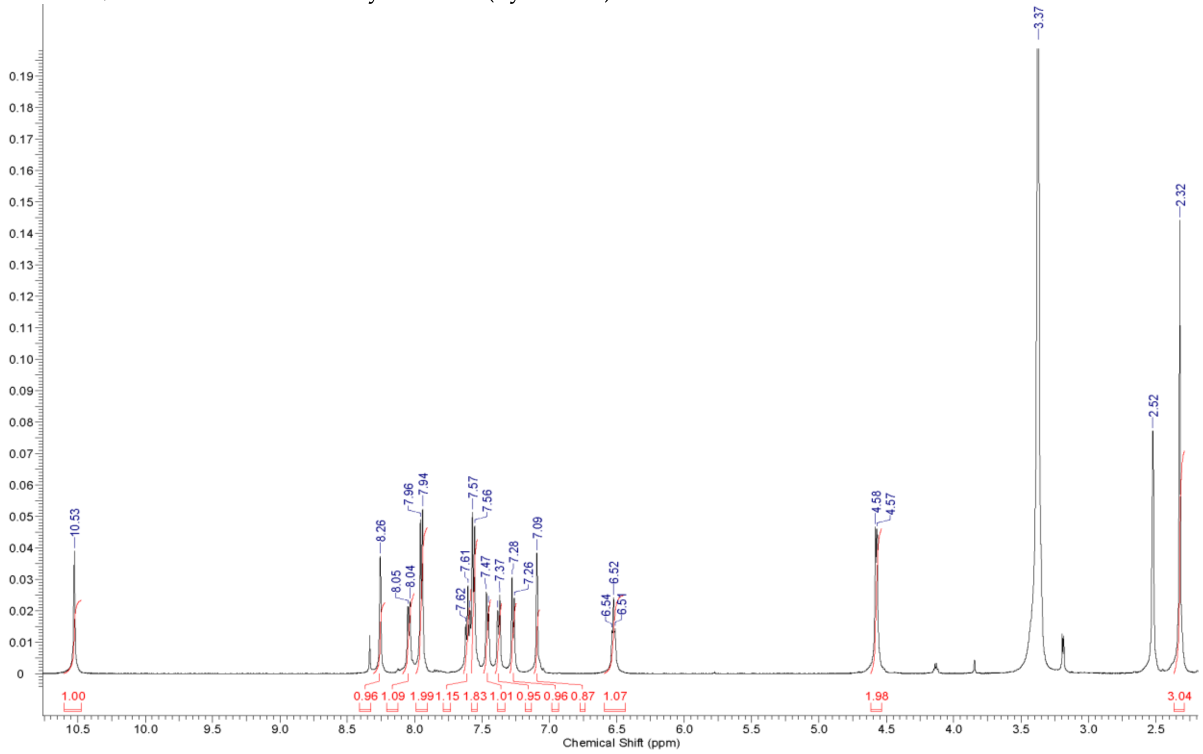


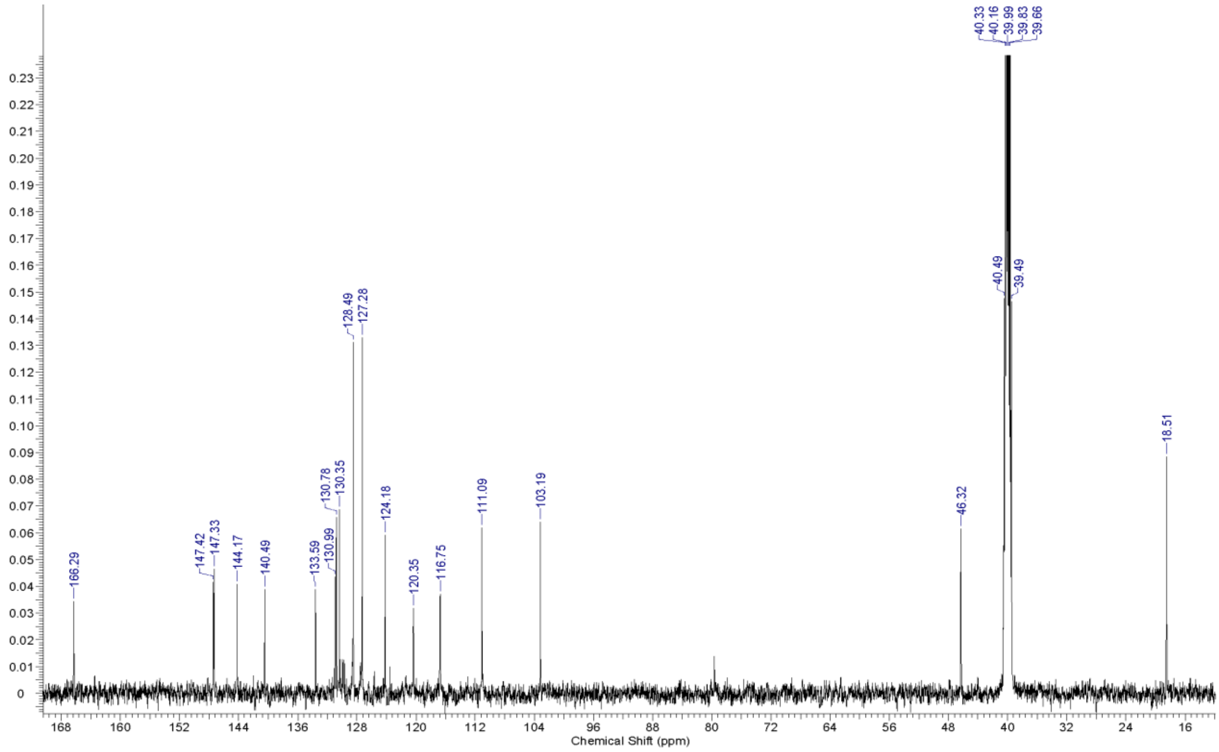

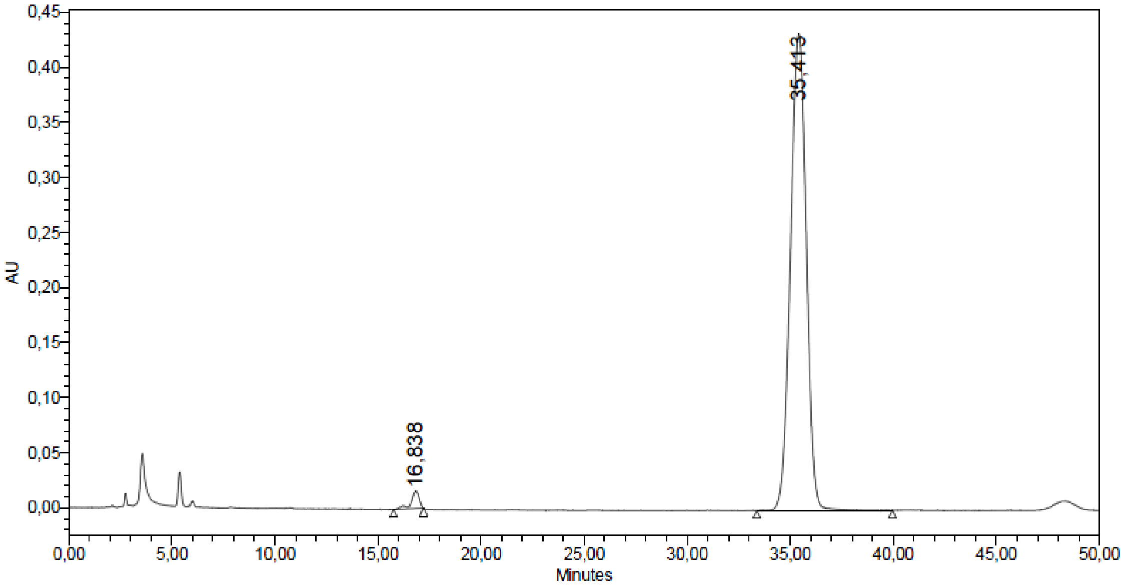


| | RT | Area | % Area | Height |
|---|---|---|---|---|
| 1 | 16,838 | 428307 | 1,82 | 15646 |
| 2 | 35,413 | 23076489 | 98,18 | 432507 |

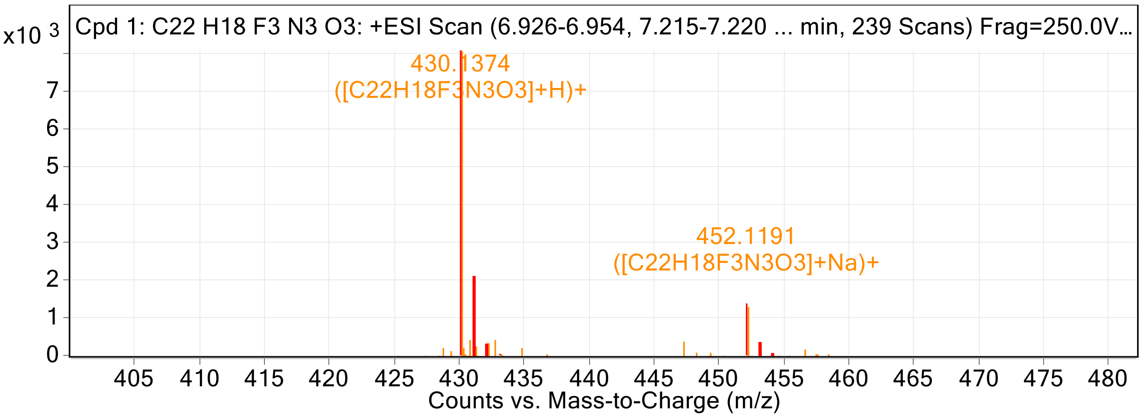

***Methyl 4-amino-3-[(4-{[(2-methy-5-nitrophenyl)amino]methyl}benzoyl)amino]benzoate*** **(19)** was synthesized from compound **7b** and methyl 3,4-diaminobenzoate **(f)** as a yellow solid. M.p. 208-210 °C. $^{1}$H-NMR (DMSO-$d_6$, 500 MHz) $\delta$: 9.63 (s, 1H), 7.98 (d, $J$ = 8.3 Hz, 2H), 7.79 (d, $J$ = 1.6 Hz, 1H), 7.60 (dd, $J$ = 8.7, 1.9 Hz, 1H), 7.53 (d, $J$ = 8.0 Hz, 2H), 7.38 (dd, $J$ = 8.0, 2.2 Hz, 1H), 7.27 (d, $J$ = 8.0 Hz, 1H), 7.10 (d, $J$ = 2.2 Hz, 1H), 6.79 (d, $J$ = 8.7 Hz, 1H), 6.50 - 6.55 (m, 1H), 5.86 (s, 2H), 4.54 - 4.59 (m, 2H), 3.77 (s, 3H), 2.33 (s, 3H); $^{13}$C-NMR (DMSO-$d_6$, 126 MHz) $\delta$: 166.6, 166.1, 148.9, 147.4, 147.4, 143.6, 133.6, 131.0, 130.8, 129.4, 128.8, 128.6, 127.0, 122.3, 116.6, 115.0, 111.0, 103.2, 51.8, 46.3, 18.5. HRMS(ESI+) m/z calcd for $C_{23}H_{22}N_4O_5$ [M + H]+ 435.1590, found 435.1656. Purity: 95.87% (by HPLC).

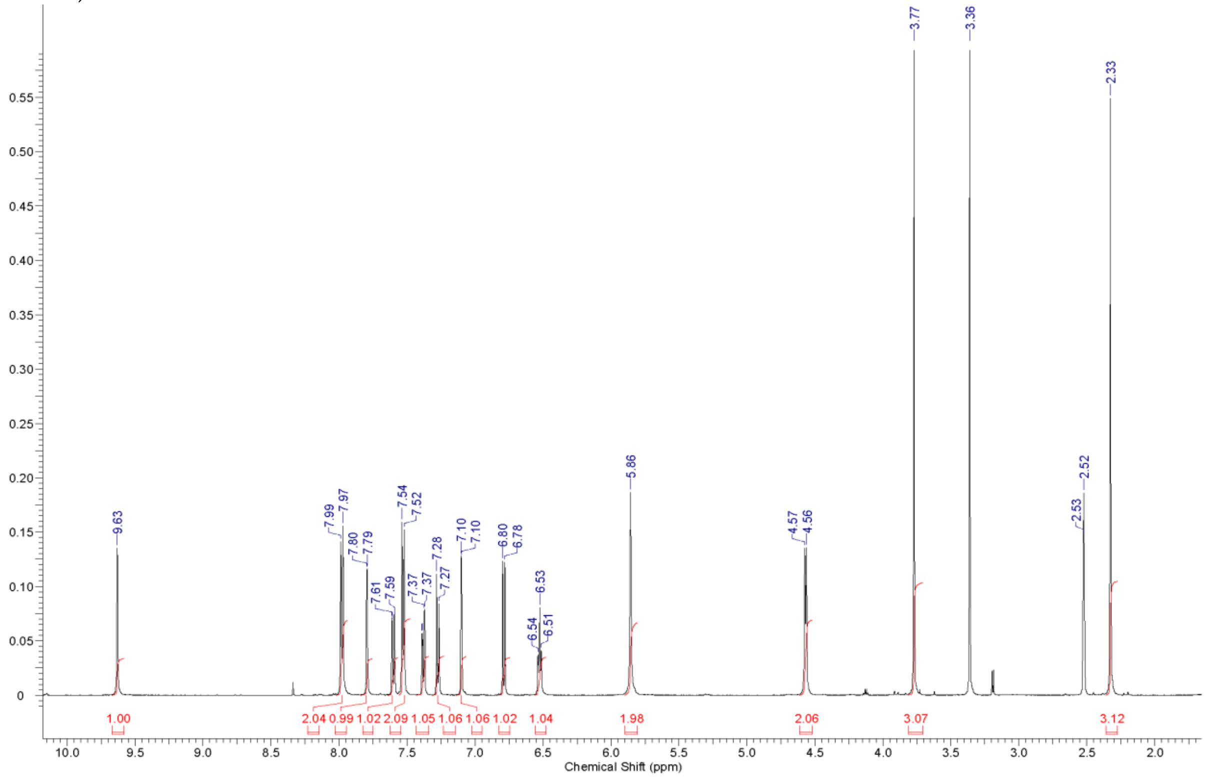


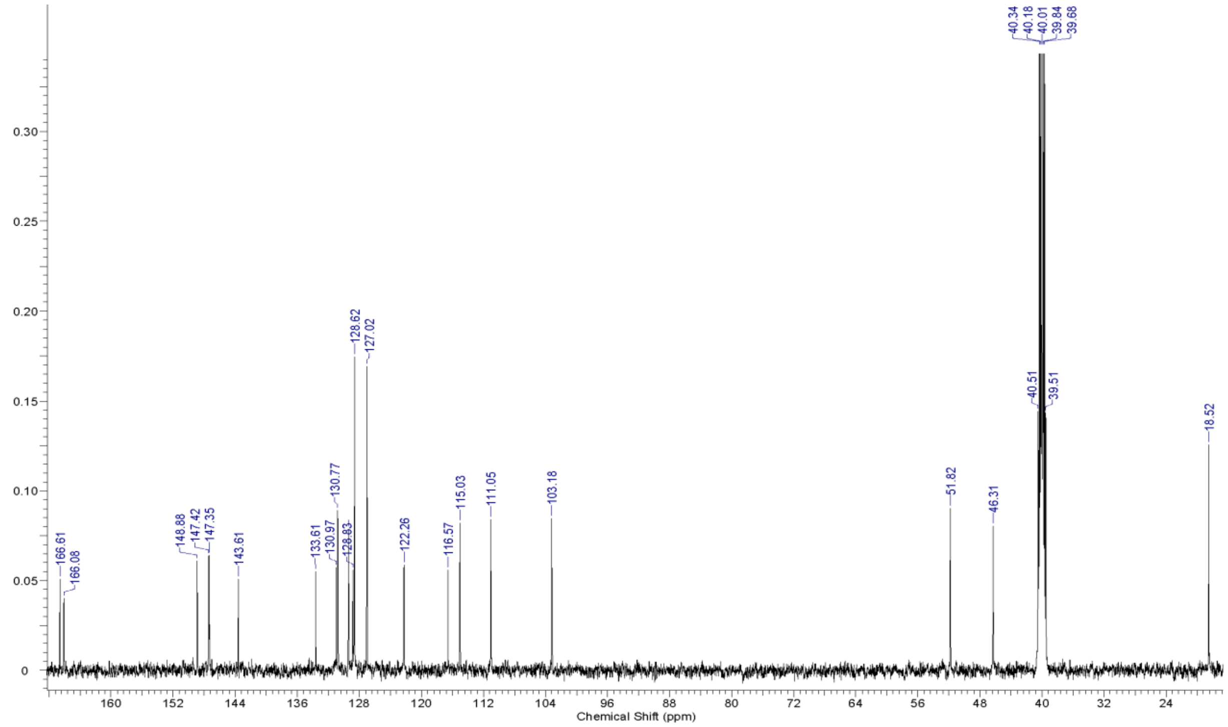

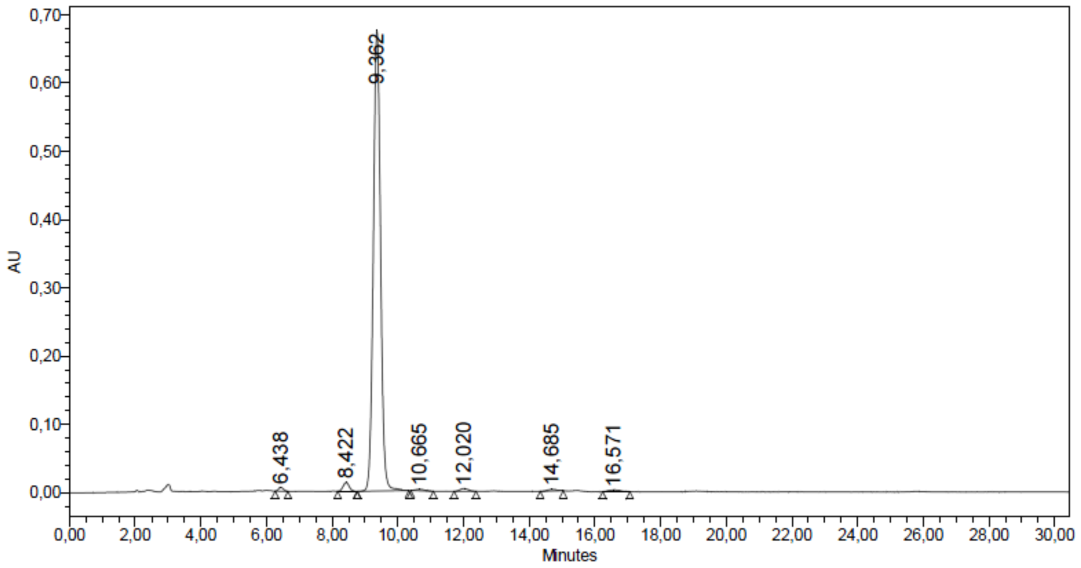


|   | RT | Area | % Area | Height |
|---|---|---|---|---|
| 1 | 6,438 | 58574 | 0,55 | 5793 |
| 2 | 8,422 | 166704 | 1,56 | 12976 |
| 3 | 9,362 | 10262515 | 95,87 | 674785 |
| 4 | 10,665 | 43142 | 0,40 | 2158 |
| 5 | 12,020 | 69271 | 0,65 | 3960 |
| 6 | 14,685 | 48607 | 0,45 | 2541 |
| 7 | 16,571 | 55643 | 0,52 | 2463 |

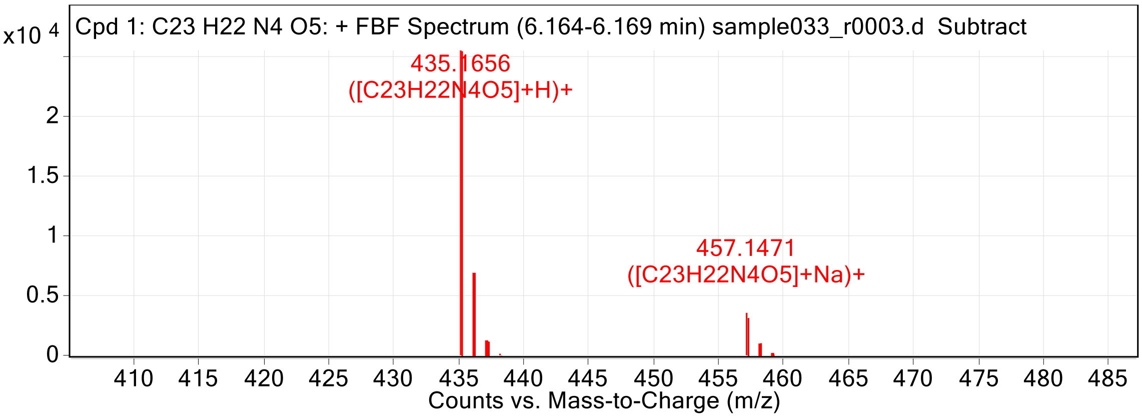

***4-{[(4-{[2-(methylcarbamoyl)pyridin-4-yl]oxy}phenyl)amino]methyl}-N-[3-(trifluoromethyl)phenyl]benzamide* (20)** was synthesized from compound **7c** and 3-(trifluoromethyl)aniline **(g)** as a white solid. M.p. 170-171 °C. $^{1}$H-NMR (DMSO-$d_6$, 500 MHz) δ: 10.54 (s, 1H), 8.77 (d, $J$ = 4.8 Hz, 1H), 8.47 (d, $J$ = 5.4 Hz, 1H), 8.28 (s, 1H), 8.07 (d, $J$ = 8.3 Hz, 1H), 7.97 (d, $J$ = 8.3 Hz, 2H), 7.60 - 7.67 (m, 1H), 7.58 (d, $J$ = 8.0 Hz, 2H), 7.47 (d, $J$ = 7.7 Hz, 1H), 7.33 (d, $J$ = 2.6 Hz, 1H), 7.09 (dd, $J$ = 5.8, 2.6 Hz, 1H), 6.95 (d, $J$ = 8.7 Hz, 2H), 6.69 (d, $J$ = 9.0 Hz, 2H), 6.58 (s, 1H), 4.42 (d, $J$ = 5.4 Hz, 2H), 2.79 (d, $J$ = 4.8 Hz, 3H); $^{13}$C-NMR (DMSO-$d_6$, 126 MHz) δ: 167.2, 166.3, 164.4, 152.8, 150.7, 147.1, 145.0, 143.6, 140.5, 133.5, 130.3, 128.4, 127.6, 124.2, 122.2, 120.3, 116.8, 116.7, 114.0, 108.7, 79.7, 46.9, 26.5. HRMS(ESI+) m/z calcd for $C_{28}H_{23}F_3N_4O_3$ [M + H]+ 521.1722, found 521.1800. Purity: 98.85% (by HPLC).

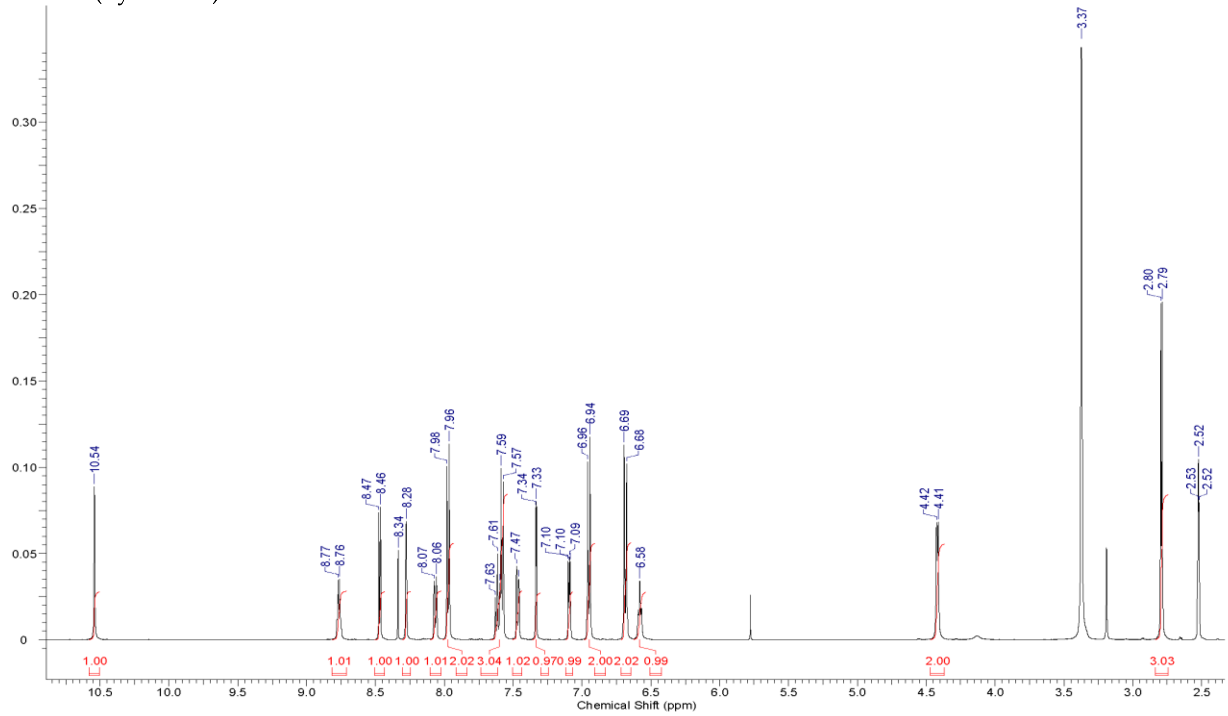


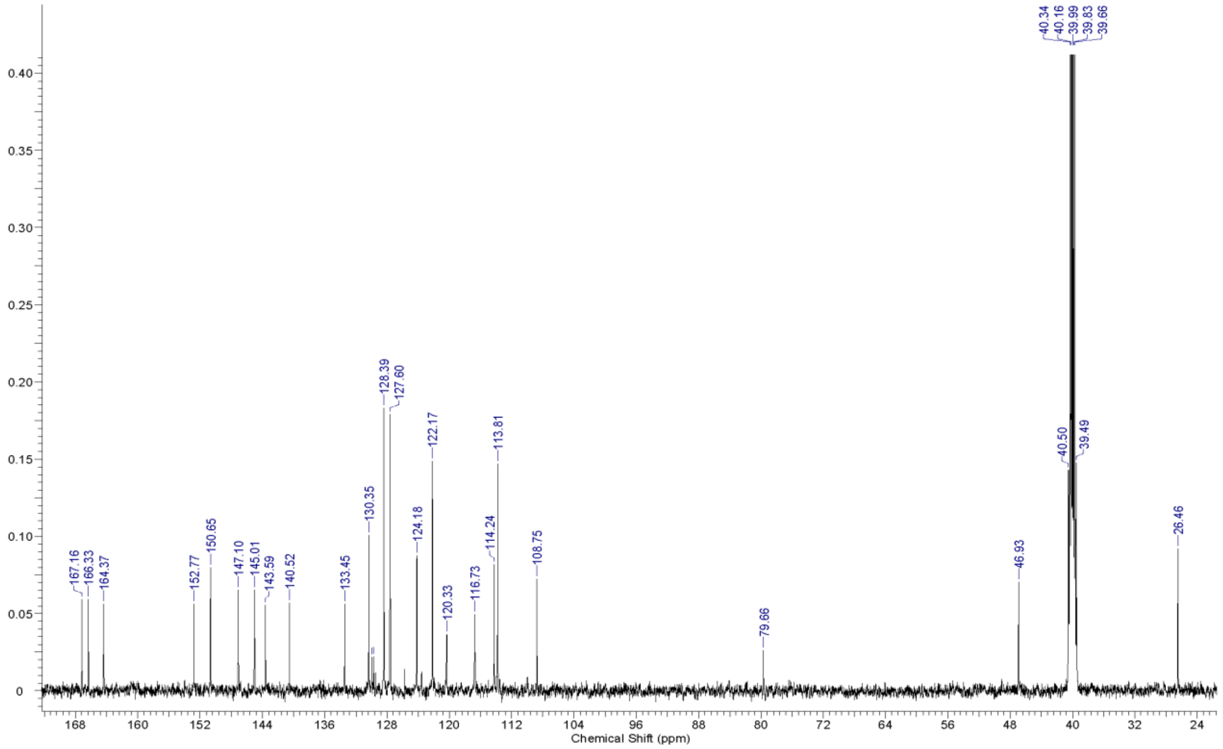

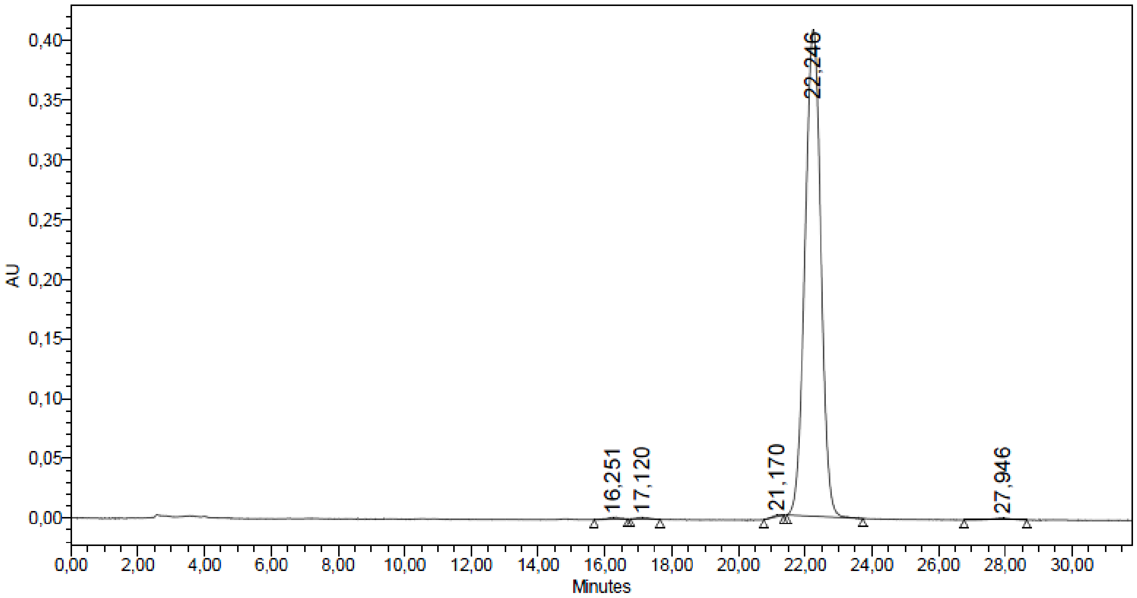


| | RT | Area | % Area | Height |
|---|---|---|---|---|
| 1 | 16,251 | 40447 | 0,30 | 1604 |
| 2 | 17,120 | 35045 | 0,26 | 1381 |
| 3 | 21,170 | 21117 | 0,16 | 1200 |
| 4 | 22,246 | 13416512 | 98,85 | 407416 |
| 5 | 27,946 | 59665 | 0,44 | 1549 |

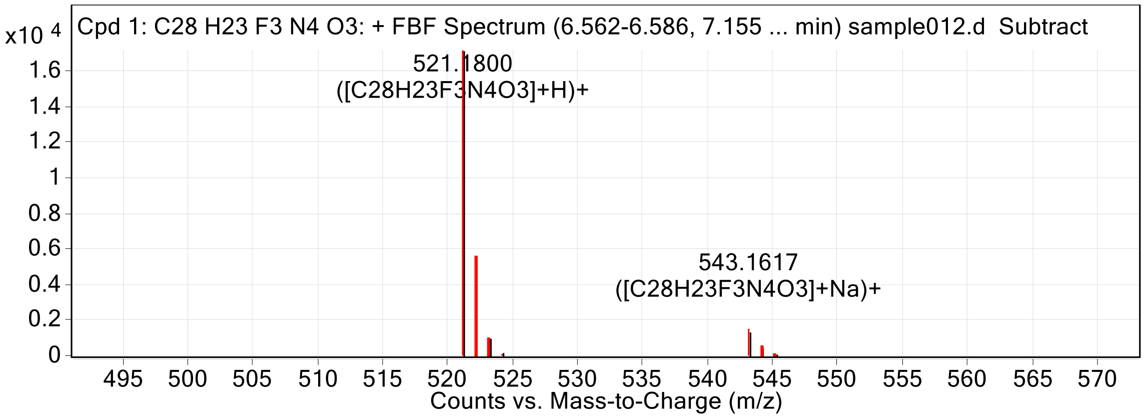

***Diethyl (4-(((4-((2-(methylcarbamoyl)pyridin-4-yl)oxy)phenyl)amino)methyl)benzoyl)glutamate* (21)** was synthesized from compound **7c** and diethyl glutamate **(h)** as a white solid. M.p. 162-164 °C. $^{1}$H-NMR (DMSO-$d_6$, 500 MHz) $\delta$: 8.76 (q, $J$ = 4.8 Hz, 1H), 8.70 (d, $J$ = 7.4 Hz, 1H), 8.47 (d, $J$ = 5.4 Hz, 1H), 7.87 (d, $J$ = 8.3 Hz, 2H), 7.51 (d, $J$ = 8.3 Hz, 2H), 7.33 (d, $J$ = 2.6 Hz, 1H), 7.08 (dd, $J$ = 5.4, 2.6 Hz, 1H), 6.91 - 6.95 (m, 2 H), 6.65 - 6.68 (m, 2 H), 6.53 (t, $J$ = 5.9 Hz, 1 H), 4.45 (ddd, $J$ = 9.6, 7.4, 5.1 Hz, 1H), 4.38 (d, $J$ = 6.1 Hz, 2H), 4.12 (qd, $J$ = 7.1, 2.4 Hz, 2H), 4.06 (q, $J$ = 7.1 Hz, 2H), 2.79 (d, $J$ = 4.8 Hz, 3H), 2.44 - 2.48 (m, 2H), 2.08 - 2.17 (m, 1H), 1.97 - 2.07 (m, 1H), 1.19 (dt, $J$ = 13.1, 7.1 Hz, 6H); $^{13}$C-NMR (DMSO-$d_6$, 126 MHz): $\delta$ = 172.7, 172.3, 167.2, 167.1, 164.4, 152.8, 150.7, 147.1, 144.4, 143.6, 132.7, 128.1, 127.4, 122.1, 114.2, 113.8, 108.8, 61.0, 60.4, 52.5, 47.0, 33.8, 30.7, 24.9, 14.6. HRMS(ESI+) m/z calcd for $C_{30}H_{34}N_4O_7$ [M + H]+ 563.2427, found 563.2502. Purity: 98.00% (by HPLC).

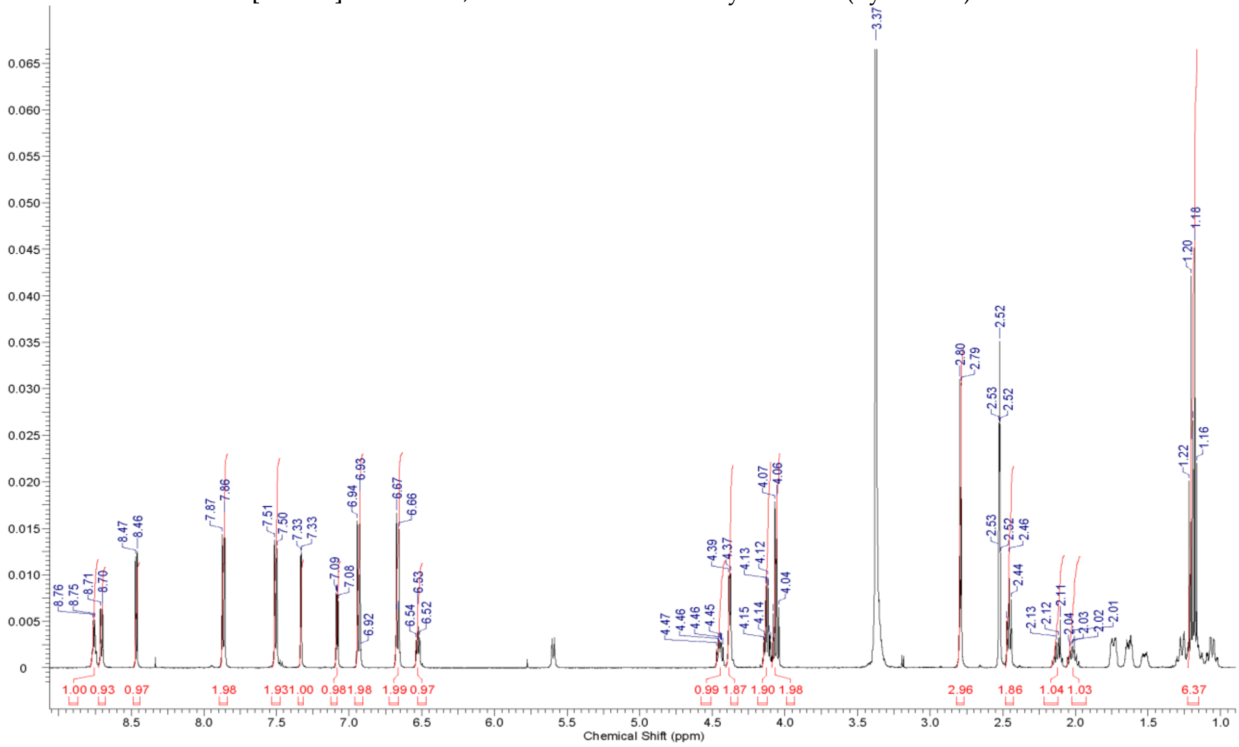


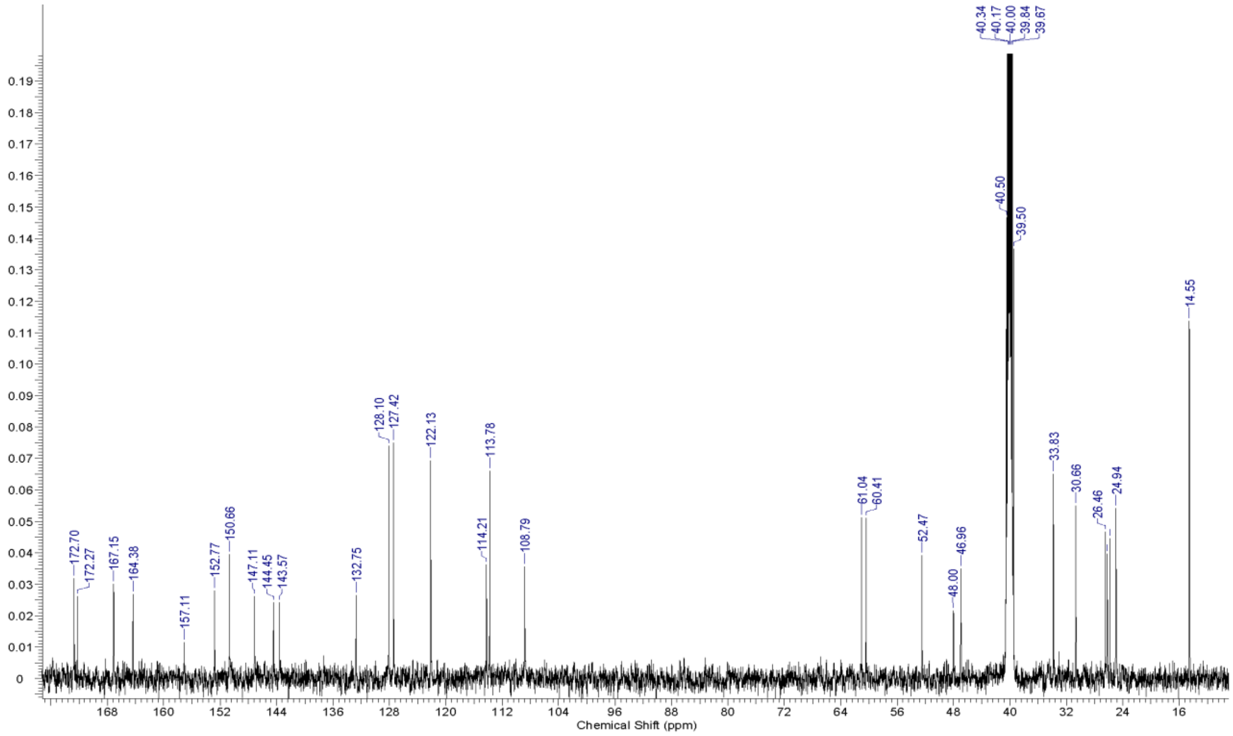

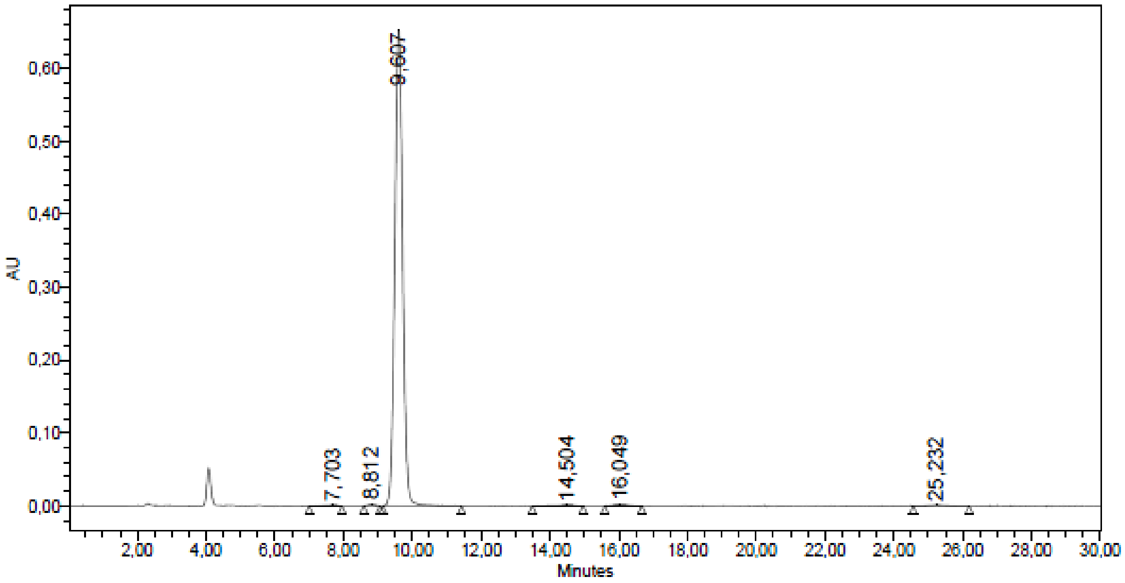


| | RT | Area | % Area | Height |
|---|---|---|---|---|
| 1 | 7,703 | 27948 | 0,26 | 1213 |
| 2 | 8,812 | 28930 | 0,27 | 2121 |
| 3 | 9,607 | 10371509 | 98,00 | 653440 |
| 4 | 14,504 | 52567 | 0,50 | 1864 |
| 5 | 16,049 | 61617 | 0,58 | 2420 |
| 6 | 25,232 | 40703 | 0,38 | 1090 |

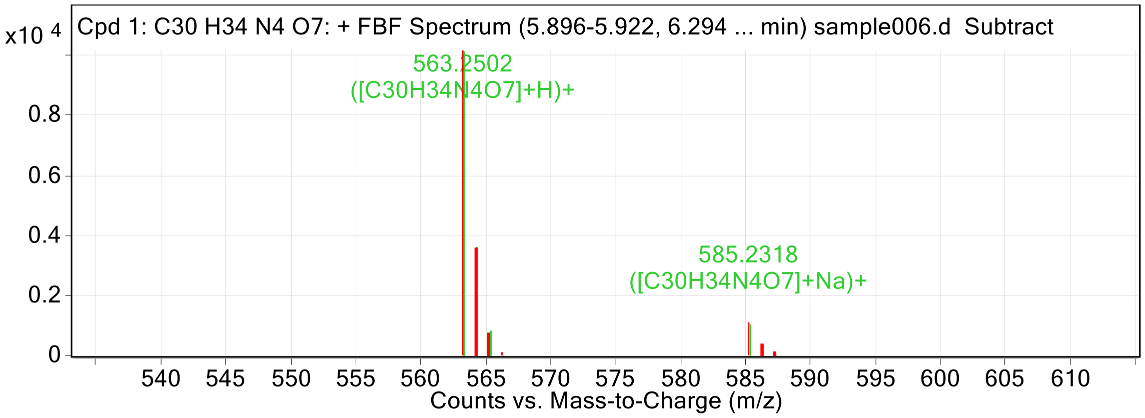

***N*-(3-(4-methyl-1H-imidazol-1-yl)-5-(trifluoromethyl)phenyl)-4-(((4-(pyridin-2-yl)pyrimidin-2-yl)amino)methyl)benzamide** **(22)** was synthesized from compound **7d** and 3-(4-methyl-1H-imidazol-1-yl)-5-(trifluoromethyl)aniline **(a)** as a white solid. M.p. 150-152 °C. $^1$H-NMR (DMSO-$d_6$, 500 MHz) $\delta$: 10.70 (s, 1H), 9.26 (br.s., 1H), 8.71 (br.s., 1H), 8.44 (d, $J$ = 4.8 Hz, 2H), 8.34 (br.s., 1H), 8.31 (br.s., 1H), 8.18 (s, 1H), 8.05 (t, $J$ = 6.1 Hz, 1H), 7.99 (d, $J$ = 8.3 Hz, 2H), 7.74 (s, 1H), 7.55 (d, $J$ = 13.1 Hz, 4H), 7.29 (d, $J$ = 5.1 Hz, 1H), 4.69 (d, $J$ = 5.1 Hz, 2H), 2.21 (s, 3H); $^{13}$C-NMR (DMSO-$d_6$, 126 MHz): $\delta$: 166.4, 162.9, 151.8, 148.5, 141.9, 138.3, 134.7, 132.8, 128.3, 127.6, 124.3, 115.5, 114.8, 112.1, 49.1, 44.5, 13.8. HRMS(ESI+) m/z calcd for $C_{28}H_{22}F_3N_7O$ [M + H]+ 530.1838, found 530.1913. Purity: 96.67% (by HPLC).

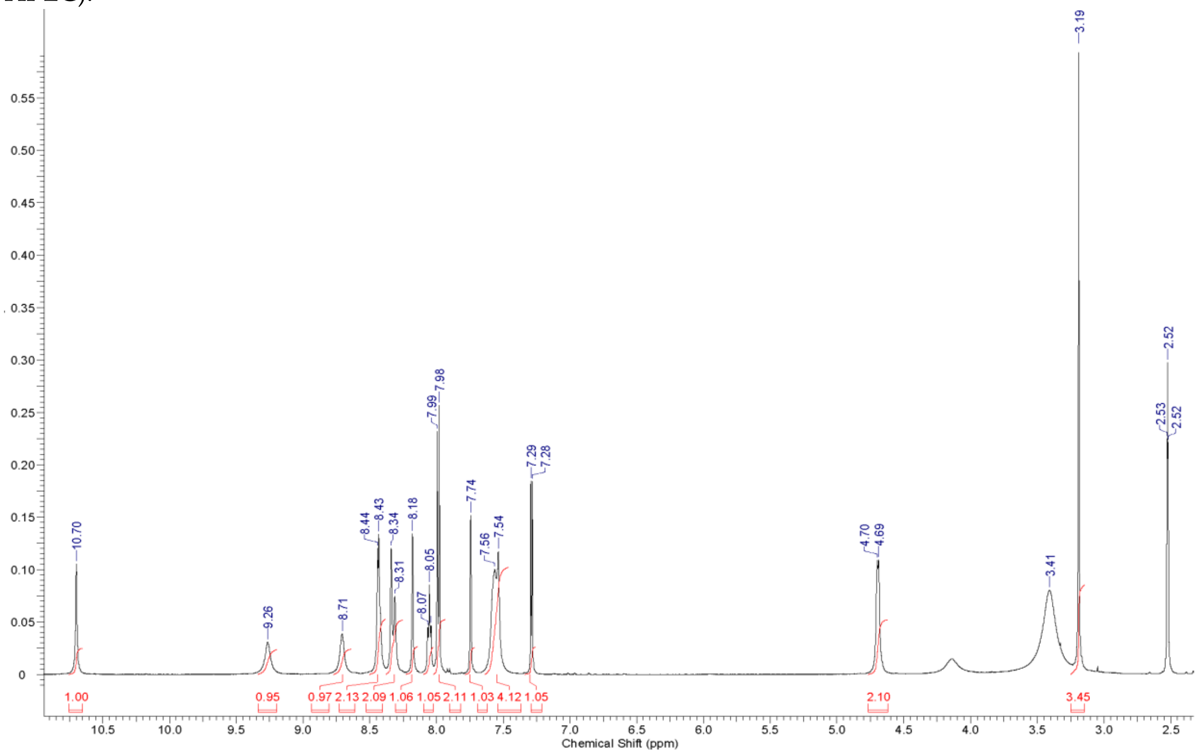


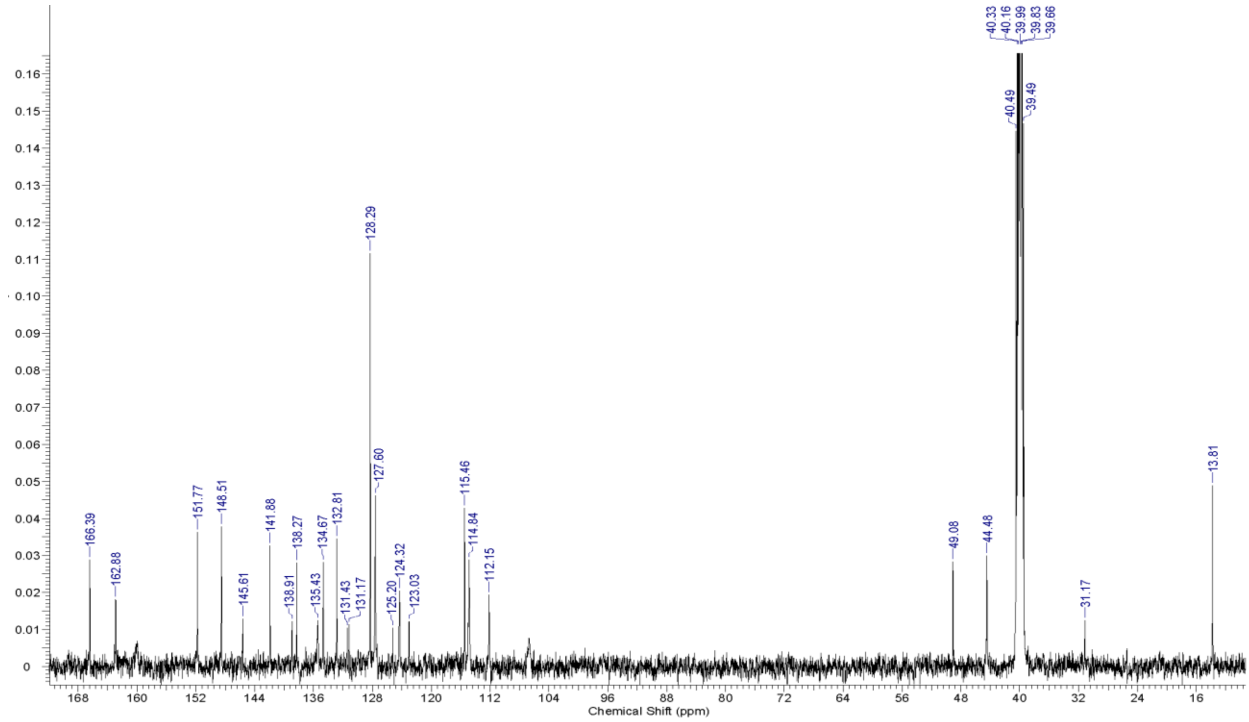

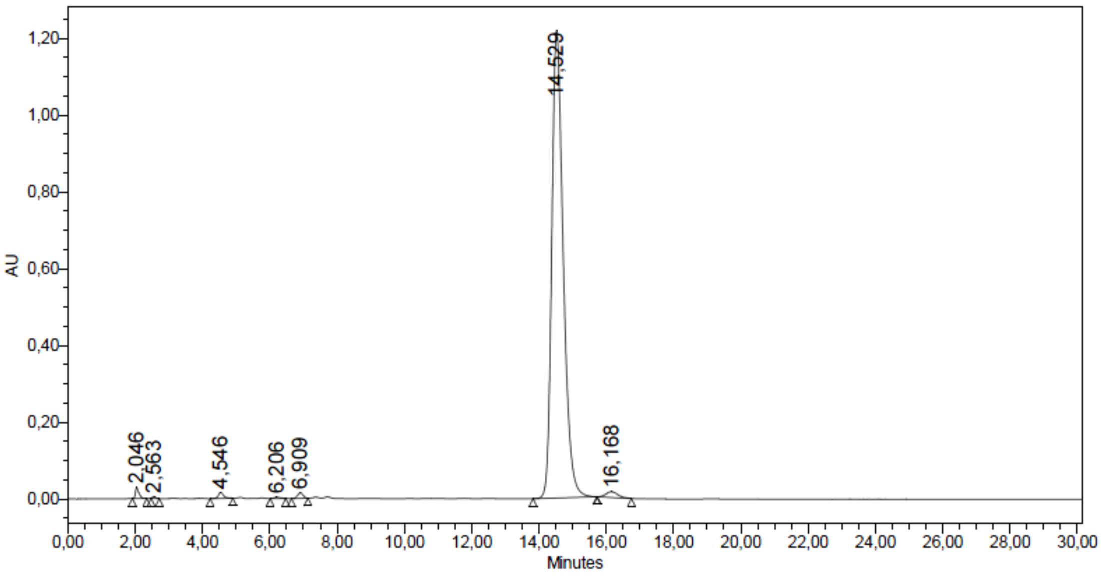


| | RT | Area | % Area | Height |
|---|---|---|---|---|
| 1 | 2,046 | 203609 | 0,68 | 28497 |
| 2 | 2,563 | 32192 | 0,11 | 5347 |
| 3 | 4,546 | 170305 | 0,57 | 15943 |
| 4 | 6,206 | 42632 | 0,14 | 3216 |
| 5 | 6,909 | 163060 | 0,55 | 14126 |
| 6 | 14,529 | 28807353 | 96,67 | 1217403 |
| 7 | 16,168 | 381489 | 1,28 | 14964 |

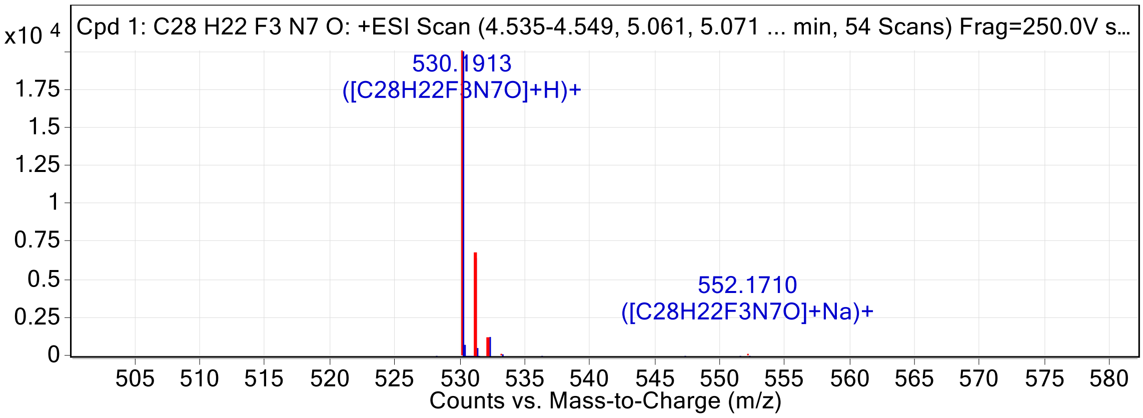

***Diethyl (4-(((4-(pyridin-3-yl)pyrimidin-2-yl)amino)methyl)benzoyl)glutamate*** **(23)** was synthesized from compound **7d** and diethyl glutamate **(h)** as a white solid. M.p. 130-132 °C. $^{1}$H-NMR (DMSO-$d_6$, 500 MHz) $\delta$: 9.23 - 9.30 (m, 1H), 8.69 - 8.75 (m, 1H), 8.65 - 8.69 (m, 1H), 8.43 (d, $J$ = 5.1 Hz, 2H), 7.98 - 8.06 (m, 1H), 7.84 (d, $J$ = 8.3 Hz, 2H), 7.53 - 7.61 (m, 1H), 7.42 - 7.52 (m, 2H), 7.28 (d, $J$ = 5.1 Hz, 1H), 4.61 - 4.69 (m, 2H), 4.40 - 4.46 (m, 1H), 4.01 - 4.19 (m, 4H), 2.44 (s, 2H), 1.95 - 2.19 (m, 2H), 1.12 - 1.25 (m, 6H); $^{13}$C-NMR (DMSO-$d_6$, 126 MHz) $\delta$: 172.7, 172.3, 167.1, 151.6, 148.3, 134.9, 132.9, 132.5, 127.9, 127.4, 124.4, 61.0, 60.4, 52.4, 44.4, 30.6, 26.2, 14.5. HRMS(ESI+) m/z calcd for $C_{26}H_{29}N_5O_5$ [M + H]+ 492.2169, found 492.2238. Purity: 98.89% (by HPLC).

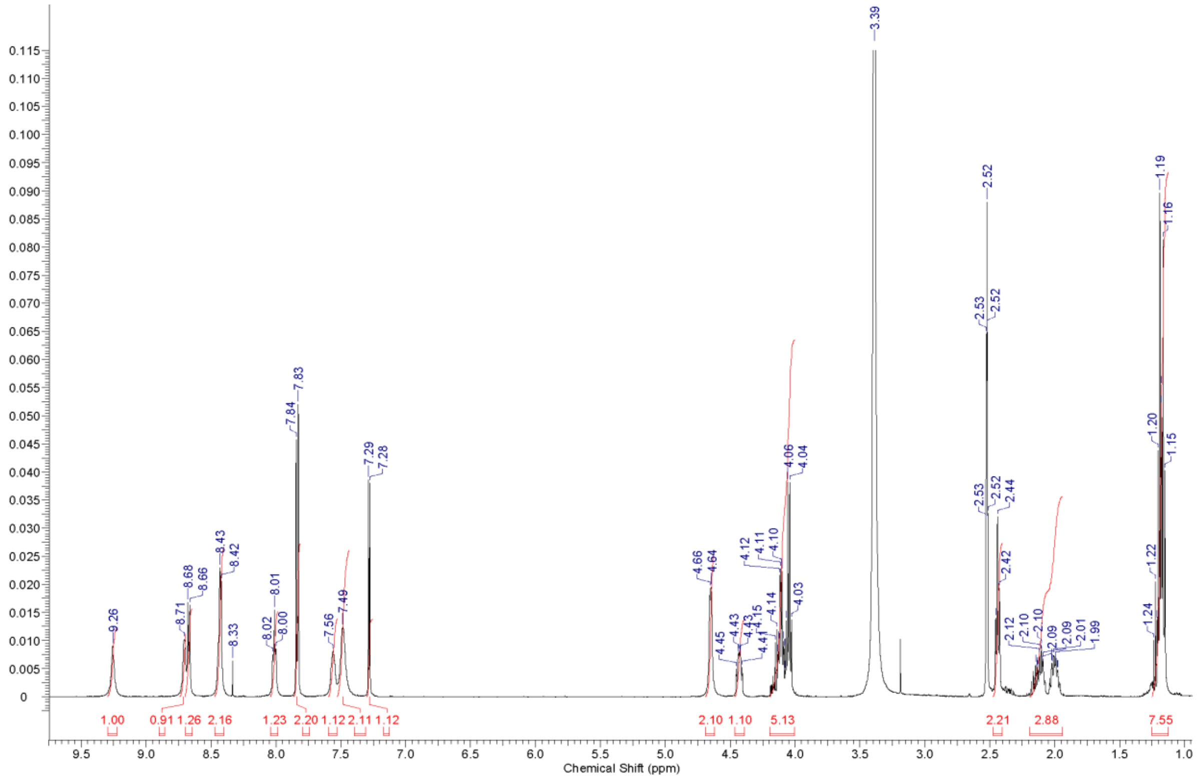


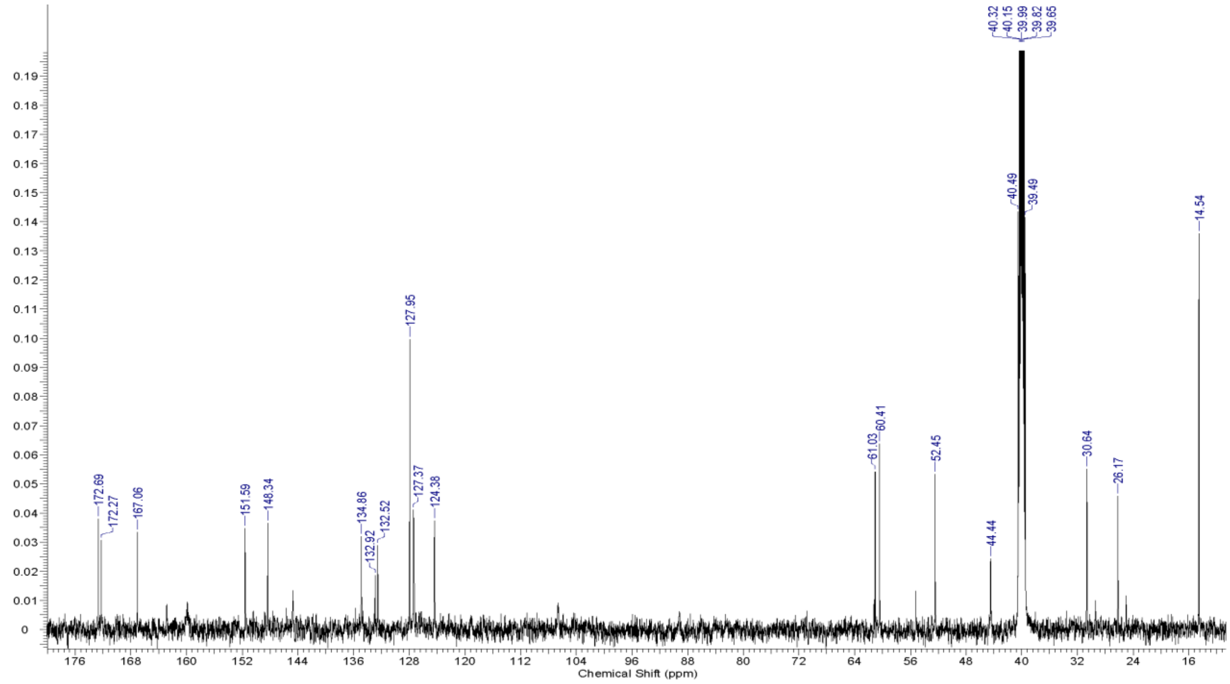

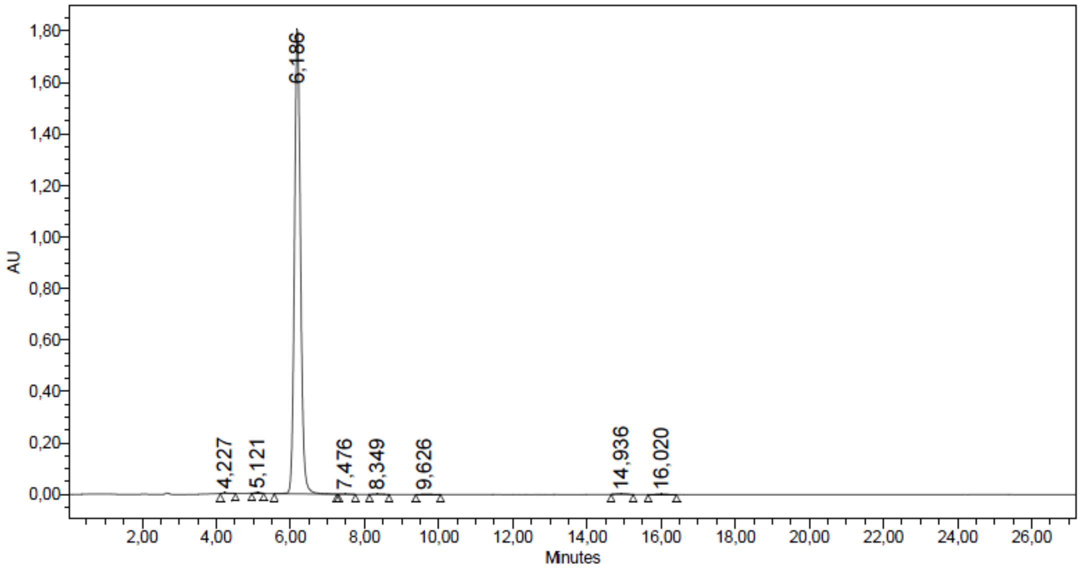


| | RT | Area | % Area | Height |
|---|---|---|---|---|
| 1 | 4,227 | 30381 | 0,15 | 3390 |
| 2 | 5,121 | 38730 | 0,19 | 3724 |
| 3 | 6,186 | 20456978 | 98,89 | 1804225 |
| 4 | 7,476 | 8353 | 0,04 | 614 |
| 5 | 8,349 | 27300 | 0,13 | 1724 |
| 6 | 9,626 | 23021 | 0,11 | 971 |
| 7 | 14,936 | 62153 | 0,30 | 3277 |
| 8 | 16,020 | 38923 | 0,19 | 1795 |

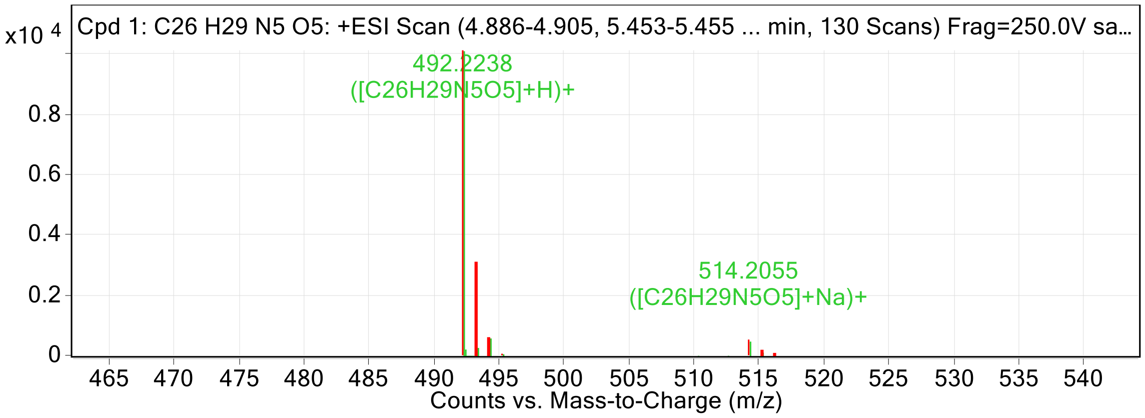

***4-({[4-(pyridin-3-yl)pyrimidin-2-yl]amino}methyl)-N-[3-(trifluoromethyl)phenyl]benzamide* (24)** was synthesized from compound **7d** and 3-(trifluoromethyl)aniline **(g)** as a white solid. M.p. 138-141 °C. $^{1}$H-NMR (DMSO-*d*$_6$, 500 MHz) $\delta$: 10.51 (s, 1H), 9.29 (br.s., 1H), 8.75 (br.s., 1H), 8.52 (d, *J* = 7.7 Hz, 1H), 8.46 (d, *J* = 4.8 Hz, 1H), 8.26 (s, 1H), 8.09 (br.s., 1H), 8.05 (d, *J* = 8.3 Hz, 1H), 7.95 (d, *J* = 8.3 Hz, 2H), 7.52 - 7.66 (m, 4H), 7.46 (d, *J* = 7.7 Hz, 1H), 7.32 (d, *J* = 5.1 Hz, 1H), 4.69 (br.s., 2H); $^{13}$C-NMR (DMSO-*d*$_6$, 126 MHz) $\delta$: 166.3, 150.9, 147.7, 145.2, 140.5, 135.7, 133.2, 130.3, 128.3, 127.5, 124.7, 124.2, 120.3, 116.7. HRMS(ESI+) m/z calcd for $C_{24}H_{18}F_3N_5O$ [M + H]+ 450.1463, found 450.1535. Purity: 99.00% (by HPLC).

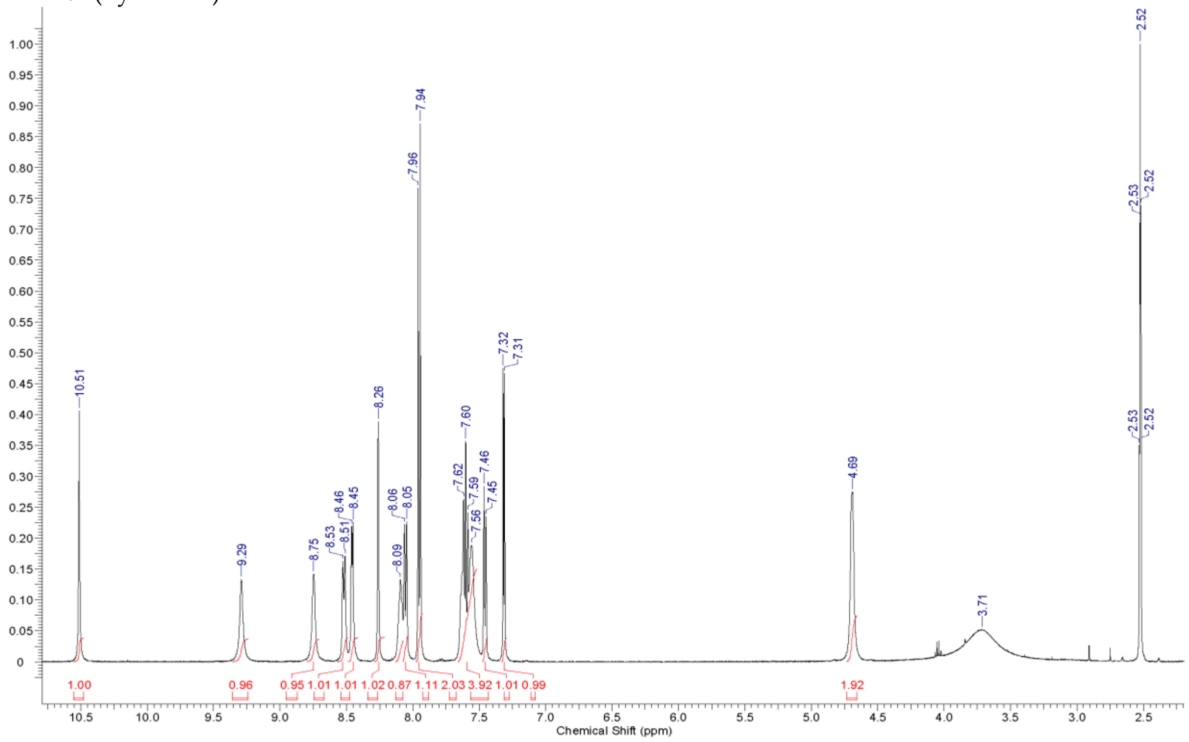


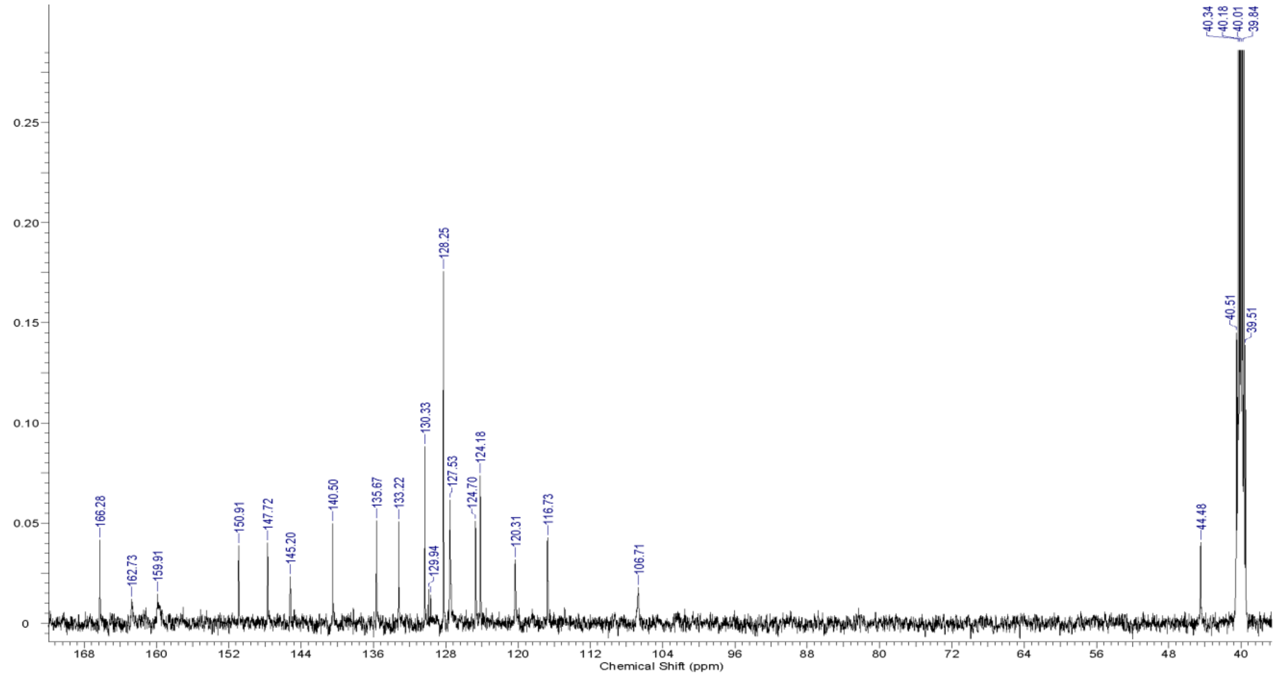

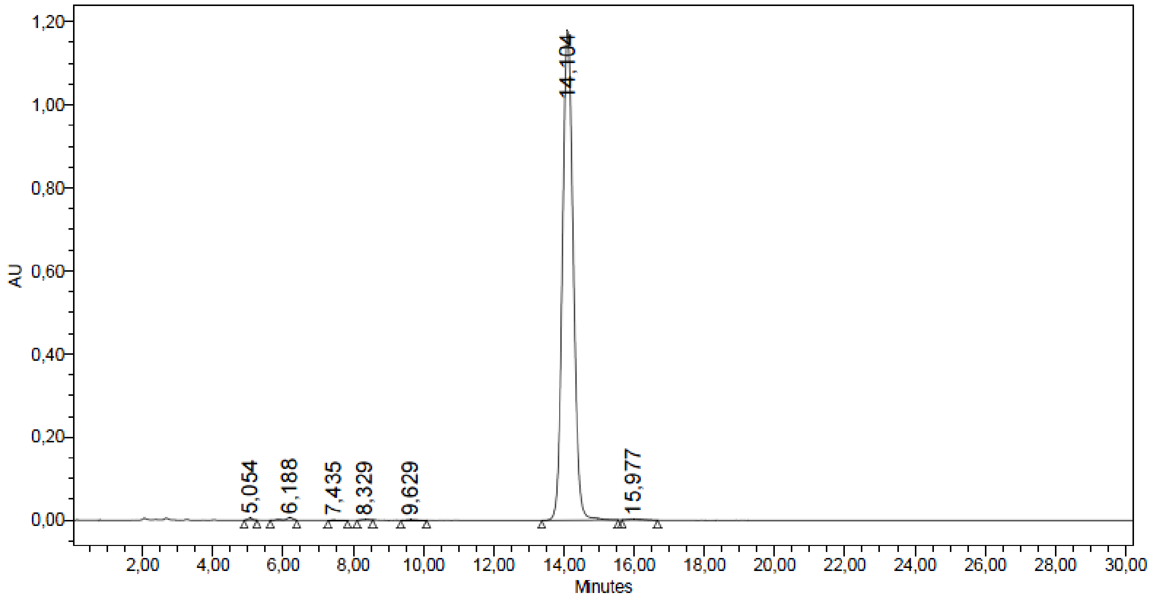


| | RT | Area | % Area | Height |
|---|---|---|---|---|
| 1 | 5,054 | 40834 | 0,16 | 4528 |
| 2 | 6,188 | 86101 | 0,34 | 6480 |
| 3 | 7,435 | 9793 | 0,04 | 614 |
| 4 | 8,329 | 21269 | 0,08 | 1484 |
| 5 | 9,629 | 26987 | 0,11 | 1228 |
| 6 | 14,104 | 24914823 | 99,00 | 1179120 |
| 7 | 15,977 | 67917 | 0,27 | 2608 |

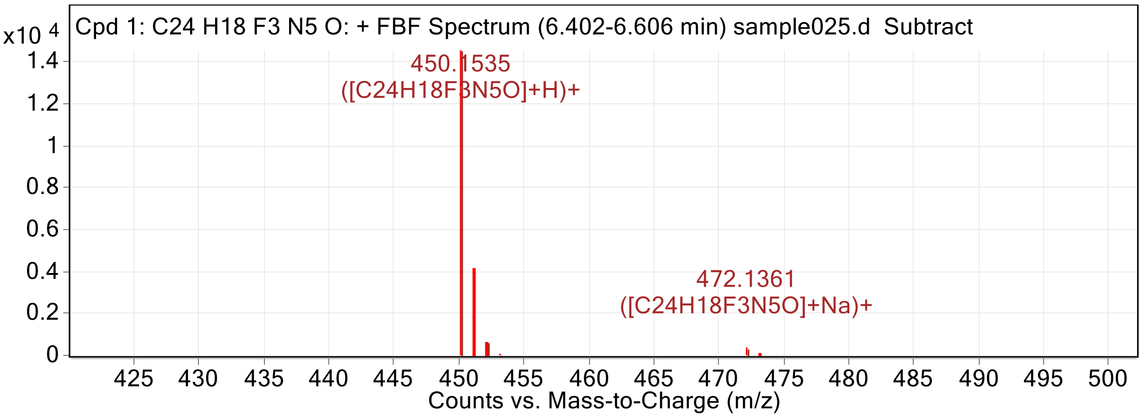

***4-({[4-(pyridin-3-yl)pyrimidin-2-yl]amino}methyl)-N-{[(2-methyl-5-nitrophenyl) benzoate* (25)** was synthesized from compound **7d** and 2-methyl-5-nitroaniline **(b)** as a white solid. M.p. 175-176 °C. $^{1}$H NMR (DMSO-$d_6$, 500 MHz) $\delta$: 10.08 (s, 1H), 9.27 (br.s., 1H), 8.71 (br.s., 1H), 8.44 (d, $J$ = 4.5 Hz, 2H), 8.36 (d, $J$ = 1.9 Hz, 1H), 8.02 - 8.08 (m, 2H), 7.97 (d, $J$ = 8.0 Hz, 2H), 7.58 (d, $J$ = 8.3 Hz, 4H), 7.29 (d, $J$ = 5.1 Hz, 1H), 4.69 (d, $J$ = 4.2 Hz, 2H), 2.39 (s, 3H); $^{13}$C NMR (DMSO-$d_6$, 126 MHz): $\delta$: 166.1, 151.7, 148.4, 146.2, 141.9, 137.9, 134.8, 132.9, 132.8, 132.0, 128.3, 127.6, 124.4, 120.8, 120.7, 44.5, 18.7. HRMS(ESI+) m/z calcd for $C_{24}H_{20}N_6O_3$ [M + H]+ 441.1597, found 441.1670. Purity: 99.42% (by HPLC).

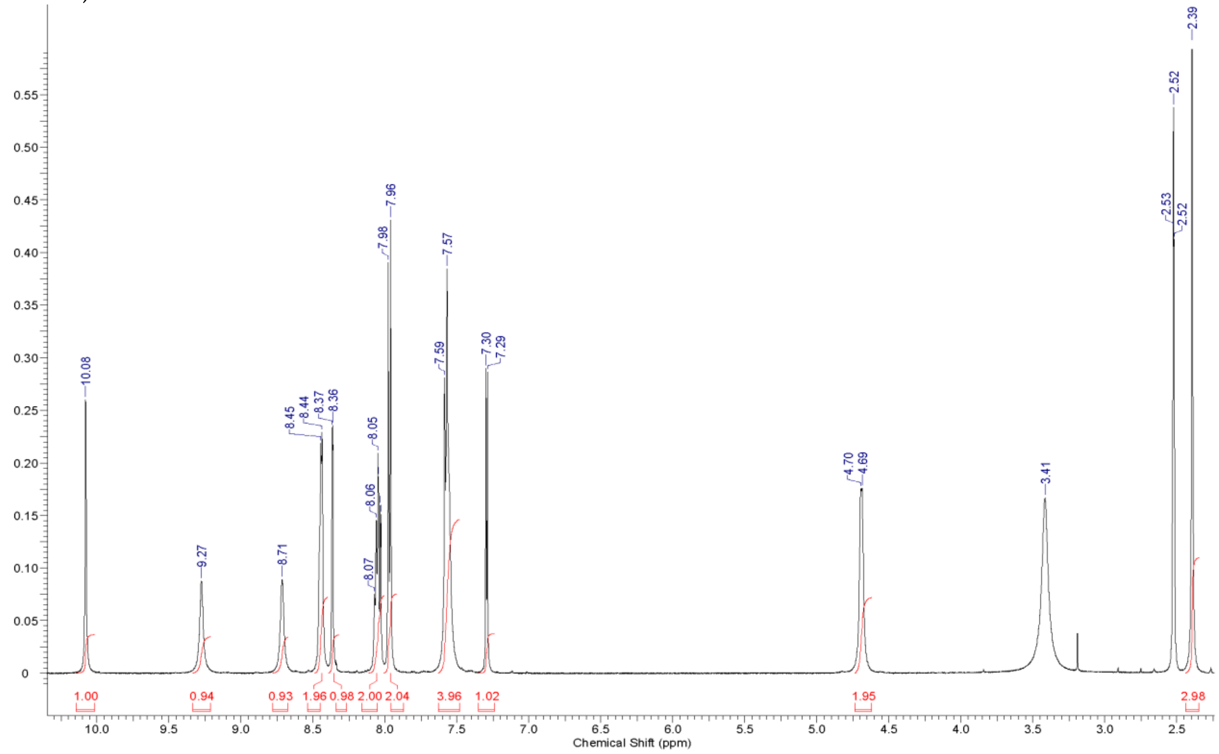


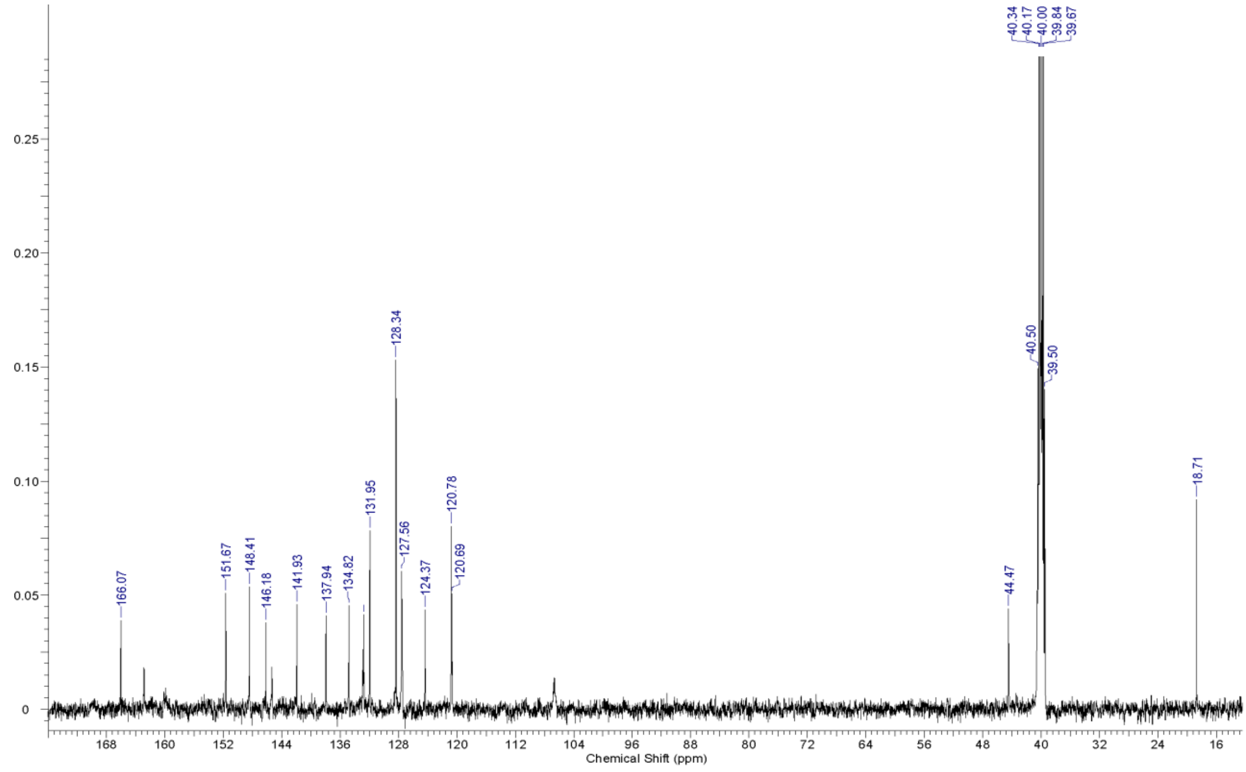

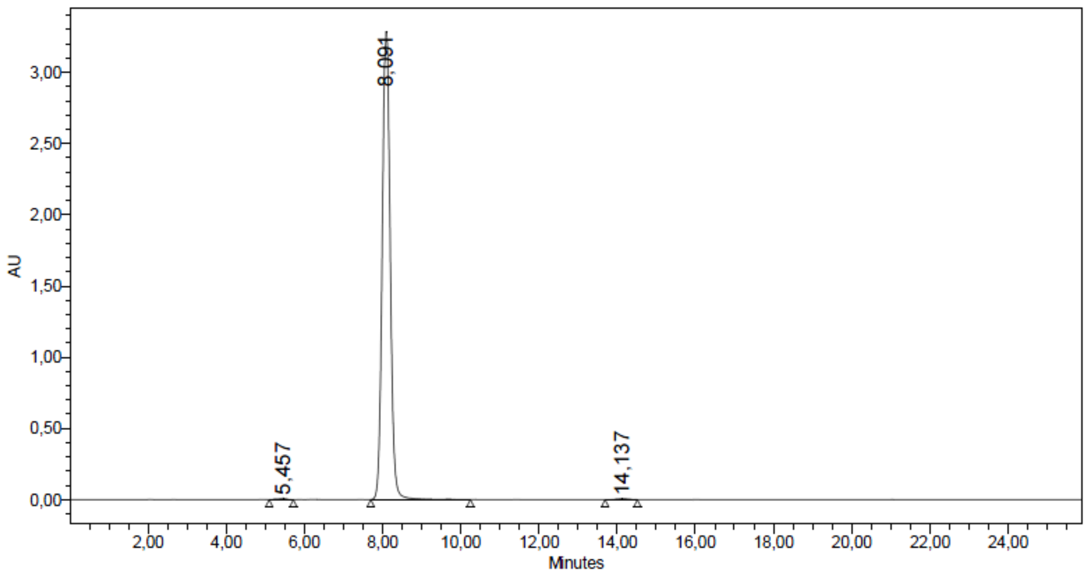


| | RT | Area | % Area | Height |
|---|---|---|---|---|
| 1 | 5,457 | 139488 | 0,30 | 10055 |
| 2 | 8,091 | 46707225 | 99,42 | 3281528 |
| 3 | 14,137 | 134504 | 0,29 | 6613 |

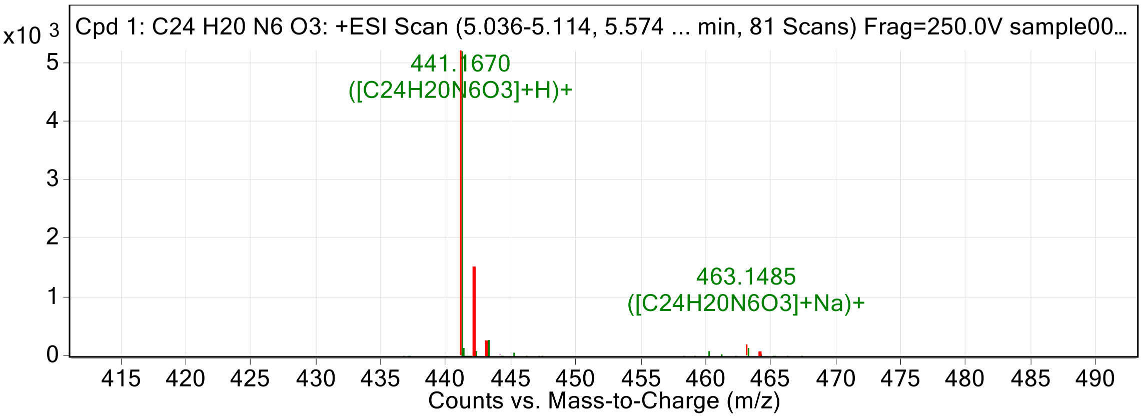

***Methyl 4-((4-(4-methoxybenzoyl)piperazin-1-yl)methyl)benzoate*** **(26i)** was synthesized from (4-methoxyphenyl)(piperazin-1-yl)methanone and methyl 4-formylbenzoate (**1**) as a white solid. The yield was 75%. $^{1}$H-NMR (DMSO-$d_6$, 500 MHz) $\delta$: 7.94 (d, *J* = 8.3 Hz, 2H), 7.49 (d, *J* = 8.3 Hz, 2H), 7.36 (d, *J* = 9.0 Hz, 2H), 6.99 (d, *J* = 8.7 Hz, 2H), 3.84 - 3.89 (m, 3H), 3.80 (s, 3H), 3.60 (s, 2H), 3.43 - 3.58 (m, 4H), 2.32 - 2.46 (m, 4H); $^{13}$C-NMR (DMSO-$d_6$, 126 MHz) $\delta$: 169.4, 166.6, 160.6, 144.2, 129.7, 129.5, 128.9, 128.2, 114.1, 79.6, 61.8, 55.7, 53.1, 52.6.

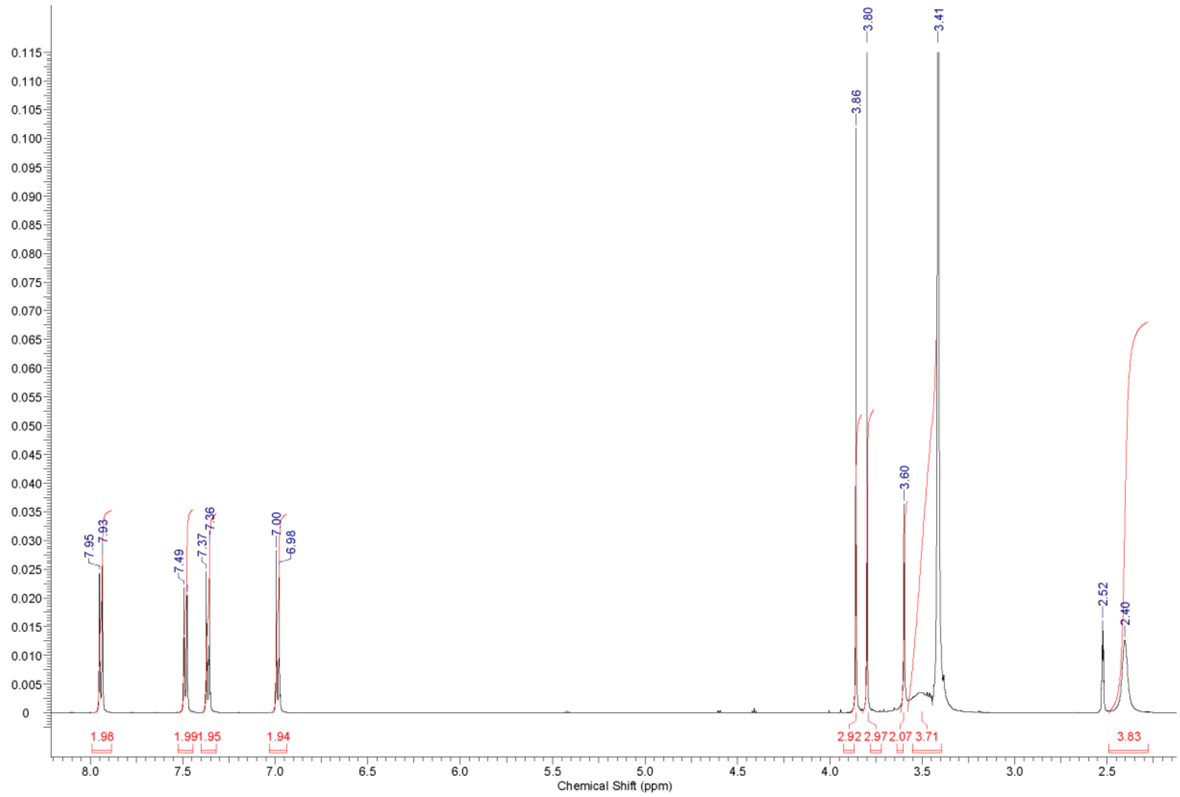


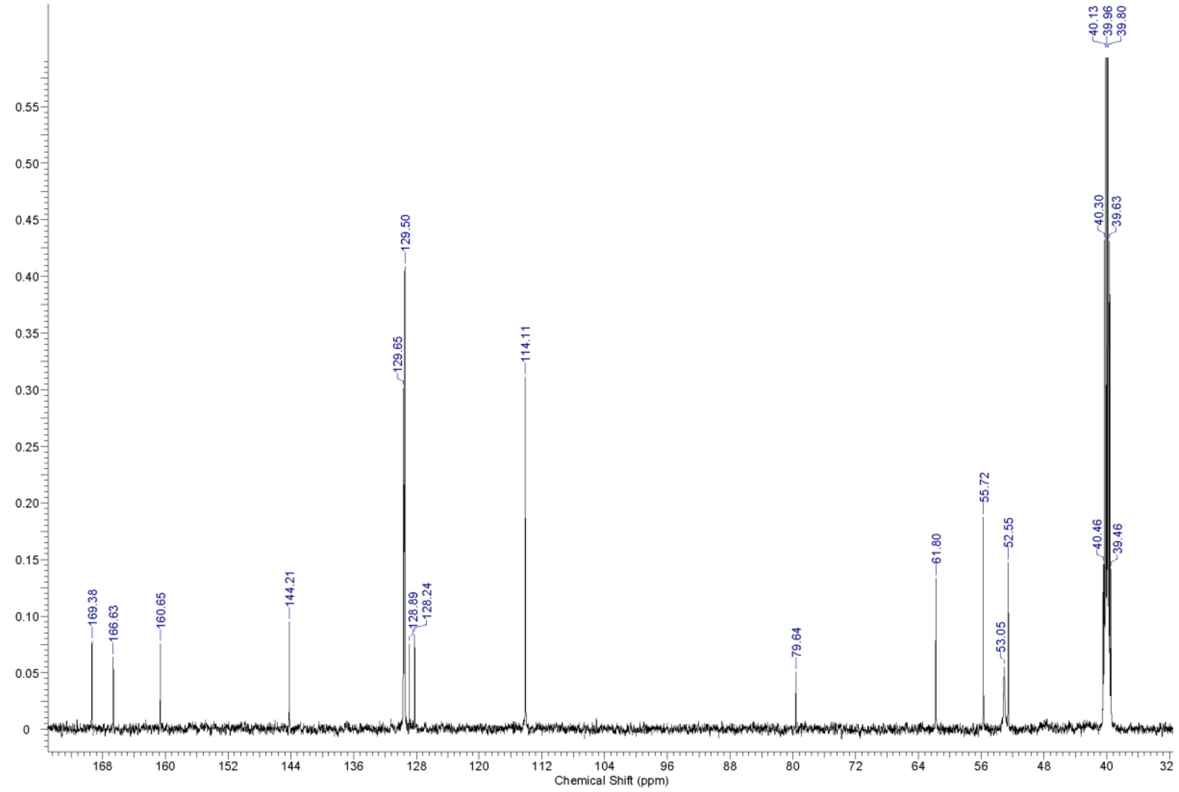

***Methyl 4-((4-(2-fluorobenzoyl)piperazin-1-yl)methyl)benzoate* (26j)** was synthesized from (2-fluorophenyl)(piperazin-1-yl)methanone and methyl 4-formylbenzoate **(1)** as a white solid. The yield was 22%. $^{1}$H-NMR (DMSO-$d_6$ ,500 MHz) $\delta$: 7.94 (d, $J$ = 8.3 Hz, 2H), 7.48 (d, $J$ = 8.3 Hz, 3H), 7.37 - 7.42 (m, 1H), 7.27 - 7.33 (m, 2H), 3.86 (s, 3H), 3.63 - 3.73 (m, 2H), 3.60 (s, 2H), 3.19 - 3.28 (m, 2H), 2.40 - 2.50 (m, 2H), 2.30 - 2.39 (m, 2H); $^{13}$C-NMR (DMSO-$d_6$, 126 MHz) $\delta$: 166.6, 164.4, 159.0, 157.0, 144.1, 131.9, 129.6, 129.5, 129.2, 128.9, 125.4, 116.3, 61.7, 53.2, 52.7, 52.6, 47.0, 41.8.

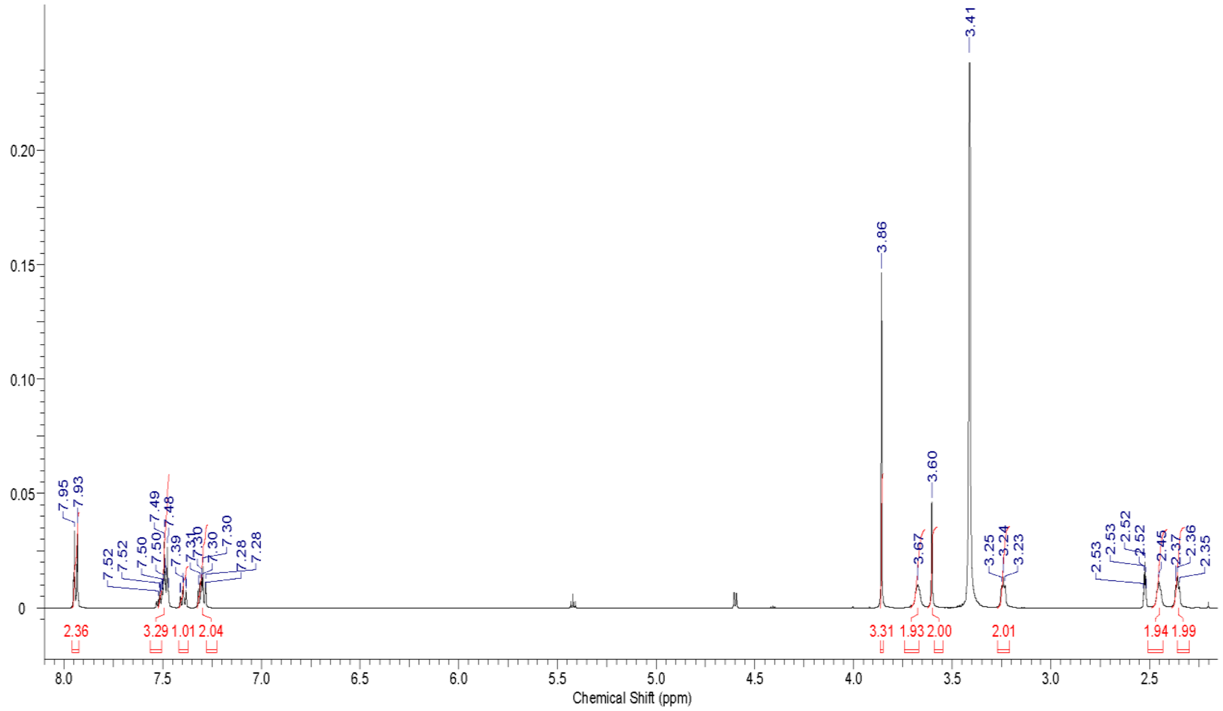


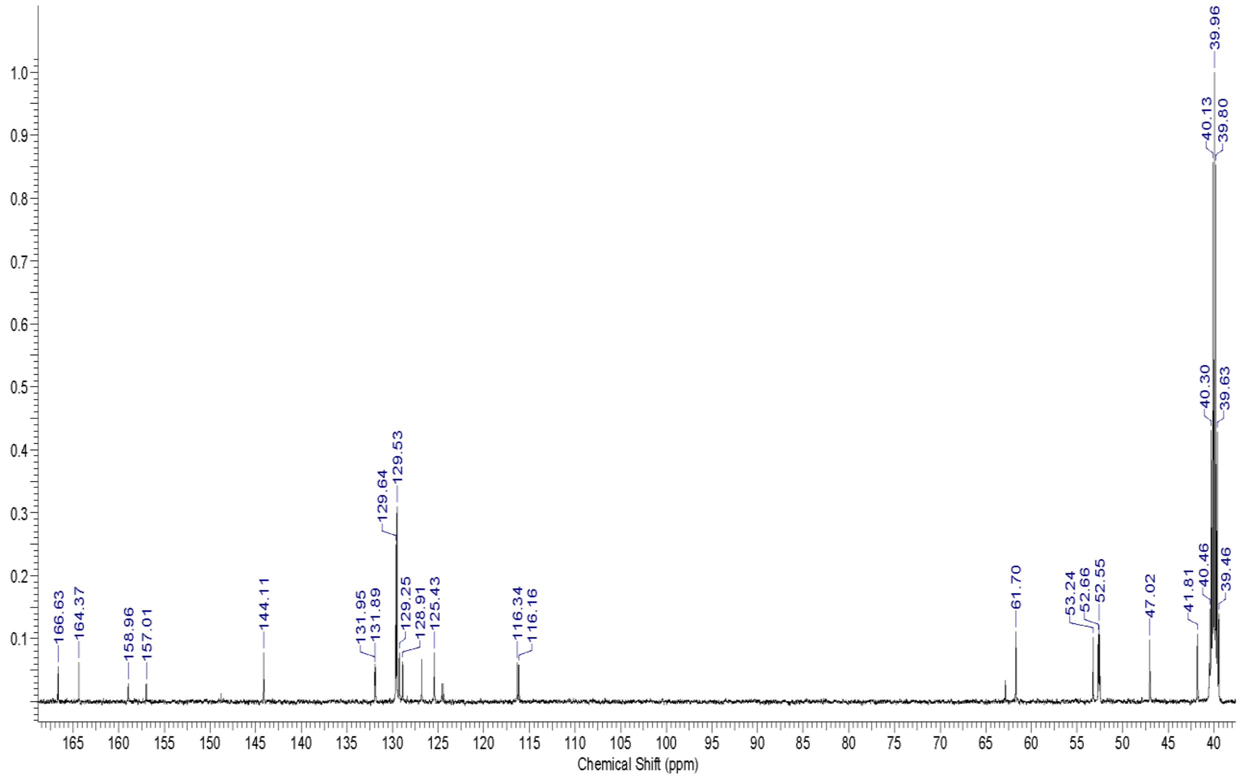

***Methyl 4-((4-(3-fluorobenzoyl)piperazin-1-yl)methyl)benzoate*** **(26k)** was synthesized from (3-fluorophenyl)(piperazin-1-yl)methanone and methyl 4-formylbenzoate **(1)** as a white solid. The yield was 19%. $^{1}$H-NMR (DMSO-*d*$_6$, 500 MHz) $\delta$: 7.94 (d, *J* = 8.3 Hz, 2H), 7.46 - 7.53 (m, 3H), 7.28 - 7.33 (m, 1H), 7.21 - 7.26 (m, 2H), 3.86 (s, 3H), 3.64 (br.s, 2H), 3.60 (s, 2H), 3.33 (br.s, 2H), 2.45 (br.s, 2H), 2.37 (br.s, 2H); $^{13}$C-NMR (DMSO-*d*$_6$, 126 MHz) $\delta$: 167.9, 166.6, 163.3, 161.3, 144.2, 131.2, 129.7, 129.5, 128.9, 123.4, 116.9, 114.4, 61.7, 53.2, 52.5, 47.5, 42.0.

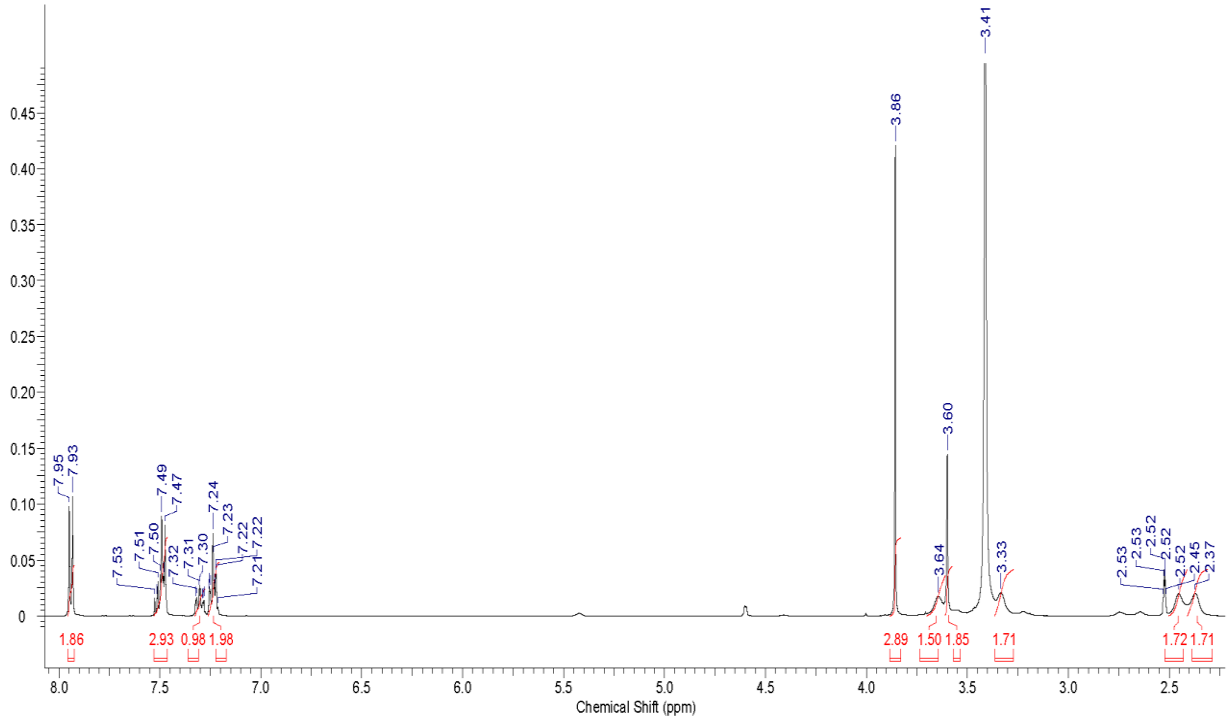


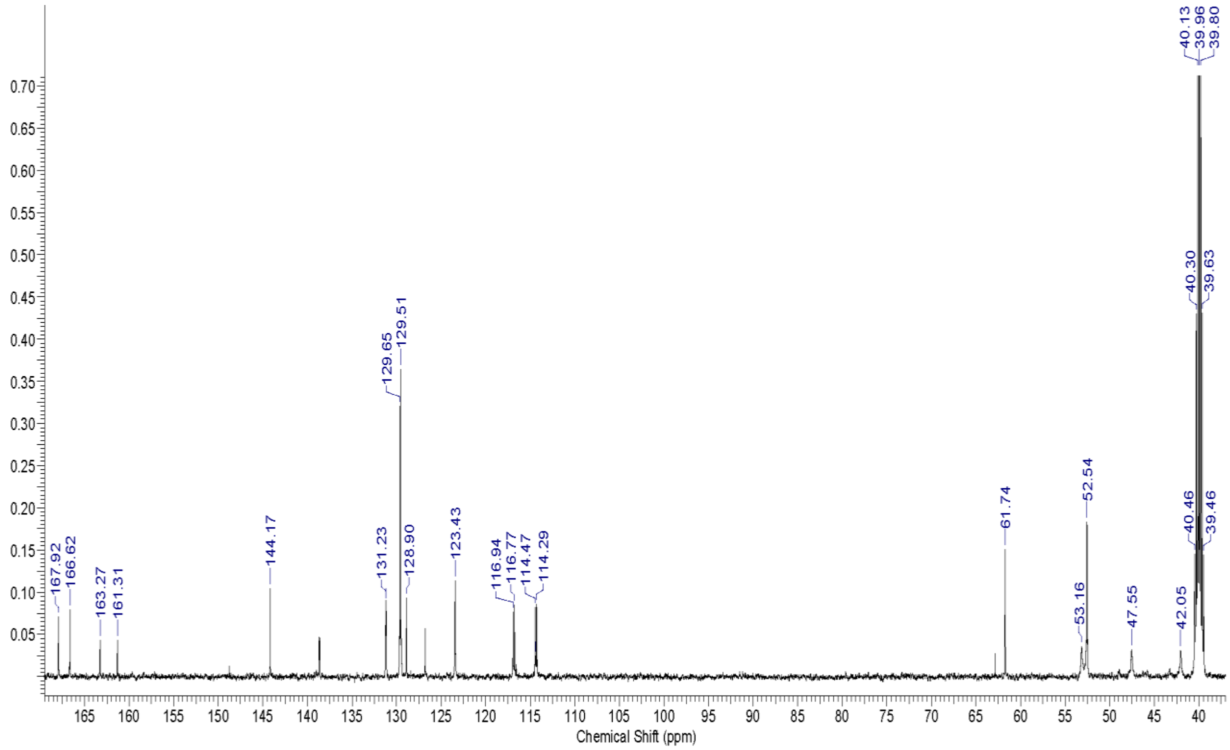

***Methyl 4-((4-nicotinoylpiperazin-1-yl)methyl)benzoate*** **(26l)** was synthesized from piperazin-1-yl(pyridin-3-yl)methanone and methyl 4-formylbenzoate (**1**) as a white solid. The yield was 83%. $^{1}$H-NMR (DMSO-$d_6$, 500 MHz) $\delta$: 8.65 (dd, $J$ = 4.8, 1.6 Hz, 1H), 8.61 (d, $J$ = 1.3 Hz, 1H), 7.94 (d, $J$ = 8.3 Hz, 2H), 7.84 (dt, $J$ = 7.7, 1.9 Hz, 1H), 7.47 - 7.51 (m, 3H), 3.86 (s, 3H), 3.67 (br.s, 2H), 3.61 (s, 2H), 3.36 (br.s, 2H), 2.47 (br.s, 2H), 2.39 (br.s, 2H); $^{13}$C-NMR (DMSO-$d_6$ ,126 MHz) $\delta$: 167.2, 166.6, 150.9, 148.0, 144.2, 135.3, 132.2, 129.7, 129.5, 128.9, 124.0, 61.7, 53.2, 52.6, 47.7, 42.1.

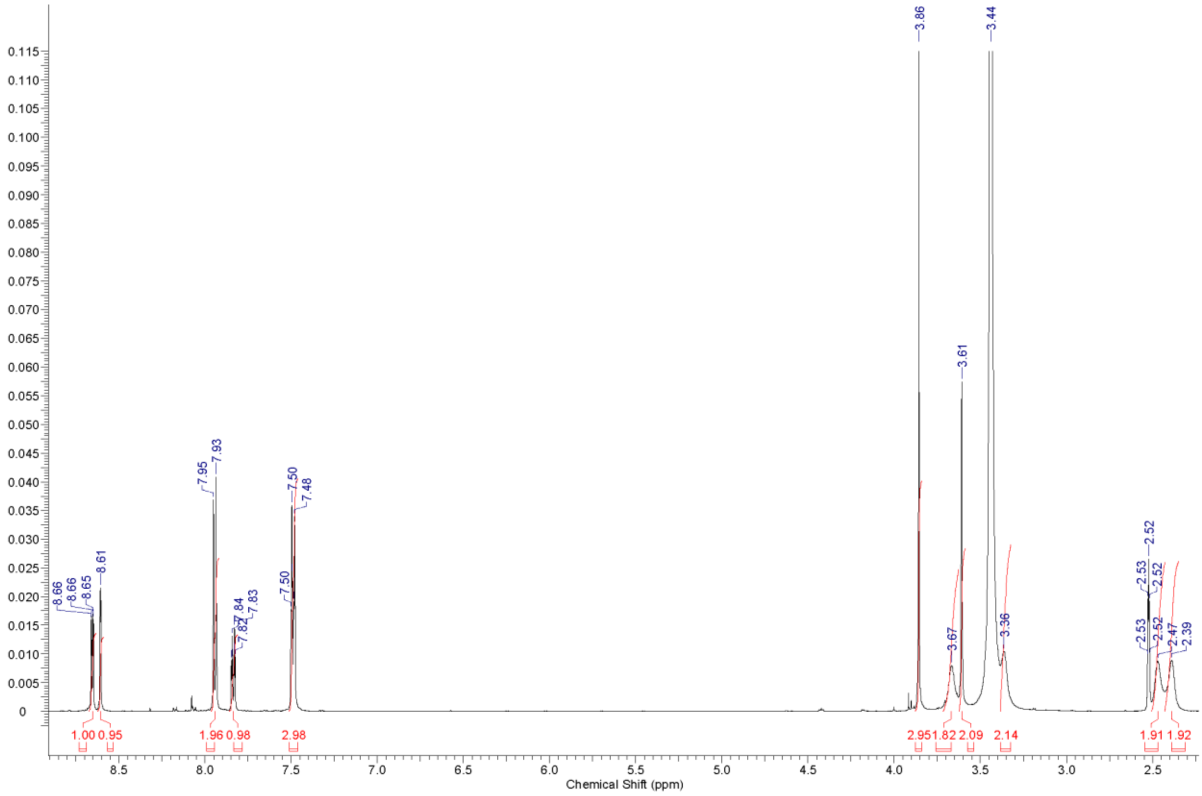


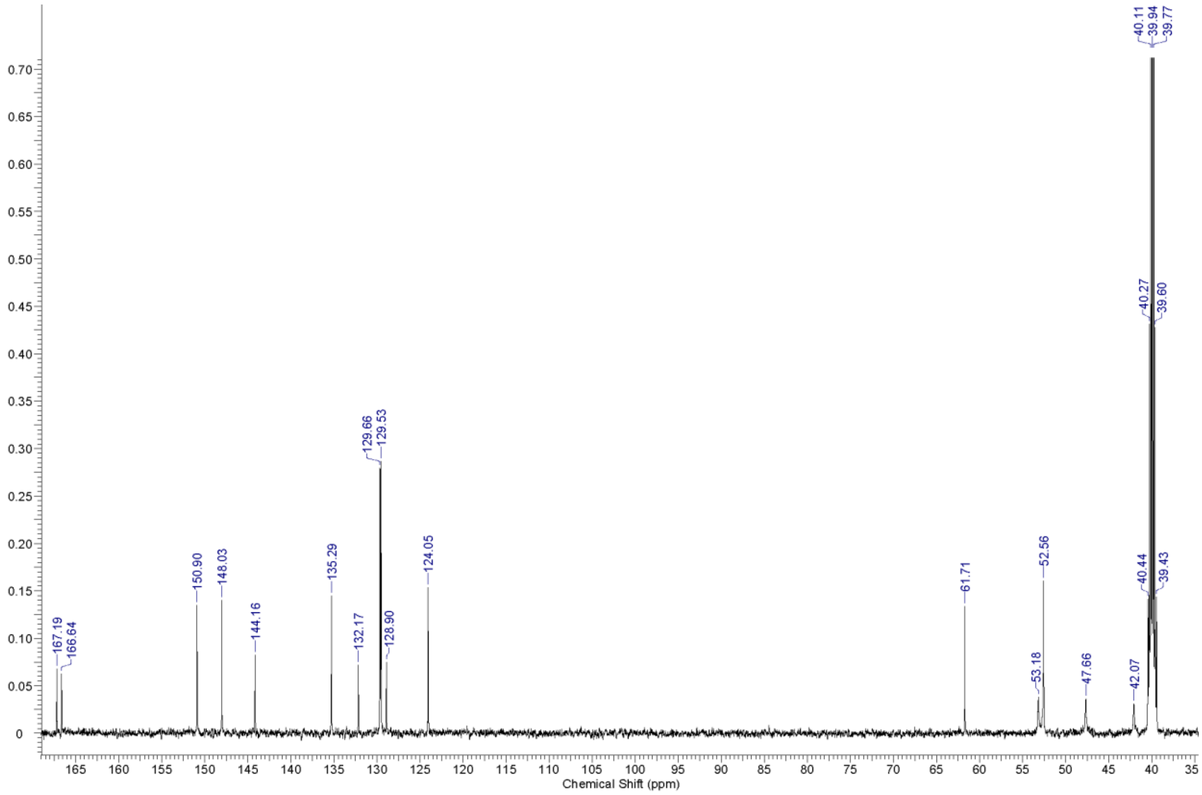

***4-((4-(4-methoxybenzoyl)piperazin-1-yl)methyl)benzoic acid* (27i)** was synthesized from compound **26i** as a white solid. The yield was 60%. $^{1}$H-NMR (DMSO-*d*$_6$, 500 MHz) δ: 7.92 (d, *J* = 8.3 Hz, 2H), 7.46 (d, *J* = 8.3 Hz, 2H), 7.34 - 7.39 (m, 2H), 6.96 - 7.02 (m, 2H), 3.80 (s, 3H), 3.53 - 3.70 (m, 6H), 2.41 (br.s, 4H); $^{13}$C-NMR (DMSO-*d*$_6$, 126 MHz) δ: 169.4, 167.7, 160.6, 143.6, 130.1, 129.8, 129.5, 129.4, 128.2, 114.1, 79.6, 61.9, 55.7, 53.1.

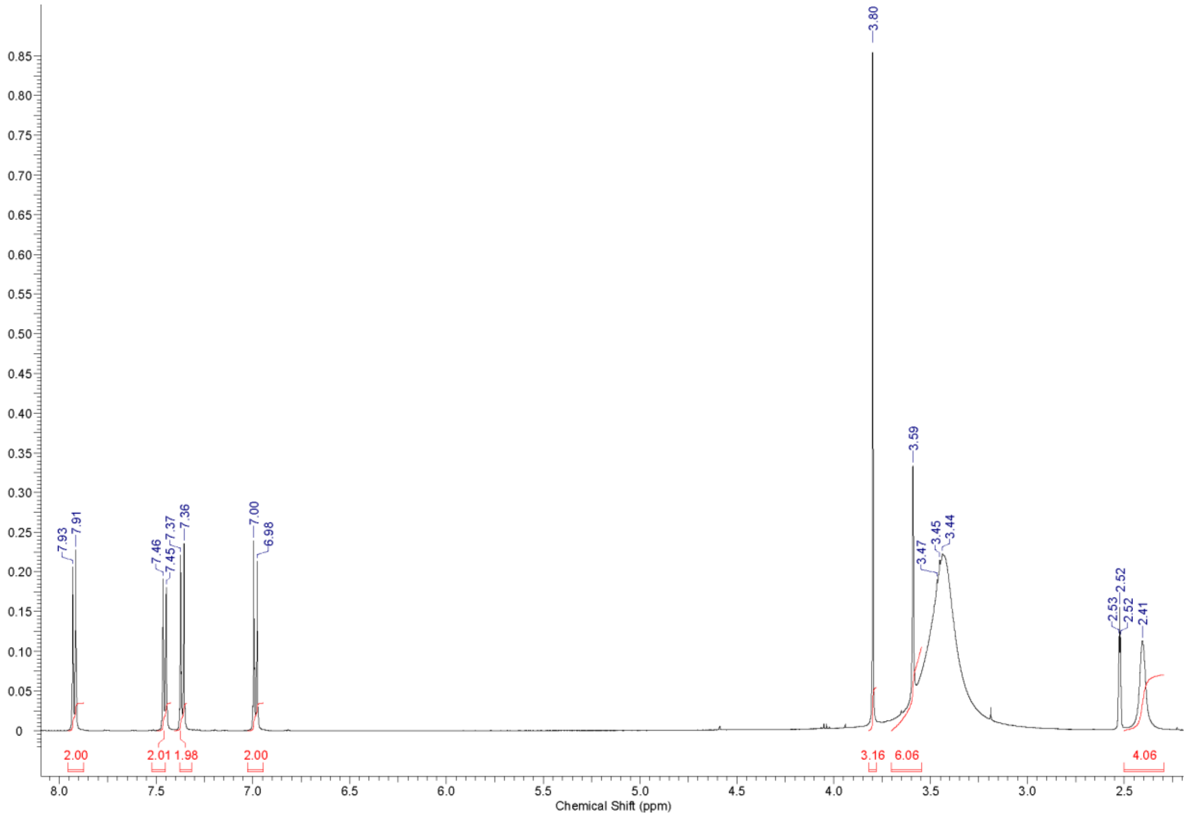


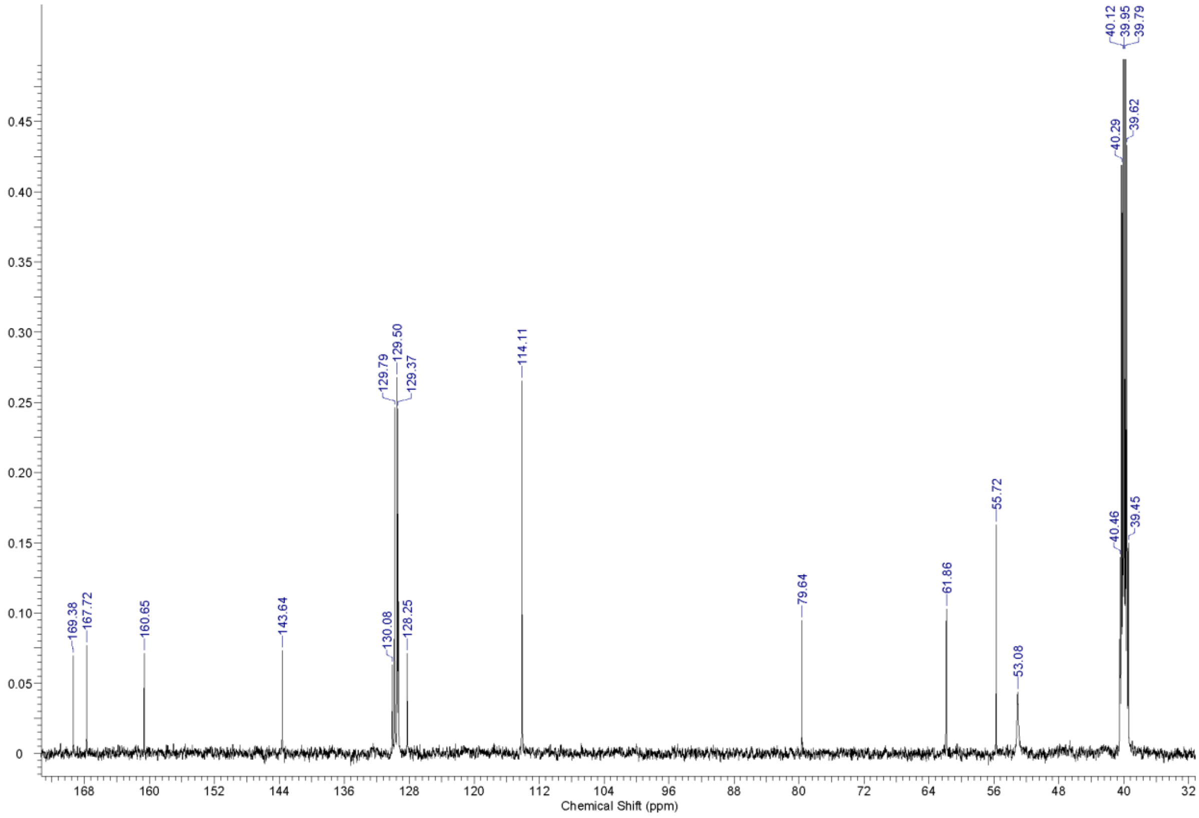

***4-((4-(2-fluorobenzoyl)piperazin-1-yl)methyl)benzoic acid*** **(27j)** was synthesized from compound **26j** as a white solid. The yield was 60%. $^{1}$H-NMR (DMSO-$d_6$, 500 MHz) δ: 7.93 (s, 2H), 7.48 - 7.54 (m, 1H), 7.45 (d, *J* = 8.3 Hz, 2H), 7.40 (td, *J* = 7.3, 1.8 Hz, 1H), 7.27 - 7.33 (m, 2H), 3.67 (br.s, 2H), 3.58 - 3.61 (m, 2H), 3.22 - 3.26 (m, 2H), 2.45 (br.s, 2H), 2.36 (d, *J* = 9.0 Hz, 2H); $^{13}$C-NMR (DMSO-$d_6$, 126 MHz) δ: 167.7, 164.4, 159.0, 157.0, 143.5, 131.9, 130.1, 129.8, 129.4, 129.2, 125.4, 116.3, 61.8, 53.2, 52.7, 47.0, 41.8.

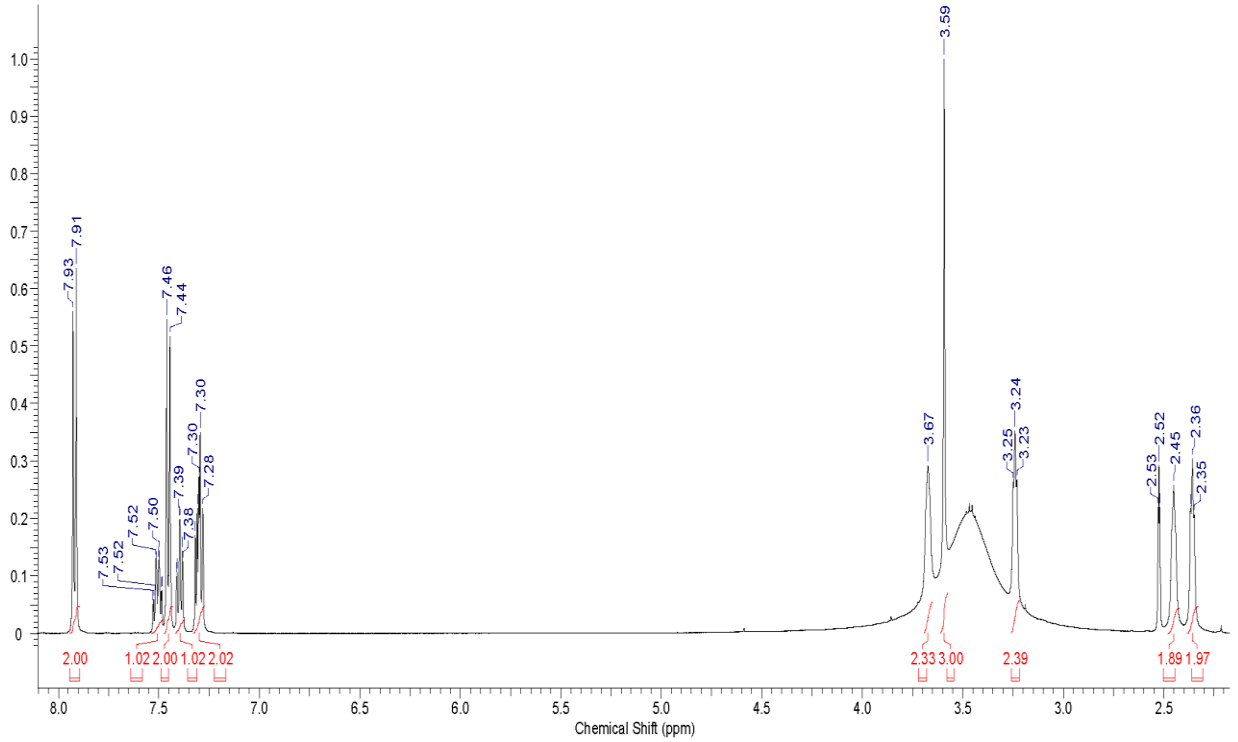


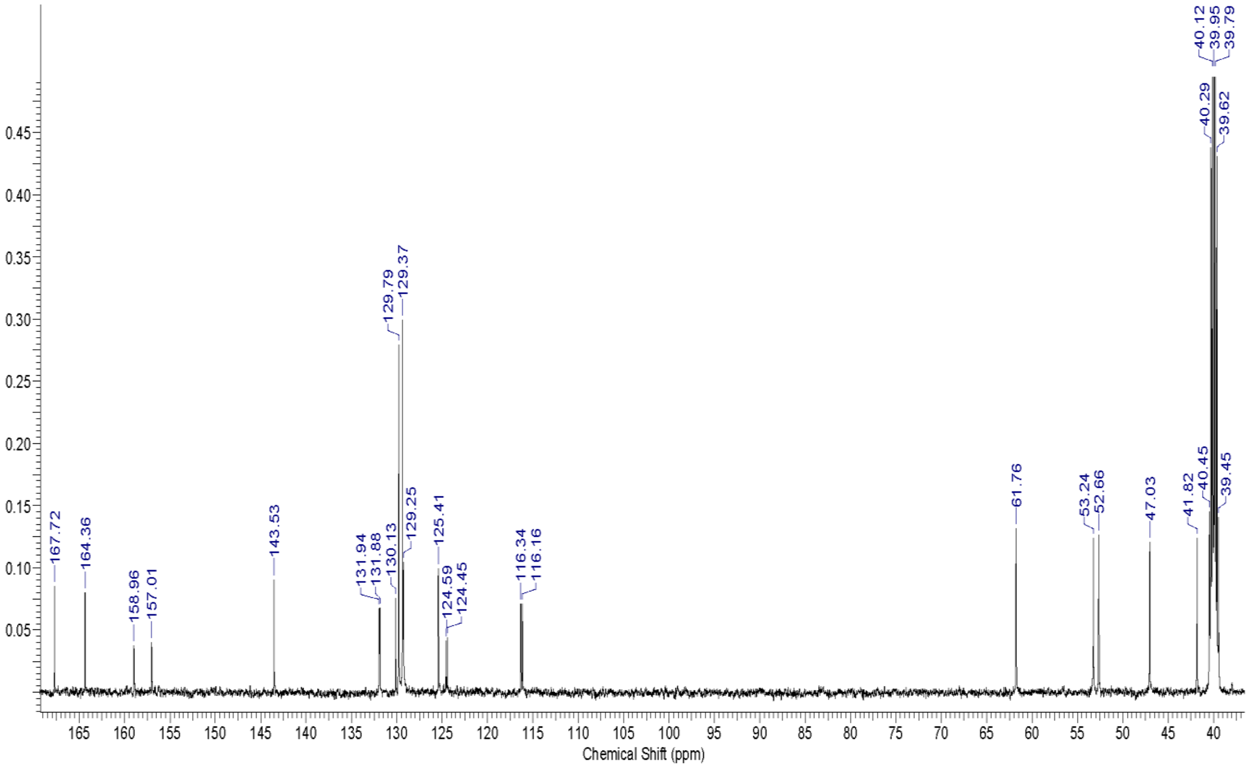

***4-((4-(3-fluorobenzoyl)piperazin-1-yl)methyl)benzoic acid*** **(27k)** was synthesized from compound **26k** as a white solid. The yield was 53%. $^{1}$H-NMR (DMSO-$d_6$, 500 MHz) δ: 7.92 (d, *J* = 8.3 Hz, 2H), 7.50 (td, *J* = 7.9, 5.9 Hz, 1H), 7.45 (d, *J*=8.0 Hz, 2H), 7.27 - 7.34 (m, 1H), 7.21 - 7.26 (m, 2H), 3.64 (br.s, 2H), 3.59 (s, 2H), 3.33 (br.s, 2H), 2.45 (br.s, 2H), 2.38 (br.s, 2H); $^{13}$C-NMR (DMSO-$d_6$ ,126 MHz) δ: 167.9, 167.7, 163.3, 161.3, 143.6, 131.2, 130.2, 129.8, 129.4, 123.4, 116.9, 114.4, 61.8, 53.1, 52.6, 47.5, 42.1.

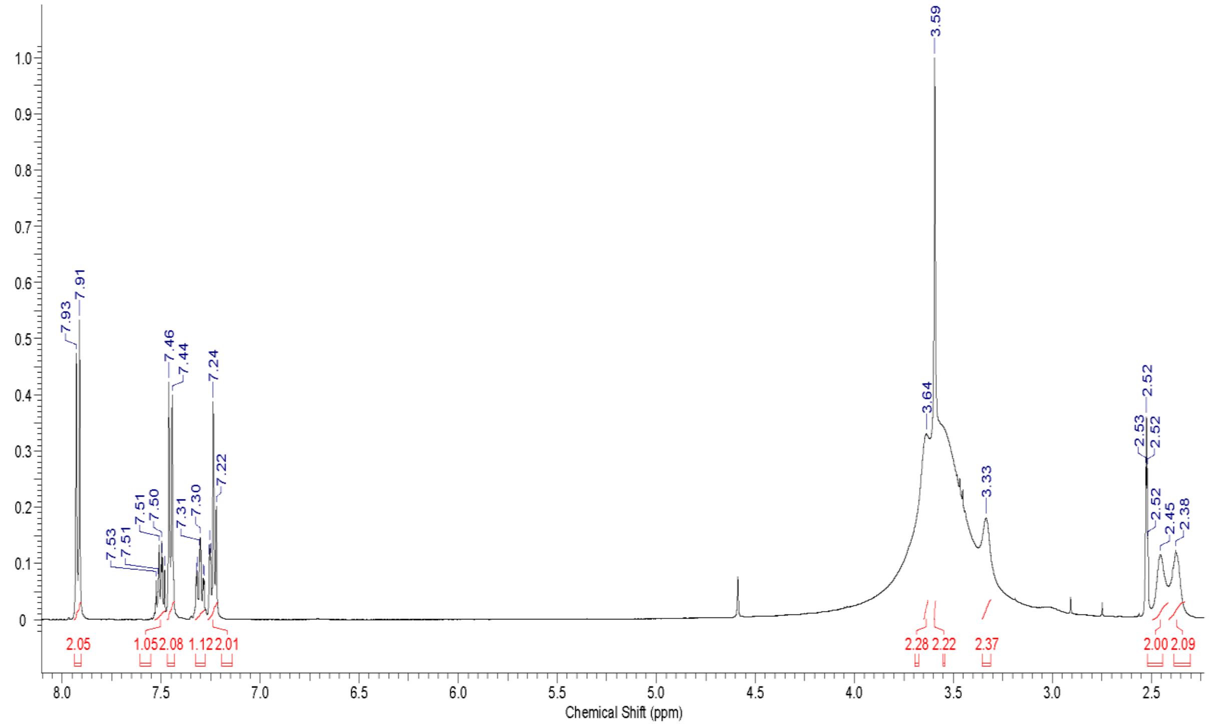


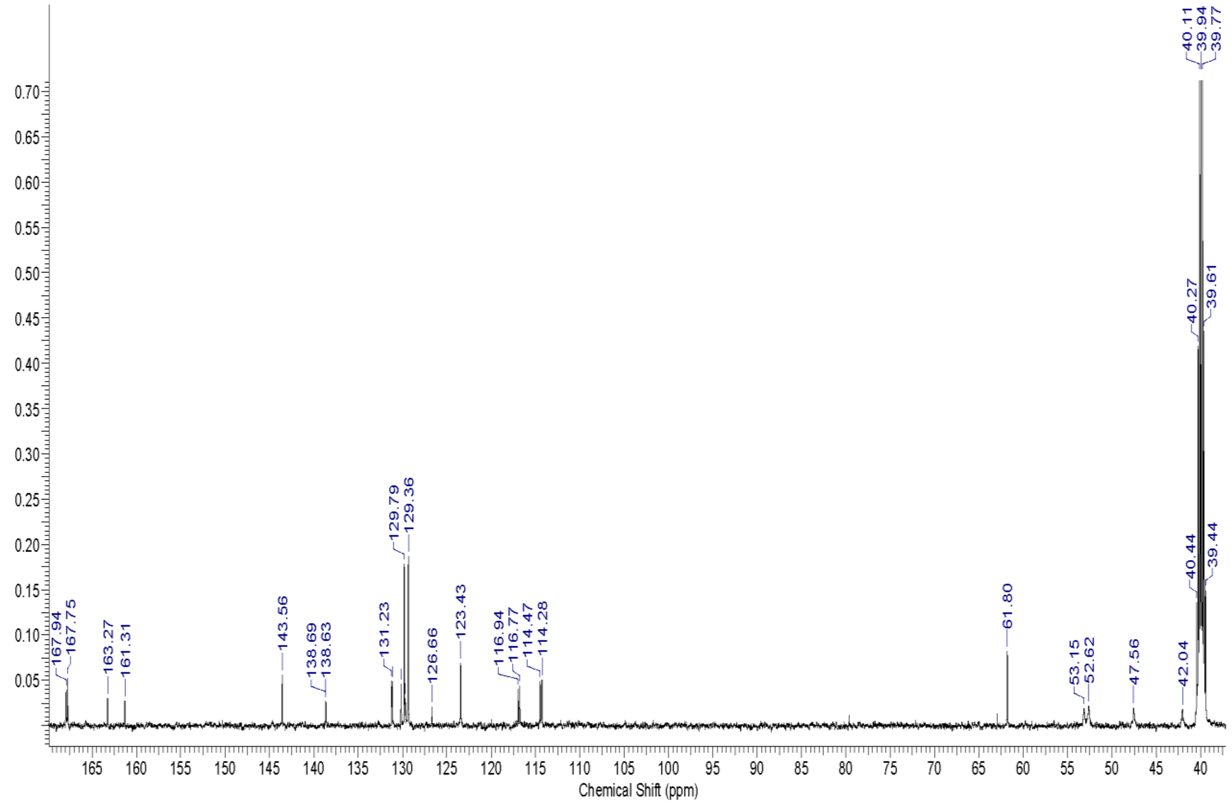

***4-((4-nicotinoylpiperazin-1-yl)methyl)benzoic acid* (27l)** was synthesized from methyl 4-((4-nicotinoylpiperazin-1-yl)methyl)benzoate **26l** as a white solid. The yield was 72%. $^{1}$H-NMR (DMSO-$d_6$, 500 MHz) $\delta$: 8.65 - 8.67 (m, 1H), 8.61 (d, $J$ = 1.6 Hz, 1H), 7.92 (d, $J$ = 8.3 Hz, 2H), 7.84 (dt, $J$ = 7.7, 1.9 Hz, 1H), 7.47 - 7.50 (m, 1H), 7.46 (d, $J$ = 8.3 Hz, 2H), 3.67 (br.s, 2H), 3.60 (s, 2H), 3.36 (br.s, 2H), 2.47 (br.s, 2H), 2.39 (br.s, 2H); $^{13}$C-NMR (DMSO-$d_6$, 126 MHz) $\delta$: 167.7, 167.2, 150.9, 148.0, 143.6, 135.3, 132.2, 130.1, 129.8, 129.4, 124.0, 61.8, 53.2, 52.6, 47.7, 42.1.

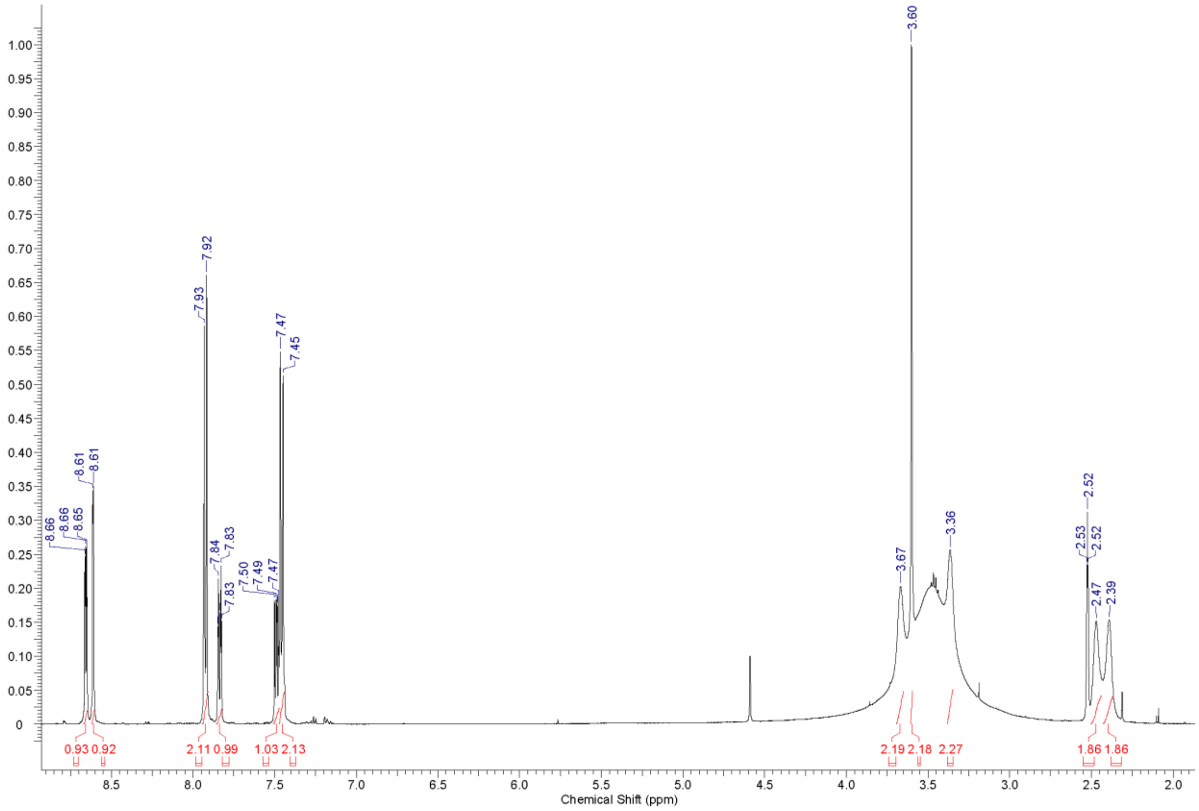


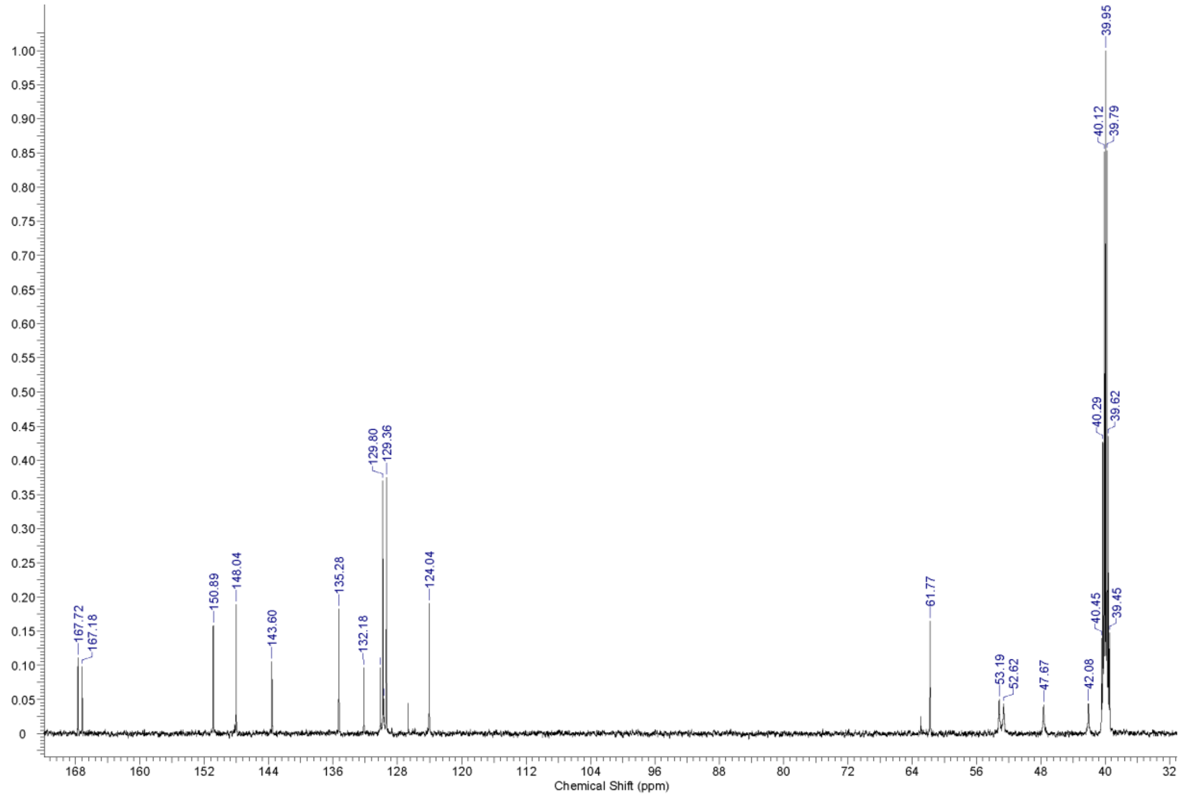

***4-{[4-(4-methoxybenzoyl)piperazin-1-yl]methyl}-N-[3-(4-methyl-1H-imidazol-1-yl)-5-(trifluoromethyl)phenyl]benzamide*** **(28i)** was synthesized from compound **27i** and 3-(4-methyl-1H-imidazol-1-yl)-5-(trifluoromethyl)aniline **(a)** as a white solid. M.p. 98-102 °C. $^{1}$H-NMR (DMSO-$d_6$, 500 MHz) $\delta$: 10.69 (s, 1H), 8.30 (s, 1H), 8.22 (d, $J$ = 1.0 Hz, 1H), 8.18 (s, 1H), 7.98 (d, $J$ = 8.3 Hz, 2H), 7.75 (s, 1H), 7.53 (d, $J$ = 8.0 Hz,  H), 7.50 (s, 1H), 7.34 - 7.40 (m, $J$ = 8.7 Hz, 2H), 6.97 - 7.02 (m, $J$ = 9.0 Hz, 2H), 3.80 (s, 3H), 3.62 (s, 2H), 3.45 - 3.60 (m, 4H), 2.37 - 2.47 (m, 4H), 2.20 (s, 3H); $^{13}$C-NMR (DMSO-$d_6$, 126 MHz) $\delta$: 169.4, 166.4, 160.7, 142.9, 141.8, 139.4, 138.4, 135.5, 133.3, 129.5, 129.4, 128.3, 115.4, 114.7, 114.1, 61.8, 55.7, 14.0. HRMS(ESI+) m/z calcd for $C_{31}H_{30}F_3N_5O_3$ [M + H]+ 578.2301, found 578.2362. Purity: 95.81% (by HPLC).

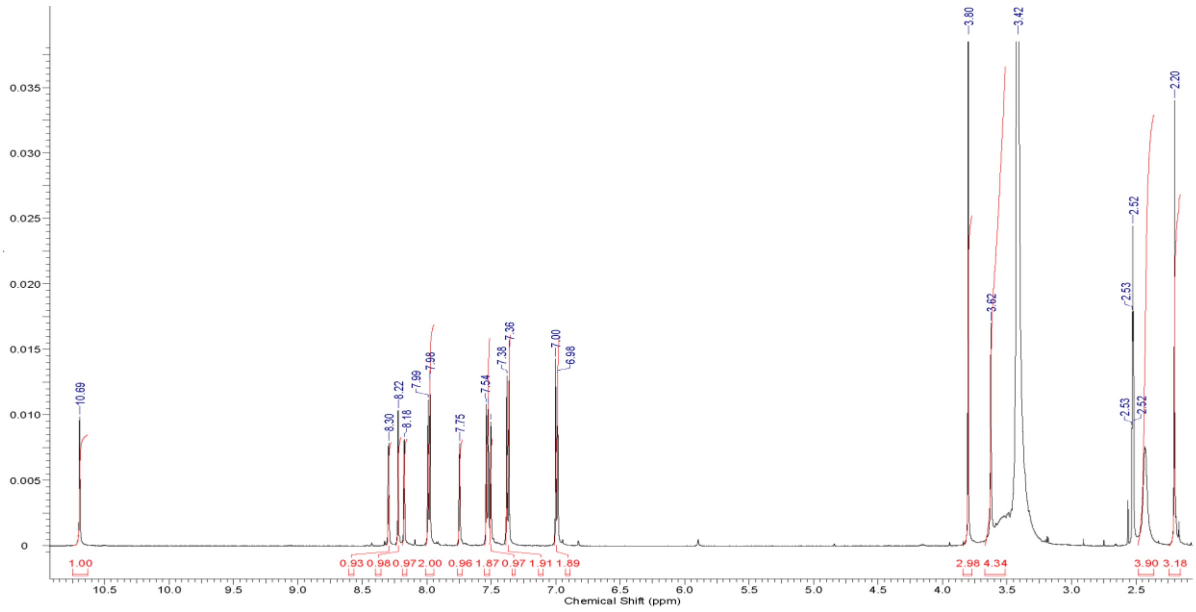


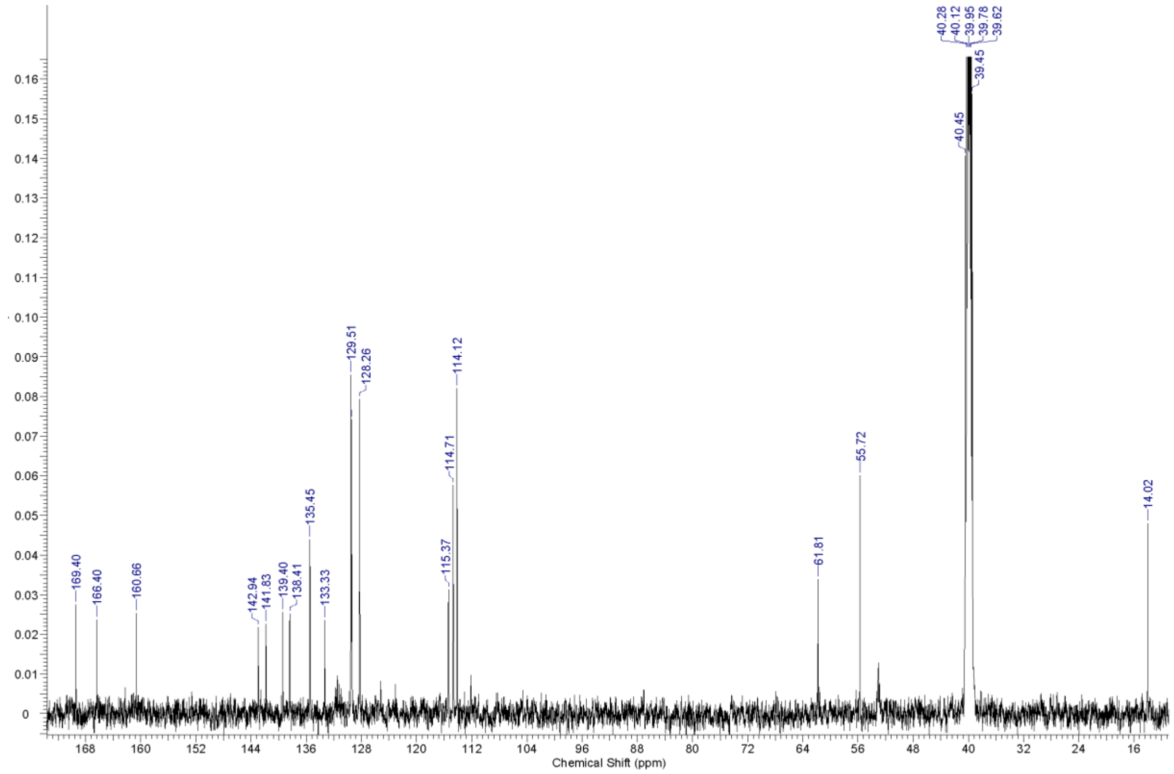

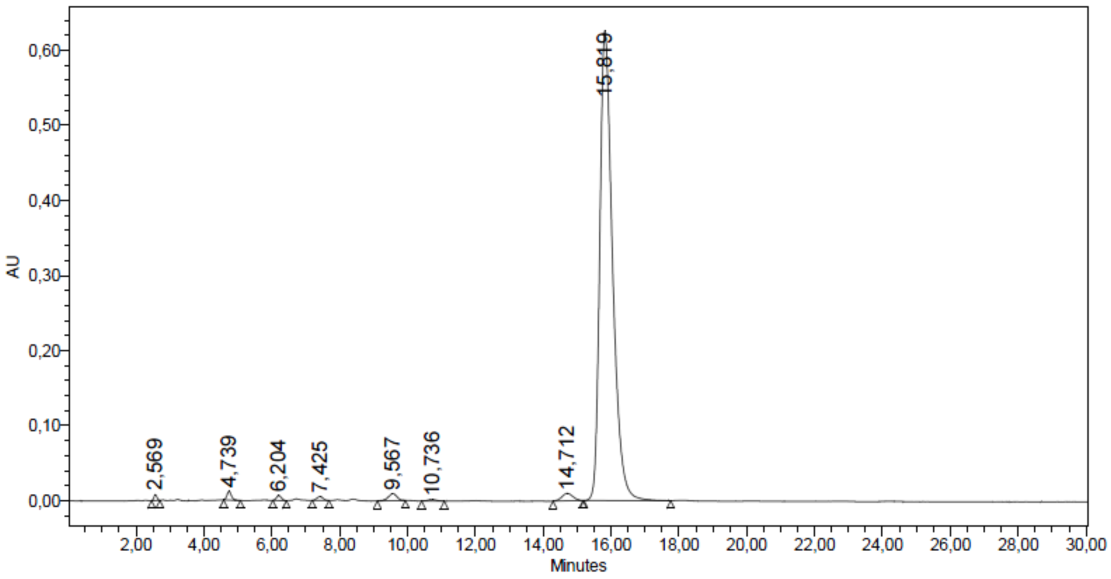


| | RT | Area | % Area | Height |
|---|---|---|---|---|
| 1 | 2,569 | 48998 | 0,29 | 7922 |
| 2 | 4,739 | 105481 | 0,62 | 11718 |
| 3 | 6,204 | 65451 | 0,38 | 6501 |
| 4 | 7,425 | 66677 | 0,39 | 5181 |
| 5 | 9,567 | 167759 | 0,98 | 9535 |
| 6 | 10,736 | 33439 | 0,20 | 2021 |
| 7 | 14,712 | 225982 | 1,33 | 9915 |
| 8 | 15,819 | 16330072 | 95,81 | 625602 |

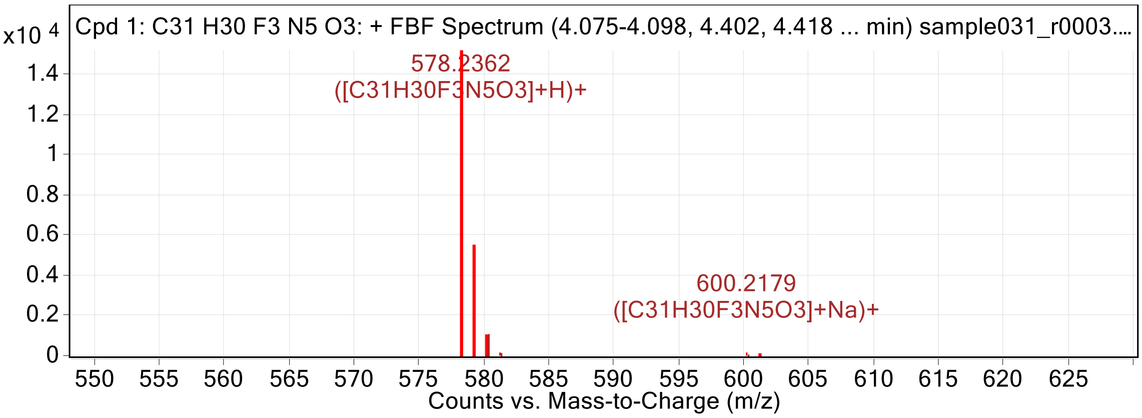

***4-{[4-(2-fluorobenzoyl)piperazin-1-yl]methyl}-N-[3-(4-methyl-1H-imidazol-1-yl)-5-(trifluoromethyl)phenyl]benzamide*** **(28j)** was synthesized from compound **27j** and 3-(4-methyl-1H-imidazol-1-yl)-5-(trifluoromethyl)aniline **(a)** as a white solid. M.p. 106-109 °C. $^{1}$H-NMR (DMSO-*d*$_6$, 500 MHz) δ: 10.68 (s, 1H), 8.31 (s, 1H), 8.22 (d, *J* = 1.0 Hz, 1H), 8.17 (s, 1H), 7.99 (d, J = 8.3 Hz, 2H), 7.75 (s, 1H), 7.49 - 7.55 (m, 4H), 7.38 - 7.43 (m, 1H), 7.28 - 7.34 (m, 2H), 3.69 (br.s, 2H), 3.63 (s, 2H), 3.26 (t, *J* = 4.3 Hz, 2H), 2.48 (br.s, 2H), 2.39 (d, *J* = 4.5 Hz, 2H), 2.20 (s, 3H); $^{13}$C-NMR (DMSO-*d*$_6$, 126 MHz) δ: 166.4, 164.4, 159.1, 157.0, 142.9, 141.8, 139.4, 138.4, 135.5, 133.4, 132.0, 129.4, 128.3, 125.4, 116.4, 116.2, 115.4, 114.7, 112.1, 79.7, 61.7, 53.3, 52.7, 47.1, 41.8, 14.1. HRMS(ESI+) m/z calcd for $C_{30}H_{27}F_4N_5O_2$ [M + H]+ 566.2101, found 566.2176. Purity: 95.05% (by HPLC).

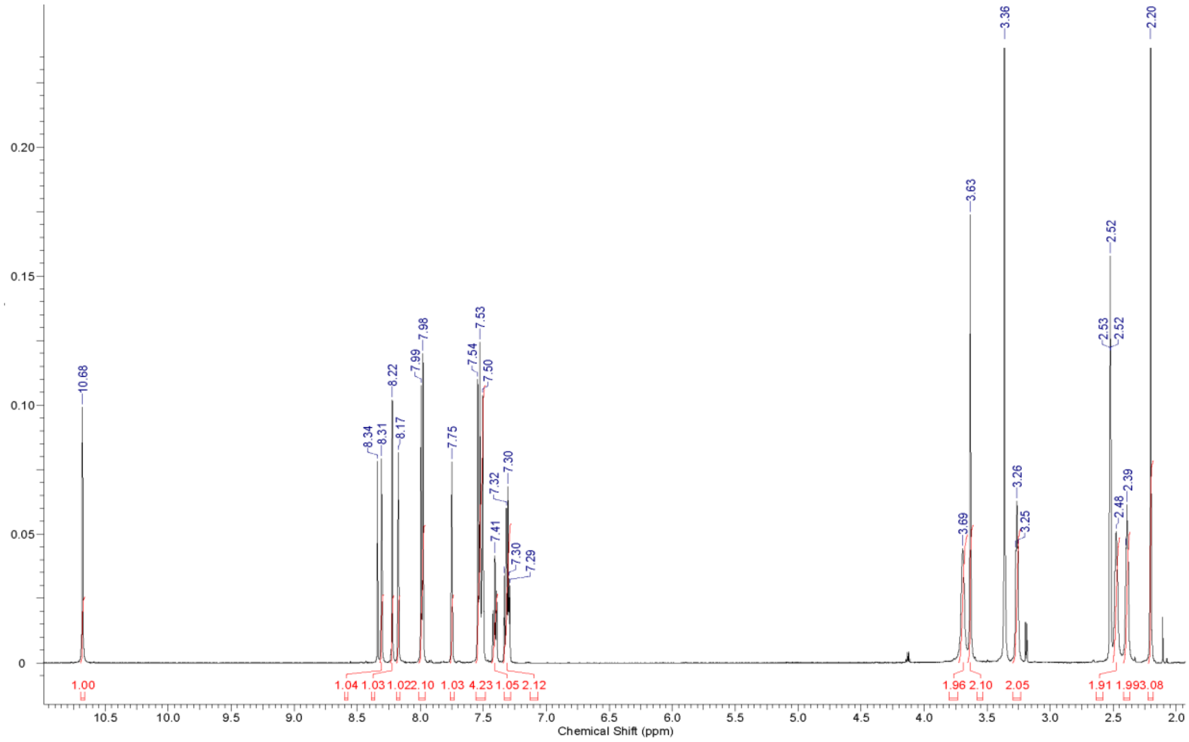


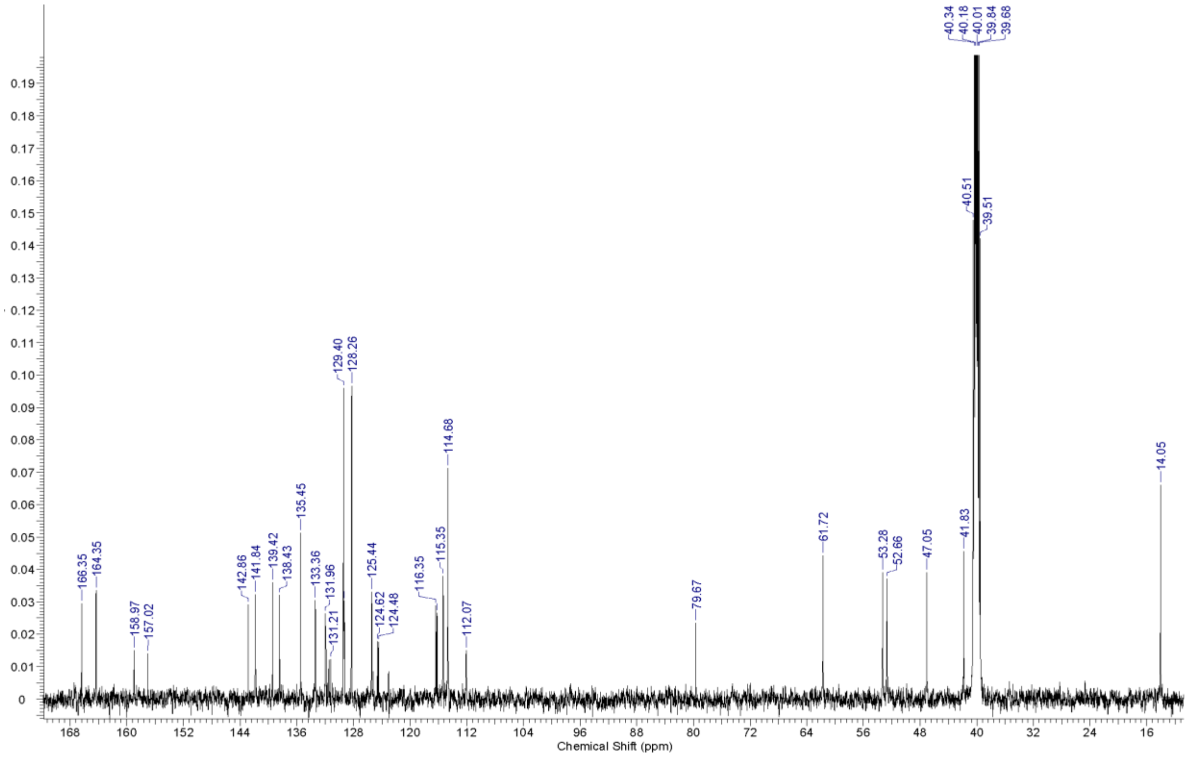

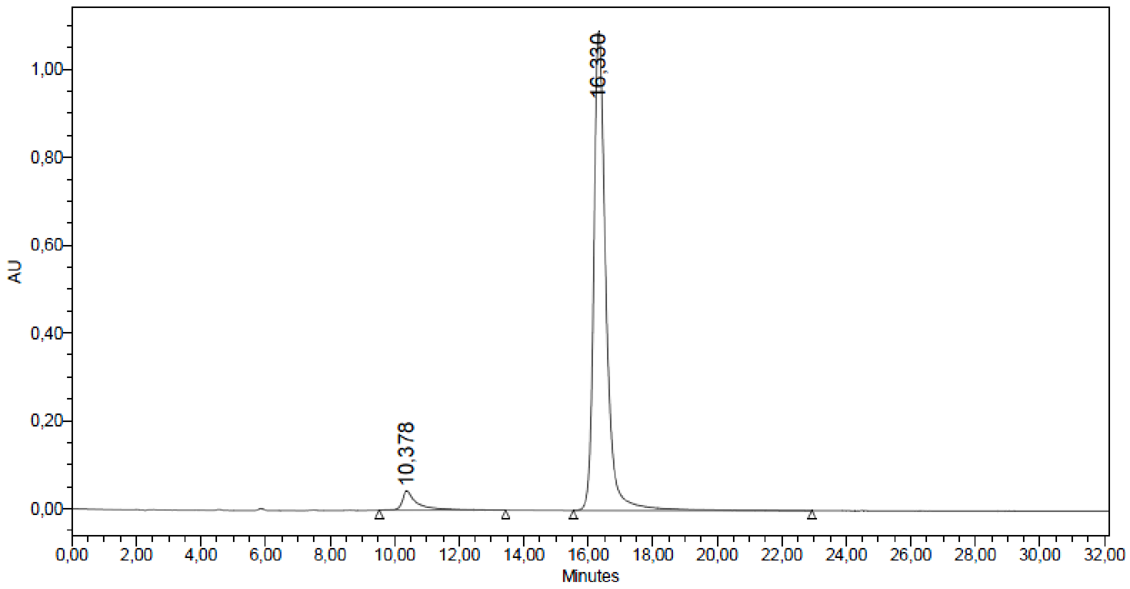


| | RT | Area | % Area | Height |
|---|---|---|---|---|
| 1 | 10,378 | 1496970 | 4,95 | 43980 |
| 2 | 16,330 | 28728344 | 95,05 | 1090272 |

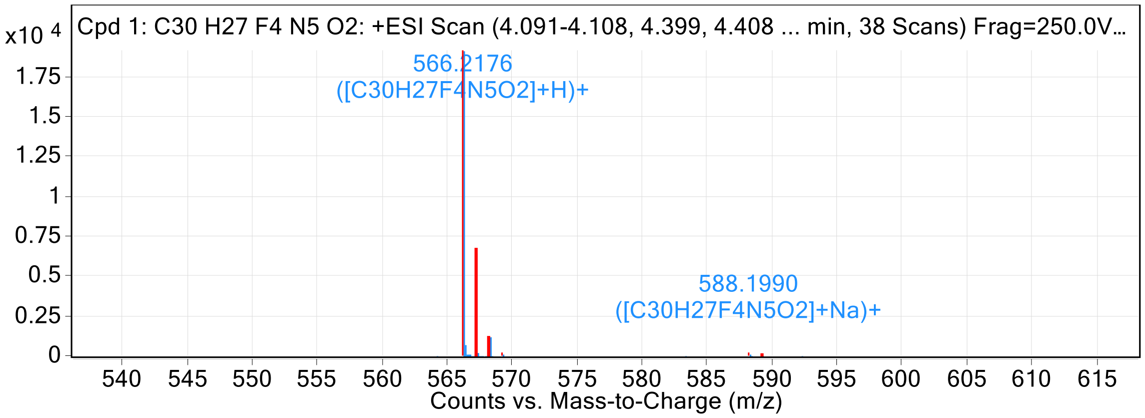

***4-{[4-(3-fluorobenzoyl)piperazin-1-yl]methyl}-N-[3-(4-methyl-1H-imidazol-1-yl)-5-(trifluoromethyl)phenyl]benzamide*** **(28k)** was synthesized from compound **27k** and 3-(4-methyl-1H-imidazol-1-yl)-5-(trifluoromethyl)aniline **(a)** as a light yellow solid. M.p. 90-93 °C. $^{1}$H-NMR (DMSO-*d6*, 500 MHz) $\delta$: 10.69 (s, 1H), 8.31 (s, 1H), 8.22 (d, *J* = 1.3 Hz, 1H), 8.18 (s, 1H), 7.99 (d, *J* = 8.3 Hz, 2H), 7.75 (s, 1H), 7.48 - 7.55 (m, 4H), 7.29 - 7.34 (m, 1H), 7.22 - 7.28 (m, 2H), 3.61 - 3.71 (m, 4H), 3.33 - 3.38 (m, 2H), 2.35 - 2.50 (m, 4H), 2.20 (s, 3H); $^{13}$C-NMR (DMSO-*d6*, 126 MHz) $\delta$: 167.9, 166.4, 163.3, 161.3, 142.9, 141.8, 139.4, 138.4, 135.5, 133.4, 131.2, 129.4, 128.3, 123.4, 116.9, 116.8, 115.3, 114.7, 112.1, 79.7, 61.8, 53.2, 52.6, 47.6, 42.0, 14.1. HRMS(ESI+) m/z calcd for $C_{30}H_{27}F_4N_5O_2$ [M + H]+ 566,2101, found 566.2176. Purity: 94.86% (by HPLC).

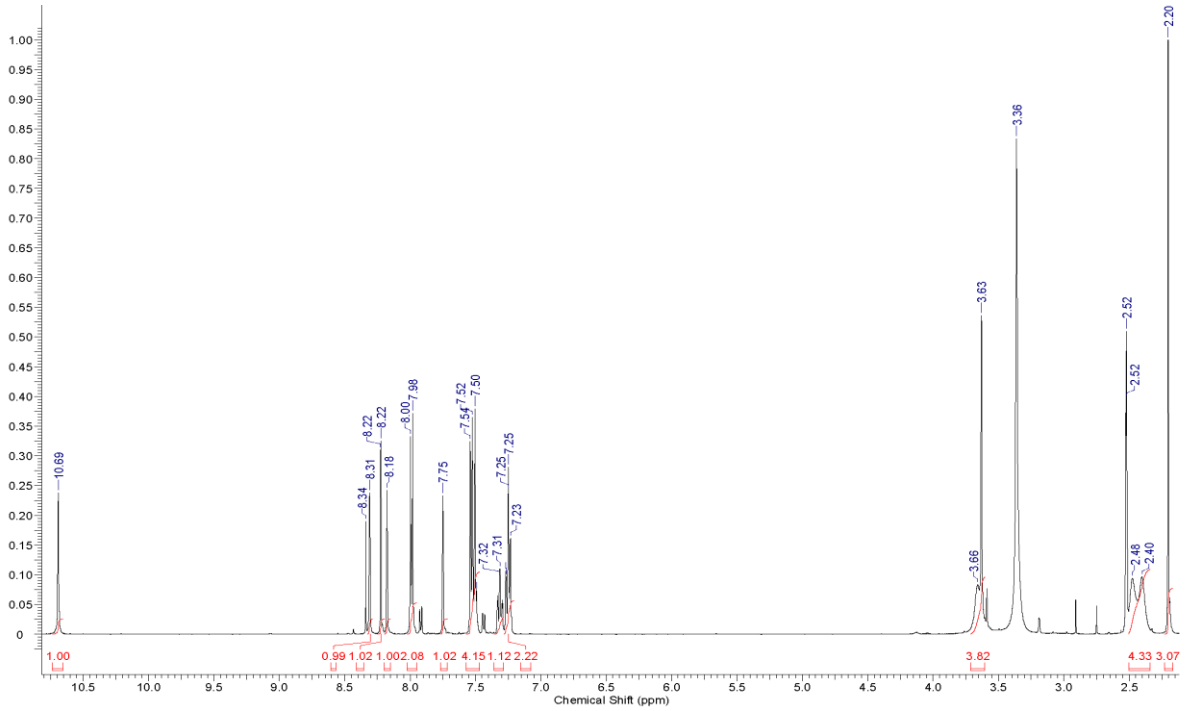


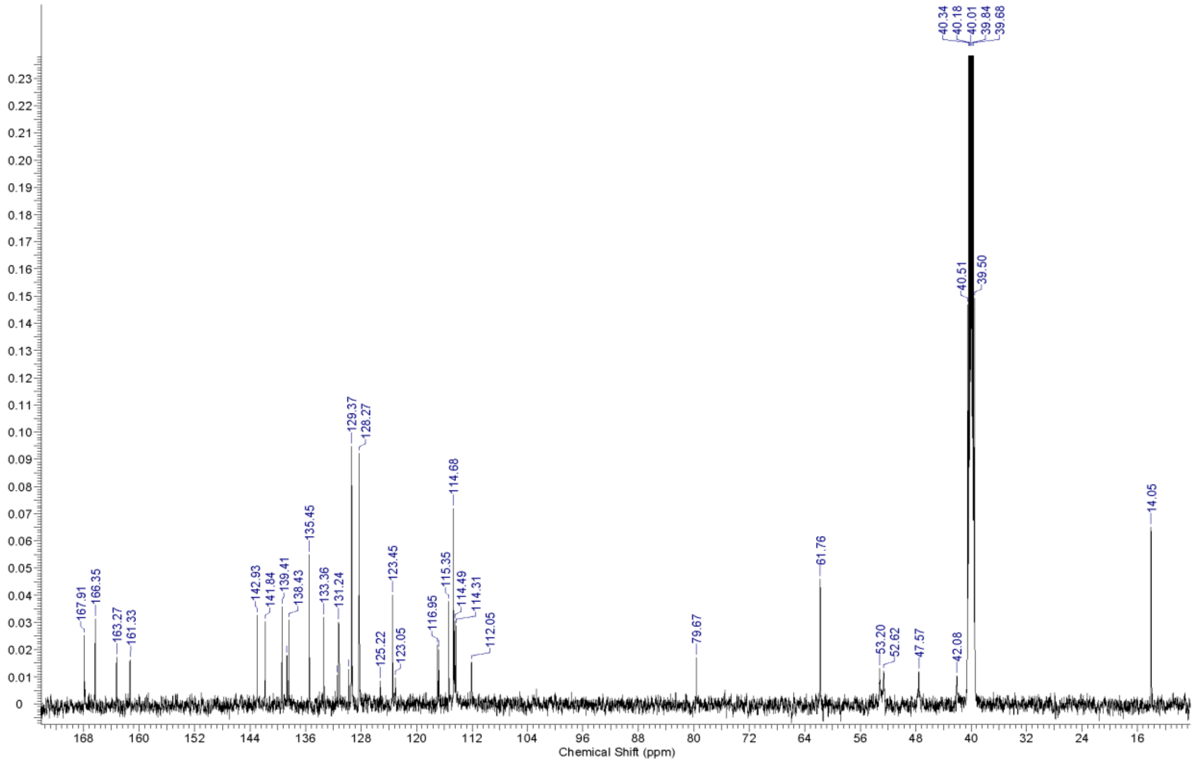

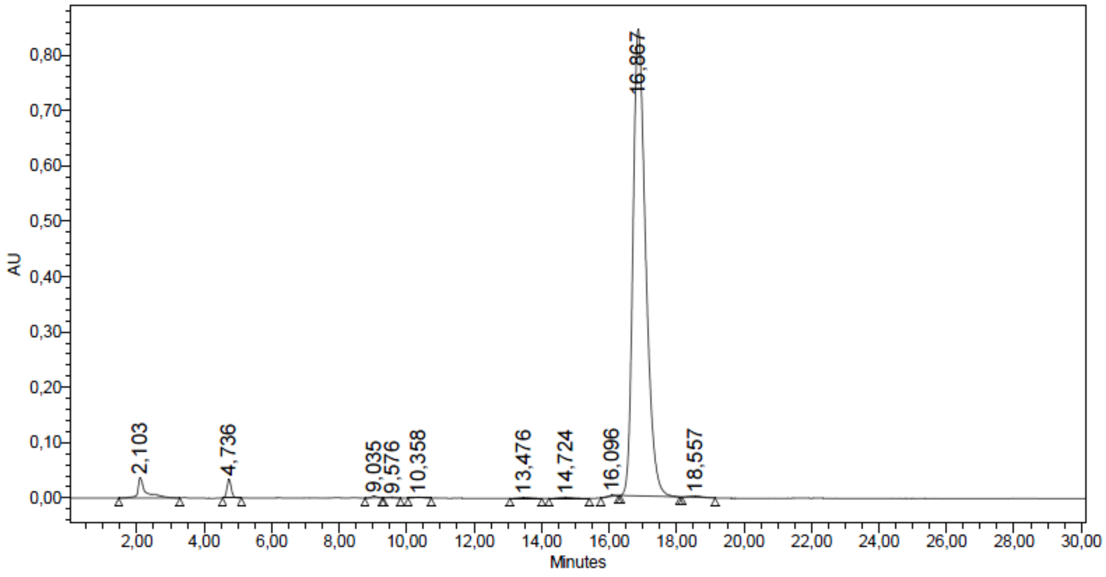


| | RT | Area | % Area | Height |
|---|---|---|---|---|
| 1 | 2,103 | 616348 | 2,66 | 36065 |
| 2 | 4,736 | 295866 | 1,28 | 33396 |
| 3 | 9,035 | 42504 | 0,18 | 3152 |
| 4 | 9,576 | 23525 | 0,10 | 1598 |
| 5 | 10,358 | 29475 | 0,13 | 1622 |
| 6 | 13,476 | 38224 | 0,16 | 1668 |
| 7 | 14,724 | 45176 | 0,19 | 1876 |
| 8 | 16,096 | 32032 | 0,14 | 2122 |
| 9 | 16,867 | 22001794 | 94,86 | 842345 |
| 10 | 18,557 | 68116 | 0,29 | 2611 |

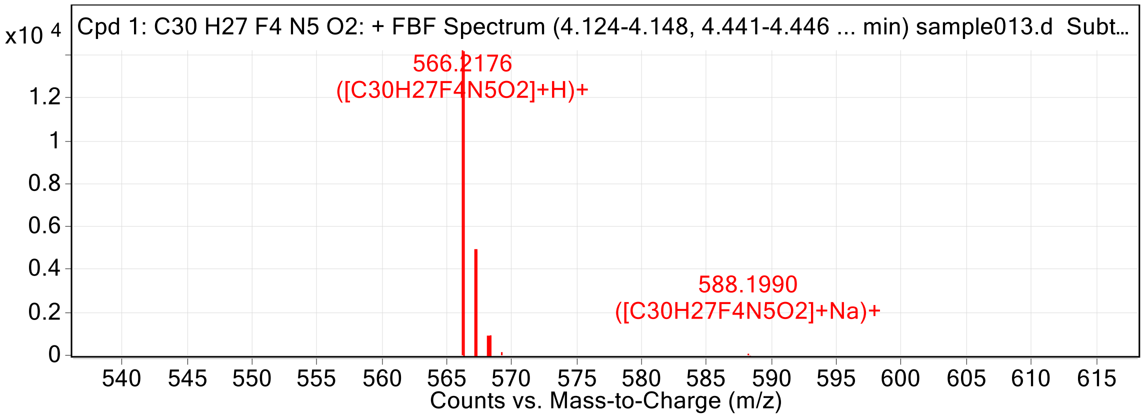

***4-{[4-(pyridin-3-yl-carbonyl)piperazin-1-yl]methyl}}-N-[3-(4-methyl-1H-imidazol-1-yl)-5-(trifluoromethyl)phenyl]benzamide* (28l)** was synthesized from compound **27l** and 3-(4-methyl-1H-imidazol-1-yl)-5-(trifluoromethyl)aniline **(a)** as a white solid. M.p. 96-100 °C. $^{1}$H-NMR (DMSO-$d_6$, 500 MHz) $\delta$: 10.68 (s, 1H), 8.67 (dd, $J$ = 4.8, 1.6 Hz, 1H), 8.62 (d, $J$ = 1.9 Hz, 1H), 8.31 (s,  H), 8.22 (d, $J$ = 1.3 Hz, 1H), 8.17 (s, 1H), 7.99 (d, $J$ = 8.3 Hz, 2H), 7.85 (dt, $J$ = 7.8, 1.9 Hz, 1H), 7.75 (s, 1H), 7.54 (d, $J$ = 8.3 Hz, 2H), 7.49 - 7.52 (m, 2H), 3.66 - 3.72 (m, 2H), 3.64 (s, 2H), 3.37 - 3.42 (m, 2H), 2.50 (br.s, 2H), 2.42 (br.s, 2H), 2.20 (s, 3H); $^{13}$C-NMR (DMSO-$d_6$, 126 MHz) $\delta$: 167.2, 166.4, 150.9, 148.1, 142.9, 141.8, 139.4, 138.4, 135.5, 135.3, 133.4, 132.2, 129.4, 128.3, 124.0, 115.4, 114.7, 112.1, 61.7, 53.2, 52.6, 49.1, 42.1, 14.0. HRMS(ESI+) m/z calcd for $C_{29}H_{27}F_3N_6O_2$ [M + H]+ 550.2148, found 550.2242. Purity: 98.12% (by HPLC).

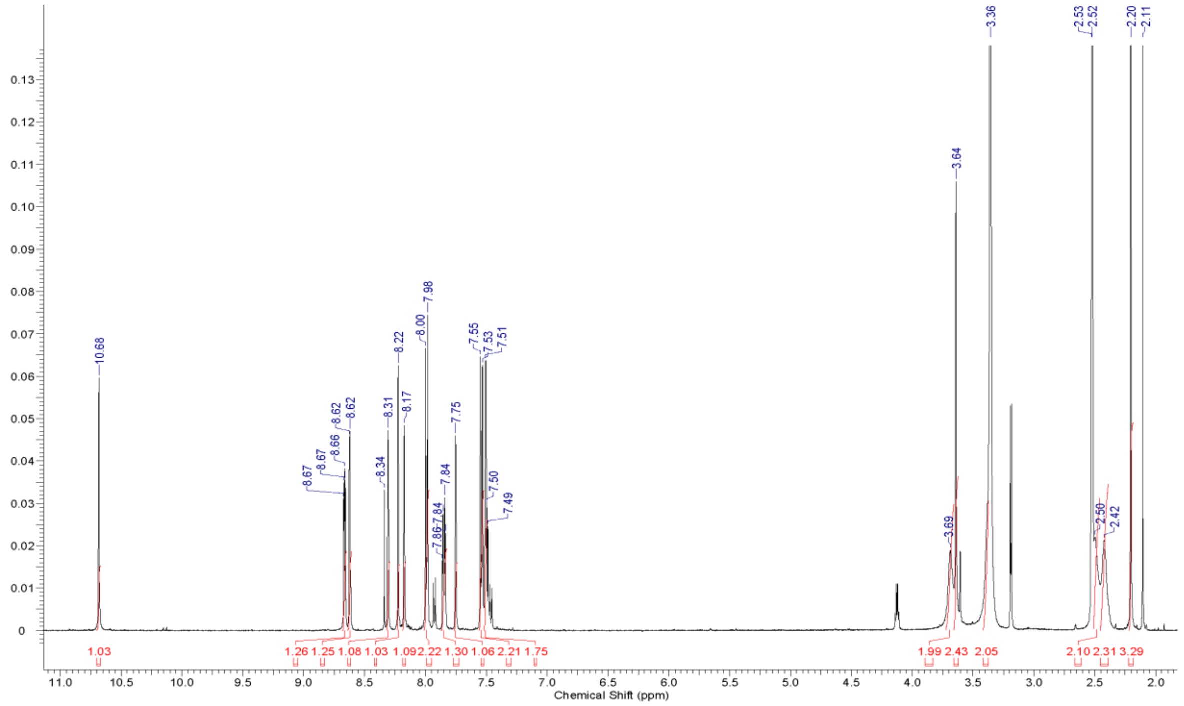


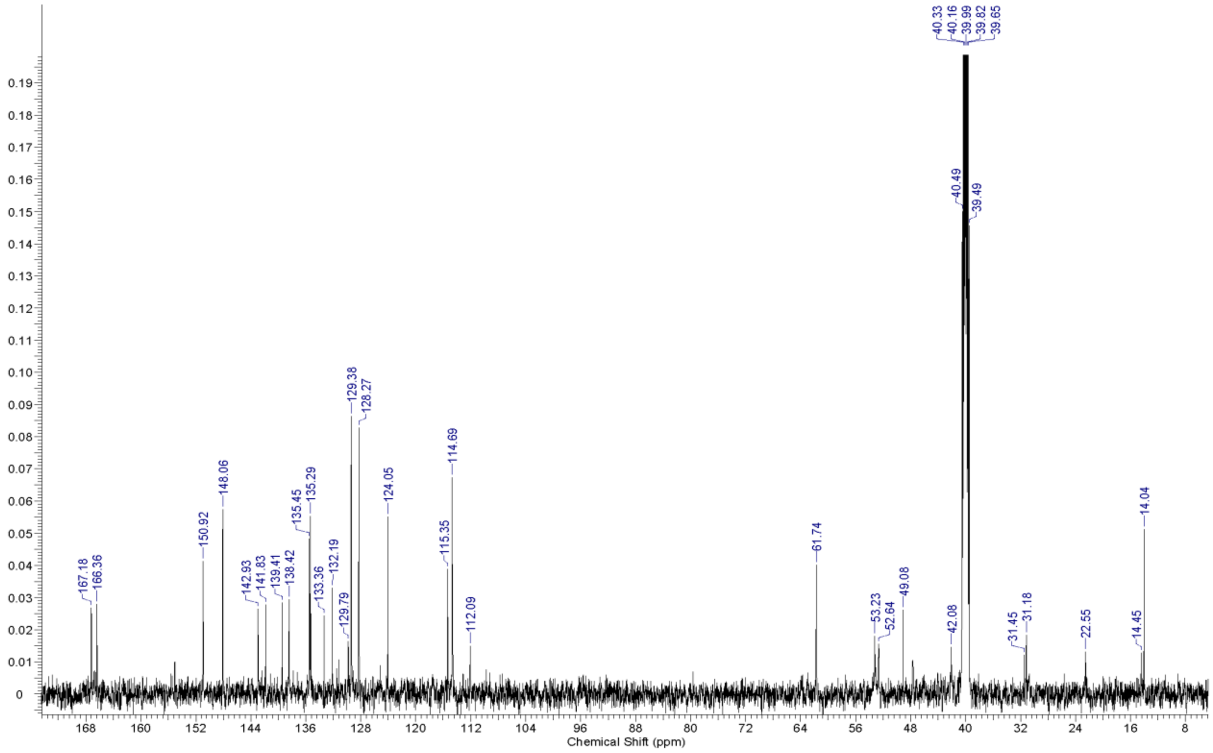

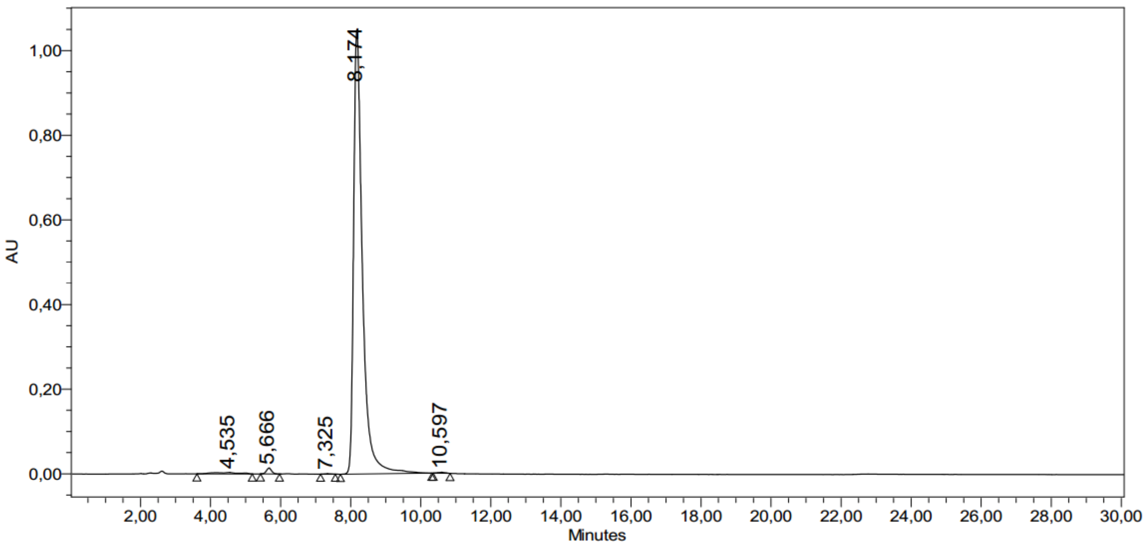


| | RT | Area | % Area | Height |
|---|---|---|---|---|
| 1 | 4,535 | 166278 | 0,89 | 3394 |
| 2 | 5,666 | 140762 | 0,75 | 13626 |
| 3 | 7,325 | 12230 | 0,07 | 958 |
| 4 | 8,174 | 18382530 | 98,12 | 1049217 |
| 5 | 10,597 | 33132 | 0,18 | 2220 |

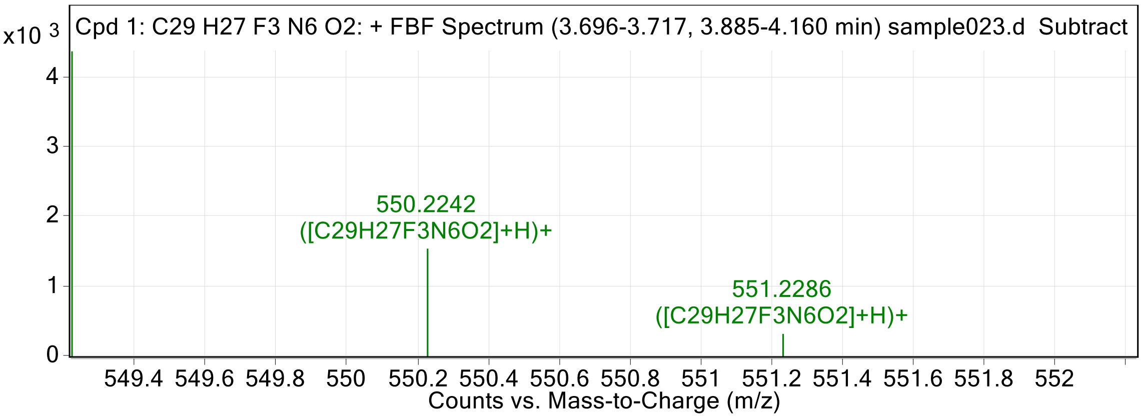

## Biological assays

3.3. *Cytotoxicity Assay*

Each *in vitro* experiment was performed at least in triplicate and the standard deviation of absorbance was less than 10% of the mean. For the *in vitro* assays, a stock solution (1% DMSO in the appropriate buffer with the tested compound diluted under sonication) was prepared from which several dilutions were made with the appropriate buffer.

Human cell lines were obtained from the Institute of Cytology, Russian Academy of Sciences. The cell lines studied were maintained in RPMI 1640 medium (AppliChem, Darmstadt, Germany) supplemented with 20% (HL-60, RPMI 1788) fetal calf serum (HyClone, Cramlington, UK), in DMEM (A 549) and Eagle's MEM (AppliChem) (MCF7, HeLa) with 10% fetal calf serum (HyClone). Cells were cultivated at 37°C in a humidified atmosphere of 5% $CO_2$. Cellular sensitivities to clofarabine and its lipid derivatives were measured with the MTT assay. Cells were plated in triplicate in 96-well plates ($10^5$ cells/well for suspended cell cultures and $5\times10^3$ cells/well for monolayer cultures); at the same day (suspended cell cultures) or on the following day (monolayer cultures), compounds were added at the appropriate dilutions. Plates were incubated under standard conditions for 48 h. Thereafter, 10 μL MTT (Sigma) in phosphate buffered saline (5 mg/ml) were added. The plates were incubated for an additional 4 h and 150 μL dimethylsulphoxide (DMSO) were added to dissolve the formazan crystals. The optical density was read on HALO MPR-95 Microplate Reader (Dynamica Scientific Ltd., Australia) at 570 nm. The antiproliferative effects were as degrees of inhibition of tumor cells (%).

*3.4. Kinase Inhibitory Assays*

The assay was performed using Kinase Selectivity Profiling System (TK-1+ADP-Glo™ Assay and TK-2+ADP-Glo™ Assay, Promega), where the kinase activity was measured by quantifying the amount of ADP produced during a kinase reaction. The assay is performed in two steps; first, after the kinase reaction, an equal volume of ADP-Glo™ Reagent is added to terminate the kinase reaction and deplete the remaining ATP. Second, the Kinase Detection Reagent is added to simultaneously convert ADP to ATP and allow the newly synthesized ATP to be measured using a luciferase/luciferin reaction. The luminescent signal generated is proportional to the ADP concentration produced and is correlated with kinase activity. Our compounds were diluted with Kinase Buffer (5% DMSO) and 1 mkL of the dilution was added to a 5 mkL reaction so that the final concentration of DMSO is 1% in all of reactions. All of the enzymatic reactions were conducted at 23 ℃ for 60 min. The 5 mkL reaction mixture contains 1μl of the Compound Solution, 2μl enzyme solution and 2μl Kinase substrate. After the enzymatic reaction, 5 μl of ADP-Glo™ Reagent (Promega, USA) was added to each reaction and incubate the plate for 40 min at 23 ℃. Than 10 μl Kinase Detection Reagent (Promega, USA) was added to each reaction and incubate the plate for 30 min at 23 ℃. Luminescence signal was measured using an Infinite M 200 plate reader (Tecan, Switzerland).

Kinase activity assays were performed in duplicate at each concentration. The luminescence data were analyzed using the computer software, Magellan™. The difference between luminescence intensities in the absence of Kinase (Lut) and in the presence of Kinase (Luc) was defined as 100% activity (Lut – Luc). Using luminescence signal (Lu) in the presence of the compound, % inhibition of kinase was calculated as:

% inhibition = [1-(Lut – Lu)(Lut – Luc)] x 100%,

where Lu = the luminescence intensity in the presence of the compound.

## Computational methods

*3.5. Docking*

Autodock Vina was used for docking studies [24]. Ligands from PDB-complexes were extracted by copying of HEATATM section of PDB-file. Two-dimensional structures were created using Marvin Sketch [31]. 3D-structures were generated using molconvert [32]. Conformations generation and structure minimization were performed with Open Babel [33]. Missing protein residues were restored with MODELLER [34]. GROMACS with AMBER FF99SB-ILDN force field was used for obtaining minimized receptors. Binding site coordinates of receptors were determined by the size of ligand in PDB-file and were scale-up by 20% in each dimension. Docking results were limited by only one docking pose having best docking score. Preparation of ligands and receptors for docking was carried out in MGL Tools [35]. Visualization of docking results and H-bond search was made in Chimera [36]. Two-dimensional interactions maps were generated by PoseView [37].

*3.6. Molecular dynamics*

Molecular dynamics was carried out using GROMACS package using AMBER FF99SB-ILDN force field. All ligands were parametrized with ACPYPE [38]. Simulation workflow was as follows: solvation, neutralizing and adding NaCl ions up to concentration of 0.15 mol, energy minimization, NPT and NVT equilibration steps 200 ps each and final 2 ns production run at 300K. Dodecahedron box of 1.2 nm and periodic bounding conditions were used. Berendsen thermostat was used for equilibration. Long electrostatic coupling was treated according to PME method. G_mmpbsa tool was used for MM-PBSA binding energy calculations [26]. To perform energy calculations every twentieth frame of final molecular dynamics trajectory was extracted skipping first 100 frames.
